# Why Latrines Are Not Used: Communities’ Perceptions and Practices Regarding Latrines in a *Taenia solium* Endemic Rural Area in Eastern Zambia

**DOI:** 10.1371/journal.pntd.0003570

**Published:** 2015-03-04

**Authors:** Séverine Thys, Kabemba E. Mwape, Pierre Lefèvre, Pierre Dorny, Tanguy Marcotty, Andrew M. Phiri, Isaak K. Phiri, Sarah Gabriël

**Affiliations:** 1 Department of Public Health, Institute of Tropical Medicine, Antwerp, Belgium; 2 School of Veterinary Medicine, University of Zambia (UNZA), Great East Road Campus, Lusaka, Zambia; 3 Department of Veterinary Tropical Diseases, Faculty of Veterinary Science, University of Pretoria, Onderstepoort, South Africa; 4 Department of Biomedical Sciences, Institute of Tropical Medicine, Antwerp, Belgium; 5 VERDI-R&D, Louveigné, Belgium; Dodowa Health Research Centre, GHANA

## Abstract

*Taenia solium* cysticercosis is a neglected parasitic zoonosis occurring in many developing countries. Socio-cultural determinants related to its control remain unclear. Studies in Africa have shown that the underuse of sanitary facilities and the widespread occurrence of free-roaming pigs are the major risk factors for porcine cysticercosis. The study objective was to assess the communities’ perceptions, practices and knowledge regarding latrines in a *T. solium* endemic rural area in Eastern Zambia inhabited by the Nsenga ethno-linguistic group, and to identify possible barriers to their construction and use. A total of 21 focus group discussions on latrine use were organized separately with men, women and children, in seven villages of the Petauke district. The themes covered were related to perceived latrine availability (absence-presence, building obstacles) and perceived latrine use (defecation practices, latrine management, socio-cultural constraints).The findings reveal that latrines were not constructed in every household because of the convenient use of existing latrines in the neighborhood. Latrines were perceived to contribute to good hygiene mainly because they prevent pigs from eating human feces. Men expressed reluctance to abandon the open-air defecation practice mainly because of toilet-associated taboos with in-laws and grown-up children of the opposite gender. When reviewing conceptual frameworks of people’s approach to sanitation, we found that seeking privacy and taboos hindering latrine use and construction were mainly explained in our study area by the fact that the Nsenga observe a traditionally matrilineal descent. These findings indicate that in this local context latrine promotion messages should not only focus on health benefits in general. Since only men were responsible for building latrines and mostly men preferred open defecation, sanitation programs should also be directed to men and address related sanitary taboos in order to be effective.

## Author Summary

Livestock owners from small scale farms are most vulnerable for Neglected Zoonotic Diseases (NZD) in developing countries and their risk behavior leads to more intense and complex transmission patterns. Studies in Africa have shown that the underuse of sanitary facilities and the widespread occurrence of free-roaming pigs are the major risk factors for porcine cysticercosis. However the socio-cultural determinants regarding its control remain unclear. We hypothesize that via a bottom-up culture-sensitive approach, innovative control strategies can be developed that are more adapted to the local reality and more sustainable than current interventions. By assessing the communities’ perceptions, practices and knowledge regarding latrines in a *T. solium* endemic rural area in Eastern Zambia, we found that more than health, seeking privacy underlies motivation to use latrines or not. The identified taboos related to sanitation practices are in fact explained by the matri- or patrilineal descent and because men are responsible for building latrines, sanitation programs should focus more often on men’s knowledge and beliefs. In order to contribute to breaking the vicious cycle between poverty and poor health among livestock owners in developing countries, disease control strategies should always consider the socio-cultural context.

## Introduction

*Taenia solium* taeniosis/cysticercosis is an important neglected parasitic zoonosis prevailing in many developing countries. The adult tapeworm lives in the intestines of humans, causing taeniosis, while the metacestode larval stage (cysticercus) usually develops in pigs following the ingestion of eggs excreted with the stool of tapeworm carriers, causing cysticercosis. Cysticercosis may also occur in humans upon accidental ingestion of eggs via faeco-oral contamination and may cause severe neurological disorders when cysticerci lodge in the central nervous system (neurocysticercosis, NCC) [1]. NCC is the most important parasitic neurological infection, to which almost 30% of acquired epilepsy cases are attributed in endemic areas [2].

Many surveys carried out in Africa have identified the general lack of and use of sanitary facilities as the major risk factors for cysticercosis [3–6]. Studies have demonstrated the positive effects of health education on the incidence of porcine cysticercosis in Tanzania [7] and on the prevention of epilepsy in Kenya [8]. However, an increased use of latrines could not be demonstrated. Many sanitation projects, implemented by governments or NGOs, which led to the construction of latrines in rural areas, faced refusal of the communities to use them and adopt safe hygienic practices [9–11] because the drives to motivate latrine adoption were often not identified and interpreted in messages and strategies to promote sanitation grounded in a given cultural context [12]. Unfortunately, in many African rural communities, open defecation practices were not adequately analyzed or taken into account before project formulation and implementation. Practicing open air defecation is linked not only to the presence or absence of water or latrines, but also to social and cultural determinants [13].

Improved latrine use as a control measure potentially has implications for many other sanitation-related pathogens [1,14,15], such as soil-transmitted helminths [16] and diarrhoeal agents [17]. According to the World Bank, 2.5 billion people worldwide live today without access to improved sanitation and 1 billion of these people practice open defecation. In sub-Saharan Africa, 70% of the population still lack access to improved sanitation, thereby indicating the urgent need for improvement [18].

Currently, *T. solium* control program managers need to understand *why* latrines are not used in endemic areas of Africa. Even though the significance of social and behavioral influences on the spread of human cysticercosis is known [7], culturally adapted control measures have not yet been implemented in endemic areas such as Zambia where the prevalence of *T. solium* cysticercosis in rural areas (in both human and pigs) is very high [19–22].

The objective of this research was therefore to assess the communities’ perceptions, practices and knowledge regarding latrines in a *T. solium* endemic rural area in Eastern Zambia, in order to identify possible barriers to their construction and use and to propose, eventually, adaptations of strategies to overcome cysticercosis, and other sanitation related diseases locally.

## Methods

### Study area

Focus group research was conducted in a rural area (Kakwiya) in Petauke district in the Eastern province of Zambia. The Kakwiya Rural Health Centre (RHC) has a catchment population of 11,344 (Clinic headcount records). People practice subsistence farming, growing mostly maize and groundnuts primarily for home consumption. Pig production is common; most households have owned pigs at least once to resolve financial issues.

The main ethno-linguistic group in this area is the Nsenga, which have a matrilineal descent. The district was selected based on reports indicating high porcine [20] and human cysticercosis prevalence, presence of a high number of free-roaming pigs, and reports of cysts observed in pigs slaughtered in backyards [21].

The Kakwiya catchment counts approximately 261 households and 138 individual toilets which is equivalent to an overall toilet coverage of 52.9%. The number of toilets varied quite markedly between villages (Table 1). There are no communal toilets as such. The sanitation facilities found in the study area were built following the simple pit latrine model. Completed, partially completed or abandoned, they generally consist of a pit dug into the ground, sometimes covered by a hygienic slab made from crushed stones and cement with a hole. Latrines were covered with a shelter (with or without a roof) and fitted simply with a sack or sometimes with a door.

**Table 1:** Latrine coverage in the study area (Kakwiya catchment).

No.	Village	Number of Households	Number of toilets	Latrine coverage (%)
1	Kakwiya	97	56	57.7
2	Chifwiti	22	10	45.5
3	Kambawino	1	0	0.0
4	Mzeka	5	4	80.0
5	Chonjo	8	2	25.0
6	Lubangu	3	1	33.3
7	Komboka	5	3	60.0
8	Sikabi	1	0	0.0
9	Maseya	1	1	100.0
10	Nsamba	15	6	40.0
11	Misolo	63	40	63.5
12	Chawala	1	0	0.0
13	Wonzi	1	0	0.0
14	Chiludzu	3	0	0.0
15	Mulembelembe	11	8	72.7
16	Chilima	1	0	0.0
17	Chingolo	2	0	0.0
18	Kalikeka	1	0	0.0
19	Maloba	9	2	22.2
20	Chisenga	3	2	66.7
21	Mutambanjeleka	8	3	37.5
	**Total**	**261**	**138**	**52.9**

### Study design

Twenty-one focus group discussions (FGDs) were conducted totaling 172 participants including 56 men, 58 women and 58 children (below the age of 18) from seven villages (Table 2). The seven villages were randomly selected from villages around the health center because of its central position. They were not included in recent biomedical surveys to avoid information and sensitization biases. Separate FGDs were held with men, women and children in each village since these groups have different perceptions and behaviors regarding sanitation (gender dependent) [11]. In addition, working with heterogeneous groups is likely to hamper the quality of the data [23,24]. For children, the FGDs were gender-mixed because, unlike adults, they were able to speak freely regardless of age and gender.

**Table 2:** Characteristics of the focus group discussions.

FGD No.	Village	Category	Number of participants		
			Male	Female	Village
1	Wonzi	Children	4	4	
2	Wonzi	Women		8	24
3	Wonzi	Men	8		
4	Chimphanje	Children	4	5	
5	Chimphanje	Women		8	25
6	Chimphanje	Men	8		
7	Sikalinda	Children	4	4	
8	Sikalinda	Women		8	24
9	Sikalinda	Men	8		
10	Nyazowani	Children	4	4	
11	Nyazowani	Women		8	24
12	Nyazowani	Men	8		
13	Chimanja	Children	5	4	
14	Chimanja	Women		8	25
15	Chimanja	Men	8		
16	Chiludzu	Children	5	3	
17	Chiludzu	Women		8	24
18	Chiludzu	Men	8		
19	Mtuna	Children	4	4	
20	Mtuna	Women		10	26
21	Mtuna	Men	8		

To ensure the validity of the data collected, FGDs have been conducted until reaching data saturation of the information from the seven different villages and from the three different subgroups.

### Data collection

The data collection took place from July to August 2010. Each FGD consisted of approximately 8 participants. Participants were selected from the villages based on their availability and willingness to participate. The FG discussion guide was pre-tested and fine-tuned in one FGD performed with male participants from a village outside the study area.

Three facilitators (a female nurse, a male environmental health technician and a male community health volunteer), all familiar with the Nsenga language, were identified and trained to moderate, observe and record the FGDs. The training consisted of a two-day course during which they were briefed on the study objectives and on FGD moderation skills. Facilitators switched roles for each discussion. All the FGDs took place at the Kakwiya RHC because of its central geographical location and practical aspects. To avoid biases related to the fact that the venue was not neutral in terms of health, the first set of questions was about general pig management.

The average duration of the discussions was about an hour. The following topics were covered: the perception of pig breeding in the communities, knowledge and perceptions of taeniosis/cysticercosis infection and related risk behaviors such as people’s latrine perception and reasons for not using latrines (defecation practices, latrine management, building responsibility, socio-cultural obstacles); and opinions on control measures.

All discussions were recorded on a video camera to facilitate the transcription of a discussion involving several individuals at the same time. Encouraged by our key informants, the use of a video camera was pre-tested and did not seem to be intrusive or affecting the discussions. The facilitator was always assisted by a reporter. To ensure the good implementation and follow up of the study, the main researchers (Séverine Thys & Kabemba E. Mwape) attended every discussion.

In this paper, only results pertaining to people’s latrine perception, reported practices and factors that lead to lack of use of these sanitary facilities are presented and discussed.

### Data processing and analysis

The FGDs were transcribed and translated into English by two research assistants and two researchers who took turns in both tasks. To improve interpretation reliability, the written transcripts were reviewed independently by the two same researchers before accepting them for analysis. The analysis of the transcriptions and the notes taken during the FGDs was supported by the NVivo 8 software (QSR International Pty. Ltd., Melbourne, Australia, 2008), which allows to classify and sort data; examine relationships and trends in the data. The major themes were separately identified through coding by the same two main researchers of the study following an inductive approach. Any differences were discussed until consensus was reached.

### Ethical considerations

Ethical clearance was obtained from the University of Zambia Biomedical Research Ethics Committee (003–02–10) and from the Ethical Committee of the Antwerp University Hospital in Belgium (10 03 3 704). Further approval was sought from the local authorities and community leaders before commencement of the study. Finally, before the start of each FGD, permission was sought from the individual subjects to enter the research and to video record the discussion. Written informed consent was obtained from each participant and from parents (or guardians) for children under 18 years old. Participation in the discussion was voluntary and no names nor pictures were recorded in the transcripts. Questions were appropriately phrased to avoid embarrassing people and also to tackle sensitive issues or taboos. FGDs with children took place after school hours.

## Results

The results highlight the different themes that emerged in the analysis. To reflect as much as possible what was expressed in the discussions, the order used to present the themes in each sub-sections reflects the level of importance given by the participants to these topics (going from a strong to a weaker consensus). No significant differences were observed between villages (very homogeneous), we indicate when the main ideas were mentioned across all the FGDs and where consensus or differences arose the most among the three different categories of FGDs conducted (men, women and children).

Results are illustrated with anonymous quotes, selected on the basis of their representativeness, appropriateness and revealing quality.

### Latrine availability

**Topic 1: Perceived presence and absence of latrines**. In this section, we describe how people perceived the presence and absence of latrines in their village in order to identify factors that explain latrine availability.

People generally referred as much to situations with as without the presence of latrines. On the overall, participants agreed on: 1) the general absence of latrines at home (*no latrines at home*, *no latrines for visitors*, *not yet completed*), especially women; 2) the presence of latrines in some homes (*latrines at home*, *shared and not shared with neighbors*) (acknowledged by all categories); 3) that latrines are public among neighbors, a perception mostly shared among men and women groups (Table 3). The distinction between having a latrine at home and the presence of latrines in the village revealed a distinction between private and communal uses of sanitation facilities.

**Table 3:** List of themes and sub-themes per topic (5) according to men, women and children groups.

**A. Latrine availability**				
Topic 1: Perceived presence/absence of latrines*	**Sub-themes**	**Men**	**Women**	**Children**
No latrines available	**No latrines at home (1)**		V	
	Not yet completed		V	
	No latrines in the village		V	V
	Latrines not shared	V		
	No latrines for visitors	V	V	V
	No latrines in the field			V
Latrines available	**Latrines shared (3)**	V	V	
	Not every household		V	V
	**Latrines at home (2)**	V	V	V
	Few latrines in the village	V	V	
	Many latrines in the village		V	

Topic 2: Obstacles to build latrines*	**Sub-themes**	**Men**	**Women**	**Children**
**Men’s responsibility not assumed (1)**	Men’s laziness		V	
	When a man gets married		V	
	Difficult for unmarried women		V	
	Men too drunk		V	
**Public use (other latrines available) (2)**		V	V	V
**Poverty (3)**		V	V	V
Not if not forced by law				V
How to build is unknown			V	
No people available to help			V	
Not enough education about the goodness of latrine		V		
Not a habit		V	V	V
**B. Latrine use**				

Topic 3: Open defecation VS Latrine use*	**Sub-themes**	**Men**	**Women**	**Children**
**Open defecation (1)**			V	
Latrine use			V	

Topic 4: Arguments in favor of the use of latrines*	**Sub-themes**	**Men**	**Women**	**Children**
**Participate to good hygiene (1)**	Protect pigs from eating feces	V	V	V
	Not washing hands after open defecation	V		
	A toilet is more hygienic (less dirty)		V	
	Not effective if not every household have a toilet	V	V	
	Prevent flies to contaminate food (with human feces)	V	V	V
	Discard all the bad things from the intestines in one pit			V
**Prevent diseases (2)**		V	V	V
**More comfortable (3)**	Not being disturbed by pigs pushing you before finishing	V	V	
	When you have diarrhea	V	V	
	Less far than going to the bush	V	V	
	When it’s raining	V		
More visual privacy	When the bush has been burnt (no leaves)	V	V	
	Less risk to meet relatives	V		
	Feeling free if the taboo is observed	V		
	When it’s night	V		
Give dignity		V	V	
Nothing good about open defecation			V	

Topic 5: Arguments NOT in favor the use of latrines*	**Sub-themes**	**Men**	**Women**	**Children**
**Taboos (1)**		V		
**Not building one latrine per house (2)**			V	
**Lack of privacy (3)**	Cannot hide yourself (more shy, feel not free)	V		V
	Latrine not well made	V	V	
	Be careful to not let blemish after use	V		
	Risk to see nakedness	V		
	Too nearby village	V		
Less convenient	Washing hands is not easier	V	V	V
	Create a queue	V	V	
	Easier to head to the bush			V
	Does not allow to check for worms		V	
	Need to carry things to clean the toilet	V		
Not a habit			V	V
Not comfortable	Scary for children (fall in the pit)		V	
	Be in contact with dirt (diseases)	V	V	V
	Flies	V		
	Smell			V
Punctual absence of latrines	When working in the field	V		V
	When emergency			V
	When traveling			V
When private use (lock at the door)		V		
Maintenance difficult	100% cleanness is impossible		V	
	Need to often replace the door	V		
	Pit is quickly full	V		
No more food for pigs		V		V
How to use latrine is unknown		V		V
Promotion of latrines failed		V		
Men enjoy more open defecation			V	

**C. Latrine availability**				
Topic 1: Perceived presence/absence of latrines*	**Sub-themes**	**Men**	**Women**	**Children**
No latrines available	**No latrines at home (1)**		V	
	Not yet completed		V	
	No latrines in the village		V	V
	Latrines not shared	V		
	No latrines for visitors	V	V	V
	No latrines in the field			V
Latrines available	**Latrines shared (3)**	V	V	
	Not every household		V	V
	**Latrines at home (2)**	V	V	V
	Few latrines in the village	V	V	
	Many latrines in the village		V	

Topic 2: Obstacles to build latrines*	**Sub-themes**	**Men**	**Women**	**Children**
**Men’s responsibility not assumed (1)**	Men’s laziness		V	
	When a man gets married		V	
	Difficult for unmarried women		V	
	Men too drunk		V	
**Public use (other latrines available) (2)**		V	V	V
**Poverty (3)**		V	V	V
Not if not forced by law				V
How to build is unknown			V	
No people available to help			V	
Not enough education about the goodness of latrine		V		
Not a habit		V	V	V

**D. Latrine use**				
Topic 3: Open defecation VS Latrine us*	**Sub-themes**	**Men**	**Women**	**Children**
**Open defecation (1)**			V	
Latrine use			V	

**(1)–(2)–(3)**: The three first themes or sub-themes which obtained the most important consensus among the 21 focus groups (indifferently from the group types)

* For each of the five topics, the themes and sub-themes are ordered from the most mentioned to the least mentioned ones, indifferently from the groups.

**V**: Themes and subthemes most mentioned in groups (men, women, children)

Participants further stated that a household with a latrine had dignity and respect as visitors, passersby or guests unaccustomed to using the bush, could easily be allowed to use the facility. A latrine therefore was a necessary feature of hospitality. This was especially highlighted by people whose household was situated in close proximity to the roads:

“There is dignity especially for visitors. When a visitor comes at home and asks to use a latrine, you easily point it out. That person may be happy not to go in the bush. There is respect at a home if there is a latrine”.

(Focus group\Men Mtuna village)

“… Having a toilet gives one high sense of respect and dignity”

(Focus group\Men Wonzi village)

Conversely, at village level, the presence of latrines (*latrines shared with neighbors, few and many latrines in the village*) was more mentioned than latrine absence (*no latrines in the village, not in the field and not shared with neighbors*), except among children FGDs who pointed out that if you need to defecate while you are working in the field, you do not have other options that doing it in the open.

Even if latrines were mentioned to be available in the community, men and women stated that there were few of them, and that sharing these facilities was a common practice.

“[…] But you find that in the village there are maybe five or six toilets and everybody rushes there. You cannot stop them. The toilet becomes for common use and therefore the traffic to the toilet is very high”.

(Focus group\Men Chiluzu village)

Finally, even if very few participants mention that some of the latrines were incomplete (Table 3), They expressed a certain willingness to build latrines, although it was not often considered a priority.

**Topic 2: Obstacles to build latrines**. From all the 21 FGDs, eight obstacles (Table 3) were identified as contributing to the lack of latrines.

Because the Nsenga observe traditionally a matrilineal descent, a newly married couple lives in the wife’s relative’s household and the custom implies that when a man gets married, he ought to build his own latrine because of the taboos of a man sharing a latrine with his parents-in-law (see paragraph on taboos). In this cultural context, the responsibility of latrine construction clearly belongs to men but a constraint mentioned by the participants was that men did not consider the construction of a latrine for themselves as a priority. This lack of motivation was mostly explained (mainly by women) by the fact that some men were lazy, or preferred to spend time drinking alcohol.

The existence of other latrines in the village was the second consensual argument raised by the participants to explain the non-prioritization of latrine construction. Indeed no taboos were observed for sharing latrines with people from another household or with non-relatives. Participants commonly stated that it was also not well accepted that the toilet owner refused access to other community members. Refusal could create conflicts or have negative consequences on social relations. In addition, some male and female participants recognized that refusing access to neighbors would reduce all the benefits of having a latrine (prevent diseases, prevent pigs from eating human feces, prevent contamination of kitchen utensils) by forcing people to defecate in the bush or near their homes. The hesitations expressed about the placement of locks on latrine doors and its implications for the sanitation of the village reflected the tension between private use (leading to eventual envy, jealousy, no more benefits for the community health) and communal use (leading to increased latrine cleaning and maintenance, rapid filling of the pit, sharing the cost and responsibility). In both kinds of use, the risk of disrupting interpersonal relations was a potential obstacle to start constructing latrines.

As expressed by one man participant:

“… you would find that you are a newly married couple. It is just you and your wife only, you have no children, and then you decide that instead of me going to the bush let me build a toilet. Once you finish building the toilet you realize that your neighbor has a large family. Then you think that if I allow these people to use my toilet it will soon be full so you look for a lock and start locking it like in town. But after some time you realize that these pigs we keep will eat their human waste since they do not have a toilet and affect me as well. You stop locking your toilet.”

(Focus group\Men Chiluzu village)

A third obstacle mentioned was the lack of means to construct a latrine. This was an important constraint and commonly identified in the three groups. It was stated that some people could not afford to build a latrine of good quality materials, according to the local standard criteria of a latrine (roof, proper door, walls high enough,…) pursuing the gain of visual privacy. The physical appearance of the latrine had a bearing on whether it was used or not regarding the level of privacy offered (see latrine perceived advantages). However, local materials were often not of sufficient quality.

A fourth and fifth constraint highlighted in our study were the lack of knowledge on how to build latrines and the lack of awareness on their advantages for some participants. They pointed out that educating people about the benefits of a latrine would eradicate all the misunderstandings or erroneous conceptions. Men insisted more on the need of more “persistent” and “sustained” sanitation education campaigns, women made more reference to the hygienic benefits that campaigns would result in.

The 6^th^ reason described by some women is that unmarried women were facing great difficulties to have latrines built since the construction of latrine is a man’s responsibility.

“Those hired persons refuse. I started a long time ago, since my latrine collapsed. I am not used to be frequently going in the bush. I usually start to construct another on the moment I realize the one I am using has become half full. However, nowadays the hired men refuse; you may have money and tender it. They would say, “Why don’t they get married so their husband can do it for them?”

(Focus groups\Women Mtuna village)

Finally, the last obstacle raised to latrine construction was that it was simply not yet considered as a habit to defecate in a toilet.

### Latrine use

**Topic 3: Latrine use versus open defecation**. Even when latrines are present, going to the bush in order to defecate in the open is a common practice, and a culturally accepted norm in the area. It appeared that men were the ones enjoying more to defecate in the open (see obstacles to use latrines) than others.

As a general finding about pros and cons of latrine use, more comments against than in favor were mentioned during the FGDs.

**Topic 4: Arguments in favor of the use of latrines**. Participants manifested a strong consensus that latrine use contributed to a better hygiene and prevents diseases. Additionally, greater comfort, dignity and increased privacy were mentioned. When exploring the benefits of sanitation within communities and households, the last common argument shared, especially among women was that “*there is simply nothing good about open defecation”* (Table 3).

**Latrine use contributes to good hygiene**. All groups and especially women considered that the presence of a latrine ensured hygiene in a household mainly because it prevented pigs from eating human feces, and avoided them contaminating kitchen utensils left on the ground with dirt and feces that could bring diseases (see next section).

Men and women also pointed out that, as long as all households had no latrine, no benefits would be realized, as many would still be openly defecating.

Another common perceived advantage was the prevention of food contamination by flies, as by using latrines, all human feces would be gathered in one pit instead of being everywhere in the open.

According to some comments from children, latrines were the place where you could “*discard all the bad things from the intestines*”.

“… Because after eating the food we need to get rid of the waste material so if we have a toilet we do all that in the toilet.”

(Focus group\Children Nyazowani village)

**Latrine use prevents diseases**. It appeared that participants connected the use of latrines with their own improved health but not always straight line.

“We prevent diseases, since pigs can’t get into the toilet to eat our feces.”

(Focus group\Women Chimphanje village)

They alluded to the fact that latrines prevented diseases in general and some specific diseases as cholera or dysentery by preventing pigs, flies and unwashed hands to contaminate food with human feces.

There were linkages with the risk of diarrhea and HIV transmission only when participants referred to the pigs’ habit of eating feces of sick persons.

“If a pig eats feces of an AIDS patient and they come to feed from your plates, and then the other person without AIDS comes to feed from that plate without washing it, he will contract the disease.”

(Focus groups\ Men Sikalinda village)

**Latrine use is more comfortable, provides more privacy and increases dignity**. In the FGDs, there was a strong consensus, especially among women, that “*one advantage of using latrines was not being disturbed by pigs pushing you before finishing*”.

Adults stated that latrines were more often used when someone was suffering from diarrhea. Afraid of not reaching the bush on time and be embarrassed in front of fellows, they would rather use latrines (indicating a matter of comfort and convenience rather than family or personal health protection).

W1: “Another disadvantage of not having a toilet is when you have diarrhea, it is embarrassing and difficult… and you can’t go to the neighbors, worse still it may be too late to get to the bush. When you are in the bush you find that a pig is even waiting for you to finish, I can’t manage that”.

W2: “Worse if you live in the middle of the village, you’ll mess yourself up before you even get to the bush.”

(Focus groups\Women Sikalinda village)

Mainly for men, using latrine offered a greater comfort when they were situated closer than the bush and when it rained.

It was also very important for many to avoid being seen defecating in the open, especially men, by the opposite sex or by their in laws:

“The other advantage of having a toilet is that sometimes you run to the bush to go and help yourself… then you find that your mother in law is just squatting a few meters away from you. Because you are in such a hurry you do not see her and you help yourself but even if she has finished she will not leave until when you leave. In the case of a toilet at home people will see that a person has entered the toilet and nobody will come until when you are through posting your mail. That is when they also can write their letters and come to post them.”

(Focus groups\Men Mkopeka village)

Another major factor in favor of latrines, mentioned by participants in the context of privacy, was the seasonal availability of good defecation sites around the village. In the dry season, the bush was usually burnt for agricultural purposes, making them not dense and high enough anymore to hide villagers who wanted to defecate in the open.

Some participants, mostly males, revealed that it was quite more convenient to use latrines at night. As latrines did not always have a proper door, using it at night will avoid others to see you.

“…You know that our toilets do not have proper doors like those in town so during the day if you go in the toilet children may find you squatting helping yourself. But in the night it is better no one can see you.”

(Focus group\Men Chiluzu village)

At night, latrines presented the additional advantage of reducing the risk of being exposed to hazards in the bush.

Along with the seeking of more privacy, participants further stated that the use of latrine gave more dignity in the sense that you could hide from the others when defecating:

“Toilet should be one of dignity, not going to the bush”

(Focus group\Women Mtuna village)

**Topic 5: Obstacles to use latrines**. Thirteen reasons were identified for not using latrines (Table 3) in order to facilitate the flow of the reading, some themes are grouped together. The greatest consensual reasons among all FGDs that arise were: 1) the taboos related to sanitation practices, 2) the fact that not all households had a latrine and 3) the fact that latrines did not offer enough privacy resulting in a loss of dignity for the user.

For women and children, the main factor that leaded to not using latrines was the unavailability of the facilities, while for men traditional taboos seemed to be the central issue.

**Latrine use entails cultural taboos**. In general when the different socio-cultural obstacles for the use of latrines were addressed, most of the comments were made by men. This showed that men were much more concerned about the respect of taboos than the other two groups.

Moderator: “What is a bad thing that can arise from people sharing a latrine, even for example with an in-law?”

M1: “You may meet each other at the latrines when both are coming from different locations as you know how the intestines work, for example when an in-law is coming from the field and may be unaware that the in-law had gone to the latrine”

M8: “You may not be aware of it”

M1: “When you meet each other at the latrines, it becomes embarrassing”

M8: “It is like you have been undressed”

(Focus groups\Men Mtuna village)

As such, other people, especially children, are not allowed to see their parents or adults go to the latrine.

In the study area, traditional taboos meant that the head of household (father) could not share the same latrine with his mother-in-law, his children-in-law, older children (adults) of his own household, his grown-up daughters and his younger children when the risk to be seen was too high or when young children will use the latrine just after their father.

Often, men went to the bush pretending to go to the field, gather firewood or hunt mice (a common delicacy in the region) not to be seen entering a latrine by children.

“What we are saying is true and still happens, where you see a man picks up his axe on the shoulder and goes to the bush pretending that he is going to fetch fire wood and yet he is going to help himself, especially now that the grass has been burnt. You come back carrying a piece of fire wood just to hood wink the children when your main purpose was to go to the toilet”.

(Focus group\Men Chiluzu village)

It seemed that these taboos were strongest between in-laws and in particular between mothers and sons-in-law.

“I would be right to say we do not use the toilet the way we should because here we have a lot of respect for each other. Our culture does not allow us to use the same toilet with your grown-up daughters, son or daughter-in-law, mother-in-law and all other people. What has killed us in the villages are these cultural norms which we have clung to for so long. Our friends in town find nothing wrong with all this. They all use the same toilet in the house.”

(Focus group\Men Chiludzu village)

Bypassing these prohibitions was considered as a lack of respect and decency similar to being seen undressed. For this reason, some of the participants suggested having two latrines at the same household. When asked if they had no problems being seen going to the latrine in full view of their daughter in-law, a male participant stated:

“Yes, it is a problem, because your daughter in-law would find that may be you miss the hole and shit on the side or she would find a very big heap and start saying this man really shits. In our communities that does not show respect. It is always a good idea in that case to have two toilets.”

(Focus group\Men Mkopeka village)

On the other hand no taboos seemed to be observed between parents and very young children, between wife and husband, between women and neighbor’s children, in town and with neighbors as they often did share latrines in the community. Sons were freer to share the same latrine with the head of the family than daughters. Fewer taboos were observed between people of the same gender.

Although the origin of those taboos and reasons to observe them were not very explicitly explained in the discussions, in one way or another all the further arguments against the use of latrines developed in this section were linked to the importance of respecting those sanitation taboos.

Compounded by the existing taboos, women considered that if not every home owns at least one latrine, the practice of open defecation will not end and no benefits will be realized. Despite the men also stating this and acknowledging the benefits that would arise from the use of latrines, they were still the major obstacle towards the construction of more latrines (see above).

**Latrine use causes a lack of privacy and is less convenient and comfortable**. At the first sight, this sub-section can look contradictory with the advantages foreseen earlier by the participants regarding the use of latrine. However when participants were asked why people did not use latrines, some responded that most of the available latrines were not in a very good state. The walls were too low, they lacked a roof and a lockable door (many only had a cloth or a sack as a door) thus compromising privacy. This lack of privacy mainly mentioned by men, included the fear of leaving dirt after the latrine use as well as the risk to see nakedness. They also mentioned that latrines were often built in the center of the village, which prevented people from using them because they would be seen entering or leaving them.

The convenience perception about the use of latrines was not unanimous and presented also different opinions. It appeared that for some women and men the use of latrines was not necessarily more convenient than open defecation to fulfill its benefit of improving hygiene. First, because it was not easy to wash hands after the use of the latrine (no water supply nearby) and secondly, because it was not convenient to carry material to clean the latrine.

For some men especially, the few latrines available created quite rapidly a queue, which was not convenient in case of an urgent need (e.g. diarrhea). In addition, the queue led people know that you need to defecate (risk to be seen). Complete cleanness of such public latrines, needed to fulfill the required social norms of privacy, convenience and comfort, was also impossible to achieve due to maintenance difficulties. The most important reason was to be exposed to dirt, bad smell and flies. It was also mentioned to be a scary place for children (dark, big hole wherein they could fall,…).

According to some children, it was simply easier to go to the bush to relieve oneself also because it was more difficult to find a latrine when people work in the fields or when they were travelling.

One woman mentioned that the way the latrines were built (pit latrine) did not allow checking for worms and could delay the identification of a parasitic infection. Another inconvenience to use latrine according to some men and children, was that it did not allow pigs to feed on their feces. Open defecation was a common and affordable solution for the pig’s owner to face feed shortage.

Finally, more children than women admitted that using latrines was simply not a habit and that men from the older generation manifested a strong reluctance to build latrines.

**Limited knowledge on latrines**. If preventing diseases was an argument in favor of the use of latrines, it was not always evident for the participants that adequate maintenance was one of the most important determinants to ensure the health benefit of a latrine. According to the men it was difficult to teach children how to use a latrine properly. Also, equipment sometimes freely distributed by sanitation programs was not always properly used.

“In addition there was indeed a program which was making stand plate for the toilet and giving them to people for free. Some have stored them in their houses and some are using them to stand on them when they are taking a bath. They do not use them for the purpose they were intended for. There is indeed a need for sustained education in this area of our day living.”

(Focus group\Men Chiluzu village)

Mainly men considered that latrine promotion failed, as it did not convince them to construct and use latrines properly.

## Discussion

Considering the results presented in this manuscript, it is clear that the transmission of *T. solium* can easily continue in this very suitable fecal contaminated environment where few infrastructure for safe excreta disposal are available and correctly built or used and therefore allowing free roaming pigs to maintain the lifecycle in such endemic area.

While poverty may be a contributing reason for the lack of latrines in many communities, it does not explain why some people continue to practice open defecation long after their community has been provided with water points and learned about latrines and hygiene practices [25].

Choosing latrines means changing defecation practices and because sanitation behaviors tend to be strongly culturally conditioned, we chose to discuss our results mainly through user lenses [12] and socio-cultural lenses [26]

Like Jenkins and Curtis (2005) in Benin, we found among the arguments in favor of using latrines, drives related to prestige (gaining more respect and dignity from visitors), to well-being (better hygiene by protecting pigs from eating feces, more comfort by not being disturbed by pigs pushing you before finishing, more visual privacy) and situational drives (when it’s raining, when bush has been burnt, when you have diarrhea).

But from a consumer perspective, these authors also demonstrated through their model of motivation for latrine adoption in rural Benin that at least one drive is needed (among the 11 that they found) to motivate real changes in sanitation behavior as long as barriers do not suppress the expression of this specific drive [12].

Out of our FGDs, several obstacles for building and using latrines were identified such as sanitation taboos (avoiding sons and mothers-in-law to share the same latrines), the lack of privacy (latrines not well built, the queue) and the lack of comfort (too scary for children, flies, smell). It means that in our studied context the different obstacles identified would need to be addressed first in order to arise sufficient intensity for positive drives to be translated in concrete action taken by the target population.

Complementary of Jenkins and Curtis’ model, Avvannavar and Mani’s conceptual model of people’s approach to sanitation [11] can let us better understand the interplay of socio-cultural factors that determine how people take care of their primal urge.

In the category “culture” and “fear & superstition”, their model offers us the opportunity to discuss the taboos we identified related to sanitation practices. For the authors, attitudes and beliefs about revulsion to feces vary between cultures. Examples are numerous in Africa. In the Akan culture (Ghana) for example, the word “shit”, is as taboo as the thing itself and people when going to the bush to defecate, need to wear a blinder pretending that they will not be seen if they see nobody [27]. In Uganda, sharing latrines with in-laws is a taboo and the use of latrines could affect women’s fertility and also cause miscarriage [28]. In the Eastern Cape Province of the Republic of South-Africa, human feces were found in the bush because people were afraid to share latrines to avoid being bewitched [9].

However our results demonstrate that the type of descent, matrilineal or patrilineal, is also an important factor that has a significant influence on the sanitation practices of a community.

In our study, although no taboos seem to be observed between wife and husband, between women and neighbor’s children, in town or with neighbors, a man could not share the same latrine with his mother-in-law, his children-in-law, older children (adults) of his own household, his grown-up daughters, his younger children.

Similar taboos have been reported in a number of other African communities, reflecting important social norms. In Eastern Cape Province (RSA), for instance, stakeholders stated that people prefer to defecate in the bush because sharing a latrine as a father-in-law with his daughter-in-law is perceived as a disgrace [9]. In the case of a sanitation program (Community-Led Total Sanitation program, CLTS) introduced in a Kenyan district, the taboo for a father-in-law’s feces to mix with those of his daughter(s)-in-law was also described resulting to gender-segregated open defecation sites in the forests [29].

The origin of the taboos we identified and reasons to respect them are not very explicitly explained by the participants. It seems however logical in a matrilineal society to observe very strict proscribed behaviors towards the maternal in-law’s in order to limit contact and ensure respect. Mary Douglas in “Purity and danger: An analysis of the concepts of pollution and taboo” explained that the father in the matrilineal Trobrianders and Ashanti is credited with being an involuntary source of danger; he is an intruder [30]. If we look at the previous examples from patrilineal societies, the reported latrine practices taboos (Father and daughter-in-law) are in fact simply reversed in our setting (Mother and son-in-law) because we are in a matrilineal society.

Furthermore, being in a matrilineal or patrilineal system allows us to better understand firstly, the gender division of tasks about latrine construction and secondly, the social norms in such societies in terms of privacy, both having a strong influence on why latrines are not built or not used.

Seeking privacy, in general and from in-laws in particular, seems to be the main underlying motivation for people to use or not latrines. Using latrines when natural vegetation does not suffice to hide in the bush contextualizes and highlights the importance given to privacy and the fear to be seen going or openly defecating, especially by relatives. Embarrassment and shame that occur if being seen is the expression of a transgressed norm that underlies a number of taboos related to the matrilineal descent of this community. The perceived benefits of a latrine depends on the way it has been built and its construction is in turn strongly linked to the respect of socio-cultural sanitation practices. In our setting the benefit would be not to be seen (privacy) in order to feel free, less shy and respectful towards cultural taboos. However, the actual sanitation situation entails no or badly constructed latrines that do not offer enough privacy (e.g. no proper door, too small walls, no roof, rickety superstructures) and therefore also contributes to why latrines are not used. Avvannavar & Mani (2008) consider that the human tendency to seek privacy is the modified animal behavior attributed to the deep primal territorial tendencies. In our research, we can apply this theory as such: “By not willing to be seen or to see you when you are defecating” could be another way for men in a matrilineal system to mean that “by not defecating in the same latrine of my mother-in-law, I show respect about her territory as I am a stranger in her family”.

In general, the topic of sanitation was mostly developed by female participants. Women were more spontaneous and free to speak about latrine issues and related sanitary behaviors. The particular social, economic and political structures in most African contexts make women more concerned about sanitation and domestic duties than men [31] who carry out construction and maintenance of facilities according to the gender division of tasks. That explains why the common practice in the water and sanitation sector is to involve women, not only as a target group, but in the organization of activities at the local level [32]. In our study however, resistance to abandoning open defecation practice was mainly expressed by men. As the role of latrine construction belongs to male participants, addressing men’s knowledge and beliefs could benefit sanitation programs in many ways.

If men do not see latrine construction/use as a priority or if they do not know how to build it, latrine coverage and use will not raise. In this specific situation, not getting married can be a handicap for women to have their own pit latrine built.

In regard to sanitation taboos, in certain cases, taboos themselves can be used as arguments in favor of the use of latrines in order to facilitate even more their respect. In the CLTS study in Kenya for instance, where the targeted communities are observing quite similar cultural norms and defecation practices as in our study, the facilitators were able to break one of the defecation taboos by showing that a pit latrine located within the homestead will complicate the task for an intruder who sought to bewitch others by accessing their intended victim’s feces (less discrete, difficult to dig up the feces) [29].

### Conclusions

The existing challenges of cysticercosis control in endemic regions require a “people-centered” preventive approach that addresses both the perception of the disease and its management. Control strategies should also be directed to the patterns of people’s behavior associated with the phases of transmission of the disease [33]. In this specific study we focused on people’s perceptions, knowledge and reported behaviors regarding the use and the construction of latrines.

Out of our findings, several entry points for promoting the use of latrines were identified and discussed.

Seeking privacy and taboos were both identified as the key factors influencing the possession and use of sanitation facilities. These findings reinforce why latrine promotion messages should not only focus on health benefits.

Some taboos can be explained by the type of descent (matri- or patrilineal). By acknowledging that the descent is also a factor that influences sanitation behaviors and regulates a number of norms and practices, we can more easily anticipate the type of taboos that could entail the adoption of hygienic practice related to sanitation.

A concrete proposition that could be made is to start building per homestead gender specific latrines instead of household specific latrines, each of them located in two different places to respect privacy.

But unless program planners are not totally convinced of the necessity to direct interventions not only at women but at men as well and focus also on men issues (practices, beliefs and knowledge), latrine building and use will not be efficiently promoted. Our results also stress the importance of anthropological studies for an in-depth understanding of sanitation practices within particular contexts in order to enhance the design of adapted interventions.

## Supporting Information

S1 Dataset FGD transcriptions. (ZIP)
