# Supplementary material for: Why Latrines Are Not Used: Communities’ Perceptions and Practices Regarding Latrines in a Taenia solium Endemic Rural Area in Eastern Zambia
Source: PLoS Negl Trop Dis. 2015 Mar 4;9(3):e0003570. doi: 10.1371/journal.pntd.0003570 (PMC4352092; doi:10.1371/journal.pntd.0003570)
Supplement: S1 Dataset — (ZIP) [file pntd.0003570.s001.zip › FGD transcriptions_Zambia-2010/Chiluzu_Mr Sakala/Children_Chiluzu_04-08-10.docx]

**Title: Focus Group CHILDREN**

**Date:** 04/08/10

**Site:** Kakiwa Rural Health Center

**Village:** CHILUZU  **Location:** Petauke district, Eastern Province, Zambia

**Duration (total time video): 53 min - Start:** 11:57 am **- End:** 12:46

**Participants:** 8 children

**Informed consent:** signed and available

**Moderator:** Mr Benjamin Mvula **Recorder:** Dr Andrew Phiri

**Transcription/translation:** Dr Sakala

**Abreviations: M: moderator, R: recorder, I: intervener, C: children, W: women, M: men**

**Disposition (from the left of the Moderator):**

Gender distribution:

**C3-C7-C8**: females

C1-C2-C4-C5-C6: males

Age composition:

C5: the youngest

Typology:

C5-C7-C8: shy/quiet

C3: dominant among girls

C2: expert

C6: dominant among boys

**M**

C1

C2

C3

C4

C5

C6

C7

C8

**R**

**Comments about participants:**

**Group dynamics:**

*Introduction*:

M: The first thing we need to do is knowing each other, my name is Benjamin Mvula

C1: Lameck Mwanza

C2: Kennedy Chirwa

C3: Phiri

C4: Agrey Phiri

C5: Esnart Phiri

C6: Weluzani Phiri

C7: Judith Mwanza

C8: Aliness Phiri

M: This group is composed Phiris!

I (Dr. Mwape): Are you related?

M: The things we are going to discuss are not supposed to be discussed with people who are not here. So all things we shall discuss end here, but all of you should feel free to talk you do not need to lift your hands when you want to say anything. I want us to discuss how you keep pigs, I am sure you all know pigs.

*All nod their heads.*

*Discussion*:

M: Why do you keep pigs?

C7: Pigs are good because if there is no money for school requirements we sell a pig and get the money to pay for our fees.

M: Anyone else?

C2: You can sell a pig and use the money to plough your field.

M: Anyone else?

C1: The goodness of the pig is that if there is a funeral you slaughter a pig and use the meet to feed the people.

M: C8 the goodness of rearing pigs?

*Silence*

C8 is very shy.

M: The bad part of a pig?

C4: The badness of the pig is that it eats human feces.

C2: After they multiply and swine fever strikes, all of them would die.

M: Anyone else?

C6: If it eats feces from the bush and come to eat from the plates, if the feces had a disease we would also suffer from the same disease.

M: Do people keep pigs in your village, all of you?

All: Yes.

M: How do you keep these pigs, are they left to fend for themselves?

C6: We build them a kraal.

M: Do they stay inside during the day and night?

C3: Some stay outside.

C6: Some stay outside

C3: After they are let out in the morning they stay outside.

M: Anyone else?

*Silence*

M: What is the goodness of keeping the pigs in a kraal?

C4: They do not eat feces or any dirt they find but only feed on the food we give them.

M: Anyone else?

*Silence*

M: The goodness of keeping pigs in a kraal, what I mean here is that they are kept inside day and night?

C2: The goodness is to prevent diseases.

M: Like which disease?

C3: Cholera

M: Anyone else?

C6: To prevent them eating dirt so that they do not contaminate us with malaria.

I (Dr. Mwape): How does a pig bring cholera?

C6: It can eat feces from the bush and when it comes home it would eat from the plates which we use.

M: Anyone else?

*Silence*

M: We keep pigs in our family, how do we know that these are our pigs?

C2: We know them.

M: How do we know?

C3: Others cut a bit of the ear.

M: Others?

C3: Others just master the colour of the pig.

M: And still others?

C6: There are certain appearances and the colour that we would use to recognise our pigs.

M: Anyone else?

*Silence*

M: Some of you are not talking, I had said at the beginning that we should all participate and be free to discuss. Are you all free?

R enters the room to take his sit.

Dr. Mwape and the moderator ask the participants to contribute to the discussion without fear of anything.

I, M: You must all contribute to the discussion tell us what you know and do in your own words.

M: In the rearing of pigs what problems do we find?

C4: In some cases you find that the pig goes to eat some ones maize in a field.

M: Anyone else, C8?

*Silence*

C2: It would go into the bush and eat someone’s food.

M: Good, Anything else?

C1: In the process of its moving up and down it would drink someone’s bathing water.

M: How do we know that this pig is sick?

C6: When it comes home it is not active, you would notice that it is always sleeping and it loses appetite.

M: Anything else?

C2: If you give it food it would not eat.

M: How about others how would you know that this pig is sick?

C3: Appearance.

M: How does a sick pig look like?

C3: If it was fat it would start getting thinner.

M: It gets thinner? C8, how would you know that this pig is sick?

*Silence*

M: Okay let us continue, like I said let us be free to say anything, when people choose you I am sure they trusted that you would contribute. What do we feed the pigs?

C5: Maize bran

M: Anyone else?

C6: We collect *nsembe* from the maize mill we mix with water and give the pigs.

M: Anyone else?

C4: Pumpkins.

M: Anyone else?

C1: Maize.

M: Anyone else?

C3: During the season of mangoes we give them mangoes.

M: Anyone else?

*Silence*

M: We live with these pigs, what do you give them to eat?

*Silence*

M: Who feeds these pigs in the homes where we come from?

C3: Sometimes grandmother feeds them.

M: Anyone else?

C2: Anyone who stays at home.

M: Anyone else?

*Silence*

M: Don’t our parents feed the pigs?

C2: Yes, our parents, when they are at home they feed the pigs.

M: When we all have gone to school our parents give them food don’t they?

All: They give them.

M: Where does the food we feed our pigs come from?

All: It is our food.

M: It is our food?

All: Yes.

M: What is your role in the rearing of pigs at home?

C3: When a pig is slaughtered, we eat the meat.

C2: Our parents would not hesitate to sale a pig if we had no money to pay for our school.

M: What is our role in the rearing of pigs?

C2: When you are at home you get maize bran and feed the pigs.

M: Others, C8?

*Silence*

M: Do you have pigs at home?

C8 Just shakes her head in refusal.

M: C7 since we started you have not said anything, is there a problem?

C1: She is just shy.

M: Now, C7 I am asking you, do you have pigs at your home?

C7: *(Shakes her head.)*

M: Even if you do not have pigs, you are a pupil you must know something?

C1: When I am at home I go to the field, bring some pumpkins, break them and feed them to the pigs.

M: How about others?

C6: You can also feed them cassava.

M: Okay, what do we like to do in pig rearing?

*Silence and smiles*

M: Like us children what do we like to do in the rearing of pigs?

C2: I like to feed them.

M: Anyone else?

C1: I like to give them water.

M: How about others?

*Silence*

M: You do not help in putting them in the kraal for the night?

All: We do

M: Why did you not mention that?

*Laughter*

M: Are there any of your relatives who help you in the rearing of pigs?

C3: Yes they are there.

C2: Yes like if you are not there, they would feed them

M: Those who do not come from your house, they help you?

C2: Yes

M: Is there any where, where we get assistance in the rearing of pigs?

C3: No, there is nowhere.

M: How about at home who makes that decision that this pig should be slaughtered today?

C3: It is our father.

M: Any other contribution?

C6: We are told to catch it when we want to slaughter.

M: No I mean who makes a decision which pig should be slaughtered; do we also make that decision?

C4: Yes if we were also feeding the pigs we can also contribute to making the decision to slaughter the pig.

C2: Yes because sometimes we know the animals well.

M: How many times do we feed the pigs?

C3: Three times

M: Three times a day?

All: Yes

M: What is good about feeding the pigs maize bran?

C2: They get fat

C3: That they should be satisfied so that they do not go into the bush to eat feces.

M: Okay, anyone else?

C6: That they should grow up well.

M: Anyone else?

C1: So that they should get fat.

M: Anyone else?

*Silence*

M: You can see that you are now answering the questions, let us pick it up.

C4: So that they can breed well.

M: What is bad about giving the maize bran?

*Laughter*

C3: There is nothing bad

M: Thank you very much. How do we feel when we see a pig eating feces? *(Pointing at C8)*

C8: I do not feel alright

M: You do not feel alright, how do you feel?

C8: I feel like vomiting.

M: Any other?

C6: Sometimes when you are eating *nshima*, you feel the smell of feces then you realise it is the pig which has brought the smell then you chase it away.

M: Anyone else?

C1: It makes feel like puking because it eats all things that are not good.

M: What good is there if the pig is eating feces?

All: There is nothing good.

M: How about the badness of the pig is eating feces?

C4: Maybe it’s the day when we want to slaughter it and that is when it feeds on feces, and then you find that we also participate in eating feces.

M: So we start eating our own feces?

*Laughter*

M: Anyone else?

*Silence*

M: Anyone else, C5?

C2: Sometimes it would eat feces and come to eat in the plates.

M: Do we manage to keep pigs in the kraal during the night and during the day?

C3: We do not manage.

M: Why?

C6: We do not care ourselves.

M: Why?

C6: Some manage some do not manage.

M: Are there some who manage to keep pigs in the kraal day and night.

C4: Yes they are there, those with food.

C3: Some after the rain season they let them free.

M: After they let them out what do they eat?

C3: That is when they go to eat feces.

C2: They go into people’s fields and scavenge on whatever they can find from post harvest.

M: That is why I asked what is good about pigs eating feces, because if they do not have food to feed them and they eat feces they would not die, so is that not a good thing?

C3: No, that is not good.

M: Do we eat pork regularly?

All: Yes

M: When do you eat pork?

*Silence*

Moderator repeats the same question.

M: is there any time when we eat pork?

C2: Any time when a pig is slaughtered.

M: When do we slaughter pigs to eat?

C4: During Christmas.

M: Eeeh! How about others?

C3: During a funeral.

C4: On the New Year’s Day celebration.

M: Anyone else?

C3: During celebrations, during a marriage ceremony.

M: Where does this pork come from?

*Silence*

M: Do we buy, are they our pigs or where does it come from?

C3: We just buy.

C1: Sometimes we just slaughter our pig.

M: sometimes you are just given, is that not so?

All: yes

M: Is this pork examined?

C3: No it is not examined.

M: They do not examine after slaughter?

C3: No, it is not examined.

M: Why?

C3, C4: There are no inspectors.

M: Do you know that Mr. Mvula can examine pig meat?

All: We do not know.

M: You do not know?

All: Yes.

M: Now you know so when you slaughter a pig you can call him?

All: Yes

M: Will you be calling him?

All: Yes.

M: Would you love that your meat should be inspected?

All: Yes

M: Why?

C3: To know whether it has a disease or not.

M: If you find out that it has a disease, and I recommended that we need to burn the meat or burry the meat, would you agree?

C3: Yes we would agree to have the meat burnt or buried.

All: Yes

M: You would not hate me?

All: No, we would not hate you.

C6: Some would not agree they would think you are cheating them; therefore as soon as you leave they would share the meat.

C4: In that case then it would mean one pig may kill more people, if they disobeyed your expert opinion.

M: How do we want our pork prepared, cooked, roasted and dried?

C2, C4: Cooked meat.

M: Why?

C1: Because when it is cooked and it is fried, you check the pot and see how well it has been cooked you start salivating.

*Laughter*

M: How about others?

*Silence*

M: C5, what type of meat do you like best, cooked, roasted or dried?

C5: Cooked

M: Why?

C5: Because of the fat.

M: Does it mean roasted and dried meat has no fat?

All: It has but we just enjoy cooked pork.

M: Roasted pork has no fat?

C3: It has but usually roasted meat does not cook.

M: How does your mother cook the pork when they buy it?

C1: They cook.

M: They cook all the time?

C1: Yes

M: All the time?

C1: Yes, they cannot manage to roast for the whole family.

M: At least they roast for your father?

All: They would roast for him if he wanted.

M: Have we ever seen *nsembe* in pork?

C1: Yes.

M: *Masese*, have you ever seen them

C1: Yes

M: *Masese*, do we know them?

C3: Yes they are found in pork.

M: C8, have you ever seen *masese* in pork?

C8: Shakes her head to signify no.

M: Do you eat pork?

C8: Yes.

R: You have never seen *nsembe* in pork?

Some of the panel members say they have never seen *nsembe*, even when their friends try to explain how they look.

M: Now if for example we know that this meat has *nsembe*, would we feel nice to eat that meat?

All: No

M: You eat the meat despite knowing that the meat has *nsembe*?

C6: Some of the people would not eat the meat themselves, but they would take the meat and go and sale it to people who do not know.

M: They sell the meat with *nsembe* to others? How about others?

C1: If we see that it has *nsembe* then we know that the meat has a disease so we throw it away.

M: Others?

*Silence*

M: Do we have toilets where we come from?

C3: Some have got, some do not have.

M: Those without toilets, where do they go?

C1: In the bush.

M: They go to help themselves in the bush, is that good?

*Laughter*

C3: That is not good, because the pig would be watching and no sooner than they leave than the pig would be eating their feces.

M: Now when the pig eats, what happens?

C1: Then the pigs would eat in the kitchen utensils after eating the feces.

M: What is good about going to the bush?

C4: Some of the toilets in the village are not well made because sometimes you find it has no door, so those who are shy they usually go to the bush.

M: Are we free when other people see us go into the toilet?

C3: No.

M: C2?

C2: It feels uncomfortable.

C3: You cannot be free.

M: Why?

C3: I feel shy.

M: Why do we feel shy?

C1: Because once people see you going that way they always start thinking that he has gone to shit.

M: Is it bad to shit?

C1&3: No.

M: Is it a sin to go and shit.

All: No, it is not a sin.

M: Then why do we feel shy?

C1: It is because we want to respect ourselves.

M: To respect oneself, how?

C6: If you go to the bush you may think that no one is seeing you but some people would actually be seeing you.

M: Let us talk about self respect, the one who goes in the bush or the one who goes in the toilet?

All: The one who goes in the toilet.

M: Why?

C3: Because people would not see him.

M: The one who goes in the bush people would see him?

C3: Yes

M: Any other?

C6: People would see him.

M: How about pigs?

C3: They would follow him.

M: So the one who goes to the bush has not respected himself, is it not so?

All: Yes.

M: Then why are we not free to go to the toilet?

C4: Sometimes the feces we find on the rim of the toilet make you feel shy.

C6: Sometimes it is because of the way the toilet is made. Sometimes it’s just a sack which is used as a door so when you are squatting and there is a person who is just passing by; they would see you squatting down. Sometimes someone also wants to use the toilet and peep they would see you squatting down that is what makes most people shy.

M: Are we free to go in the same toilet as our parents?

C3: It is rather difficult to go in the same toilet as your father.

M: Why?

C4: Because you may be meeting him each time you go to the toilet.

M: So if you met him, is that a sin?

C4: It just does not look right.

M: why do you feel shy?

C6: To meet your father.

M: Do you think your dad does not go to the toilet?

All: Considering the respect in our culture, that is not okay.

M: When we come from the toilet do we wash our hands?

All: Yes we do.

M: What time?

C3: Soon after coming from the toilet.

M: How about those who go to the bush, do they wash their hands?

C6, C3: Some wash and some do not wash.

M: The water we use, where does it come from?

C3: From the wells.

C6: From the bole holes.

C3: But in our village we do not have bole holes

M: Before we eat anything do we wash our hands?

All: Yes.

M: when we eat fresh maize do we wash our hands?

C3: Yes

M: Let us be truthful, do we wash our hands?

All: Yes we do.

C1: Before we even touch the maize we must wash our hands.

M: Are you saying the truth, how about when eating groundnuts?

All: We wash our hands.

M: Why?

C6: We are scared of diseases

Mod Why do we have to wash our hands C5?

C5: Maintain cleanliness.

M: C3, why do we wash our hands before we start eating?

C7: We are scared of diseases.

M: Why do we wash our hands before eating *nshima*?

C7: We are scared of suffering from malaria.

M: Any other thoughts?

C8: We fear to contaminate our food if we go to the bush.

M: Have we ever heard of worms?

All: Yes

M: Have you ever seen them?

All: Yes

M: Have you ever seen them in a grown up person, a child or even in our own feces?

C6: Yes

M: Do we know worms, have you ever heard of fits?

C6: Some are bewitched and some it just comes.

Moderator distributes pictures of stool with proglottids.

M: Have you ever seen these?

R: Where have you seen worms?

C7: In the feces.

R: Whose?

C6: In person’s feces, he called me to come and see what was in his feces, that is when I told him it was worms.

M: Others?

*Silence*

M: How do we know that a person has got worms?

C2: You eat very frequently.

C6: You experience Stomach pains.

M: Anymore?

C3: Unnecessary movement in the stomach.

M: How would you know that the child has worms?

C3: The stomach appears swollen and hard.

M: C7, you do not see these children with a hard stomach?

C7: I see them.

M: How would you know that this person has got worms?

C1: The person would complain of stomach pains every now and then.

M: Where do these worms come from?

C3: Some say they come from rape.

C6: Some say they come from eating soil.

M: Anyone else, C7?

*Silence*

M: C8?

*Silence*

M: C2?

*Silence*

M: Is there medicine for worms?

C3: Yes, it is there.

M: Where is it found?

C3: At the clinic.

M: Anyone else?

C1: Yes the medicine is there.

M: C3 said the medicine is found at the clinic, what do others say?

*Silence*

M: Can we prevent worms?

C2: By coming to the hospital if we notice anything that is indicating that you have worms.

M: How about others?

C1: If you come to the hospital they would examine you and find the problem, then they would prescribe treatment for you.

M: Anymore thoughts?

*Silence*

M: How do you think toilets can prevent worms?

*Silence*

M: Are we afraid to have worms?

C4: They kill.

M: Anyone else C7?

C2: You are not comfortable because you are always in pain.

C6: If you go to the hospital and they give you medicine which makes you vomit, they say that you should not bite it when vomiting otherwise you die.

M: Is there a disease that we know that is more dangerous than having worms?

C4: AIDS.

M: Anyone else?

C1: Fits.

M: Anymore?

*Silence*

M: Where do fits come from?

C6: Some are bewitched.

M: Anymore?

C1: Some are born with it.

M: Still others?

C2: Some if they go and steal and if the owner of the item that stole put medicine they can suffer from fits.

M: C8, where do fits come from?

*Silence*

M: Do many people in the village have worms?

All: Yes.

M: If a person has worms, is it a problem?

All: Yes.

M: How?

C2: The person does not look happy.

M: Why is it a problem if a person has worms?

*Silence*

M: C8?

*Silence*

M: C3?

*Silence*

M: If a person has worms what follows?

C6: Some would die.

C2: Some children do not grow up healthy.

M: And others?

*Silence*

M: How about small children when they have worms what happens?

C1: They have a swollen stomach.

M: Anymore?

C7: Some the stomach rumbles too much.

R: Have you ever seen in feces of a child or your friend things that look like a wood borer or shaded skin of a snake?

*Silence*

R: Did you understand the question?

C2: Yes

*Silence*

C4: We did not understand.

R: Have you ever seen in a child’s feces or your friend, pieces that look like a wood borer or like a shaded skin of a snake?

C1: We have never seen that.

C6: Some of us have seen that.

C1: I have also seen something like that.

M: *Masese* or *nsembe* do you know them?

All: Yes.

M: How would we know that this pork has *nsembe*?

C7: When you are cutting the meat you would find the *nsembe* in the flesh.

C2: Some would check the mouth before slaughtering it and would see *nsembe* in the mouth.

M: *Nsembe*, is it a disease for animals or for human beings?

C1, C2: It is a disease for animals.

M: Anymore?

*Silence*

M: C7, *nsembe* is it a disease for animals or people?

C7: Shake her head

It is difficult to say what she is trying to say

M: Is there a person we know who had this disease?

C6: No one.

M: How would we contract this disease?

C6: If you eat pork which has *nsembe*, we can contract the disease.

M: How does this disease come?

C2: By feeding pigs with *nsembe*, that is why even the disease is called *nsembe*.

M: Anyone?

C7: When the pig begins to slim down it can develop *nsembe*

M: C8?

*Silence*

M: Can we prevent *nsembe*?

All: Yes.

M: How?

C2: If you notice that pork has *nsembe* you do not eat the meat.

M: How do we prevent it?

*Silence*

M: What did we say about the toilets?

*Silence*

M: Or the toilets do not help in anyway?

C4: They help.

M: How?

*Silence*

M: Can this disease be cured?

C6: It can be cured.

M: How?

C1: If you do not give them *nsembe*.

M: Are we afraid to contract this disease?

C2: Yes we are afraid, because we must be scared of all diseases.

M: Is there a disease which you are afraid of which surpasses *nsembe*?

C4: Fits, HIV and AIDS.

M: Anyone else?

*Silence*

M: Is it a problem to have *nsembe*?

All: Yes.

M: What is the problem?

C2: That is one has a disease.

M: Anymore?

*Silence*

M: If you have *nsembe* what is the problem, C8?

*Silence*

M: If a person has *nsembe* what problems would that person have?

C6: The person would be sad because they may not know what to do.

M: Any More?

C3: They may not have a good body.

M: Anymore?

*Silence*

M: Let us say it this way if the government says all the pigs should be kept in the kraal day and night and they should be fed in there, would that be a problem?

All: No

M: Can we manage?

C3: The problem would be food.

M: Can we manage?

C6: We would stock pile the bran.

M: Can we manage say we had twenty pigs?

C4: We can manage because we want to prevent diseases at our home.

M: If the government says every home should have a toilet, can we manage?

All: We can manage.

M: Why are we now not managing?

C6: Because most people are used to go to the bush.

M: Now those who are used to go to the bush, can they manage to use a toilet?

C1: They can manage because the law has been decreed by the government.

M: If there is no such law does it mean these people cannot have toilets?

C4: We can have toilets.

C3: They cannot, because they need to be forced.

M: What if we who are here grow up and have our own homes. Can we manage to have toilets at our homes?

C4: We can manage

M: Why?

C6: It is the law to have a toilet at home.

M: Does government force people to have toilet?

C6: No it does not.

M: What is the use of a toilet at a home?

C4: When you have a toilet at home if there is a visitor he goes to the toilet because they may not be aware of the bushes you go to.

M: The visitors of those people without toilets where do they go to?

C4: The visitor goes into the bush.

C6: They either go in to the bush or maybe to a nearby toilet if a neighbour has.

M: What if the neighbour also has no toilet, what happens?

C6: Then the visitor also goes to the bush.

M: So the visitor is shown the bush?

*Laughter*

C1: Sometimes you just escort the visitor to the bush.

M: What if they said all pork should be boiled thoroughly well, Can we manage?

All: Yes we can manage.

M: Why?

C4: That is when it tastes nice.

M: Anyone else, can we manage, C7?

C7: Yes

M: Why?

C3: We can manage to prevent diseases.

M: What if they say all pork should be examined, can we manage?

C3: We can manage.

M: Can we?

All: Yes we can manage.

M: Why are we not managing now?

C4: Because there are no inspectors.

C2: The inspector is not there.

M: What if the inspector examined the meat and finds that the meat is not fit for human consumption and recommends that the pig be thrown away, can we agree?

All: Yes we can.

C2: Because when we come to the doctor we know that if it found with a disease it will be destroyed or thrown away.

M: We cannot go and take it after the doctor is gone?

All: No.

M: Do we manage to clean vegetables?

C2: They manage.

M: Even pumpkin leaves you manage?

C2: Yes.

M: Tell the truth, do you clean pumpkin leaves before cooking them?

All: Yes we wash them.

M: Let us now talk on personal hygiene, how many time do we bath in a day?

C1: Twice.

M: Twice?

C1: Yes.

M: Anyone?

C2: Three times.

C6: Three times?

C3: Yes, in the morning, in the afternoon and in the evening.

M: Anymore?

*Silence*

M: Why do we bath?

C4: So that the body can be soft.

M: Others, why do we bath?

C3: To open up the poles on our skin, which close if we do not bath.

M: If they close what happens?

C3: You fall sick.

M: what disease?

C4: Malaria.

M: How about others?

C1: If you do not bath you do not look nice.

M: Is hygiene a good thing to someone’s body?

C1: Yes

M: Why?

C1: Because you look clean.

M: Why is cleanliness good to a person?

C6: Makes the body smooth, and if you do not take a bath your body feels itchy.

M: How about others?

*Silence*

M: The cleanliness we are talking about is even washing your hands when you come from the toilet or the bush?

C4: They wash the hands.

M: When they come from the bush?

C4: Yes.

M: Do they manage to wash the hands when they come from the toilet?

C6: They do

M: If the government says that there is medicine for worms, can we go to get treatment?

All: Yes we can go.

M: If we saw someone suffering from worms, can we bring them to the clinic?

All: Yes.

M: What if the government said all pigs should come for immunisation can we manage to bring them, would there be a problem?

All: Yes we can bring them.

M: Would there be a problem?

All: There would be no problem.

M: If the government said that for this vaccination of pigs you have to make a contribution towards the buying of medicine, can you manage?

All: Yes.

M: Why?

C1: Because we want to protect them from diseases.

M: How about others?

C6: So that they can grow well and be smooth.

M: Anyone else?

*Silence*

M: Since we started we have talked about many things, about pigs, the reason why we rear pigs, who feeds the pigs and also about possible control measures. If there is anyone with a question they can now ask. Thank you very much for coming.
