# Supplementary material for: Why Latrines Are Not Used: Communities’ Perceptions and Practices Regarding Latrines in a Taenia solium Endemic Rural Area in Eastern Zambia
Source: PLoS Negl Trop Dis. 2015 Mar 4;9(3):e0003570. doi: 10.1371/journal.pntd.0003570 (PMC4352092; doi:10.1371/journal.pntd.0003570)
Supplement: S1 Dataset — (ZIP) [file pntd.0003570.s001.zip › FGD transcriptions_Zambia-2010/Chiluzu_Mr Sakala/Men_Chiluzu_04-08-10.docx]

**Title: Focus Group MEN**

**Date:** 04/08/10

**Site:** Kakiwa Rural Health Center

**Village:** CHILUZU  **Location:** Petauke district, Eastern Province, Zambia

**Duration (total time video): 92 min - Start: - End:**

**Participants:** 8 men

**Informed consent:** signed and available

**Moderator:** Mr Emmanuel Mwanza **Recorder:** Dr Andrew Phiri

**Transcription/translation:** Dr Sakala

**Abreviations: Mod: moderator, R: recorder, I: intervener, C: children, W: women, M: men**

**Disposition (from the left of the Moderator):**

Age composition:

M6: the oldest

M8: the youngest

M5: young

All the rest are middle age (grand-fathers)

Typology:

M8: shy

**M**

M1

M2

M3

M4

M5

M6

**R**

M7

M8

**Comments about participants:**

**Group dynamics:**

*Introduction*:

Mod: I would like to welcome you all the to Kakwiya clinic and thank you for coming to this discussion, our discussion will be about pig rearing and of course some other livestock will be discussed in the process. I would also like to urge you to speak up during the discussion when you are contributing so that you can be easily identified that it is so and so who is speaking. But before we start our discussion lets get to know each other, me who will lead this discussion my name is Emmanuel Mwanza I am one of those who help with livestock service here.

M1: I am Lawrence Phiri, one of those involved in livestock keeping

M2: I am Felix Mwanza I come from Chiludzu village, I am also among those who rear livestock like pigs.

M3: I am Francis Mwanza I come fro Chiludzu village I also am involved in the rearing of livestock.

M4: Iam Daniel Phiri I come from Chiludzu village I have come here to learn about pig keeping, and I also keep some livestock.

M5: My name is Mike Mwanza I come from Chiludzu village.

M6: I am Stanslaus Mwamba, I come from Chiludzu village I am an elder for Chief Nyamphande.

M7: I am Henry Mwanza; I come from Chiludzu village I am mainly involved in crop farming.

M8: I am Jackson Zulu I come from Chiludzu village.

*Discussion*:

Mod: Like I said before in my opening remarks we should all be free to contribute to the proceedings. My first question is do you think pigs are necessary to rear in the villages?

M4: Yes pigs are necessary to keep in the villages because they help us when we have problems like paying for school fees, when there is a funeral and also to use as relish at home.

Mod: What is the other good thing about pigs?

M6: The other good thing about pigs is that they multiply very easily.

Mod: Any other addition?

M2: The other good thing about pigs is that during the rainy season you would sale a pig and use the money to pay hired labour to assist with weeding in the field and also use the money to redeem you fertiliser, this way I find that pigs help us in many ways.

Mod: Any more additions?

M5: The good thing about pigs is that it is a good relish, it has very good meat that is very tasty and with very good fat and the meat is plenty. That is, my brothers, in my own opinion the outstanding advantage of a pig.

Mod: Any more thoughts?

M7: The other advantage of pigs is that they multiply very quickly. Let us take an example of a goat. The goat only give one or two kids per year while a pig can give several piglets and if swine fever does not attack them then in a short time you would have a very good herd.

Mod: Now let us discuss the bad side of pigs, what would you say is bad about a pig?

M2: The bad thing of pigs, say like we keep them in our village where we take little care about them they can eat human waste.

Mod: Ay other addition, M8?

M8: Pigs bring diseases.

Mod: Any thing else?

M4: The other bad part of pigs is that those who suffer from fits if they eat pork, they will immediately get a seizure so the medicine man usually stops them from eating pork.

M5: To add to what has been said, you may be aware that here most people have no pit latrine, so most people go into bush to relieve themselves. You would find that as the person is sited helping himself the pig will come running almost pushing him of so that it can eat the human waste. The same pig would go and drink water from the same spot where the women draw water for home use. In turn the women would come and draw water from the same spot and take it home. Same water would be used as drinking water. Now imagine that this person who was helping himself in the bush was suffering from a diarrhoea disease like dysentery and also if the same pig found kitchen utensils out side and ate from there and your wife, without cleaning the plate gave you food using the same plate, then you would all be sick. That is what I find very bad about a pig. It brings a lot of diseases especially diarrhoea diseases. That is why when you think about these things you almost want to stop keeping and eating pork.

Mod: Any other contributions?

M1: Like everyone else has said, the pig is a carrier of various diseases and here in the village the meat is not inspected but the meat has a wonderful taste. May be if the meat was inspected it would help.

Mod: So what caution would you give to those who eat pork?

M7: If we can look after them better, make them better pens and keep them in an enclosure and not let them out to scavenge.

M2: In addition the pig is a carrier of many diseases like my friend already pointed out so the diseases cannot finish, as long as we continue keeping the pigs the way we do.

Mod: Any thing else?

*Silence*

Mod: What problems do you find in the rearing of pigs?

M2: The problems we face in the rearing of pigs especially during the hot season are that their death rate is very high when swine fever attacks. We lack medicines to treat them when they are sick.

Mod: Any more thoughts on the problems you face?

M1: When the pigs multiply as you have already said that they produce more litter per sow, the owner usually fail to provide enough food and therefore you find them all over scavenging and eat anything they come across and even go to eat from other people’s fields.

Mod: Any other contributions?

M3: Like we have already stated the pig multiply very fast and one would be comfortable and say I shall depend on my pigs, but because of the way they eat, they will go in the bush eat human excreta contract a disease and they all die, especially in the coming hot season. Then you make a total loss.

Mod: Any other problems that you see?

M2: The other problem we see is that when they have multiplied in number, owners do not provide pens for them, so most pigs just roam in the village and owners just identify them when they see roaming about but they do not know what the pigs eat and where they stay.

M5: The other difficulty we face is that as you are aware pigs have a very big appetite and because most owners are poor they cannot afford to feed them, they just roam about. If the wonder into someone’s field especially those with nearby fields the pigs will eat the maize in a manner which you may not like. We therefore face a lot of cases from the owners of the fields.

Mod: How do you keep you pigs, do you keep you pig like free range or you keep them in a fence so that they do not roam around?

M6: We keep pigs that are free range and can roam anywhere. We do not fence them in.

Mod: Any other contribution?

M4: Yes we keep free range pigs but we usually keep them in a pen for the night. In the morning we give them maize bran and let them out to scavenge, then in the evening a child calls them in and give them maize bran. But they are not kept in a fence.

Mod: Now since you mention that you keep pig which are free range what bad things do you experience with this method?

M6: There are many bad things that we see with this method. First, they wonder into people’s fields and cause a lot of damage to their crops. And because the room anywhere they eat all kinds of stuff and bring a lot of diseases in the community

Mod: Now why is the major problem that stops you from constructing good pens and fences to keep them in?

M1: It is because people attach very little value and they do not care. Otherwise I do not see any problem that hinders us from keeping the pigs in an enclosure.

M5: The other problem I see is that because of the pig’s huge appetite we may not manage to keep them in a fence and feed them.

Mod: What is the major problem for you to make pens for the pigs?

M3: The major problem is that some people will decide to keep a pig because they have seen neighbour rearing pigs. Now they would not have planned how they are going to care for the pig, whether they have the capacity to buy feed for the pig. So when they fail to feed the pig and are afraid that the pig will die they let it out to fend for itself.

M5: Sometimes what causes all this is poverty because sometimes you would have very little money enough to buy a chicken but then you negotiate and buy a pig. Since the pig has a potential to multiply very quickly you invest in so that when they multiply you can have a bit more cash, but all this is due to poverty.

Mod: Now since all of you would be keeping pigs and you let them roam anywhere, how do you know that this is my pig?

M2: To know that these are my pigs, because like it has been said in the evening they are called to the pen by the children. These pigs are also born in that home steady so as they grow we know which ones are yours. Even the pigs themselves know which home they come from as they are growing. What also help is that they get used to their home when they are called every evening.

M1: Sometimes when these pigs are born they are given a mark, like you cut off an ear lobe, cut off the tail so that even when you catch it by accident you would know that this is someone’s pig. There are people who know how to mark animals. Even goats are given identification mark these days.

*Laughter*

M5: In addition these pigs look very different, some long in length, some have short snout, and some have a long and thin snout, the colour differs also. To a point that we know who owns what type of pig even if you found it very far you would know that this pig belong to Mr. Telegugu.

M1: The other thing is that not everybody in the village would rear pigs. In a village you may find that only three to four families rear pigs, so when you see a pig you immediately know who owns that pig. It is different from chickens which are reared by so many families. Also some families rear their pigs very well so you immediately recognise them from others.

Mod: Now, these pigs that you keep under the free range system what do you think they eat?

M8: They scavenge.

*Laughter*

M8: They eat *nshima* remains and human excreta.

Mod: Do they feed on remains from *nshima* or what do you think they eat?

M3: They usually feed on human excreta and some other fed they can scavenge. If they do not get human excreta they follow the people in the fields. During the planting season they come to the field and eat the seeds we plant. If it’s during the time when maize is ripening they come to the fields and eat the maize.

M4: During this time after the harvest they go to the field and eat the bit of sweet potatoes which are left during the harvest and then in the evening we call them and they are fed with a bit of bran and we pen them in for the night.

M5: In addition as it as already been discussed on this forum, pigs usually thrive on human waste. They act like the council in the villages because they are the ones who eat all the rubbish that is thrown away and even human excreta for those who have no toilets. They eat anything that is thrown away.

*Laughter*

Mod: What role do the men play in the rearing of pigs in a home?

M4: The role of a man in the rearing of pigs is that when you want to buy a pig, the man builds a kraal made of wood, thatch it then put the pig inside. We do not build a pen with brick and cement like the commercial livestock farmers do and we do not put a fence around the kraal.

Mod: Say in a family how do you help in the rearing of pigs?

M2: In a family we also contribute in the rearing by finding money and buy some bran from the grinding meal to feed the pigs when they are called in the evening for the night. In our small way that is what we contribute, the women and the children role is to feed them.

Mod: Now for those pigs which just roam about how do you know that my pig is sick?

M2: It is difficult to know, if it is sick in the bush and there is no one who knows that this is your pig it would just die in the bush, unless some who knows you pig sees it and informs you then it is just a loss.

M1: The other contribution is that sometimes when they are called in the evening those feeding them would notice that it is not very active and not eating with vigour. They would therefore notice that this pig is not very well.

M5: The pig is a very active animal so when they are called in the evening you would notice that this pig is not active, the appearance of the eye. The front snout would appear white and it will always stand still with the snout on the ground.

Mod: The pigs you rear what do you feed them on?

M3: Like this time after the harvest we feed them with maize bran and we cut the pumpkin and feed them when they come in for the night. But if you have nothing you just give them remains from the pots and they sleep hungry.

Mod: Who in the family feed the pigs?

M2: In most cases we give the children to feed the pigs. In *Nsenga* land we have belief that if you give a particular child who has a good rearing hand, the animals would usually multiply. So such a child may be assisted by another are usually responsible for feeding them so that in the evening when the child calls the pigs recognise the voice and come running. So in most cases this is a role played by children.

Mod: For example how many times do you feed your pigs from morning to evening?

M2, M3: Twice a day in the morning when opening for them and in the evening when they are called.

M5: Depending on how well of you are in terms of maize stock. Yes there are some who feed the pigs twice like it has been said but in all honesty they are only fed once in a day in the evening when they are called. That is even the time we count them because like we said they just wonder about and anything can happen.

Mod: Do you get any help from other family members apart from you immediate family?

M1: Yes there are. In *Nsenga* we have a name we call it “*Kuvuula*”. This where a family member will agree with the relative who owns pigs to be bring maize bran with a promise that when the pigs give birth they will be given a female pig for them also to raise their own colony.

Mod: When you decide that you want to slaughter a pig where do you do it?

M6: Well there is no special place where we take pigs for slaughter we do it anywhere, we just go to the nearby bush cut some branches with leave and kill it and start to remove the hair.

*Laughter*

Some in the panel says even at home.

*More laughter*

M4: It is done just at home. Once you decide you catch it and tell the children to cut branches with leaves, cut it and start removing the hair, cut it up if it is for sale you start selling and keep some meat for the family. Mind you this is very tasty meat.

Mod: Who makes a decision in the home, like what to do with the pigs?

M6: This decision to rear anything at home is done in consultation with your wife. She must agree on what livestock would be kept in the home.

M3: This decision to sale a pig is arrived at by you and you wife and some of the older children, because if you are faced with a problem like school fees, children have no clothes and money to be used in the home you consult and arrive at a decision.

M5: In addition, yes we consult the whole family and agree as a family, but the final decision to sale or not is made by the man, because you might refuse to sale the pig and say I will do some piece work and meet those expenses. So you, the man, make a decision.

Mod: what do you see that is bad in the pig eating human excreta?

M5: It brings diseases.

Mod: Others what do you think is bad?

M6: Like it has already been said it’s because it brings a lot of disease, some of which are incurable, like fits which goes into madness. We human beings suffer from all kinds of diseases which the pigs contract from eating human waste and in pass it from them to us when we eat pork.

Mod: What caution or advice would we give to others in their life in the eating of pork?

M5: Like it has already been said when a pig eats human waste, some of the people doing that may be infected with some these very dangerous diarrhoea diseases like cholera, dysentery and many other. And if the pig came and ate from the plate which is used by us we would contract this disease and would result in an epidemic of very high magnitude which can wipe the whole village if not contained in time.

Mod: At which time of the year when pork is eaten frequently in a year

M2: Usually pork is eaten very much during this time and in December during Christmas celebration. Right now if you go in the village outside you will find pork on sale. During Christmas people appreciate more pork than goat meat because of its taste.

M1: Pork is also eaten in large amounts when there is a marriage ceremony, funeral and during other ceremonies.

M5: In addition also when there is swine fever like now, because you find that there are several dead ones and the meat becomes very cheap.

Mod: If a vendor is going round selling pork what do you pay particular attention to in the pork?

M3: We pay particular attention to the appearance of the meat. It should not have *nsembe* in the flesh. If it presents that appearance we reject the meat. Good meat must have good fat and the appearance must be attractive to you.

M1: Sometimes you can tell even from the blood which is coming from the meat. Blood from a diseased pig has a very different appearance from a health pig. Sometimes even the vendor would tell you that this pig died on its own.

M7: We want to see the care that was taken when the pig was slaughtered and that the hairs were removed properly

Mod: Any additions?

I (Dr. Mwape): If the pig is sick and the owner slaughters it before it die, how does the meat look like?

M4: Meat from such a pig usually has a very red appearance even the blood coming from it has usually a very red appearance. You are aware that you cannot remove all the blood completely from meat.

M1: And when it’s sick for a long time the meat usually has no fat. All those signs show that the pig was sick.

Mod: would you be happy if pork was inspected after slaughtering?

M4, M7: Yes, we would be happy if the meat was inspected because right now we are just eating the meat and we do not know whether it is sick or not, including chickens.

Mod: What type of preparing pork do you like best?

M2: I like best roasted meat because it tastes better since there is no water involved in its preparation. Especially us who drink beer when the food is placed on the table with roasted meat we really enjoy the food because the meat is very sweet.

M4: Yes and we even ask for the meat with fat from around the snake.

*Laughter*

M5: Boiled pork is also fine but not over boiled and the finish must be fried so that it does not stick in between your teeth.

Mod: What other cooking methods of meat that you like before the meat comes to the table?

M7: Some women do not initially add water to the meat when they start cooking, they will boil for some time and when the water dries they fry the meat and add a bit of water and fry again. They repeat this several times until when the meat is cooked. Then she would add a bit more water for the soup to finish. If she does not fry the meat the soup would be white and there we would say that woman does not know how to cook.

Mod: How do they make the soup thick?

M2: This is made when the woman is frying the meat. Now those small pieces which tease off as she is frying with a cooking stick are the ones which settle down and make the soup thick.

M3: Roasted pork is the best you cannot compare it to any form of preparing meat especially pork.

Mod: What time do you not like the taste of pork?

M2: The only time I do not like the way the meat is prepared is usually during the funeral and during some other events when there so many people who are involved in the cooking, and sometimes when you cook for a group it’s very difficult to take good care when cooking. And sometimes you have no control on how they prepare the meat.

Mod: Have ever seen *nsembe* (grains) in the flesh of a pig?

All: Yes.

Mod: What are these *nsembe* in a pig’s flesh; do you know what cause these?

M4: I am not very sure.

M2: I believe they come from the food they eat

Moderator passes a picture around showing the *nsembe* in a pigs flesh.

M3: I think they come from feeding pig with *masese* (solids) from the local beer. I remember my grandmother who was fond feeding pigs with this material they always developed this condition. Also if the one feeding them is not a good herd’s person the goats develop this condition.

Mod: What would you do if you found a pig meat which was infected by these *nsembe*, would you eat or sale the meat?

M3: We sale and eat the meat.

I (Dr. Mwape): Do you buy the meat?

M3: Yes they buy the meat. Because when they cook it *nsembe* gives it a feeling like they have added rice to meat.

*Laughter*

I (Dr. Mwape): Is it cheaper or it is the same price?

All: It is the same price.

*Laughter continues*

Mod: Anybody else would you throw away this meat with *nsembe* or not?

M7: You see we eat the meat, because you find that you have this whole big pig and when it is slaughtered you find these *nsembe*. You cannot throw it away, you just eat the meat.

I (Dr. Mwape): Would you be happy if the government asks you to throw away the meat from a vey huge pig which you have slaughtered?

M3: We would be happy, because at the time the government would explain the reason for the *nsembe* in the flesh of the pig. Now we are just eating the meat without knowing the cause of those grains and what they do to us.

Mod: Do you use pit latrines in the village?

M5: To say the truth there are very few people who have pit latrines in their home. Most people use the bush and that is how pigs survive eating human waste. You may find in the village there are only two latrines in the whole village. You therefore ask the children to go to the bush while you use the latrine at home.

Mod: What is the advantage of using the toilet?

M2: The advantage of using a toilet is that our pigs would not eat the human waste and therefore would not carry the diseases which they get from eating human waste. We would prevent the spread of disease if we use the toilet.

Mod: Any more thoughts?

M1: The toilets assist in implement good hygiene.

Mod: In a family where you have mature daughters, small children and your sons in law do you use the toilet the way you are supposed to?

M5: Let me tell you the truth. Usually in home there is only one toilet so what happens in a home where you have older daughter, small children and your son in law? It is either your son in law uses the toilet and you go to the bush or he goes to the bush and you use the toilet because we avoid to crash with your son in law, especially in the morning when you all want the toilet.

Mod: Any more thoughts, do we use the toilet in accordance with the way it is supposed to be used?

M3: I would be right to say we do not use the toilet the way we should because here we respect each other too much. Our culture and norms do not allow us to use the same toilet with your grown up daughters, son or daughter in law, mother in law and all other people. What have killed us in the villages are these cultural norms which we have clung to for so long. Our friends in town find nothing wrong with all this. They all use the same toilet in the house.

M4: So you find that you go to the bush.

I (Dr. Mwape): If you go to the bush are you not going to meet your daughter or son in law?

*Laughter*

M3: Yes you can because it so happens sometimes that where your mother in law has come to help herself that is where the son in law had gone to cut a pole. Then as he is moving he finds the mother in law squatting down helping herself, that it what can you do?

*Laughter*

M5: Some time it is because we feel shy with all your grown up daughters sitting around and they see you busy rushing to the toilet, that also make us not to use the toilet.

*Laughter*

Mod: So even us are not free to go to the toilet?

M5: What we are talking about happens.

M3: It is true like my brother has said you find your daughter can not use the same toilet as you or you cannot use the same toilet as you daughters. So you take an axe and go to the bush to help your self. It is just our tradition that you cannot use the same toilet as you’re grown up daughters. Yes in towns it is normal practice that the whole family uses one toilet but here in the village that is taboo. This still happens in the villages even today.

I (Dr. Mwape): So even you; you cannot use the same toilet as your daughters?

M5: What we are saying is true and still happens, where you see a man picks up his axe on the shoulder and goes to the bush pretending that he is going to fetch fire wood and yet he is going to help himself, especially now that the grass has been burnt. You come back carrying a piece of fire wood just to hood wink the children that you had gone to look for fire wood when your main purpose was to go to the toilet.

*Laughter*

I (Mr Mvula): Do you go in the night?

Several men: it is even better at night because under the cover of darkness nobody can see you.

*More laughter*

M2: If you delay in the toilet everybody would be counting the minutes and start thinking that has the old man passed away in the toilet.

*Laughter*

M3: May be they also want to use the toilet.

*Laughter*

M3: Sometimes you just pick a hoe like you are going to look for mice, so that when you come back without the mice people just think that you failed to find any. You know that our toilet do not have proper door like those in town so during the day if you go in the toilet children may find you squatting helping yourself. But in the night it is better no one can see you.

M5: Because sometimes when you enter the toilet your head will be seen so when you sit everybody sees that the old man has now sat down to help himself.

*Laughter*

R: Why do you not put doors and build a high toilet where your head can not be seen?

M3: Like you have explained, you would find that all of us seated here have toilets well-constructed with doors and a good roof. But you find that others villagers either have a poorly constructed toilet or have nothing. So how do you practice good hygiene? The poverty levels in the villages also make things worse.

M2: In the village it is very easy to dig a pit and find pole to put on the pit. Yes we think about constructing a toilet with a good door, a proper roof but you find that to have a good door made you need a carpenter to make you the door. It will cost 60,000 Kwacha for the door alone. A frame for the door 20,000 kwacha and you need to pay the carpenter so you find that this cost is too high for you to meet but your need a toilet. It is when things turn out that way that we start making short cuts to just put a sack instead of a door. All this is because of the poverty in the villages.

R: You confuse me when you talk of poverty. Is it not just digging a hole and cutting some fire wood and you have your toilet?

M5: Yes that is what M2 said. The simple part is for you to dig and cut some trees but you need a carpenter to make you a door, frame, nails and fix the door for you. That is the major problem. Otherwise we can get all the other materials from the bush very easily that is why you have heard us referring to poverty so often.

R: How about the door for your house where you sleep? Where does it come from?

M5: Yes, you would say that but the problem is that in the village you may go into the bush cut some trees and weave a simple door for the toilet. But you find that in the village there are maybe five or six toilet and everybody rushes there. You cannot stop them. The toilet becomes like common user and therefore the traffic to the toilet is very high.

M3: Yes like he has asked that where do you get the door for your house where you sleep, we make an effort to find the money to have the door made for your house, either you sale one of the animals to make sure you have a door made. In the case of the toilet you find that even your friends who come would discourage you to have a proper door made, because they would say why you want to waste a good door when the toilet will be used by an entire community? Why not just put a sack? That is why most toilets do not have good doors like our homes. But like it had been stated if your self feel that I want my toilet to look nice and have a good door made it, is up to you, but here are those who are still in the past who would discourage you.

Mod: Why do you talk about poverty?

M2: Yes like Mr. Mwanza has said you find that if you have a properly made door on your toilet because of so many people using the toilet and the door is frequently opened, you find that the hinges would damage in a very short time. Then you would need to replace them every so often. That is why we talk about poverty. But if you put a sack it will last long because it will just be flying about and is much cheaper to buy or find and you do not need anybody to fix it for you.

Mod: There are some people, who make a toilet and make a very good door. Then they fix a lock on it and they just want to use it with their family. Does that toilet perform its function well?

M2: It does not perform its function well.

Mod: What are the reasons?

M1: The reason for making a toilet is to reduce diseases. Your neighbour should also use the toilet so that the experience the beauty of having a toilet because they would hide in the toilet when helping themselves.

M5: The other factor that makes such an arrangement not very effective is that I as a father I would have keys then I hide them how about the children? What if one child has the key and other children want to use the toilet? And also there are those who are passing by and want to use a toilet. You want your family to feel free to use the toilet and by so doing you free everybody to use the toilet. I who is speaking I have a lock to my toilet but I do not lock it, because I have eight children. It is not possible for me to make a key for each child so in the end you leave it opens for all to use. That way the toilet is fully utilized for the purpose for which it was built.

M6: The problem of locking a toilet. Yes you can tell your children and their family members where they would find the key but how about your neighbours? Where do they go if they do not have a toilet? Then you create enmity with them, they would start talking that how does he locks the toilet where does he think we shall go? You therefore become an enemy. That is the problem of locking the toilet.

M5: I come back again. Yes you would lock the toilet and then your neighbour wants to help himself. Now since the toilet is locked he goes behind the house and helps himself and then the pig goes and eats the excreta and come to eat in my plates, and if we get infected by a disease in my family it means the problem I am trying to solve comes back to me. That in my opinion defeats the whole purpose of having a toilet. That is why we leave our toilet open for all to use hoping that the neighbour will learn from you and build their own toilet in time.

I (Dr. Mwape): You have stated that most of the people do not have a toilet. What if the chief passed a law that he wants every house hold to have a toilet in two weeks do you think the people would agree?

M5: Ah Now that is a possibility but what need to be done is to educate the people about the goodness of a toilet. Most people are forced because of the rain, but if a persistent education campaign was mounted and taught the people about the goodness of a toilet that would achieve desired results. You know it is very difficult to change the mindset of a person. Some would say we started a long time doing this even our great grandfathers went into the bush, what are you saying. In my own view the only solution is to teach those who have not yet seen the benefits of using a toilet.

M7: Yes, we can talk of educating the people who are not yet aware, but if you remember not long time ago we had people going around and teaching us to have a toilet, rubbish pit and build a place where to dry plate when they are washed. But some people did not follow that. Some people are just difficult and strong headed. They cannot be taught anything.

M1: In addition there was indeed a programme which was making stand plate for the toilet and giving them to people for free. Some have stored them in their houses and some are using them to stand on them when they are taking a bath. They do not use them for the purpose they were intended for. There is indeed need for sustained education in this area of our day today living.

Mod: Do you mean they have the stands? And do they go to the bush?

M1: Oh yes they run there faster.

M2: I think like some other speakers have said we need a sustained education campaign. You know that education never ends and for some people to learn anything it takes them a long time. Some people are very quick in learning bad habits and a bad thing is easily accepted by many people. If now I suggest that let us kill a person and get money, several will follow me but if you try to teach them about the advantage of building a good toilet they will brush you aside and say that you can please tell us something else. Are you not tired with this? We therefore need a sustained education campaign which will teach us how to rear pigs and show us that yes pigs brings money but they also can bring diseases and these are the measure you should take to mitigate this. We need sustained education awareness where people would be taught the connection between pigs, toilets and our health.

M3: Still on the same issue, you would find that you are a newly married couple. It is just you and your wife only, you have no children, and then you decide that instead of me going in the bush let me dig a toilet. Once you finish building the toilet you look around that your neighbour has a large family. Then you think that if I allow these people to use my toilet it will soon be full so you look for lock and start locking it like in town. But after sometime you realise that these pigs we keep will eat their human waste since they do not have a toilet and affect me as well. You stop locking your toilet.

I (Mr Mvula): Yes we can teach but if you notice there are less than 50% in the villages who have a toilet so how many times are we going to be teaching the people?

Mod: Okay, Do you wash your hands before you eat *nshima*?

M2: Yes we wash our hands.

Mod: May be should put it this way. At what time do you wash your hands and what are the benefits of washing your hands?

M6: You need to wash your hands when you come from wherever because you may not be sure of the things you have been handling they could contain some disease and also when you come from the toilet, so before you touch even a cup used for drinking water you must wash your hands.

Mod: Do you think children wash their hands when they come from the toilet, do they take care of that part?

M6: Children should be taught to wash their hands when they are about to eat any food.

M1: In some families they teach their children to wash their hands when they come from the toilet even if there is no food to eat. This teaches them that the place they visit is dangerous.

Mod: Now some of you have said unless you are there that is when you tell the child to wash its hands, what happens when you are not there?

M3: That child has learnt, even if you are not there that when he goes to the toilet he must wash his hands and before eating food he must wash his hands, because he was taught properly and understood.

Mod: Like in our village setting, when a person goes to the field and carries water in a plastic container, then after sometimes he goes to the toilet and when he wants to drink water does he wash his hands?

M8: No it is not possible because we carry very little water so what happens is you just continue with your job.

M5: In addition to speak the truth, in our village setting the only time that we wash our hands is when we are about to eat *nshima*. There are very few people who wash their hands when they come from the toilet, may be but in all honest they are very few who put that into practice

M6 agrees.

Mod: What is the benefit of personal hygiene?

M5: In short it helps you to have a long life span.

M8: It helps to prevent diseases.

Mod: Have you ever seen worms in human excreta?

All: Yes.

Mod: How do the worms come and where do they come from?

*Silence*

M3: We do not know how they infect us. Even when you are sick sometimes you do not know what is causing that, until after you are examined at the clinic. That is when you are told that you have worms.

M7: I have heard from some people that if you eat too much rape you would be infected with worms, but I do not believe that theory because I do not understand how rape can infect you with worms.

Moderator is circulating some pictures

Mod: Have you seen those pictures? What you see there is a child’s excreta, and then within it you see some white thing which looks like a wood borer and like the after shade skin of a snake. Have you ever seen that in a child’s excreta?

All the men agree having seen them but not with a lot of confidence. The men are discussing in whispers.

Mod: Have you ever seen that after a child goes to the toilet?

M7: Well like round worms we see them.

Mod: Do you think you worm infection can be prevented?

M4: Yes, you can prevent worm infection.

Mod: In which way?

M4: In 1975 my niece was infected with worms we took her to the clinic where she was given treatment. So after that we changed her eating habits, because what used to happen before she would drink tea and then eat left over *nshima*. After that experience we stopped her from eating left over *nshima* which was kept overnight, after that she never had the same problem.

Mod: How do you know that this person has worms when you see them, M1?

M1: Tears in the eyes

Mod: Tears in the eyes?

M1: Yes, especially in the young ones

M3: As for me when I was in town I used to feel cold in the morning and feeling itchy on my skin and I would scratch myself and my stomach would feel full. I feel the same in the evening when it gets cold. Then in 1992 I came here and I came to this same clinic. I found Dr. Daka. I explained to him how I was feeling. He said I suspect you have worms and he gave me two tablets to drink and told me that I will not go to the toilet for the next two days. True to his word I did not go to the toilet for the next two days but on the third day when I went to the toilet I passed out a huge ball of worms. When I came to the clinic to report to him what had happened he told me to put them on a sheet of paper and bring them here, then he said yes this is what we wanted.

Mod: Any more additions?

M1: Yes a person with worms usually eats too much.

Mod: Yes anymore?

M5: Again a person with worms usually appears light in weight like. He would be blown away by the wind and also his hair is thin like a person who had been sick for a long time and with a large appetite like it has already been said.

M6: And the stomach has an appearance of being hard and bugging out.

Mod: What threat do these worms pose to the person’s life that is infected with worms?

M5: These worms share the food with this person so his health is under threat. The person does not gain weight so he has no energy to do any manual work therefore affecting his personal development and that of his family and he may even die.

Mod: Now is there any other disease that we have in the village that are bigger and more frightening than worm infection?

M2: Yes we have, this new disease called AIDS that is more frightening.

Mod: How about those which do not frighten you?

M1: A cold.

Mod: Do you know fits, what is it?

M1: It is a sickness.

M5: It is a disease of seizures.

Mod: It is a disease of seizures, for example do you have people in your village who suffer from fits?

All: Yes they are there.

Mod: How do fits come to a person? How many ways can you explain?

M2: We have been told that people who suffer from fits are usually bewitched or if they eat warthog meat. What we know is that it is caused by any of the two.

Mod: I want us to discuss the way in which fits infect a person and how you know that this person is suffering from fits?

M4: In some cases it comes when children have a very high body temperature and if it is not controlled within a week you see the child starts to fit.

Mod: Anyone who can continue, Mr M6?

M6: Yes like someone has explained it starts when you have very high body temperature and you think you have malaria. After a few days if not controlled you start fitting, then you start to wonder may be it is that pork you ate.

*Laughter*

Mod: What problems would you find in a family if there was a member of the family who was suffering from fits?

M6: The problems that you would face if a member of your family was suffering from fits are that you would all look like you are suffering from fits. Then in most cases these people become mad and if you do not quickly treat the madness the person dies.

Mod: Any additions on the badness of fits?

M4: In most cases a person suffering from fits cannot warm themselves near a fire even if it is cold because they are scared that they may be attacked and fall on the fire. Also even when they are happy they cannot afford to be over excited because they would experience a seizure from nowhere and also if there is a celebration in the family if they dress up in a suit you would find that they have an attack in their best clothes. So usually they are not happy people.

Mod: Anymore?

M2: The other thing those who are married when you go in the bedroom at night you start feeling each other so that you prepare to have sex and when he penetrates his and they start making love if it is the husband suffering from fits, you find that at the time when things are nice and he is just about to ejaculate that is when he has an attack and the seizure happens. That is a very big disadvantage of suffering from fits.

*Laughter*

R: Have you heard something like that in your village?

All: Yes.

*And more laughter*

M2: Not in our village but in other village. Yes even marriages finish because the wife thinks he may die. Because she thinks it is the sweetness that has caused that when in fact the person have just the disease.

*More laughter*

I (Dr. Mwape): Do marriages really finish?

M2: Yes, because your wife thinks he is going to die. So you lose a wife because of the disease that is a very bad thing about this disease.

*Laughter*

M1: The problem with this disease is it also brings poverty, because you are always moving from one medicine to the other looking from medicine. You sale chicken so that you find money to pay these medicine men and then poverty enters in your home.

I (Dr. Mwape): How about these young women with fits do they get married?

All: Yes they do.

M1: Yes, they get married they say “love is blind”.

M2: Yes they get married but the problem comes in the future if the fits continue unabated, then the marriage would finish.

I (Mr Mvula): She may bite you on the chest.

*More laughter*

M2: Sometimes it happens so often, that after feeling each other and when you are ready to do make love then that is when you have seizures. After so many disappointments you give up and say let me go and try somewhere else then the marriage breaks up.

M5: In the same vein, if for example you went out with your wife to a celebration and then you had seizures in a public place, she calls you and you are not responding. If you had sex with her two days ago then you can be sure you never have her again because that would be the end of the marriage.

I (Dr. Mwape): Just when AIDS started when people heard that you were HIV positive they would not greet with their hands and would avoid you. Do you do the same with people who suffer from fits?

Several people in the audience say no, these people are accepted in the community with no reservations

M2: No, the problem only comes in marriages.

R: Do these things happen in your village?

M2: Not in our village, but these things happen and we hear. They are told about them and we have witnessed marriages which have finished.

R: Do they tell you the reasons why the marriage finished?

M5: In anyway because they would say because of problems, when in fact we all know what the actual problem is because the partner is suffering from fits.

*Laughter*

Mod: Do you know if that fits can be prevented and is medicine available?

M1: May be those specialists but it is difficult.

M5: Ah the prevention we do not know because first of all we do not know how the disease is transmitted. May be my friends would shade some light.

M2: Prevention we are not sure but treatment yes there are experts who know medicine. Yes they heal the diseases, but prevention no because we do not know how the disease is transmitted so we cannot talk about prevention unless we knew the mode of transmission.

M3: Yes if a disease is a normal one, people get healed but if you have been bewitched by a person because they do not like you then forget about being healed.

M6: Even in the hospitals medicine is available.

Mod: Like we have pointed out that even marriages can break up because your partner is suffering from fits, is there a disease which is severe than fits?

*Silence*

Mod: A disease which would be greater (severe) than fits? Which you would say fits are much better than this?

M3: AIDS.

M1: I agree with my friend AIDS is greater than fits, because a person suffering from fits is usually fat, but a person suffering from AIDS despite them eating well they become very thin.

Mod: Do you think these two ailments are different or are not any different?

M1: There is a difference. AIDS has no cure while fits can be completely cured.

M7: If you have a family that is caring with fits you can manage because about fits the only fear is not to fall in water and on fire, but with AIDS the problem is even worse because even if you ate well there is a problem. If you starve it is even worse you would not last long.

*Laughter*

M5: After careful consideration in my own opinion of the question which one is a greater disease I find fits a much worse off disease because if it is a bad one, the person cannot do any work but a person who is HIV positive can get medicine from the hospital and once he gets well he can do anything.

M6: A person suffering from fits if he falls on a fire he will never be cured even with the best medicine man alive.

M3: The problem with fits is that it needs a lot of attention from family members. You cannot leave him alone because he would fall on a fire, in water and that would be it. So this disease is much greater than AIDS.

Mod: But a person with AIDS is free to move about on his own.

M3: Yes, sometimes you even admire her if it’s a woman.

M1: I think here why I thought that AIDS was much greater it is the enormous effort and cash out lay that the ministry of health has put up to fight AIDS, care giver, teachers, health clubs and NGO involved in fighting AIDS, compare that to fits. That is why I thought “fit “was a much better disease because all it requires are family members to care for the patient.

M6: The problem with fits is that the only care givers are family members. You cannot leave him alone near fire, water while an AIDS patient once he starts receiving medication he can completely be left alone. That is the beauty of aids.

Mod: Now which disease in better than fits?

M1: In my own opinion there is no small disease, because you find that a person suffers from tooth ache die. A boil on the finger can kill you therefore in my opinion any disease that can make you come to the clinic is not small.

M3: A boil in the head can kill you.

*Laughter*

Mod: Alright, if a law was passed that we should keep all our pigs in the fence would there be a problem?

M3: There would not be any problem.

M6: That is when it would be very good.

Mod: Alright, if another law was passed that every house hold should have a toilet, would there be any problems?

M7: There would be no problems because that is what we were being taught most recently.

Mod: What if you were told that you need to boil relish for a longer time what problems would you encounter?

*Silence*

I (Mr Mvula): If you were told that you must stop roasting but boil the meat for a long time on the fire what problems would that cause?

All: No problems.

M7: As long as we are served with well-prepared meat on the table there would be no problems.

M2: It would be fine because it prevents other diseases and those *nsembe* found in the pig flesh would also cook.

M6: They do not cook.

M1: It would be better for each house hold to have an earthenware pot because it gets much hotter than an aluminium pot. Even when it is taken off the fire it will still be boiling for some good minutes, not a pot.

*Laughter*

Mod: So, on this rule it should specify the use of an earthenware pot not an ordinary pot?

M1: Yes

Mod: An earthenware pot for cooking pork only?

M1: Yes, just for cooking pork.

M5: Like it has been said it would be better to use an earthenware pot because we all have experience that it cooks thoroughly but you do not have an earthenware pot. It would be better to boil pork thoroughly even when you use an ordinary pot. The only disadvantage is that when pork is over boiled it sticks the lips together when you are eating.

Mod: If a law was passed that all pigs should be inspected before they slaughtered what problems would that bring?

All: There would be no problems

Mod: How about hygiene, cleaning the meat before cooking would that be a problem?

All: There would be no problem,

Mod: Treatment of diarrhoea diseases, if the campaign for immunisation came what would be the problem?

All: No problem

Mod: If a law was passed that all pigs should be vaccinated against swine fever in all the villages what problems would that cause?

M3, M4: There would be no problem.

Mod: What if they say before we vaccinate make a small contribution towards the cost of the medicine, would you agree with that proposal?

M3: That would be a very good idea.

M6: The one who would refuse to have his pig vaccinate should have his pig killed.

M1: As long as this contribution is reasonable.

M5: Let us listen to each other, no all us talking at the same time. You are all aware that most recently there was a vaccination campaign by the department of veterinary services to vaccinate cattle and they were charging 15,000 kwacha per animal. You all know how much money cattle make in the village but most cattle owners were failing to pay. Now a pig does not bring as much income because it takes a few months for a pig to mature and be ready for the market. You can be sure that if you were charged 15,000 kwacha per pig you would manage considering that you may have only four pigs. In my opinion we should ask the government to assist us the first time and once all people see the advantage of vaccinating pigs then they would ask us to make the small contribution you are talking about. Let us not agree to something then afterwards fail to pay for the service and let us not just agree because it is easier to say yes and please our visitors.

M6: The problem with all of us who keep animals is that we don’t want to take care of all our animals. It is not only pigs which eat human excreta, chickens do and they also carry diseases. All these animals should be kept in the fence so that they do not contract diseases and in turn infect us. The only thing maybe we can do is to ask where they sale the medicine for vaccinating pigs so that we can do the vaccination ourselves may be that would help.

M1: You see these people here they are in a group and are writing whatever we are saying, and us we are about seven meaning that we are representing all those who are not here. It is therefore important that we say things which will help all those who are not here even the old grandmother. Let us be objective and serious with our deliberation, least we are accused of having agreed to things which may not be helpful and reflect the opinion of our community. Let us not just agree and say yes sir, yes sir.

*Laughter*

M5: You all know that some of us can manage but when we talk we are speaking also for the vulnerable, those without means of support. If just say yes sir, yes sir in future we shall be blamed for agreeing to conditions which cannot work and are not sustainable. So when we talk let us bare in mind those who cannot afford and are not here, not about ourselves. That is why some of us talk like this.

M1: Mr Mwanza (Mod) a short time ago you asked us about toilets. How many toilets are in Chiluzu village? Very few when for a toilet you just dig a pit and cut a few poles from the bush and all these are free and the stands were distributed free. What more if you ask people to go and buy medicine? Let us be serious and agree to terms which can be met without much difficulty.

M5: We had several workshops teaching people how dig toilet and we spent three days with Mr. Mwanza *(Mod)* sensitising people at Chiluzu Village but how many toilets are there? As my predecessor has said all the materials were free. What more if you ask people to buy? Nothing will be done.

Mod: What problem would be there because the government has come with this help, and all they are asking is for you to make a small contribution, what is the problem?

M1: The problem is the small contribution. At first even with cattle the government was offering free service and teaching the people on good animal husbandly, but immediately they started asking for the same contributions and people started failing to pay. You notice now there are more deaths in cattle.

M2: The moderator asked a very straight forward question that if the government brought a law to vaccinate all pigs would that be okay. We all said yes. He again asked what if the government asked you to make a contribution towards the medicine expense we said it would be okay. But what we did not find out is how much is that contribution is supposed to make or what percentage are is that contribution supposed to be? Is it 25% or 75%? We all know that cattle owners make money every day. In the case of a pig you have to wait for months and because it eats too much, all you do is feed it. Also a pig gives birth to a lot of piglets, so if the small contribution is not so small people would fail to pay.

*Laughter*

M2: I really think to know the percentage it’s important, because we would be happy that they have brought us medicine for swine fever but when the small percentage we are talking about is 50,000 kwacha then what happens?

Mod: Alright I know that since we started our discussion we talked on a lot of things, we discussed about the rearing of pigs, the advantage and disadvantages of free range rearing of pigs, we discussed about toilet, we also discussed about worm diseases, we discussed about fits, we went on to discuss about which disease is more frightening, we discussed about human immunisation against worm infection and vaccination of pigs. It time that anybody with any question can ask, Mr M6?

M6: I do not have any question.

Mod: Mr M8 anything to say?

M8: I have nothing to say.

M7: My contribution would be that, we have had a lot of workshop as you have heard Mr Moderator. We were taught on how dig toilets, dip pit for our rubbish… But you can see we do not seem to implement them. In my own opinion that is not encouraging to those people who come to teach us. We should try to implement what we learn and put them in practice, not when we leave this place and go into *Kakwiya village* we forget everything and is business as usual.

Mod: Any one else?

M1: These lessons we have learnt, are they meant for our pigs which we have in the village or is there another programme for pig restocking that is coming?

R: It is for meant for you to improve your pig rearing in your village. Do you want to eat your pigs and wait for the government to give you pigs?

*Laughter*

M1: No it was just a question.
