# Supplementary material for: Why Latrines Are Not Used: Communities’ Perceptions and Practices Regarding Latrines in a Taenia solium Endemic Rural Area in Eastern Zambia
Source: PLoS Negl Trop Dis. 2015 Mar 4;9(3):e0003570. doi: 10.1371/journal.pntd.0003570 (PMC4352092; doi:10.1371/journal.pntd.0003570)
Supplement: S1 Dataset — (ZIP) [file pntd.0003570.s001.zip › FGD transcriptions_Zambia-2010/Mtuna_ Dr Phiri/Women_Mtuna_05-08-10.pdf]

Title : **Focus Group WOMEN**

Date : 05/08/10

Site : Kakiwa Rural Health Center

Village : MTUNA **Location :** Petauke district, Eastern Province, Zambia

Duration (total time) : 85 min **- Start :** 12:51 **- End :** 14:14

Participants: 10 women

Informed consent: signed and available

Moderator : Ms Ruth Chiwa **Recorder :** Dr Andrew Phiri

Transcription/translation : Dr Andrew Phiri

Abreviations : M : moderator, R : recorder, I: intervener, C : children, W : women, M : men

Disposition (from the left of the Moderator):

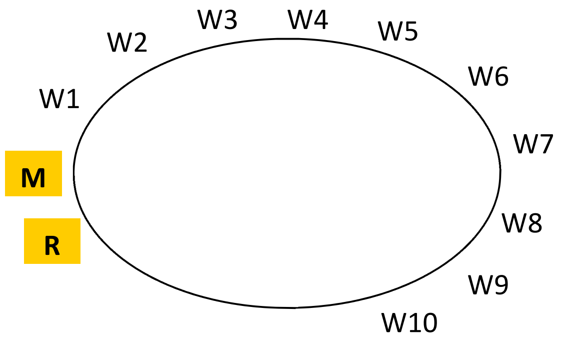

<u>Age composition</u>:
W1-W2-W3-W9: oldest
W5-W6: youngest
All:the rest: middle age
<u>Typology</u>:
W1-W10: dominant
W4-W8: shy/quiet
W5: rambler (expert and funny)

Comments about participants :

Three are active pig keepers.

Group dynamics :

Text:

Introduction by moderator:

M: How are you ladies?

All: We are well and you?

M: We welcome you here at Kakwiya, ok?

All: Yes

M: Before we start our program, we need to know each other, not so?

All: Yes, it is so

Introductions are made from starting from W10

M: I warmly welcome you all because you have come here. I believe you now know who is who, not so? I beg you that you should be people who are free, we need to discuss what we do especially those things we do at our homes.

There is no answer that is wrong or right. You are all adults; you should speak about things you know. When you do that you would not be saying untrue things. Therefore we need to be free.

Pause

M: We start now?

All: Hhhmmmm

Discussion:

M: Do we know pigs?

All: Yes

M: Do we keep them?

A few murmur in the affirmative

M: How many of you keep pigs?

W3: (*raises hand)*

M: Does it mean there is only one who keeps pigs?

W10, W9: *also raise their hands*

Murmur

W1: We have kept pigs before

M: What about how many eat pork?

Silence

M: How many of you eat pork?

Silence

M: Does it mean no one eats pork?

Most: *(rambling)* we eat

M: But, why don't we respond, what is making it shameful?

W3: We just need to raise our hands and say that we eat pork

M: Don't we all eat meat here?

All: We do *(in low voices)*

M: Does pork bring shame?

A few say: No

Laughter

I (Dr. Mwape): Are you ashamed of your own relish?

Brief silence

M: We all need to speak so that we know that there are many participants, not hearing from one person.

We need to speak up; I speak a lot. Don't fear to speak up. No one will blame or castigate you that *Nsengas* speak a lot. Not so?

A few women say in the affirmative

M: I am *Ngoni* and I speak a lot.., but it is you who have taught me to be so.

Laughter

Small talk follows

M: Why do you keep pigs in your village?

W10: We keep pigs so that when they breed, we get help with school fees after selling them buying school uniforms

W8: Pigs are good, when you sell the money realised is used at home; they offer us a source of income

W5: Pigs are good because they help cover funerals costs

W3: Transport costs to attend funerals in town are met after selling pigs

W2: Pigs help in both agriculture and at funerals

W9: In times of illness, when one is unwell, income to help with costs can be gotten from pig sales

W4: Helps us with illness, generally assists after selling pigs

W1: Pigs are good in that when we are faced with various problems at the household level, money from pig sales can be used to purchase maize,… It helps in times of hunger

M: What about the dangers of pigs, how do you look at it?

W10: The badness of pigs is that, in case there is no latrine at home, it goes to eat human feces in the bush, and returns to mess with cooking and eating utensils…That's the badness of pigs

W5: We can say pigs are bad too because it brings us diseases....from what it consumes

W1: A pig is bad in that there are some of us who don't keep our utensils in a proper manner. Those same utensils when a pig eats from them it leaves diseases in there. Others don't clean the utensils properly and therefore leaving diseases there…In that many of us do not have latrines, we go in the bush.

M: Others? The dangers of pigs?

W2: The badness of pigs is that there is no caring at the household, a free roaming pig can easily have access to the utensils...That's the danger

Silence

M: Others? The dangers of pigs?

W7: When an infant defecates, it consumes the excreta and can enter the kitchen if left open to drink water

M: Who can add on?

W3: What I can add is that: after consumption of pork one can get disease because of what the pig eats, those things that are not prevented

M: What type of disease can be found?

W9: Ringworm, others have deformed skin as a result of ringworm

W5: Number ones are worms and *nsembe*

W6: Others have sore throat

W10: Others even get epilepsy

M: What are other dangers?

Silence

W8: Indeed we keep pigs but the problem is that when disease outbreaks come, they all die

W1: They brings diseases at home, because others like ourselves don't take care...diseases just have to be there

Silence

M: Do pigs have any use?

W10: Yes, pigs have work ...Looking for maize bran and feeding them in the morning and the evening. If one does not have enough food to feed them, that's when they go in the bush in search of feces and get back to eat in utensils

W1: It has a lot of work. If you haven't constructed a kraal, it means *milandu* will continually be brought at your house. In the rainy season, it can eat from other people's agricultural fields. You need to construct a kraal and have enough feed. If you don't give it enough food, that is why it goes to the bush.

Milandu = crimes

If there are those that have constructed kraals and are keen to confine, things are much better. If you have not constructed a kraal, pigs lie and stay outside and therefore they go and eat indiscriminately and bring diseases in a home

W5: We can say a pig has usefulness because it helps when we have encountered problems. There may be a death, it helps us....may it be hunger alleviation, it helps

W6: It helps in various ways. We can say that it has both use and no use....Because it helps with many things

M: What type of dangers do we think can come from pigs?

W3: One has to build a kraal and a place where the pigs need to hang around so that they do not go outside and bring diseases. That pig would be a good animal because it won't contract diseases

M: The dangers that can arise from pigs, what are they?

W1: The dangers that can come from pigs is a disease of epilepsy because pig eats various things, they can transmit such diseases to humans

M: What about you W5?

W5: Pigs give worms and *nsembe*. All that; Even death; Worm infestation kills

M: What about you sister? *(Addressing W8)*

W8: If pigs are not properly taken care of, they can be a source of disease. When they eat feces and come back in the yard to knock around in the cooking utensils because of poor hygiene, they can transmit disease they would have gotten from the bush. Like that diseases never finish because of not taking care of pigs

M: Others?

W10: Pigs do not mind...If you have a baby and a pig doesn't have a kraal and it's is starved, it can come speedily and catch a baby...

Laughter

W10: …Bite it or even kill it. From there, you eat such a pig, diseases cannot be eradicated

I (Dr. Mwape): Why is it that people do not construct kraals in your village?

W10: The men… If they have not constructed them, that is the problem. A woman can find feed for the pigs, but if men haven't paid attention look for wooden poles for construction a pig's kraal, that is the problem that arises.

I (Dr. Mwape): Why are these men do not construct the kraal?

On why men don't build kraals, discussion was very animated. Women were submissions at once

W5: Not being cooperative...

W6: If we say please construct a kraal...

W5: ...Ignoring

W8: Laziness

Silence

M: Yes, let us continue…

Laughter

M: What other dangers can come from pigs?

Silence followed by nervous laughter

W10: Other dangers are that those pigs die anytime from disease outbreaks. When such a pig has died from suspected disease outbreak and you eat it that disease that has killed it would be transmitted to you humans. You yourself start getting ill and unfortunately by bad luck, you can die… All those come from pigs.

Disease outbreak = Presumably African Swine Fever

M: Can anyone add on?

W5: We don’t necessarily know the disease that pigs suffer from. We just eat pork and risk contracting the disease

M: What about meat…?

Giggles followed by laughter when both Moderator and W1 want to speak at once

W1: To add on…Truly, when there is a pig disease outbreak, all kinds of diseases come out that even humans contract

W2: One can even get a cough from eating an animal that dies on its own

M: Are there any other livestock that you women keep by yourselves?

W1: Other livestock are goats,

W5: Chickens, goats…

W9: Goats,… We keep all kinds of livestock

M: Even cattle?

W9: Hmmm

M: By yourself?

W9: In association with spouse of course. All those at home are involved

Laughter

A few women contribute simultaneously

W7: We do all that together

M: Why do you keep pigs?

Many women contribute simultaneously

W5: Farming

Simultaneous contributions continue

W5: Many reasons why we keep them

W6: Keeping them is good, if you don’t have livestock you suffer

W1: If you don’t have money for milling maize, you sell some chickens. If you don’t have soap, you sell chickens. If ducks are many you can sell some. With that we can find money for milling and for soap

W5: Cattle for ploughing.....You cannot suffer; there is goodness in having livestock

W4: If you have an urgent problem or need, you can sell a goat and have money to travel

W7: If a child at school needs some financial help, I can sell a goat and send the child money

M: What role do you play in keeping pigs?

W1: We have a role in looking for food. If you don’t look for food, it means pigs not grow well

W6: They starve

M: Where do you look for food?

W6: From what we humans eat

W3: Maize bran... For us who do not pound maize, we buy such from millers at the boma

W1: When you go to have your maize milled, the maize bran as a by product is collected

W6: .and given to pigs

W8: Sometimes one gets pumpkins..You chop them up into pieces and feed the pigs. In that way, one finds feed for pigs

M: Who within the family helps you in keeping pigs?

W1, W5, W6, W9, W7: No one helps, it is just you alone

W7: It is you alone who has keeps them. Organise maize bran and feed them

W6: In the rainy season, you give them boiled pumpkin leaves

M: So, at your home it is only you who does the duties of keeping pigs?

W5, W6: Yes, it is only you

W5: When you rare them, it is your duty

On discussion on roles, the contributions were animated; everyone wanted to talk

W10: Just oneself and children. No family member helps.

W5: No one helps

W10: …It is you to keep, even when it comes to selling, you select one for sale yourself

W6: When a family member has a problem, it through your own compassion that you render support

M: What about the men?

Uncoordinated submissions by a lot of women at once

W7: We do things with the men

W5: To speak truthfully, three-quarters of the men don't pay attention to pigs and goats. Women should look after the pigs, meanwhile men are around

W1: Sometimes, you have to sell a pig for someone to build a kraal for you

W5: Meanwhile a husband is around

W1: Sometimes you slaughter a pig for meat, others will give me maize. I have to look at the same pig for all these

Pause

W1: When you have agreed with a husband to build a kraal, he may agree. Many times the men refuse

M: Does it mean pigs are not for men?

W5: Yes, because the men don't pay any attention

W6: They are for men by virtue of pigs belonging to a household

W5: Many times they belong to a woman

W7: Sometimes it takes two years for him to build a kraal

W5, W6 and W7 continue exchanging views about subject at the same time as others laugh

W5: You do everything, meanwhile a husband is around

M: My sis, say something? *(Pointing to W8)*

Laughter

W8: I kept pigs before, but I used to do all things by myself, I used to confine the pigs alone. They say it is a duty for women

Other women smile and laugh softly

W5: A women has to do the shepherding while a husband is lazing around...Even putting pigs in a kraal

W6: Even if it means chasing pigs...it is a woman, meanwhile him is sitted. He is only concerned when the pig has reached the door or yard

Small talk ensues among four women and is accompanied with laughter

W1: If you want to be equal with men, then there are problems in the home, they would say men and women's intellect is the same

W5, W6, W7: They would say there is belittling

W5: People would say a husband is there; meanwhile he isn't. He is like the back of the book, at the back cover

Figurative speech meaning even if a husband is present at home; he does not perform his duties.

Long Laughter

M: Apart from your family members, who else helps you with pig keeping?

All women: There's no one else

W5: If a husband fails to help, what more an outsider?

W10: He or she would say they also have their own house. My husband and children

W5: For sure

W2: They would ask why we purchased the pigs for.

W5: Why did they buy the pigs? They would say her husband failed to make the pigs grow, what about me?

W1: They would say "let them do it as they do when they have their pigs sold...

Other women: Ehee!!!

W1: ...the same way they should do when they eat pork. Let them slaughter their own pig"..

Laughter and mixed exchanges

W5: They would say the pig owners hardly give me even a small morsel of meat…I don't even know the smell of meat

W6: They eat well themselves leaving us leftovers

Continued laughter

I (Dr. Mwape): What about children?

W10: Children? ...

W5: They also take part

W10: Children are part of a home and so take part. Sometimes you as a mother is tired, you can request the children to chase the goats and put them in the kraal, or if there are pigs you would say "Here is maize bran, go and feed them". The child would go and feed them

W5: The child would feed them

W7: When you are not at home, on your return you may find kids have helped with the chores

W10: However, not the men

Laughter

W1: When grown children have gone to school, it means it is you alone...Even if you have come from a funeral you will still find the pigs outside

W5, W7: Meanwhile the men are around

Laughter

W1: When at a funeral, you just tell your friends that “Oo I have helped in the burial let me dash home to take care of the pigs”…

W10: I will find the pigs still outside

W5, W7: The husband would be at home, ignoring all that

W5: However, when you sell some pigs the hubby would ask for money for a pint of beer.

A quick chattering continues on the subject among the women accompanied by laughter

W6: The men are chiefs

R: Meanwhile you would give money to protect…

Most women answer at the same time

W5: To avoid noise and arguments

R: Protecting....?

He learns forward to hear response

W5: Protecting arguments

W7: So that noise dies down

R: Not protecting marriage?

W5: Of course

Other women nod their heads in agreement

R: Protecting arguments or marriage?

W5: Marriage

Others agree verbally

W3: Speaking truthfully, it is about protecting a marriage for fear it may end

W5: ...Protecting a marriage for fear it may end… Because of that money from pigs

Laughter

W1: They would say “my wife doesn’t respect me”.

Laughter still

W7: That she doesn't pay attention to me

Laughter

W7: Instead of me wandering after marriage dissolution...

W6: …I would just give some money

M: What about who decides whether to slaughter a pig or sell it?

W5: It is a woman

W7: It is I who feeds and usually I would say "let us slaughter this pig so that we buy such and such items"

W5: …And to sell it is because of a certain problem. A man only needs to be informed,

W6: Just to be informed

W5: ... just for him to agree, he has no part...he is at the back cover of the book. So you the one who is in the middle of the book or leading should lead him

Laughter

W8: For when I kept some pigs, we used to sit down e.g. when school children had needs, we (my husband and I) would say "let's sell this pig"; the two of us would agree even on the price

M: Lady? *(Addressing W1)*

W1: Yes, when selling, I would liaise with my husband. I would say" the father of Charles, what are we going to do? Look at the problem that has arisen, there is no soap here. Where are we going to get money from; Let us sell one of the chickens". He would respond "why do you wish to keep on selling the chickens, don't you know that the numbers keep on dwindling and there may be a problem that can arise later?. I would say "What about this, isn't it a problem as well? He would reply "Ok, you can sell it". Like that you would have agreed on what to do.

M: What about children?

W1: You call the children and inform them as well

W5: You also tell children

W1: Children also help in keeping the pigs. You call and invite them in the evening before anything takes place. Before then the two of you plan, both wife and husband in a home. When we have planned, that's when you invite the children, the bigger children; the younger ones only sit and listen in when you plan with them

I (Benjamin): What if it comes from children?

W1: If a problem comes from children?

I (Benjamin): When a resolution or decision has come from children

W1: When it comes from children?

I (Benjamin): Yes

W1: We help each other there; if its husband who refuses, I would say “hey you, don’t refuse what a child has proposed”. Let us follow, this is a problem. In fact, this child is in our home. It is us to help this child; everything will need to come from us. If we refuse where else can he/she go? Like that we would have agreed

R: Children were earlier complaining that when they keep livestock, you parents do anything including selling the livestock without their knowledge. Even when you have given such livestock as belonging to a child, when it reaches market value or weight, you slaughter or sell without informing the child

Many women disagree at once, noticeably W1, W4, W5, W6, W7, W8, and W10

W5: That’s a lie

W4: A child is better informed

W10: Livestock at home are sold in the name of children because of schools.

All: Hmmm

W10: A child will come from school saying that “my teacher has chased me; they need a certain amount of money”. Now as parents when you ponder about it, you don’t have any money; that’s when you sit down with your husband and decide on getting one livestock , may it be a goat or a pig itself, or chicken so that we sell in accordance with the money that is needed at school. So this money will be used to take the child back to school.

So even if you have told the child about the complaints he/she has brought, you just sell a livestock and give the child saying “here is the money that you requested; take to the teacher”. The child takes that money to the school. In that way, this decision would have passed through the child

W1: And also when a girl child has reached puberty we are told in that book that you don’t tell a child that now you have come of age and therefore go and look for your own soap. It means you would have sent her to...

W5: It is sending…

W1: ...prostitution. But when she complains, you know a child can be free with you as a woman. She would say “mum I don’t have lotion, soap, my lotion is finished”. I would ask “they finished”, “yes” she would say. Now in the evening when going to bed, I would tell the father that listen to what the girl was complaining. She doesn’t have soap and lotion, even a book she doesn’t have. The child was proposing that we sell the billy goat. Then we you both agree and in the morning after she has returned from school, you tell her “that issue you requested yesterday, listen, your father said this and that. “Ooh” *(girl)* “yeah” *(mother)*. So now you go about, look for a customer who would want to buy the goat

The billy goat = the male goat

M: Who feeds the pigs?

W10: Us women

Pause

W10: Even children who are of grown up in age...Husbands can’t do anything

Laughter

W10: ...Help in feeding pigs.

W2: They say that it is a duty for a woman.

W10: Even you are crawling-crawling (signifying not feeling well), you rise to go and feed the pigs

W5: Even when you have left the home and gone somewhere, on your return you will find pigs with hunger, even goats you will find them like that....outside and not fed, like that. So especially in the rainy season, that's why you find them going to eat from agricultural fields and bring disputes that "Ooh!! Your pig has eaten in my crops in the field" because you do not mind feeding them. You a woman alone, what happens with women is a lot, the problems are numerous.

M: What kinds of food do you feed them on?

W1: Maize bran.

W8: Pumpkins.

W7: Pluck pumpkin leaves and cook them before feeding the pigs

W10: In the rainy season, we collect green vegetables and feed them

W1: There is usually perpetual hunger that comes and doesn't end; we feed them pumpkin leaves only. Where can we find maize bran? Nowhere have we also eaten together with the maize bran then.

M: How many times do you feed them per day?

W10: Three times

W5: Three times if the owners have enough feed.

W3: many it is mornings, afternoons and evenings

W1: Especially, when sows have piglets, uhmm!!

W5: They eat

W1: They feed. They need to be fed in the mornings, in the afternoons, they even know that our master can give us feed, following you everywhere you go, even in the house. What remains now is that let me mill some maize bran and increase the water to add to the bran. You increase the water so that after eating, they distance themselves from you

M: What kind of pigs do you keep which you feed three or four times? Is the type that free roams or what?

All women: The ones that are free roaming

W6: The free roaming ones

W7: They don't have kraals

W10: When pigs are used to feeding frequently, they do not go far from house

W5: They don't go in the bush.... Just at home

W2: Even if they may wander, they still come back, they would be lying around

W5: They don't go far

W1: Even if the goats are many, when they come home you give them water. They know that there is our masters house, let us go and drink water...Even if there many!! You just notice they have come, even if there many, they know that now is time for drinking water, and when they come you give them water in a bucket

M: What goodness is there in pigs eating human feces?

Silence

M: What goodness is there in pigs eating human feces?

All women: It is bad

M: There is no goodness at all?

All: No

M: What kind of badness is it?

Pause

W1: The badness?..The badness is that when it eats those feces it contracts diseases.It gets diseases and brings us those diseases at home..Pig disease outbreaks wouldn't end. Sometimes it may go and eat an animal that would have died on its own, in that case can contract the disease outbreak. It is the same with chickens

W6: The badness of pigs is that when you have defaecated, it's like you eat own dirt because when the pig eats feces it may bring it back and I would eat it again myself. It can come and eat in my utensils

W7: and leave those things there

W6: and leave those things there, then I would take those utensils wash them and eat from them myself

M: What do others see as the badness of pigs?

W5: Pigs eating feces is bad. What is needed for everyone is to build toilets. When you protect yourself by building toilets, it means that even pigs cannot eat feces. And building kraals..they may not eat feces.

M: Is pork eaten frequently?

W1: Periodically

A few other women also say periodically

M: Like which times?

W5: During the pig disease outbreak times,

W6: When there are initiation ceremonies

W5, W6: and at funerals

W9: Sometimes even at those times, pork may not even be there

W1: They don't slaughter anyhow...They don't slaughter pigs anyhow. Many times it is *malonda*, they sell live pigs and so that they get money

Malonda = business

M: We are talking about eating...You can sell but you can also eat

W1: When they kill what is called *Kajinya*, you eat some, but for you to slaughter it is not possible

Kajinya = meat from the neck area

W5: That is occasionally. It is not as often as goats. It is goat meat that we eat every day, not pork

M: So pork is not found there at Mtuna, at "*shokas*"?

All (*with W5, W1 more prominently)*: No

W5: There isn't *shokas* at our place

Silence

W5: If there is meat, then it is goat meat, not pork

W10: Not pork. When a pig is slaughtered, it takes long for it to finish because of those diseases

W5: Because of diseases

W7: Many do not eat, a few people consume pork

W10: Few people eat pork

W1: So those that order pigs struggle with them. However, for a goat, goat meat is very popular and doesn't not last throughout the day

M: So these animals that you slaughter at initiation ceremonies and funerals, where do you think they come from?

Silence

W6: From the villages

W10: Where there are no toilets, the same thing of bringing diseases

W5: Even kraals, same thing of bringing diseases

W10: The way they keep them we don't know; we don't know whether they are kept well or not. When you arrive at a funeral, you find pork and they tell you "You who have come from afar, come and prepare this pork?".

W5, W8: ...You just collect and you cook

W1: Without knowing how it was kept, did they have kraals or not? Just staying around and consuming feces

M: If there was to be brought here some meat, what would you look out for? Or if you are not to buy it, what would you look out for?

W1: Pork...Pork, I can see it if it is reddened, I would know that it died on its own...It must show that it doesn't have blood in its flesh, it is *mbee.* And you would say, this is better

Mbee = it is bright or clean

W5: Some of us who do not eat think a lot about the meat…What the pigs eat in the bush there. We think a lot about this that I cannot even touch the meat

M: Some of you may not eat, but your husbands may. So you cannot stop from buying for them because you think about the meat. When the meat is passed, you must bear in mind that here there is someone who eats..

W10: Let me buy, this is relish

M: What makes you to buy or not buy?

W2: They way the meat looks

W5: The appearance of the meat. It doesn't have cysts (*nsembe*), no worms of any kind… Or a handicap, it looks good (*mbee*). That I can buy

M: What kind of handicap?

W5, W1: Like *nsembe*

W6: *nsembe* and a lot of blood

A few women mutter some things not clearly audible

R: Are *nsembe* a disability?

W5: Cysts (nsembe) are a disease itself

R: Are *nsembe* a disability or disease?

W10, W5: It is a disease

W10: The disease is found in the body

I (Dr. Mwape): What about the one without fat?

W10: The one without fat, only have moderate fat, not too much fat, that is the good meat.

W5: Not that one with a lot of fat

W10: Not that one with fat that is over, when you eat it you can get diarrhoea. It brings diseases. Others the fat slab is too much that the flesh part is not enough, there are just oils. If one had to cook it and give it to children, the kids would start getting sick, having diarrhoea

With fat that is over = meaning too much fat

W8: *Laughter*

W5: There is one that doesn't have adequate fat, it is just skin. So if one wishes to buy it, it makes one nauseous and you fail to even eat it. Where would you eat it from? It is better not to buy it, instead just cook boiled vegetables for him even when the meat is there

W10. He wants also to taste the goodness of pork

Laughter

M: Does this pork get inspected?

All: It is not inspected *(more prominently W10, W7 and W5)*

M: Why is that so?

W1: Because here it is a village

Others join in mumbling about the village setup

W1: We just eat anyhow

W10: Sick or not sick, we just slaughter it. When you see the animals the way they look, you think and conclude that this animal is ok. Meanwhile it may be suffering from *chimumphe*. You just buy it and cook for children...You get surprised that those with bad hearts and blood, start vomiting, maybe having diarrhoea or coughing. You may be surprised that where has the disease come from, you would have forgotten about the consumption of pork

chimumphe = presumably African swine fever

"Those with bad hearts and blood" = meaning easily susceptible to getting ill

M: Would you love it if meat was to be inspected?

All: Yes

W10: We can love it

M: Why?

W10: So that we know very well the germs that are in the pork, the health in it, the goodness of the pork

M: Others?

W5: They may have diseases

W8: If found with a disease, they should burn it

W5: Yes, we may not therefore eat such a pig. It would need to be discarded

M: May you not complain if such meat is found with a disease and you are told to burn or discard it?

W7 and other women: We may not complain

W10: We may in fact be protecting ourselves

All: Can love to have meat inspected

M: What about the preparation of meat, how do you do it?

Silence

W1: Preparation? ...We cut the throat of the pig so that it bleeds. This is followed by burning off the hairs and cleaning it well. *Whispers* (that's how we prepare it)

Burning off the hairs = depilation

W5: Making sure that the pork doesn't have cysts, preparing it very well; Even when it has cysts it needs to be well-cooked, boiling it for a long time. That way, those cysts won't be able to cause disease. You may not even have worms if you take good care of cooking

W3: After the animal has been slaughtered, we need to chop it and place it in a dish that is well cared for. Not the kind of dish we wash in, no, but the one used for relish. We need to take it and clean it to allow for blood to ooze out. That's when we put in pot and place it on fire. We place it on fire and after it has cooked, we start to fry it

W5, W8, W1: Yes frying it

W3: Frying it, the way we say it here, until has become reddened (crisp). We fry it until is ready

Laughter

W3: And then add some water. Not only adding water once and say the meat is cooked? It means all the germs that were there have not been destroyed. We need to boil the meat adequately so that we really realise it is cooked before removing from the fire. ...That's how we can prepare meat

Not been destroyed = literal meaning "not burned"

M: How do others prepare the meat?

W10: Others may have a tomato, an onion; after you have adequately fried it, I add a tomato and turn the meat. When a tomato is done I add a chopped onion so that the smell of pork is reduced. Then you start adding water bit by bit until it is well cooked. That's when I remove meat from the fire and place it...

M: What do you have to say concerning *shokas*?

Shokas = method of preparing pork where it is fried, and semi-cooked on a wide home-made pan at a roadside by young men

W1: At *shokas* there is no cleaning of pork... Speaking truthfully

W8: There is no cleaning

W5: There is taking good care

W1: There is no cleaning or taking good care of any kind because he only looks at it in terms of money

W5: It's business

W1: When they slaughter they put the pork in a dish of any kind, other don't choose where they place the meat, even where people bathe from, chopping it there. It is not cleaned at all

W10: and not covering it

W1: Not covering it and putting it for preparation in there

W10: There is no covering at all

W1: When you buy raw one you can clean it at home...That's the goodness of it

W5: Others just go at the *shokas*...

W10: Without washing…

W5: find the meat cooking and getting the meat and placing it in a cup and going with it at their homes to have it with *nshima*. By then *nshima* would already have been cooked. There is no re-cooking it to allow those things to get destroyed. They just eat it directly without correcting it

M: What about the issue of *mateta*?

Mateta = habit of giving nice and good things especially roasted or fried meats to a husband seen as a form of lavishing and endearment

W5: *Mateta* with pork?

All laugh loudly

W5 even clasps hands with W8 and claps her own hands

W10: Let me answer. *Mateta* is roasted meat. If I want to prepare such for my boss, I get a piece and clean it properly in a dish, then I spread some salt on it and if there is a brazier, I put on some charcoal. I get that piece of meat and place it on that fire with good ambers of charcoal and I turn it slowly until it has reached a stage when it has become brownish. I take it and place it on covered plate before taking it to my boss

My boss = my husband

Brazier = metal hand-made equipment that has a receptacle for charcoal similar to a braii

W8: On a wire so that it doesn't get smoky

Loud laughter

W1: The boss would be waiting in the house and even crossing his legs like this (demonstrates)

Laughter

W1: Meanwhile you yourself would have knelt and say "I have brought you some roasted meat

W10: Here is a piece of meat. Even though you don't have a wire mesh you can roast directly

Loud laughter

I (Dr. Mwape): Why is it that meat should have a smell of smoke?

W6: The meat should be put to a boil in a pot, done *fwa-fwa*

Fwa-fwa = brief boiling

W10: eh? *(Looking at interjector)*

I (Dr. Mwape): Why is it that ….?

W10: Smoke? … It can't get smoke on a brazier there is no firewood

W5, W8: That scent doesn't feel good at the throat

W10: When you put on firewood. Smoke irritates the throat

W8: If on a brazier, you need to let the ambers catch fire properly before you put a wire for roasting

W5: Others do not like roasted meat. You get it and slice it properly into strips, get a pan and place cooking oil on it and place everything on a brazier, you also add some salt, and fry it properly before putting pieces on a plate and taking to the *mudala*.

Mudala = husband

W8: *Mateta*

W5: Thereafter, it is *mateta!*

Laughter

M: What can we say concerning these roasted meats? Is it good or what?

W5: With roasted meat, speaking truthfully, those germs don't get killed. It doesn't get well cooked

W1: It is raw inside

W5: It is raw inside, it doesn't get well done

W1: To do it well, it only needs to make *biltong*

Biltong = some form of grilling

W10: It gets dry even on the inside

W6: For germs to finish …

W5, W7: It needs to be boiled first; it gets dry properly by boiling the meat a bit

W6: Even for grilling, you need to boil it briefly first before placing on a brazier

M: Have you ever seen *masese* (cysts)

W5: Yes, I have seen

M: *Nsembe*?

W5: Yes, I have seen them before

M: What do you think about those cysts?

Pictures showing cysts in the pork are passed around for women to see

W5: It means the meat is not good

W8: It looks fluidly, fluctuant with a whitish thing in the inside

W10: It looks white and with a lot of water

W1: There is no way you may say that the meat is good. It looks like that. It would be placed in a dish; meanwhile the cysts would be plentiful. When you touch it, it ripples *(demonstrating)*

The women continue discussing in low tones as they pass the pictures about

M: Have you eaten such meat before?

Most: No we have not

W5, W10: No, it can't even be bought or sold

W1: Even with our parents when they would see such pork, they would discard it

W2, W5, W10: They would throw it away

M: Have you never eaten such meat before? Let us speak truthfully!

W10: We are speaking truthfully.

W5, W6: No we have not

W10: Maybe we ate such pork when we were young. That's what she is saying, maybe we ate, we never know

M: We are not saying those that eat now, but long time ago

W10: Long time ago, we may have eaten, we may not know or beware. We may have been young or grown up a bit; you know how craving for meat can be

Laughter

W10: Yes, sometimes meat with cysts can be brought and you would be told "madam, this meat is ok. There are only a few cysts. When you clean the meat they would come off it. Because of not thinking properly some people just purchase such meat and eat it. You can deny for yourself personally, but not for everyone.

W5: Eating, indeed, we may have eaten in the past. That's why Mr Mwape *(pointing at Evans)* came to investigate the diseases of pigs. He found them because we ate *nsembe* in the past days...

M: That same meat, when you cook it, are they removed?

W5, W8, W10: They do not finish

W7: They remain so

W5, W9: They remain like that

M: What about when you put in the mouth to eat?

W5: It doesn't taste good

W10: The cysts pop up

W9: The meat pops up in the mouth releasing water

M: But you were saying that you do not eat pork, but now you are saying the meat has cysts that pop!

W10: Yes, they pop

W5: We ate in the past, not nowadays

W10: From childhood up to my age...By this time we can deny. However, there are many things we have eaten, we can deny now but we may have eaten

W1: When we were youngsters

W10: Yes, when we were youngsters

W5: After we had some knowledge that pigs can have cysts, it doesn't need to be eaten, it is bad. From that time, that's when I stopped eating pork

M: Madam, have you ever eaten pork with *nsembe*? *(Addressing W9)*

W10: No I have not

M: How do you think it tastes?

W9: It is bad, how can one eat such meat, it is thrown away

Laughter

R: Where is the meat thrown?

W9: Throw it in the bush

Other women mumble

W5, W7, W8: Burying it

W2: Burying it by digging a pit

M: Do you have toilets?

W1, W5: We have

W3, W8, W10: Some of us don't have

W3: Speaking truthfully, some of us had toilets but they fell in (*kudiikila*). When you are alone, unmarried and there is no one to help you construct a latrine, they fall in. Speaking truthfully, I can't say we have or we don't have

W10: Maybe they might come to your home

Laughter and mixed feelings

M: Those of you who have toilets, do you use them?

A few women: We use them

M: Sometimes, we have toilets but we don't use them, not so?

All: Hmmm *(in agreement)*

M: What is the goodness or usefulness of having toilets?

W1: The goodness of a toilet is that when it is constructed, they must attach a door so that when you enter inside you can close it. Unlike others who construct a toilet and put a sack, that sack *(demonstrating a sack acting like a door)* is usually a short one so that when you look outside, it is clear. Now when you sit down and notice that someone is coming, you quickly rise, *mama ine* and go on squirming.

Mama ine = oh my mother

She stands up and turns around as she demonstrates

Loud Laughter

M: The goodness of toilets, others?

W5: The goodness of toilet is this: Men/husbands need to pay attention. He should construct a toilet that has a deep pit and have a proper door. Not building a short and shallow toilet with a short door. When using it if I squat, the underneath of a short door would be open, meaning there is no benefit

Rises and demonstrates what she means

Laughter

W5: It is therefore better to go in the bush… Because it means there is no toilet

M: I said the goodness of a toilet!

All: Hmmmm

M: The goodness of having a toilet, how do you see it?...The goodness of toilets

W3, W5: The goodness...

W10: The goodness of a toilet is: If it is well constructed and maintained and has no problems, it is good because when you enter it no one would know that so and so is there. You enter there and help yourself with dignity and come out. There other kinds of toilets like what these have alluded to, they are incomplete, without a good door, there is no roof, when you sit people notice that so and so is in the toilet. When you want to rise you may be unaware that an in-law is coming to the toilet pressed and without knowing your presence. Before you are done, the in-law is pressed.

Laughter

W1: Pressed…

W10: By the time he tries to enter the toilet and realise it is the in-law, he finds the in-law nude (*Ali chete*).

To be naked = Ali chete

M: The goodness of the toilet?

W5: The goodness of a toilet is that it protects against diseases. There are diseases that arise from lack of latrines. It needs to be taken care of by you personally. After coming from using a toilet, you need to wash your hands. Even a pig can't eat feces if you use toilets

M: Others?

W8: The goodness of toilets is that when you use it you are free unlike using the bush where you need to be turning around scanning. It is good to have a toilet at home

M: What about those without toilets where do you think you go to?

W4, W7, W8, W10: Go in the bush

W4: For us without husbands, we go to the bush

Laughter

W7: Just go in the bush, sit and help yourself and come back home. That's the bush

W4: When you go in the bush you may meet other people. Now what can one do?

Laughter

M: What goodness is there in going to the bush?

All: There is no goodness

W1: You may think that you have hidden…

W9: Someone might be coming behind you

W1: You may be in the process of defaecation, that's when someone is keenly watching you. There's no goodness in going to the bush. It is because of poverty

W5: This time the bush is burnt over

W7: You may think that there is anything good, trying to hide behind blades of grass (demonstrates) and think to yourself that you have hidden meanwhile your backside is exposed and those people coming form that direction can easily see you

Laughter

W6: Toilet should be one of dignity, not going to the bush

M: The other bad thing about going to the bush?

W7: The other bad thing is that when you receive visitors, maybe those visitors are dignified and respected ones. If at the village, they would say go to the headman Mtuna. They would not find a toilet there and instead they would be requested to use the bush

W6: That is not respect

W8: That is not respect

M: Others? The badness of not having toilets?

W1: Just like that, there is nothing we can say. There is no respect accorded to you, you look like someone who does not know good health

W3: When you have diarrhoea, by the time you reach the bush....

W10: ...to reach the bush

W5: You would have messed yourself

W1: Visitors that come to the home suffer, they feel ashamed

W6: Before being given food, visitors that come would first wish to know where the toilet is. They would think that this food that I would be given here, if the need to use the toilet comes where will I go to relieve myself?

W10: Where is the toilet?

W6: They would first wish to see the toilet. Others refuse food and say “no thank you” because of the toilet is not there

W1: Would say I will get satisfied here, where would I go to the toilet?

W5: Should I go to the bush? ...This time the bush is burned over

R: So, when we come to your home tomorrow, are we going to eat some food?

W6: You will eat

R: Are we going to see the toilet?

W5, W6, W7: Yes

I (Dr. Mwape): Very well, the toilet with a sac and no roof

R: Is he going to fit? *(Pointing at Evans being tall)*

W6: He will fit

Laughter

Dr Kabemba stands up

More laughter

W6: In fact, my husband is also tall

W5: Even the roof is there

I (Dr. Mwape): There is no *Nsenga* who is tall as me!!

W6: There are there, tall *Nsengas* as tall as you are

W10: There is Mr Mwanza. *(Pointing at him)*

Laughter

I (Dr. Mwape): This one is short

W1: In fact you have the same height

M: What about the issue of hygiene? Concerning children? Whose duty is it?

W5, W6, W7, W10: It is a woman’s duty

M: A husband doesn’t have any function in terms of hygiene?

W6, W8, W10: He has

M: Hmmm?

W6: He has a duty by looking for soap so that I would use it for washing and bathing the children. He has to buy soap and *mafuta*.

mafuta = lotion etc

M: What can others say about it? Can’t a man take care of children?

W10: Sometimes, you are busy in the kitchen. The husband has power of taking water in the bathroom and washes a child properly. He can also apply *mafuta* and take the child to its mother. Therefore, a husband can take part in children hygiene

W5: Sometimes a woman can be ill or find herself in an accident and the illness is big. You may not be able to take care of the children. When a husband is there, he take care of them by washing the children and keeping them clean

I (Dr. Mwape): They have power or they are needed...?

W5: They have power

I (Dr. Mwape): Do the men do such things?

W10, W1, W5: Yes

W5: When you are in agreement and there is cooperation, these things are done. No, many people when they see a husband wash and clean plates, others say “look the husband was made to eat”.

Meaning was given some traditional medicines that compel him to perform those tasks

W5: Not knowing that that is love. A human being needs to look after children

I (Dr. Mwape): What about if you are well, you are not ill?

W5, W6, W8, W10: He takes good care of them

W5: They are his children

W10: Takes care of them, still more

W1: In the past days were difficult ones. They would say “no, a man doesn’t bathe children”, but nowadays every man is understandable that these children are for the two of us

W5: Gender...There’s gender nowadays

M: At what age do you stop bathing children?

Silence

W3: Size when a child is grown?

M: Yes

W5: It‘s needed that... Others

W3: 9-10 years old. By then I would know that they would have known how to bathe themselves properly. They would do it

W5: It means he/she has grown up

W6: By then they bathe themselves properly and becomes appropriately clean

W1: If it is a child who grows up with wisdom it starts at 5 years. When you want to bathe him/her, you find the child bows the head with shyness like this

Wisdom = roughly translated “ideas”

W3 shakes head in disagreement

W1: Even you the mother realises that this child has wisdom now. That's how I look at it myself.

At 5 years, a child with sufficient wisdom, he is supposed to bathe him/herself…

W3: No, no

W1: ...and you the parents just need to check how he/she has bathed itself

W2: To see if he/she is bathing properly

Others disagree in undistinguished comments

W5: There are other children who are 8 years old but still do not have ideas of...

W6: They can't manage it

W5: ...of taking good care of themselves. You the mother still needs to teach them still. They even reach 12-13 years...

W6: You are still bathing

W5: ...you are still bathing them. It means that child doesn't have issues of hygiene. You alone the mother needs to teach the child, that's when they get jacked up

I (Dr. Mwape): For both girl and boy children?

W5, W10, W8: Yes, all of them it is the same

R: 12 years and you are still bathing them?

W5: Yes, 12 years

W6 and W7 Agree

W5: 12 years if the child doesn't bathe him/herself. Even the mother needs to intervene to teach them how to bathe

R: So, among the children that came *(for focus group discussion)* there were about four that were 12 years old. Do you bathe them? *(Pointing to W7, W6, W5)*

W5: Yes, they get bathed

W10: We bathe them; the kind of bathing them is, sometimes, you the mother would collect the water and place in the house. That child would go out to play. If you haven't paid attention to tell the child that come and bathe, hurry up there is no time, come and bathe. The child can spend 3 to 4 days without bathing

W5: When a child is like that, that's when you are compelled to hold him/her and bathe them

W10: You have to call them "hey so and so, come here". When they get nearer, you tell them "get the water and go and bathe". If you don't pay attention, some children become careless

W6: …They don't bathe at all. What remains is to force them

W10: That's why at that stage we are forced to bathe them until they reach 12 years old

R: They are conflicting sentiments here. To tell a child to bathe and actually bathing a child are two different things

All: Hmmmm

R: What I am talking about is bathing a child?

W3: He is talking about bathing

W8: You can't bathe a 12 year-old

W10: Not a 12-year-old, but 8

R: That's why I was confused and thinking these *Nsengas*

W10: Eight years and below *(repeating it).* Not a 12-year-old she/he feels shy, but only to encourage him/her to bathe

M: What do you think is the goodness of bathing?

W1: The goodness is that when a child bathes often and the clothes washed, a child does not get diseases, doesn't get sick often; The blankets that belong to a child, as you know up to 5-6 years a child would still be wetting the beddings, and what has been said already, they have to be encouraged: To avoid a child from getting sick often, blankets must be taken care of, bathing and washing of his/her clothes; With that coughs and malaria would be occurring far in between

W10, W8: They even grow better, healthier

M: What about the issue of washing hands; during which times should you be washing your hands?

W5: It is needful to wash hands when coming from using a toilet latrine, before eating, why we wash our hands at that time is to wash away the diseases that we come with from the toilet, those disease that we go with in the toilet, those diseases we have; We go in the toilet we find diseases that we go with, not going directly to eat foods without washing hands; At preparing *nshima*, we need to wash our hands so that the dirt that we have finishes

Lunsa= dirt

M: Others who can add on?

W10: It is the same thing; after using a toilet, you need to wash hands with a piece of soap. Wash hands and wipe them

W8: Washing hands, the hands are the ones you use to wipe the bottom

W1: You are at the toilet and wiping yourself *(demonstrating)*, the smell from latrine can contaminate hands. That's why we wash hands. Even if you don't have soap, it is a must to wash hands

W9: The thing you are using to wipe yourself can tear and you mistakenly touch faecal matter

Laughter

W9: That is not good, those are feces. So you shouldn't go with them at home, you need to wash your hands

M: What about after you have come from the bush?

W5, W8, W10: It is the same thing

M: That’s where you go!

Most: No, no

W7: That thing you use to wipe can tear. It is a trip to go and wash hands

W5: Those things that come from the abdomen are bad, there are not good; therefore need to wash hands after toileting

I (Dr. Mwape): You are coming from the bush and you meet the mother to Phalesi. You say “oh the mother of Phalesi!”...You may forget to wash your hands?

Most: No, no

W5: Just at that time when you have come from the bush, you need to wash hands

W7: I would say, I will be come first and go and wash my hands

W5: Even, it is morning you need to wash hands

W10: You make sure to wash your hands

M: What about at eating?

W5: You need to wash hands again

W1: You pour water on your fellow’s hands

W10: In fact, the kind of washing hands at eating is: You get a dish with water in it accompanied by a cup. And another spare dish. You get the water from one dish with a cup and pour on hands of another as he/she washes until they finish; another does the same until all are complete. But not using same dish and all washing hands in it, another one comes to wash in it; it means you have not washed hands. Diseases cannot end like that. Like that you pass dirt one from another

M: What about at the agricultural fields, do such matters get accomplished?

Most: Yes

Most women contribute at once

W5: We carry water

W1: After touching mud, how can you eat *nshima*? Weeding grass, harvesting cobs of maize

W8: That time of mangoes...

W3: She has said at the fields what happens. When we go at the fields, we carry water in a 20-litre plastic container. When we dig a bit and you can’t eat a mango you have collected without first washing your hands. You can’t eat with mud on your hands

W5: You need to wash your hands

W3: You need to wash your hands. Even for mangoes you need to pour water on each other’s hands to wash before eating. You need to wash mangoes before eating them

M: What about in June, when shelling groundnuts?

Most women with W3 more prominent say that it is the same thing

W3: The differences with us humans, others eat with their dirt, others don't. It is each one's own liking. It happens differently depending on particular households.

W10: Others are used while other don't pay attention

M: Have you ever seen intestinal worms?

W10: I saw them with my child

M: Where did others see them?

W2: At home

W9: In children that went and passed worms in their stool. That's where we saw them

M: Where do you think they come from?

W5: Worms come from food.

M: Which kind of food, W5?

W5: For example from rape, intestinal worms come from there; from Chinese cabbage, from leafy vegetables

M: Where do others think they come from?

W1: Vegetables from gardens, because such vegetables get sprayed with chemicals. Those sprayed chemicals do not come off, even when we wash them; they still bring about worms in the abdomen.

M: For those that passed intestinal worms, what do you think they complained of?

W5, W2, W8: Abdominal pain

W5: …And eating, feeling hungry fast. When they eat after 2 minutes, they could say I am hungry and need to eat *Nshima*. Even shortly after consumption, they could demand for more *nshima.* They complain of severe abdominal pain.

M: Others, what were they complaining of?

W10: Others would complain of abdominal pain, and poor appetite. They could only eat a bit and then stop as they say "My abdomen is painful". Sometimes not knowing that it is abdominal worms

W3: The abdomen becomes filled up and hardened

W1: And also a child with worms does not look good. It has a painful body and one would know that this child has worms in its abdomen

M: Do you think this disease of intestinal worms can be prevented?

W5: They are prevented at the clinic

M: How are they prevented?

Silence

W1: Going to the clinic and say or speak of your problem

W8: They would be given medicines

Women are showed the pictures of tapeworm proglottids

W9: They would be given medicines according to that ailment that has been diagnosed

M: What about cure? Does the disease get cured?

W5: Yes, they get cured

W6: When you have paid attention

W5: When you have paid particular attention

W1: When you go early to a clinic, disease is healed; if you are late to be taken to a clinic, others may not be healed. Because by then they would have reproduced and make a nest (clumped together in a ball) like this *(demonstrating the size)*

W5: For others, it is blood that reduces. There are worms that suck blood

M: Have you seen those pictures?

All women: Yes

M: What do you perceive them to be?

Murmuring

M: Have you seen such?

More murmuring and saying inaudible comments

M: Have you seen those?

Silence

M: That is human excreta!! Have you seen such in human stool?

W6, W8: I have not seen *(whispering)*

M: Not in the toilet, but human stool. Have you seen such?

Stool = the *Nsengas* here use the term *chimbudzi* interchangeably to mean either stool or toilet

Some women: Hmmm (not with conviction)

M: Have you seen them before?

Some women: Hmmm *(even lower than before)*

W8: I have not seen them

W1: I have seen it in a child. He/she passed them as a heap together with feces.

M: Like that? (Pointing at pictures)

W1: Yes. Others are small like this *(measuring with her left thumb finger)*

W5: Roundworms!!

W1: Others are like earth worms, clumped together when a child defecates

R: Like caterpillars or a peel of a snakes' skin!!

Silence

W1: A peel of the snake's skin..?

R: Very short ones....those are the ones

W10: Hmm. I saw them, very small ones

R: Where?

W10: In Ndola. There was a child in the neighbourhood who was fond of scratching its bottom. After scratching the bottom...

W5: Sometimes it is itchy

W10: small worm would come out on its own looking like a snake's skin peel in a child's feces, white in colour

R: When was that?

W5: Others they come from the mouth

W10: A long time ago

W1: I have seen such nowadays

W5: These are the red ones *(handing back a picture)*

M: Is having such a disease a fearful thing?

Most women: It is a fearful thing!!

M: Is having worms fearful?

Most women: Yes

M: How fearful is it?

W10: They bring germs in the abdomen because when a human being…

W5: Or even dying, you can even die because other worms suck blood. Even dying there is possible

W10: Even where they bite in the intestines, what remains are some ulcers

W4: I had them myself...

W10: You need to have them prevented against

W4: I had worms myself. If they did not come out I wouldn't have given birth. When I would eat a bit of food, my abdomen would be full.

M: Do they prevent child bearing?

W4: Yes...yes

Laughter

W5: When they are plentiful in the abdomen, they even sit in the uterus

W10: A nest is like this *(making a shape like this, making a fist).* I saw it in my child. When you defaecate a lot, when you have passed them a lot, at the end it is a small nest of them that drops looking like small earth worms inside the nest.

M: Do you think there are other diseases more severe than intestinal worms?

W6: Hmmm

W5: They are there

M: Like which ones?

W5, W6, W7: Epilepsy, fitting

M: Others, what do you perceive are the severe diseases?

W10: There are those of epilepsy

M: What about the less severe ones?

W5: Headache, sneezing, those are the less severe diseases, malaria

W1: Increased body temperature

W10: You feel a headache

W1: Sometimes not eating when the temperature is very high

M: In your village, are there many people who have worms?

Silence

W1: We may not know

W5, W6: We may not know

Pause

W1: We may not know

W4: There was someone, a grandchild of mine *(showing the approximate height and small stature of the child)*

M: What about in your daily life, if you have intestinal worms, what kinds of problems/challenges can you face?

Silence

W1: The problems we can meet, if you have worms in the abdomen; the problems we can be faced with can be frequently getting ill...

W2: Not eating properly

W1: Sometimes you do not have hunger *(inappettance)*, sometimes you may want to eat, but a short while later become inappetant. Whenever you want to eat, you experience a pinching pain in the abdomen

W2 agrees

W10: And making abdominal noise

W1, W2: Yes

W10: But the abdomen is not painful

W10 mimics the abdominal sounds-*chololo-chololo*

W5: Sometimes, having a swollen abdomen... sometimes diminishing blood

Diminishing blood = anaemia

M: Others? What kind of problems can we meet?

W5: All those are problems

W3: You can meet some problems where you can die. When they are many after reproducing, and if you vomit and you bite into them, macerating them, you can die from their poison

W1: Even a child, when it vomits them, it is danger. When it is passing them in diarrhoea it is better off

M: Are there people that died?

Silence

W1: When they bite in them?

M: Yes

W1: When they bite in them, they die

A few women say things in a very low voice seemingly in agreement

M: Have you seen them?

W10: Yes, a child.

W2: Especially children

W10: I saw a child where we used to live in town.

W1: This size *(demonstrating a measurement of a child that a toddler or a few years old)*

W10: The child used to vomit a lot, in the night, it would come to the throat *kha* and t would fall. One time it came when the child was asleep, it came out and reached here *(demonstrating half way out of the mouth).* When the mother woke up to hold the child, she found the child had bitten and cut it off. When the child was taken to the hospital, the child died.

kha = a term depicting that the worm is stuck

M: Do you know what epilepsy is?

Most women: Hmmmm

M: Where do you think epilepsy comes from?

Silence

W5: Many times, epilepsy comes from pigs

M: What do others, think epilepsy comes from?

W6: Others are bewitched; it comes from people

= meaning witchcraft

M: What do others think, epilepsy comes from?

W1: Some of us know that epilepsy is familial. Others make traditional medicine, there is no one who cannot pass through that type of epilepsy; it runs in the family, it comes as a result of being bewitched. Another one will have it, yet another will have just like that.

M: What do others think it comes from?

W10: Similarly, it is found in families. Even when it was not found in families, others may not have it, but it changes or jumps from *Njiru* to epilepsy. Saying "so and so has epilepsy".

Saying "so and so has epilepsy" = tending to occur in more members of a family than expected by chance alone

W5: Another type comes from malaria..When you are sick and you have elevated body temperature, you fit

M: Do you know of people with epilepsy?

W5: They are there who have epilepsy

M: Where do you think such a disease came from?

Slight silence

W10: enhu *(shrugging off and laughing)*

W2: I think malaria. When the body is very hot, one can fit

W5: Others it is because of being bewitched as we have already said. That's where it comes from many times

W1: That type of epilepsy that comes from malaria, if you rush and come to the clinic, it is cured

W3: It gets cured

W5: the one that has come because of being bewitched it doesn't get cured

W10: It truly does get better

W5: If it is the bewitched kind…

W10: It means every month one has to "fall".

One has to “fall” = fitting and falling to the ground

W9: It means it is the bewitched kind

W10: If a child or an adult comes to the clinic and after being given drugs, they get better. If it recurs at home, then it is not malaria that caused the epilepsy

M: Do you think this disease of epilepsy can be prevented?

W5: If it as a result of malaria and one fits, they get cured. The bewitched type doesn’t get cured

W2: It doesn’t get cured

M: Protection, Prevention...Can it be prevented?

Most women: Yes

W1, W5, W6: Not the bewitched type

Murmuring

W1: There are many we have seen

W3: Medicines can be found and the person gets cured. That time before one has fallen on fire, getting burned, medicine are found and a person gets healed

M: We are talking about prevention. Do we know what prevention is?

W6: They cannot be prevented

W3: How can one prevent it?

M: Like you, you do not suffer from epilepsy?

Women: Hmmm

M: Now so that you do not suffer from that disease?

Silence

W5: Preventing here at the clinic...They can be prevented

W3: Meaning you do not suffer, but now you want to prevent yourself

W10: They can be protected from the hospital, if disease is not coming from anyone else, they way they were doing it for measles…They used to get protected

M: What about getting cured? Are there some who get cured?

Brief silence

M: There is no one who gets healed from epilepsy?

W3: There are some who get cured

W5 There are some who get cured

W8: Others get cured while others do not

M: Where do they get assistance to enable them get cured?

W5: Others from *chibulaki* (black medicines) others from *Chizungu* (conventional medicines)

chibulaki = black medicines; Chizungu = conventional medicines

M: What kinds of drugs are given so that they get cured?

W1: If it *chibulaki*, they get roots from the bush…There is way they know how to dig for them. They give them to you and you are told how to make/constitute

Silence

M: Is it fearful to have this kind of disease?

All women: It is fearful

M: How is it fearful?

W10: It is fearful. Sometimes you can enter your bedroom and you sleep on the bed with your husband. The husband now says "this one is my wife, let's eeh. This help now". The husband thinks it's time/ready now, meanwhile the wife is fitting

W7: he is busy *(mimicking fitting episode)*

Laughter

W9: He is inside you

More laughter

W5: Equally, a wife can say that with my husband today we will make love, sometimes you find that the wife is fitting. The husband can be left alone, or maybe it is husband who is fitting!

Laughter

W5: As a wife you are left like that!

More laughter

M: What other dangers can there be?

W8: A marriage can end because of fitting

W5: Others get burned

W10: At the fireplace

W6: Falls in water

W1: The other danger that can be there is that you may say I want to be with my husband, but you may think that this is what he usually does, perhaps he dies…eee.

Mild laughter

W1: They may think of you… *(Coughing).* They may suspect that you may have killed your husband.

W10: That you put drugs in his food

W1: That you killed him, forgetting that epilepsy… its time has come that he may not wake up. So if it is husband and wife, we manage that marriage be there. If it is a child, you may say that Satan who has given me this handicap/disabled person, let me just have it

M: Is it a good thing for those people with epilepsy not marrying?

W1, W2: It is good

M: hmm?

W1: It is good not to marry because if one is working like our male counterparts. At the workplace he may work well but afterwards on his return, *khu*. It may be on a bicycle or a motor car. People may be shocked; all the people can be surprised. Ehh, that time it becomes another problem. If he goes by bicycle, it becomes a problem; perhaps a motor car can come and run over him. That's why those with epilepsy there is no good luck or well being that they find.

Khu = sound of falling down.

W10: The one with epilepsy is not good going without being married. He/she is a person, he/she is complaining because he/she was made by God. He/she was made to have fruits. His/her body is complaining because of that illness that prevents him/her from having a partner at a home. So he/she is not happy, he/she may be free. But for those that observe may think it's is good for someone to be alone, but herself/himself envies how others live in their families. It never made him/her happy to be like that.

He/she = W10 used a term that is unisex, and can mean both man and woman

W5: I can also say this. You said it is a happy thing not to be married or marry?

W8 laughs

W5: My in-law, do not make me laugh *(as she briefly laughs herself).*

W10: It is not a happy thing

W5: It is not a happy thing for one to be single

W10: Yes

W5: It is not a happy thing, but at that time of when a husband has reached the love part (*pachikondi)*, don't you know that's when fits come?

Pachikondi = roughly meaning reached arousal or great excitement

Laughter

W5: At that time when you would say let me eat now…

Laughter

W5:…That's when epilepsy (fitting) comes. It is better to be single than the two of you because he will kill or dampen your desires…

W10: Because the heart is complaining

W5: The heart complains indeed, but you want (to make love) but him/her is fitting. It is dampening or destroying the desire!!

Laughter

M: W5, if it is you with epilepsy?

Laughter

M: If it is you with epilepsy, how would you feel?

W5: I may not feel good because

M: …so that people don't speak with you? (or roughly not proposing to you)

W5: I may feel good because I may be killing my partner's desire. I may want my partner to be happy or celebrate with me, but I may be fitting

W6: …Fitting…

R: This issue of the bedroom seems like it is number one?

W5: Yes

R: Is it always possible that someone fits whenever one wished to make love?

W5: No

R: Eeh?

Mild laughter and low contributions

W5: A time comes but some times...

R: How I have understood it is that every time one wished to make love, one fits

W10: What he is asking is that it is good for someone who fits to be single? That's why O said it is not good at all because one a heart of complaining, because when God created that person He wished that he/she would have someone to help her/him. However, he/she doesn't find marriage because of the illness that she/he has, so that destroys ones future that she/he gets helped…So even that doesn't gladden him/her

I (Dr. Mwape): So, if one fits only once…

R: Ehh!

I (Dr. Mwape): …Does it mean that marriage has ended?

W10, W5, W8: No

W5: If one is bewitched, others take part one fits everyday so that the marriage ends

Others take part = roughly "touch there"

W10, W8: ...ends

W5:…Have you seen? But if you are not bewitched, it comes after some time or rarely.

W8: Rarely

W5: However, for the bewitched, it happens. When you are ready, one fits

W10: But it is not common in marriages

R: Have you heard of this, or you are just talking about them?

W5: I have heard

W8: Even observing

W5: I have heard of them, even observing them that yes here. For the bewitched

R: Have you observed? Are you there where the two are?

Loud laughter

W5: I only hear what happens

Laughter

W5: It happens

Even more laughter

W1: Because as a family we stay together for many years. When you marry each other both of you are fine, even the woman, but this disease comes and you cannot say this marriage should end.

W10: It can't end

W1: Because the disease has come when you are together. At that time, marriage should not end. When times are good, you stay together, when you have found good times,

W2: Keeping or caring for each other

W10: …You work together acts of a home and make love, when he is feeling better. However, when the disease comes, you just know that here my partner is ill

W10: Problems

W1: It is the same with a wife. He can't divorce you, at that time you are united. But if you hear that this one has epilepsy, why would you accept the proposal?

Laughter

W10: You can't accept

W1: That one doesn't work

I (Benjamin): You can love him

Some women disagree: no, no!

Followed by laughter

W1: You love the one at home, that's the one you can put someone on the back like. I the one who is speaking, my husband doesn't have eyes. When marrying me he was alright, because shortly after we have grown older, he became handicap, going with him to several clinics, it failed completely. The sister knows very well, not so? *(Addressing Moderator)*

Pause

Moderator doesn't respond

Laughter

M: Do you think the illness of epilepsy? Do you think there are diseases more severe than epilepsy?

W5: There is none. Epilepsy is number one

M: What about the less severe ones?

W10: The less severe one s are, malaria, sneezing, coughing and suffering from headaches

I (Benjamin): Even HIV does not match with epilepsy?

W5: HIV is preventable, it has its own type of medication, it is curable

W10: It has prevention

W5: ... But not for epilepsy

W6: The one with HIV gets married but not one with epilepsy

W10: The problem with having epilepsy is that many sometimes end up getting burned

W2: If one is drawing water and experiences a fitting episode, she may fall in a stream

W5: It means dying

W10: There is even no marriage like that

W7: Just hearing that that one who was seeking you for marriage was seen fitting at the roadside, you say to yourself, ooh that one who fits…no

W10: Marriage fails

W5: Marriage fails completely

W3: I have a grandchild. I know the nurse knows *(Addressing Moderator)*. She stayed here, admitted in the ward (at clinic) coming from *Ulemu*. That one who got burned and was short of getting eyes blinded..?

W5: That girl…

W3: That's the one my grandchild had married. He left three children with her

W6: Twins and...

W3: It was very difficult. Every time she would fall, a short time she would fall.

W1: Are they divorced now?

W3: They divorced each other. Her family freed her. She was just sited at home.

W2: It is true

W3: That she fits, what can we do? The in-laws and their family sat down and said, now, it looks like we are troubling the other one (the husband). Our child does not perform any duties. They used to stay like that, years and years, with their hairs (pubic hair), even having children

W1: There was no one to shave the partner (pubic hairs)

Laughter

W3: The husband would not want to care for the wife for fear he may be given a charge. For a wife to care for the husband it was not possible. How would she do it, because she is sick?

W5: Nothing. Hands would be stiff

W3: They said; let us feel pity for our friend. So, they freed or liberated the husband. He is now remarried in Kakwiya. She is just sited at home, she cannot get married

W2: It is a child from our family. *(Addressing W1)*

M: What do you think are the difficulties that a person with epilepsy encounters in her/his daily life? How do you see it?

Silence

W1: The difficulties that one may meet are: Because that person is being kept/ looked after. Now for someone to look after her, the way she would take care of oneself, it becomes difficult. That one is a difficulty. Even those care givers can say "I am tired also!" or say "let that person be. Hey! you go away". Meanwhile the victim would be fitting, while the caregiver just goes away. That one is difficulty also because the person who is taking care has put on a heart of Satan and failed to render proper care to the victim

W5: Dying…There is also death of parents who take care of that person. There would be lack of caregivers if those that were caring for the victim die and the person has remained alone...All this is a difficulty that can be encountered.

M: Others?

W10: Even that person has family, if he is an adult. May be the family may comprise only of women. That person can be left fitting, sometimes even in the nude. A male child may be looked for to care of him instead of a female one. The victims of epilepsy also grieve or complain, especially those without families. If the victim has a wife or husband, he or she may find the partner caring for them, may bathe them, wash their clothes like that, properly preparing their food. Now if he has no wife, he is single, he has a huge difficulty at home because he has no source of assistance

W1: Our friends with AIDS have helped as long as they go to the clinic.

W10: Finish

W1: They drink the medications and are able to work for themselves better than those with epilepsy

M: If the government brought in laws that in order to prevent the disease of epilepsy or intestinal worms, we need all of our pigs confined and not to be let free. We need to attach a …

W1: …a fence

M: …a fence, so that they just use the area within the kraal. What kind of difficulties can be found?

W1: To speak truthfully, speaking truthfully, it is good but we may suffer from lack of those to construct them. Because, you see, during Welensky's time such a law was passed of building a fence for pigs so that they would not go outside. Some of us even kept such pigs which were not coming out of fences, giving them food inside, during that time diseases were not commonly or frequently

found. Speaking truthfully. Now what can trouble us these days is that our fathers used to have skill/talents, not the men that we marry nowadays, they don't have skills. Even constructing a chicken run can be met with a lot of difficulty.

During Welensky's time = a governor of Northern Rhodesia, before Zambia got its independence signifying the period before independence

M: What do others say about it?

W10: Some of us, some men have ears, they can understand. To find protection of pigs can be met because what the government can say they would understand because pigs go in the bush to eat feces and come back to eat from the kitchen utensils. Ailments do not finish. So, they may understand that a kraal is built and a fence made so that pigs would stay inside. It can be of benefit

M: What are other difficulties that can happen to us?

Silence

W1: Let us now use our heads there

Giggles

M: Feed! Can there not be any difficulty with pig feed?

W3: Feeding can be a problem. Also 2) those pigs just wander and loiter about in the bush. Now to just decide that time and start to confine them, you may find all of them have a pig disease outbreak. Even the joints would be become weak, fail to walk and get polio

Laughter

W3: They are not used to stay in the kraals

Laughter

W7: They are used to just roam about...If they had to stay just for one week, they would start having limb problems

W3 All of them would fail to move

Laughter

W7: They would develop polio and limbs criss-crossing like this *(demonstrating with crossing of her hands)*

W5: If there is unity and cooperation with the men, there would not be any difficulty...concerning pigs

M: Now if they say, all of us should build latrines and also to use them, what do you think is the difficulty that we can encounter?

W8: They would not be any problem because we need the toilets

W1: Similarly, we may find those to dig pits for us

W4: Us without someone to dig or construct, we suffer

W1: Anha, and the one to construct the house. Sometimes you may find a piece work for one to dig the pit but may fail to afford one to build the structure to house the pit. That is a difficulty

I (Dr. Mwape): Now, if you have pigs, would you not sell one of them to facilitate building a latrine

W1: You can sell and use the proceeds to build latrine

W3: Those hired persons refuse. I started a long time ago, since my latrines collapsed. I am not used to be frequently going in the bush. I usually start to construct another one the moment I realise the one I am sing has become half full. However, nowadays the hired men refuse; you may have money and tender it. They would say, "Why don't they get married so their husband can do it for them?"

Laughter

W3: Now looking at my age, where can you find a man to be married to?

Loud Laughter

W7: What they are saying is that it is good at the bush. When you say there is a job here, they would reply that it is good going in the bush

W10: They feel good that when they go to the bush, the pig is following behind

W5: They go in the periphery. When they squat, the pig is behind them

W3: Some of us are not used to such, we feel shy

M: What about if there is law that after you have purchased meat .you need to have it boiled thoroughly, what difficulty can be there?

W10: There may not be a problem. Because it is one way of protecting against some diseases we are found with

M: If they say, all leafy vegetables, from rape to pumpkin leaves, cabbage, spinach, you need to wash them; what difficulties do you can be there?

Most women: None

W10: None, because even now we wash them

M: Even pumpkin leaves, we may not complain?

Most women: No, we wash

M: Do you wash pumpkin leaves?

Women: Yes:

M: Even when it has no dirt?

Women: Yes

W5: Even without dirt we still wash

W1: At our place pumpkin leaves with all those snails, you cannot cook without washing it

W5: You cannot cook it like that; it means you want to eat it with snails

W3: The rules about vegetables are that they need to be washed every time. Even when it is clean, the rule is that it needs to be washed

W10: It must be washed

W3: Most of them must be washed before they are placed on a fire

W8: Others wash, while others do not wash

W10: Others do not wash

W8: Others do not wash…

W7: They say when the vegetable is washed, it dries up. Even when it is clean, hits just like that place on fire

W3: Even tomato or onion I put in relish but a rule is that I get and wash them first

W10: I put in water

I (Dr. Mwape): At the beginning, R explained that do not to say "others" but yourself

W10: For me or by myself!!

I (Dr. Mwape): For yourself…

W10: Do you wash?

W3: That's why we are saying we wash

Laughter

W3: The way we got used, we were raised like that by our parents, we grew up like that

W10: I wash before I put in a pot

M: If they say, meat needs to be inspected, any meat from a slaughtered pig, they need to come and inspect. What kind of difficulties do you think you can find?

All women: There would no difficulties

W1: We can be happy that they would come to inspect.

W8: We were asked

W1: Were we asked?

W8: Yes, we were asked about what to do when a pig has been found with a disease. It is to get it discarded and burnt. If it is alright, we cook it.

I (Dr. Mwape): Would you be happy if they requested it (pig) to be burnt?

All women: Yes

W5: We can be happy because they would be protecting us against diseases

W10: Even for adult or big cattle, when found to be diseased are destroyed or buried. What more a smaller pig...it is to bury it

M: What about if they say we need to be hygienic people? On our bodies, what kind of difficulty can be found?

W10, W5: There wouldn't be any difficulty

W10: There would be a difficulty for those who are not hygienic, used to dirt; they are used to eating dirty things...

W3: With their *lusa lake*

Lusa lake = with their unhygienic tendencies

W10: ...with their unhygienic tendencies. Those who are clean may not find it difficult because they are used to being hygienic.

W1: It is the same with toilets. Those are used to having a toilet, they have them even now. Those of us who would say "the government is just troubling us, they is nothing they are telling us". Some of us refuse…

W5: In the bush

W1: Even the government is not telling us anything of substance, those of you who wish to dig toilets...

W5: Go on and dig them

W8: It is also the same with the issue of vegetables…

A discussion of using the bush ensues. It is disjointed and barely audible

W8: going in the bush

W5: It is difficulty especially in the rainy season when the rain drizzles, and you are pressed to go in the bush, now even the dew is plentiful at that time, like if you go you are clothed in it

M: What about if they said everyone who is suspected of having intestinal worms is needed to have prevention/vaccination? What for you is a difficulty we can encounter?

W5, W10: there would not be any difficulty

W10: That prevention enables that disease in the abdomen to be removed, you become well treated

M: So, even if we came to Mtuna village to administer medicines, would you attend

W10: Even today

W5 and a few other women: We can come

W1: What can hinder us? There are some who understand well and other who do not understand

A brief spontaneous debate about some who came and attended a one week measles vaccination/child health week and others who may not

M: What about if they said all of our pigs need to be vaccinated, what kind of difficulties can you meet?

Women: No problem

W10: That is when the prevention of pigs would be done better. Frequent diseases can be eradicated

M: If they said you need to partly contribute, no matter how small?

W3: That's where the problem would be, because that small thing *(kangachepe=some money)* is what we are looking for

Laughter

W5: speaking truthfully, what else could you do?

W10: Sometimes, the government might say give a small contribution, say K20,000 (about $4), suppose you have a lot of pigs at home, say 20 or maybe 30. Now each pig would need a K20,000, now you do not have, what would you do? That way, there may be a problem

I (Dr. Mwape): You sell some of the pigs!

W8: selling some of the pigs…

W10: I am not sure

W3: Now suppose you only have one pig, can you sell that same one? Which pig would get vaccinated then?

W10: What vaccination are they going to give it?

W8: It means it is a problem

W10: Yes, it means that would be a problem

W8: You would remain with a K20,000 in the hands

Laughter

M: I believe we have come to the end of our programme

Women agree while some are still laughing

Ruth summarized at 12:14 hours

M: We were learning some things about pigs, we discussed the goodness and the dangers of having pigs, why we keep pigs, what other kinds of livestock we keep, we also talked about those that feed pigs, the ones who make decisions about whether to slaughter pigs or not, we also discussed inspection of meat, also talked about issue of epilepsy, issue of intestinal worms, we also tackled issue of toilets, the goodness of hygiene, and finally we also talked about how to prevent those diseases of epilepsy and intestinal worms, what we ought to do, building kraals so that pigs are reared inside and to not come out, bathing; we also discussed inspection of meat, taking pigs for vaccinations or prevention and all the people to be taken for prevention or vaccinations.

Are there any questions or additions?

Silence

W5: What I can add on is only to tender my gratitude for bringing this programme. We have learnt as well as teaching each other concerning living and keeping of pigs. Some of us do not keep pigs.

We have learnt, maybe we may keep some pigs. I would know that I need to do this and that to keep pigs…That is what I can say

W1: We are very grateful in that you asked us
