# Supplementary material for: Why Latrines Are Not Used: Communities’ Perceptions and Practices Regarding Latrines in a Taenia solium Endemic Rural Area in Eastern Zambia
Source: PLoS Negl Trop Dis. 2015 Mar 4;9(3):e0003570. doi: 10.1371/journal.pntd.0003570 (PMC4352092; doi:10.1371/journal.pntd.0003570)
Supplement: S1 Dataset — (ZIP) [file pntd.0003570.s001.zip › FGD transcriptions_Zambia-2010/Chiluzu_Mr Sakala/Women_Chiluzu_04-08-10_Revision.doc]

**Title : Focus Group WOMEN**

**Date :** 04/08/10

**Site :** Kakyiwa Rural Health Center

**Village :** Chiluzu	**Location :** Petauke district, Eastern Province, Zambia

**Duration (total time) :**	77 min	**- Start :**	10:09 am	**- End :** 11h26 am

**Participants:** 8 women

**Informed consent:** signed and available

**Moderator :** Ms Ruth Chiwa	**Recorder :** Dr Andrew Phiri

**Transcription/translation :** Dr Sakala

**Abreviations :**

**Mod : moderator, R : recorder, I: intervener, C : children, W : women, M : men**

**Disposition (from the left of the Moderator):**

**M**

W1

**R**

W2

W3

W4

W5

W6

W7

W8

Age composition:

W3: the oldest

W7: the youngest

All of the rest: middle age (Mother)

Typology:

W2: expert

W3: dominant

W4: shy/quiet

W5: funny/dominant

**Comments about participants:**

One keeps pig and 7 are used to keep pigs

**Group dynamics:**

**Text:**

*Introduction:*

R: advised the women to speak up and not hold there mouth when talking because they will be laugh at by their tribe cousin

I (Dr. Kabemba): I will punish them if they do not speak up!

*Laughter*

Mod: Greets all the women present

All: Greet back

Mod: Thanks all the women present and welcome them to Kakwiya village, and said the first thing we need to do is to know each other.

Mod: Do you know the visitors?

All: The women respond that they do not know them since they have not yet introduced themselves.

Mod: Have we known each other?

All: yes

Mod: Thanks all. She asks all the women to be free and participate in the discussion very freely to talk on what they know, they have heard of and those which take place at their homes especially those which they know about. Informs the panel that our topic of discussion is on pigs and asks if there is any who keep pigs?

All: The women respond together that yes there are some who keep pigs.

W3 says that she had recently acquired some pigs (all other women say that their pigs died of swine fever recently)

Mod: says she had heard about the sickness.

*Discussion:*

Mod: Are pigs needed in our village?

W3: Yes, pigs are needed in our village?

Mod: Are we all agreed that pigs are needed in our village?

All the women agree that pigs are needed in our village

W6: Pigs are very necessary and help us in many ways

Mod: In which ways do pigs help us in the village?

W1: Pigs help us in so many ways if we have problems

W3: Pigs help us in many ways like if you rear pigs and the sow gets pregnant and gives birth, some of the piglets would be males once these are castrated and reach maturity you can sale some and use the money to pay school fees and sort other problems which may need your attention.

Mod: Any other suggestions?

W5: Pigs can also help the family in times death you can kill a pig and use as relish for the mourners and also in cases of sickness you can sale a pig and use the money for transport to take the patient to Katete Mission hospital.

Mod: Any other contribution?

W2: You could slaughter a pig and eat the meat at home.

W3: Also pigs are helpful if you receive a message from town, you sale the pig and use the money for transport.

W7: When weeding becomes tough in the field during the rain season, you can sale a pig and use the money to hire people to help you with the weeding.

Mod: Is a pig a good thing?

W3: Yes a pig is a good animal but the disadvantage of pigs is that when they give birth and the piglets are almost reaching maturity and swine fever strike then you loose everything.

W5: Pigs are okay because they help us but the problem is that they eat everything they find, that way pigs are not good.

Mod: Like what do they eat that is not good?

W5: The badness with a pig is that like here not many of us have toilets, so when a child wants to pup, the pig would stand there waiting for the child to finish and feed on the faeces, then we would kill the same pig and eat its meat. That is what I find bad with the pig.

Mod: What are other bad things associated with a pig?

W7: Other pigs have worms and therefore transmit dieses to humans.

Mod: What other bad things that is associated with the pig?

All: it is its scavenging nature that we abhor

W2: Yes and pigs can go to the graveyard where the burry still born babies dig the foetus out and start to eat.

Mod: Do you just hear rumours or this happens in real life?

All: These things happen in Muzalema in Katete even near here in Sikwenda village some years back, people witnessed these things.

Mod: What is the job of a pig?

W7: does a pig have any job?

W1: Pigs have no job. The only job they do is what had been discussed by other to sale and help one sort out their problems.

W3: That is the only job the pig does, they can not perform any manual work.

Mod: What warnings would you give regarding a pig?

W7: If you slaughter a pig and start eating the meat before it is inspected by a health inspector you would transmitting a disease to the community.

Mod: Anything else?

W4: The pig is a carrier of many diseases which can be transmitted to the people.

Mod: what else?

W2: It is true that the pigs during the swine fever some people do not throw away the dead pigs but they process them and start selling the meat to unsuspecting customers.

Mod: What are supposed to do if a pig dies from swine fever?

W2: Such pigs should be buried or burned.

Mod: Do you do that?

All: To be honest most of us just eat the meat…

Mod: Do you keep any other livestock apart from pigs?

All: Yes we do.

Mod: Like what animals?

All: goats, cattle and chickens.

Mod: On your own, not with your husbands?

W7: It is rather difficult to say because there are times when animals are bought for the home.

It means that the husband buy some animals not just for a person in particular

*Laughter*

W5, W6: Yes, sometimes livestock can be bought for a child, a chicken or cow may be bought for Mary, it therefore becomes very difficult to establish the sole owner.

Mod: How about others?

W3: Yes, I have my own animal and so does my husband. Even my children have animals of their own. But they are all being kept in the same family kraal.

Mod: Why do you keep all these animals?

W3: We keep them to assist us when we have problems either you sale whole animal and you solve the problems or you slaughter and sale the meat.

Mod: Why do you keep these animals?

W7: We keep them to assist solve some financial problems, we reduce poverty in the home; sometimes you exchange your livestock with other for kitchen utensils.

Mod: Like what kitchen utensils?

W7: Utensils like dishes, pots and plates.

All: We also exchange for buckets.

Mod: How about clothes?

All: Yes even clothes.

W5: Especially chickens are very good to keep at home because if a child is suffering from Malaria and refuses to feed, you can easily slaughter a chicken and you would find that the child would start feeding and by the time the chicken finishes, the child would have recovered from Malaria.

Mod: Any thing else, W4 can you say something?

W4: Chickens help in many ways.

W2: Chicken helps during funerals. You slaughter chicken for the mourners.

W7: Chickens can be slaughtered for the guest during one’s daughter initiation ceremony and in many other numerous ways.

Mod: You do not rear them because you want to show off that you are rich in your village?

All: no.

W6: Would you say you are rich because you have a chicken?

Mod: No I mean all animals?

All: No, we rear them because we need to sale them and use the money to solve our problems.

W5: The livestock we keep helps us and also show that we are able as persons to solve our problems, and also demonstrates how responsible we are as individuals.

Mod: In the rearing of pig what role did you play even you whose pigs died, what role did you play?

W7: Our role was to feed the animals, build kraals for the pigs…

Mod: Do women build Kraals?

W7: It is the men who construct kraals.

W2: In the past, the way we used to keep pigs was that we just opened the kraal and let the pigs out to scavenge around the village. This led to a lot of pigs either being stolen in the bush or dying a thing which made a lot people loose out. It is only very recently that we have been taught to keep the pigs in a fence; that is why many people do not have pigs because they lost them to diseases or were stolen in the bush.

Mod: Are there any other people who have come to teach you about pig keeping?

Most women: We have never heard anyone teaching about pig keeping

W2: I’ve heard from some people and I’ve also seen a poster at the clinical teaching about pig keeping.

Mod: congratulation W2, you are very observant! Which other persons help you in the rearing of pigs?

All: we do it alone.

Mod: You do it all alone?

All: The men help us; they are the ones who construct the pens.

Mod: the children?

W6: Yes, they help if you are not around. The children get maize bran, call the pigs and feed them, then close them in for the night.

Mod: Who makes a decision which pig should be sold, slaughtered or used for weeding in your homes?

W3: Most of us are married; some of us are also not married. For us who are married, the men make the decision *(laughing)* a woman can not make that decision on her own but in most cases it is done in consultation with your husband.

W7: The decision is made by both man and wife to sale the pig so they can solve the problems at had.

W2: Even as a woman you can suggest to your husband that this child has no clothes, can we sale a pig to buy her clothes.

Mod: How about those without husbands?

*Laughter*

All: We make our own decisions

W7: Those without husbands make their own decision. After accessing their needs they can make a budget and come to a decision to sale a pig or not.

*Laughter*

W5, W6: I make my own decision to sale because if for example I want to be assisted with weeding, I am the one to consider and make a budget on whether to sale or not. During the Christmas time you would find a lot of hired labour ready to assist with weeding chores, this is how we are helped by rearing pigs because even during harvest you can do the same.

Mod: Who feeds the pigs?

All: It is us who are responsible for the feeding.

Mod: The men?

Several women: Men do not usually participate in feeding the pigs, very rarely.

W3: The main job of the men is to construct a kraal.

Mod: What if you are away?

W6: May be at that time, they would.

Mod: How about the children?

W6: The children help but sometimes they would be busy playing therefore not very dependable.

Mod: Does it mean that you only need one person to be in charge of feeding the pigs?

W8: Yes you need only one person to be responsible for the feeding unless the one responsibeis not there, then others can help.

Mod: Why should it be like that? Any reason?

W8: Yes, the reasons are many. Some of which are that we believe that if some people feed the pigs they would be able to produce more piglets, sometimes more males would be littered if some particular individual feed the pigs or sometimes more females. We only change to another person if the desired results are not being achieved. But it is acceptable that if I’m not there those around can feed the pigs.

Mod: What type of feed do we feed our pigs and how many times per day?

W6: We feed them maize bran three times a day.

W3: When the pigs are penned in the morning you let them out, they go and scavenge, and then in the evening you call them to the pen and give them maize bran. So in most cases the pigs are fed once in the evening during the dry season but in the rain season when there is zero grazing that is when they are fed several times. We are scared that they may stray and eat someone’s crops in the fields.

W6 supports the same scenario

Mod: Other what do you say? This time of the year do you let them out to go and feed?

W3: Yes we do, but you have to call them back in the evening so that they get used to being penned.

Mod: You just bring them in for them to get used to being penned or you would rather let them sleep outside?

Several women: In many cases some people do not pen their pigs during this season.

W3: Yes but it is very bad practice to leave the animals roaming outside in the night; if possible they should be in pens.

W6: These days most people keep their animals outside, they do not construct pens for them.

W7: Most pigs are kept outside. Most house holds have no pen for the pigs, but goats are usually kept in door.

W8: Those who care usually keep them in door and let them out side in the morning.

Mod: Feeding them only once?

W8: Maybe two time because when you let them out in the morning you give them some maize bran and in the evening when you call them you give them some maize bran as well. This scenario is when one has pretty of maize bran but they are definitely fed once in the evening.

Mod: If you do not feed them in the morning do you think that they look for their own food?

W6: Yes they look for their own food.

Mod: What type of food do you think they look for?

W5: The proper way of pig rearing is to feed it at least three times a day and maintained in door, but as for us we usually let them out in the morning so during the day they to go and eat those bad foods that we told you before. Then in the evening we call them and give them food only once. This is the way we have been keeping pigs, but we are now ready to be taught the good method of rearing pigs.

W6: Let’s teach one another.

W5: Yes let’s teach each other, we are behind.

W6: The problem with pigs is that they do not get satisfied you would give it food, but then you would shortly find it feeding on rubbish as if you had not fed it.

All the women agree.

Mod: What type of food do you think it will find where it is looking for food?

W6: Faeces offcourse.

Mod: Do you think it is a good thing for pigs to feed on faeces?

All: It is not good.

W6: It is not good because they transmit diseases.

W2: Yes and when they eat the faeces, the faeces go into the meat and then we eat the meat. And sometimes that is why you find worms in children and some suffer from fits because of eating pork.

Mod: What do some of us observe from the pig eating human waste?

W5: This animal is bad when it comes to its eating habit. That is why those who are choosy do not eat the meat. The only good thing is the money you make and also the nice taste and smell of the meat when you slaughter it.

W6: That is why I stopped eating pork, because it eats anything including things that are so dirt.

W7: Despite all these things that have been said there is a lot of money from selling pigs or its meat.

Mod: Please feel free to comment, in you own opinion how many time is pork eaten in the community?

The women are stuck by this question; the moderator asks the question again.

Mod: At what time is pork eaten in large quantities in the community?

W6: When there is an out break of swine fever.

W5: It is also eaten much during the month of October.

W1: Yes in October during the dry season when swine fever strike that is when they eat a lot of pork.

Mod: So it’s only during the swine fever period in October?

W2: Also in December during Christmas time. That’s when a lot of pigs are slaughtered and pork is consumed in large quantity.

Mod: And any one else?

W6: Also when one want to kill a pig and eat some pork to eat in the home and some of the meat you sale.

Mod: W7 would you say something?

W7: Yes, pork is found all the time in the community and you could by some any time even now if we went outside we would find pork being sold.

Mod: During girls initiation ceremony?

All: It’s when you find a lot of pork being eaten in the community.

*Soft laughter*

Mod: Where is the source of these animals which are slaughtered so frequently?

W6: Some come from the surrounding village and some are from our village.

W8: They come from the community from those who rear pigs.

W1: I agree *(by murmuring)*

Mod: If there is pig meat it means every body should see it even those who do not eat it?

All: Yes.

Mod: What do you see?

W7: Fat.

*Laughter*

W7, W6, W3, W1: If it has no fat even us who do not eat will not buy, the trader will go back with his meat.

Mod: Why do you look at the fat?

W1, W5, W6: The fat tells you if the animal was in good health or not and the meat would appear red. This would definitely tell you that the animal was sick.

Mod: What other signs would you look for when you are buying pork?

W7: Some animals have what looks *nsembe* in the flesh; if one notices this then you do not have to buy the meat.

Masese = when they brough a lot of beer….

Nsembe = grained the maize, after sieving, what remains in the sieve, the courser

Gaga = maize bran

W6: If it has this *nsembe* in the flesh you do not need to buy the meat, it should just be thrown away.

Mod: Any more contributions? Why do you not buy meat with *nsembe*, because some say that it is very tasty when you put it in your mouth and feels nice to chew?

I (Dr.Kabemba): They taste nice?

All: It is not good meat

W2: That *nsembe* in the flesh has diseases.

I (Dr. Kabemba): Do people in the villages throw away meat with *nsembe*?

All: Usually the meat is eaten

W5: Lets be honest, I have never heard that some one had slaughtered a pig and found that it had *nsembe* and the meat has been thrown away, most people eat the meat. Even when they notice that the pig has no fat and they suspect that it was sick they still eat the meat.

W2: Some people when they find that the customers are refusing the meat because it has *masese* they dry the meat and feed it to their children.

*Laughter*

I (Dr. Kabemba): Do you dry the meat with *nsembe*?

All the women agree that yes the meat is dried

Mod: Is the meat inspected after it slaughtered?

All: The meat is not inspected.

W6: They just slaughter any how without following any health condition.

Mod: Why do they not have the meat inspected?

W6: It is very far at the Boma. That is where the meat can be inspected.

W5: Like now you have called this meeting which is the first of its kind to discuss pig rearing, because the people in the village are behind in modern methods of pig rearing. There is need to hold these meetings to sensitise the people, tell them what to do when they want to slaughter a pig, who to call so that the meat can be inspected. If these rules can be strengthened and enforced, then the people would have their meat inspected before sale. But as of now people have this freedom to slaughter pigs when they want and where they want. But I am sure that if they were brought to the clinic here as you have done with us and taught the rules about what they can not do and how to slaughter and the rules regarding health, we would be assisted very much, and then there will be a change.

W7: We also need know the person who would be responsible for the inspection so that he can be called in to inspect the meat.

W4: At the Boma the inspector goes round to inspect meat where the slaughter pigs, but here we do not even know who should be doing that.

Mod: At the Boma do the inspectors know those who slaughter pigs?

W4: Yes the people who slaughter pigs are known and they know the inspectors and call them after slaughtering a pig.

Mod: Would we be happy if meat was inspected?

All: Yes.

Mod: Why?

W7, W6: To reduce diseases.

Mod: What diseases?

*Silence*

Mod: So when the meat is inspected then the diseases are reduced?

W2: Pigs usually have worms in the stomach and flesh and they the pigs transmit fits.

Mod: How do you prepare the meat?

W7: For us in the homes we wash the meat, boil it for a long time, and then fry the meat. But we do not know these people who roast the meat on the fire whether the wash the meat or not, because they conduct their business any where that is convenient to them.

W4: We boil the meat so that the germs are destroyed and after the water in the pot is dry, we fry the meat.

Mod: How about you W1?

W1: I follow the same procedure like my friends.

Mod: Why do you add a lot of water to thoroughly cook the meat?

W6: Yes we add a lot of water and some one taught us that boil the meat for thirty minute to kill the germs. But for those who roast, the germs do not die.

Mod: Others, what do you think? What is your comment on roasted meat?

All: In the case of roasted meat the germs do not die.

W8: In case of roasted meat you find that the meat is not evenly roasted, so you find that one side would be roasted while the other side is not. Therefore the germs are not killed.

W3: Roasted meat does not cook well especially pig meat it is just the fat burning on the fire.

W2: Sometime people are in hurry to leave, so the meat would not roast well. Cooked pork usually in funerals, where people are also in a hurry, they do not boil thoroughly and people would fall very sick after eating half cooked pork. Then some one told us that you need to boil pork thoroughly before eating and when we came to the clinic you read the posters, that is how we changed to thoroughly boil pork.

I (Dr Kabemba): don’t the men eat roasted pork?

W1: They eat, that is the meat they enjoy *(laughs)*

All: the men say that is the meat which smells nice

I (Dr Kabemba): they bring the meat then you roast? Yes

*Laughter*

All: The men like the fat part from the neck of the pig.

*More laughter*

Mod: What is then your comment on those who sale roasted meat?

W5: Those selling roasted meat, the rules of how they conduct their business must first be strengthen because you find them roasting at every corner and sometimes they do not even clean the meat like we do in the homes. The sanitation is really not pathetic. People just go there and buy the meat. There is very clear need that this trade should be regulated.

W2: Sometimes they wrap for you even meat which is not properly roasted.

W6: It is sad because when they give you a piece which is not well roasted, you think that is a favour. They have given me extra meat when in reality they are giving you a disease to take home.

Mod: Have you ever noticed *nsembe* in the meat?

All: yes we have seen them.

Mod: When you see them what do you think about?

W1: When we see those *nsembe* we think that the meat has a disease.

Mod: What do you think when you see the *nsembe*?

W6: We think that the meat has a disease.

W3: We think of a lot of things.

Mod: Let us talk about those thing which you think about when you se those grains?

W5: Some times a pig would have very good fat but after slaughter you find that it has *nsembe* in the flesh, so what we were told was that the one who feed the pigs have not “good hands”, so there was need to change the person feeding the pigs and then the condition would change.

Not having good hand = not having green hands, not having the gift to raise those animals

W6: In the past we used to hear that those are brought about by feeding pigs with solids from the local beer, but funny enough these days there is no local brew but these *masese* are still found in the meat.

W7: The moderators question is what would you compare these *masese* you find in the flesh of the pig? We need to look at them very carefully and tell her what they can be compared to.

W2: It looks like inside the flesh there is an insect or something which causes that.

Mod: W3 can say something?

W3: The *nsembe* that do not look well; they are like the disease which we women suffer from.

Mod: What disease that women suffer from that look like that?

*Laughter*

*Silence*

Mod: Those *nsembe* are they the same those women have?

*Laughter*

W3: That is why we said customer would not buy your meat if it has *nsembe* because that is a disease.

*(Soft laughter continues but with a serious edge)*

W3: It is not every woman who has that disease but that is sickness.

R: It is not very clear on the *nsembe* part. Can you explain properly so that we understand?

*Laughter*

I (Dr. Kabemba): You are just laughing can you explain about this disease.

Mod: Please can you explain about the disease you are talking about and compare it to the *nsembe*?

W5: To teach a child you must explain thoroughly and to the point.

W8: Yes there are similar *nsembe* which are found in the vaginal tract all the way to uterus of a woman and sometimes women affected with these have difficulty in conceiving, but when elderly women give them medicine these are usually treated, but sometimes they do not cure.

Mod: Would a person know that she has *nsembe*?

W8: Yes the elderly women are the ones who notice these because if they multiply, they even come out.

W3: What is the name of this disease?

W8: We say the woman has *masale*.

Masale =

*Laughter*

W8: That is the name I know

W7: They call it “Masale”, that is the name they use. *Masale* is the things they use to bewitch a woman who suffers from this condition.

*Laughter*

R: *Masale* is not the same name as *nsembe* in pork?

W7: Yes, *masale* is the same as the *nsembe* in pork flesh.

I (Dr. Kabemba): How prevalent is this condition in women?

W7: Yes it is very common condition among women.

Mod: Do you have pit latrine?

All: yes we have.

Mod: Have you ever eaten meat with *nsembe* in them?

All: Yes we have eaten it before.

*Laughter*

I (Dr. Kabemba): how does it taste in the mouth?

W2: It tastes waterly?

Mod: How about others? How does it feel in the mouth?

Most women: We do not remember because we ate that meat when we were very young.

Mod: W5 how does it feel?

W5: It does not feel okay.

W7: They look like small balls and they would feel crunchy since they have some fluid inside.

Mod: Is it fluid?

W7: Yes it is a fluid.

Mod: Do we all have toilet, how many have toilet?

All: yes *(by lifting up their hands)*

Mod: Do we all use these toilets?

All: Yes.

Mod: The children?

All: Yes.

Mod: And your son in law?

W6: No, the son in law must have his own toilet.

I (Dr. Kabemba): Do the men carry an axe when going to fetch fire wood from the bush?

All: They do

I (Dr. Kabemba): Do they go to fetch fire wood?

All: Yes.

Mod: So meaning that in Chiludzu Village all the people have toilets?

All: Some do not have toilets.

Mod: Those who do not have where do they go?

All: They go into the bush.

W5: That is when the pigs follow them.

*Laughter*

I (Dr. Kabemba): When we ask this question about who have no toilet, you always say it’s other. Who are those?

W7: We can only answer for ourselves.

Mod: Why do you use a toilet?

W6: To prevent diseases.

Mod: So if a person uses a toilet, are they preventing a disease?

All: Yes

Mod: How?

*Silence*

I (Dr. Kabemba): If you want to teach a child you must be explicitly.

W5: After eating we go to the toilet to get rid of the waste matter.

Mod: Any other reason why we go to the toilet?

W2: We need some privacy when we are in the toilet.

W7: If we all use the toilet we avoid pigs from eating the waste from humans.

Mod: Anything else on toilet?

W5: It is very important for each house hold to have a toilet because we prevent diseases like cholera which have become very common. Because if one has no toilet and excretes on the floor and is suffering from cholera, if the fly sits on their faeces then come and sit on the food, then if a person eats food where the fly sit he would suffer from cholera and within a short time the person dies. That is why they brought this ruling that each house hold must have a pit latrine and a pit where to throw all the garbage. But because there are always difficult people, you find that he has two grain stores full but he has no toilet. Where do they go? In the bush and the pigs would eat this human waste and that is the bad part of pig. But in trading pigs are very profitable because they grow ver fast and sale for better price.

Mod: When we talk about hygiene, what come to your mind?

W7: Hygiene means having a toilet, a pit where to throw all the litter from around the house and from the kitchen.

Mod: What do other say?

W6: Hygiene means swiping the house cleaning the kitchen utensils

W3: Let us say when you start building a house, you should build a toilet first, build a bathroom, dig a pit and start digging the foundation and then build you house once the house is finished make a stand where you will dry your plate and pots. Then the kitchen where you prepare the food must be swept early in the morning and you must take out all the kitchen utensils to wash them. When food is prepared it must always be covered, if we want to keep food for absent members of the family we must cover the food and flies should not sit on the food.

Mod: What would you say about personal hygiene?

W6: Taking a bath and washing clothes.

Mod: How many times should a person take a bath?

W7: Here in the village, it is only once, because we have too many house chores.

Mod: Any other suggestions?

W5: To be honest with you madam moderator, here in the village we bath only once because of the type of chores we do. May be this time one may try to bath twice in the morning and evening but this is very rare, the correct answer is once in the evening like the previous speaker had said.

Mod: hygiene of the children what are we supposed to do?

W1: They must be bathed and kept clean, so that they look nice.

Mod: Should we bath the children every day?

All: they should be bathed every day and wash their clothes.

Mod: Whose job is it to care for the children?

All: It is our job.

Mod: What is the role of men in child care?

W7: The men must check whether the children have blankets, reed mats where to sleep and clothes.

Mod: How about washing your hands, when are you supposed to wash your hands and how many times?

All: You must wash your hands many times.

W2: You wash your hands when you come from the toilet, when you want to eat food, when you want to prepare food.

W6: When you come from doing any task you should wash your hands also.

Mod: what if you come from the toilet?

All: You must wash your hands.

I (Dr. Kabemba): Do you really wash your hands?

All: Yes.

I (Dr. Kabemba): How about the children?

*Laughter*

All: We wash them, but it is not easy.

W2: I think the correct position most children do not wash their hands after using the latrine. In most cases children only wash their hands when they are about to eat Nshima.

I (Dr. Kabemba): Do you wash the breast before feeding the child?

W2: No, I do not wash the breast.

Mod: W4 can you make a comment?

W4: It is true that every time you should wash you hands but for the children it is difficult unless you are around because even when eating food you must re-mind the children to wash their hands.

Mod: How about us do we always wash our hands each time?

W4: Yes we always wash our hands before we prepare any food, when we come from the toilet or just coming from doing other chores.

R: How about when eating fresh maize?

W4: Yes, even when we are eating fresh maize. In fact when ever we want to eat anything we should wash our hands.

R: Even when you’re at the field?

W2: Well, there are times when you would wash your hands when you come from the field. You wash your hands but after roasting it you would find that you do not wash your hands, you just pick the maize and start eating.

Mod: Like the way we have come here, we have greeted each other, when eating these biscuits are we going to wash our hands?

All: It is difficult to wash hands, most time we just eat.

W7: When you are in the field and you get a cob of maize you would not stop ploughing and go to look for water to wash your hands you would just eat the maize.

W1: It is not possible that when you pick a mango in the field you would go looking for water to wash your hands most times you just eat the mango.

*Laughter*

Mod: Have you ever seen worms?

All: Yes.

Mod: Where did you see them?

W6: Yes we have seen them when our children pass them in the faeces. Some appear red and some appear white, especially when I was in town.

Mod: Others, where have you seen them?

W7: Sometimes we have them ourselves and some children would pass a lot of worms.

W6: I pulled one worm from my own nostrils.

Mod: Where do you think these worms come from?

W7: From food.

Mod: Which food would be a carrier of worms?

Several women: From cold food.

W7: It is from not properly cooked pork.

Mod: Now you who are saying they come from cold food which cold food, drinks?

W2: From cold *nshima* which is not warmed before serving the children. Some of this nshima has small worms which when ingested by the children start to grow in their stomachs.

Mod: Others, what do you say?

*Silence*

Mod: W5 what is your comment? Are they born with the worms?

W5: I think cabbage would infect the children if it was eaten everyday, but since we all do not have one answer may be you should teach us the correct infection mode.

Mod: Now these people who vomited worms what were they complaining of?

W6: They were complaining of stomach pains.

W3: Their physical appearance even their skin was not looking nice, their stomachs were budging.

Mod: Others, what was the person complaining of?

W5: My child was complaining of the itching in the anus, so when I checked I found some small worms in the anus that is when I decided to take him to the hospital.

Mod: Have you ever seen what is on the pictures that I have just given you? Have you ever seen something like this in someone’s faeces?

W7: We have seen worms but not these other things.

W2: I have seen them in someone’s faeces before, the flat worms.

I (Dr. Kabemba): Where have you seen them?

W2: After the child went to the toilet, I have seen them in faeces.

R: That one, which looks like a tree borer (chisenda). The rest are faeces.

W6: Some have an appearance of a tree fibre.

Mod: Do you think worm diseases are preventable?

All: Yes, it is possible to prevent the disease.

Mod: How would they be prevented?

W7: Yes, if a good sensitisation campaign was done and all the people were taught the preventive measures to take.

Mod: What do the others think?

W5: Yes they are preventable because if you discover that your child has worms and you brought it to the clinic they give you a large pill, which they tell the child to chew and even if it had a lot of worms they would all come out. And when you bring your child to the clinic during the immunisation week the children are given this tablet to chew, that way you mitigate the disease.

Mod: Any more contribution?

W7: The main prevention is if you are taught what actually causes worms and you avoid giving your child food that is a host of worms. In my own opinion that would be the best preventive measure.

Mod: Do the children who suffer from worms get cured?

All: Yes they get cured.

Mod: Have seen any and how were they cured?

W6: If they are brought to the clinic and they are given medicine they get cured. In my own opinion we can mitigate the infection by worms if we administered medicine to them after a very few months, because I think the same food we eat is the host of these worms.

Mod: Do you think it is a frightening thing to have this disease?

W6: No it is just not nice.

Mod: Why is it frightening to have worms in children?

W8: Yes it frightening because children do not grow well.

Mod: Why should you be so frightened?

W3: The fear is that sometimes children vomit these worms, and we are told that if a child chewed the worm he would die.

Mod: W2 what would scare you so much about the worms?

W2: Well you know that these things are in the stomach and if they found that there is nothing to feed on, they would start eating the intestine lining of the child and cause some sole which can be painful to the child.

Mod: Any more additions?

*Silence*

Mod: Do you think that there is any other disease which is more frightening than this?

All: There is.

W5: Yes, take for example cholera. These worms, some one will be alive for sometime but cholera does not. It is just in a few hours and one is dead.

Mod: Which disease does not frighten you?

W7: There is no disease that is not frightening.

W3: The new disease like AIDS and malaria are not frightening.

*All the women laugh*

W3: Because these days they call AIDS malaria.

*Laughter persist*

I (Dr. Kabemba): You are not afraid of AIDS?

W3: Yes they are not afraid of AIDS they (younger person) have changed the name. They say it is Malaria because when you go to the hospital they will give you medicine.

W8 leaves the room because her child is crying.

W7: I’m very scared of AIDS.

Mod: Now in every day life what problems would a person suffering from worms face?

W5: The major problem which I would have if I was suffering from worm infection would be that I would always be sad and if they multiply they would feed on my intestine lining and cause wound which if they are many would kill a person. And you always want to eat because they worms eat your food.

W6: Yes and you share your food with the worms.

W2: Yes and even the headache do not stop.

W1: You always want to eat; it is like you have not eaten.

Several women nod in agree with her.

Mod: We said we knew the *nsembe* in the pig flesh and that we have seen them before?

All women agree.

Mod: When we see them where do we think they come from? Do you think pigs are born with them?

All: No pigs are born with them.

Mod: But where do they come from?

W2: They come from what pigs eat.

Mod: Which food?

W2: Pigs are mostly fed on uncooked bran. So what is contained in uncooked bran is not known.

Mod: Any other ideas?

*Silence; only baby crying sound*

Mod: Where does this *nsembe* come from?

W1: It comes from the food they eat. Because where pigs roam they eat different kinds of foods including faeces.

Mod: Do you think this disease comes from human beings or from other animals?

W6: We think this is a human disease because the pigs eat faecal matter from humans. So even us humans when we eat infected pork we also become sick.

Mod: What do others think? Do you think this disease from livestock only or from humans?

W3: It is a disease for livestock but we do not understand the mode of transmission.

W7: Yes, we think that this is a disease for animals.

Mod: Do you know fits?

All: Yes.

Mod: What would we say on how fits are transmitted?

W5: Fits come, sometimes when a child is sick from malaria and her body temperature goes high, if it is persistently high for a certain period the child will start fitting.

Mod: Any other thoughts?

W2: There are fits which start when the child body temperature is just normal then the child starts to fit. In most case because it is suspected that fits come from pigs, when the medicine man starts to give your child medicine for fits they recommend that the child should not eat pork.

Mod: W1 where do you think it comes from?

W1: I think it is caused when the temperature is too high then you see the child starts to fit.

Mod: What about those elderly ones who suffer from fits where do you think it comes from?

*Silence*

Mod: Is it only the children who suffer from fits?

All: It does affect the old.

W3: It affects both the young and the old.

Mod: Where do you think it comes from? W5?

W5: In my opinion I think it could be family disease because if my father suffered from fits even if I do not suffer from fits myself one of my children would come and suffer from it. That is the way I see it but you can teach us how it comes.

W6: I agree with you.

Mod: how about in your own area, are there any persons who fit? Where do you think this disease comes from?

W7: I heard that from the same meat which we do not cook thoroughly that is where the disease comes from.

W6: Now how about those who do not eat pork but they still suffer from fits? That is what we want to know today.

Several women agree with this thought.

Mod: In your village are there any people who suffer from fits?

W6: Yes.

I (Dr. Kabemba): Could it be mice meat which causes that?

*Laughter in the room*

W6: Do mice also have diseases? *(Laughing)*

Mod: You have people who fit in your village, now how do you think the disease affected them?

W5: Yes this woman is elderly, in our thinking we think may be in her family there were family members in her lineage who were suffering from fits and she would have got it from them. That is my own opinion but may be you can shade more light on this issue.

Mod: Do you think we can prevent this disease, if so how?

All: Yes.

W5: You are educated persons may be you already found the preventive measure, we therefore would be glad to learn from you. With us we may be thinking that you inherit the disease from you parents when in fact not.

Mod: W7 any comment?

W7: yah, we have been told by some other people that it is the meat that you eat that causes this disease but we also note that there are some people who do not eat pork and they suffer from the disease.

W6: That is the more reason we want you to tell us the real cause of the disease.

W2: Some other people only stop eating this meat when they vomit.

Several women: It is not everybody.

Mod: Now we are talking on how we would prevent this disease. And how can we prevent the disease?

All: When you teach us then we would know how to prevent the disease.

W6: The only way we can prevent this is if you taught us what causes the disease then we would know how to prevent it and stop eating that food which carries this disease.

All the women support this line of thought.

Mod: W3?

W3: Like the last speaker has said, we know that fits from high body temperature is preventable, because if a child is brought to the clinic he stops. But the other one you find that if a child starts suffering from this when he is very young then he would continue suffering even in adult hood, some until they die. So some times we wonder whether this is from God or they are just bewitched. So unless you tell us the cause we are blank on how to prevent it.

Mod: Do you think it is frightening to have this disease?

Several women: Yes it is frightening because the disease kills.

W8 joins the group back

Mod: Any other thoughts?

W3: The frightening thing about this disease, take for example if it is a small child who is sick and you leave it with friends and you have gone to draw some water. It may fall on the fire or in the water and die.

Several women agree with her.

Mod: What challenges do you think a person suffering from this disease face in their daily life?

W7: Falling in a fire or water.

W2: Then the seizure attacks the person while on fire or in water, then she would die.

W7: Some times if she falls with the mouth open the person would die because the dirt would go into the mouth and block the breathing pass ways.

Mod: What if the government brought rules that, we need to prevent the fits and the worms and the rule is that we keep all the pigs in enclosures and surround the pen with a fence what problems would you encounter?

W5: That would be a good idea; since the pigs would eat the right food and would not go round scavenging. It also means that even us we would be protected from the diseases which affect pigs and humans; that would be very good.

W7: If the government brought this rule it would be a bit difficult for some of us, especially during the rain season when we do not pound the maize to have some bran to feed the pigs. This in my opinion, it would be the biggest challenge.

W5: You would have to buy the maize bran from the Boma.

W7: There are some people who have no money to buy the bran.

W3: There are some people who follow this rule, but others do not. That is why we have all these problems with diseases.

Mod: If the government brought a rule that all of you should dig pit latrines what challenge would you face?

W3: That is not a problem; the problem is with people who do not want to comply.

Mod: Now if it was suggested that all kinds of meat should be boiled. What problems would you face?

All: That would not be a problem.

Mod: What challenges would you face if a rule was passed that would require that all pork meat should now be inspected before it is sold?

All: That would be a very good rule and should be brought soon, as long as the people are told who is going to inspect the meat and if they can be readily available in the area.

*Laughter*

W3: Like we have already said, there are some people who are difficult. The inspector would condemn the meat and some would refuse to comply.

W7: Some people told us that if you boil the meat for a long time the germs would be killed.

R: That is the correct.

W7: They would take it to those who roast meat, and it would find its way on the market.

W6: The roasted meat is not even properly cooked.

*Laughter*

Mod: What if they propose that all vegetables should be washed properly, including cabbages, rape and other vegetables?

All: It is fine.

W7: Would it include dried pumpkin leaves?

All: there is no problem.

I (Dr. Kabemba): Do you wash vegetables?

All: We wash the vegetables but some vegetable like the pumpkin leaves harden if they are washed.

W6: You wash the vegetables before cutting them.

W7: I do not clean fresh vegetable.

W4: Me too.

W2: I have never heard of that.

Mod: Lets now discus about personal hygiene. If we take care of our bodies what problems would we encounter?

All: There be will be no problem

Mod: We would not complain that why should we take bath and look nice?

W7: No we would not complain.

Mod: What about immunisation for all those with worms. What problems would be encountered?

W6: It would be very good idea and no body would complain.

W3: Let me also contribute. There are some women who do not bring their children for immunisation. If you tell her she would rudely answer you that is it not your child. We are talking of very recent cases.

W7: Some women refused to come to this clinic for immunisation just very recently when we had our last child week.

Mod: What if they said all the pigs in the villages should be immunised. What problems would be encountered?

W7: There would not be a problem.

This problem is supported by several women.

Mod: What if they say you have to pay for the immunisation?

W8: There would be no problem.

W5: Let’s be honest. We have heard just a few minutes ago that some women were refusing to bring their children when the immunisation was free, do you think they would agree to pay even one thousand kwacha to have their pig immunised? No, let us just ask that if possible they do it free considering the poverty level in the villages.

Mod: We have now come to the end of our programme we have been discussing about the goodness of keeping pigs, the badness of pigs, the digging of latrines, worms, fits, we also talked about how we can prevent fits and worms. That we should keep pigs in an enclosure, wash the vegetable that all those with worms should be taken for medical treatment. Do you have any questions or any additional items?

W6: Would those who would come to immunise the pig go round the villages or come to the clinic?

R: Where would want it done?

W6: It is you who would tell us where it can be done.

R: You are the ones, who would choose the place, so where would you choose?

W6: If there was a call, I would not mind where, my personal view is that I would come even here.

W7: How would they know that this pig has worms and should be vaccinated?

Mod: I believe you, who come to the hospital; you know what happens, W7. You come regularly with your small children, even when you go through your medical report, you see what happens.
