# Supplementary material for: Why Latrines Are Not Used: Communities’ Perceptions and Practices Regarding Latrines in a Taenia solium Endemic Rural Area in Eastern Zambia
Source: PLoS Negl Trop Dis. 2015 Mar 4;9(3):e0003570. doi: 10.1371/journal.pntd.0003570 (PMC4352092; doi:10.1371/journal.pntd.0003570)
Supplement: S1 Dataset — (ZIP) [file pntd.0003570.s001.zip › FGD transcriptions_Zambia-2010/Chimphanje_David Mwanza/Chimphanje-Children.doc]

**Title : Focus Group CHILDREN**

**Date :** 31/07/10

**Site :** Kakiwa Rural Health Center

**Village :** CHIMPHANJE	**Location :** Petauke district, Eastern Province, Zambia

**Duration (total time) :	40min	- Start :**	10h30	**- End :** 11h10

**Participants:** 9 children

**Informed consent:** signed and available

**Moderator :** Mr Emmanuel Mwanza	**Recorder :** Dr Andrew Phiri

**Transcription/translation :** David Mwanza

**Abreviations :**

**M : moderator, R : recorder, I: intervener, C : children, W : women, M : men**

**Disposition (from the left of the Moderator):**

**M**

C1

C2

C3

C4

C5

C6

C7

C8

**R**

C9

Gender distribution:

**C2-C3-C4-C5-C9**: females

C1-C6-C7-C8: males

Age composition:

C2-C3-C4: oldest (more than 15 yrs)

C5-C6-C7-C8 (10yrs): youngest

Typology:

C3: dominant (the oldest)

C5: very quiet

C1: quiet

**Comments about participants :**

The Youngest was 9 years old; more were less than 11yrs, the oldest was 15years old. Fidgety and very restless

**Group dynamics :**

Poor level of participation, about 3 were active. Most were restless and unwilling, reluctant to freely participate. The level of interest for most was poor generally. They were nervous, restless on pig keeping practices.

Emotional aspects: Silence, pause on washing of hands, looking at each other; nervous, restless on pig keeping practices; murmuring disapprovingly when one submitted on lack of confinement; reluctant discussing toilets and f. ..(?) uses

Local language used, use of local terms of disease, food stuffs and behaviour

No spontaneous relevant discussions after meeting

**Text:**

*Introduction*:

M: Firstly, want you to know why my name is Emanuel Sani Mwanza, I am one of those who are helping with work at Kakwiya. Like I have introduced myself, you will also introduce yourselves. We will start with this one.

Participants introduce themselves, with guidance from the M

M: Here, I want you to be very free, taking part, answering questions properly so that it will be clear where it will go. Are you getting me?

All: yes.

M: So, be free, do not panic. Now the reason we have called you is that we should talk to each other. The subject at our discussion is about the livelihood of pigs. The first question is what is the goodness, advantage of keeping pigs?

*Discussion*:

C3: During weeding, we people have a problem, they will kill a pig and it helps them as	people come to weed in exchange for meat.

M: Ok! She has explained that it helps during weeding. What else?

C9: Others eat as relish *(smiling)*.

M: Yes, others keep pigs for relish, isn’t it?

All: Yes.

C4: Others sell pigs and get helped. They use the money for grinding.

M: They use the money for grinding. Isn’t it?

All: Yes.

M: You can add? You have said it helps in weeding, grinding, eating as food, what else? Who can add?

A child raises her hand and the M thinks she wants to say something but she declines while laughing, others smile and the M calms the situation.

M: Ok, you have explained on the advantages of keeping pigs, now what are the disadvantages of keeping pigs?

C3: The badness is that pigs play in the mud and dirt. When they come back, they want to play and feed from kitchen utensils and bring diseases. Pigs also eat faeces (they are dirty).

M: Now, when it feeds from the plates, what is wrong with it?

C3: It brings dirty. Diseases come because of that dirty.

M: What else?

C9: The other thing is that when we eat it, we get sick.

M: Yes! She says she gets sick after eating. What else? Boys, you don’t know the badness? Boys, how do you keep those pigs, how do you keep those pigs in our villages? Speak brother, be free, speak

C8: We feed them.

M: Do you make hats where to keep pigs?

C6: No

As others produce sounds of disapproval

M: He has spoken, well… He is saying ‘No’ meaning that pigs sleep outside. Isn’t it?

All: Yes.

M: How do you keep other pigs?

C4: We keep the in an enclosure.

All: Yes.

M: Others, how do you keep pigs?

C3: During the rainy season, we prepare an enclosure. We can take food and give them. In winter, we get food from fields and feed them in their enclosure.

M: You make an enclosure?

C3: Yes.

M: Fine, that how you keep pigs. Who can add now? Now since you keep them in an enclosure, how do you identify your pigs amidst other pigs?

C3: All that is yours is not difficult to identify. Others prick a pig on the ear to create a mark, identity to show that it is ours

M: Now, those pigs that sleep outside are they cared for or not?

C3: They are not cared for.

M: Why?

C9: They do not make enclosures for those pigs.

M: Why don’t people construct enclosures for the pigs?

C3: It depends on the person, others don’t care. Pigs would go to play in the mud and come to play in the bucket of water at home.

M: So, those are the disadvantages of pigs not having enclosures that you have identified?

All: Yes.

M: Of the pigs that you have, how do you when one pig is sick?

C3: You mean when it is sick?

M: How do you know when one is sick?

C3: You know, normally, it will not be active. When the pig is fine, it is very active.

M: You, what do you say? (*Pointing at C7)*

C8: It does not move a lot.

C9: It does not eat.

M: Meaning that when the pig is sick, it does not move. Now, what do you do when the pig is showing those signs? When it is sick?

C3: Sometimes, they just kill it.

M: They kill it?

C3: Yes, because they have seen that there is no life (hope) for that pig, so it is better to kill it.

All: Yes, yes

C8: … Because it can transmit diseases to other pigs.

M: Now, how many times are pigs fed in a day?

C3: Thrice.

M: Three times a day?

C3: Yes.

M: Others, it’s now in the morning, how many times?

All: Two times? Yes.

C1: Three times

M: You feed them three times yourselves? Who else can say something? Is food enough to be feeding pigs three times?

C3: Yes, in the morning, in the afternoon and in the evening as they get into the enclosure.

M: as they enter... fine. (*Agreeing with the speaker)* Who else ... what exactly do you feed pigs?

C3: The peels of maize grains, sometimes we use sun flower.

M: What else?

C3: If it is in the rainy season, you can get the grass.

M: What is the other food for pigs?

C2: They eat faeces.

M: Ok, they eat faeces. What’s wrong about pigs eating faeces? Whose faeces do they eat?

C3: People’s faeces.

M: Now, what’s wrong with eating faeces?

C9: There are diseases in faeces

*Laughter*

C7 stands kneels and asks M to allow him to go out and he is allowed. As he is going out, M resumes talking but the children’s attention is on the C6 going out and they look it no the direction he is going

M: As pigs are moving up and down, what food do they eat?

C3: They eat left over *nshima*. Sometimes, when you clean plates and throw away they refuse. They would come to eat...

nshima = Zambia’s staple food

C8: (comes in) Even fresh maize, after we eat, they come to eat what remains, even maize shaft.

M: What’s wrong with pigs eating what they find as they are moving about?

C3: They eat anything (bad and good), it is better if you give them food because you select what is good.

M: What else do they eat?

C8: They eat some parts of sugar cane (sugar cane peeling)

M: Who can add?

*Silence*

M: Who takes pigs into an enclosure?

C3, C4: The owners.

C3 continues explaining

C3: The parents ask children to take pigs into an enclosure but when parents are not around, children still do I on their own.

M: OK! What about others, how do they keep pigs?

C8: Children are the ones who take pigs into an enclosure.

M: Who else?

*Silence*

M: Now when there is need to kill a pig for relish, who has the authority to kill a pig?

C4: Parents

M: It is parents?

C4: Yes.

M: So, other people can authorise the killing?

C8: They do not allow…

C3: … They say that children and the rest of the people at home are just helpers, so they cannot authorise.

M: So, you do not have the authority?

C3: No, even if I want to have a pig killed, if the parents have not given permission, then it will not happen, unless they say so.

The child who had gone out opens the door as he is coming in. The attention of the children goes to the opening door.

M: Now, after you have killed the pig, how do you prepare it?

*C5 points to the door and makes some gestures*

C9: We add tomato so that the meat (pork) is delicious.

M: What else?

C3: There are some pigs that are fat, so we do not add cooking oil, we just add tomato and onion since it is already fat.

M: There are no people who roast pork?

C3: They (people) eat roasted pork?

M: Yes, now how is the preparation for roasted pork.

C3: Just add salt, that’s so.

M: Between roasted and boiled pork, which one is common?

C3, C4: Boiled, boiled is common.

C3: They (people) cut it into pieces, wash it, put it in the pot, add salt and put it on the fire. When it simmers, it is fried, add tomatoes and onions, then it’s ready, that is so.

M: During which season do some people mostly eat pork?

C3: The period between April and October.

M: Which other period?

C9: And in the rainy season, pigs are sometimes killed to help in weeding, so some kill to make people fasten weeding as they want pork.

M: When else?

C4: Also during Christmas. Pigs are killed and we eat pork with *nshima*.

M: Fine, fine, when else?

*Silence*

M: Have you heard of the *nsembe* about pigs?

Nsembe = Stony stuff

Some: Yes.

M: What are they?

C9: They are normally white, they are found on the pigs, sometimes when you are cutting the pig, you can see them.

M: Pass that picture so that everyone can see. What do you think those stony stuff are?

Children start passing the picture from one to another

C9: They are diseases.

M: Have you seen the picture, what are those meatish stuff?

C3: *Nsembe* (stony stuff)

M: Do you think you have seen these before? Have you seen a pig after being killed?

All: Yes.

M: You said that those stony elements are diseases. Do you think that those diseases can have an impact on our lives?

C3: Yes

*Silence*

This silence follows despite encouragement from M to speak.

M: In the village, do you have toilets nearby?

C3: Yes.

M: What is the use of a toilet?

C4: We smear the ground, we paint it

It appears the child has misunderstood the question

M: Ok, who else can tell us the uses of the toilet? Why do we construct a toilet?

C3: When we suffer from diarrhoea, we use the toilet.

M: In the village, do we use toilets?

C3: Yes.

M: Do we have toilets in our village?

C9: Toilets are there. While some have, others do not.

M: Now, since some have and others do not, what do those who don’t have do when they want to relieve (urinate or defecate)?

C3 smiles and then M repeats the question

C7: They g to the bush.

M: Others, what do you say?

C3: For example, if you have diarrhoea, it is very problematic if you don’t have a toilet at home.

M: Now, let’s suppose that you don’t have diarrhoea, what’s the problem?

C7: There is dirty, because it is not only diarrhoea.

M: What time do you wash your hands after toilet?

C3: Just after using the toilet.

M: Why do you wash your hands?

C9: Sometimes, whilst in the toilet, you contract some dirty.

M: Now, if you don’t wash your hands, what’s wrong about it?

C6: Some people do not wash hands, they eat without washing.

M: What’s wrong with that?

C8: They can suffer from cholera.

M: What else? ... What about you, do you wash your hands after toilet?

All: Yes.

M: What kind of water do you use to wash your hands?

C8: Water from the pond.

M: Do you only wash when eating *nshima* or even with other foods?

C3: We wash when eating any food.

M: Is that manageable?

C3: Yes but sometimes it is difficulty.

M: So, sometimes it is not possible.

C3: Yes.

M: Have you ever seen worms?

Some: Yes.

M: Have you?

C4: No, I haven’t.

C3: I have seen them before.

M passes picture for the children to see

M: What do you see?

*Silence*

M: Are there some people who have worms in your village?

C3: Yes.

M: What are the disadvantages of having worms?

C8: Eating soil.

C3: Eating soil without washing.

M: You mean you can get worms by eating soil and eating dirty foods?

C3: Yes.

M: Others, how do you identify someone with worms?

C3: Others can easily get satisfied with food, others take long to get satisfied and others have small stomachs (tummies).

M: What else?

C9: They have a pot belly.

M: Boys, what are the symptoms?

C5: Others will cry because of pain in the stomach.

M: Others?

C6: Others will have dysentery.

M: Are these diseases fatal?

C9: Yes, they can kill someone.

C3: At the clinic, they should get medicine to prevent diseases.

M: Are there worse diseases than these?

C3: Others have diseases where they diarrhoea blood.

M: Have you heard about epilepsy?

C3: Yes.

M: What do you think is the source of epilepsy?

C3: They would be sited around the fire, suddenly, they will start fitting, sometimes, there would be water boiling on fire, so when they start fitting, the water would fall on them and they get burnt.

M: Others, what do you say?

C3: Others fall.

M: Are there medicines for this disease?

C9: No, epilepsy has no cure.

M: So, people with epilepsy will have it forever?

C3: Yes.

M: What is the goodness of caring for vegetables?

C3: We need to wash before cooking.

M: What are the dangers of not washing vegetables?

C3: There are germs.

M: What can germs do?

C3 and C9 start speaking at the same time but C3 stops and lets C9 to continue

C9: The germs can make someone sick.

M: Let’s suppose, you have a garden at home, a child defecates, flies get on the faeces and they go to sit on the vegetables. Is there anything bad with that?

C3, C9: There is something bad.

M: What is it?

C9: The vegetables will get dirty, that’s why it’s important to wash vegetables before boiling them.

M: Yes, boys, speak out, why washing?

C7: Some people will get vegetables, chop them and cook.

M: Would it be difficulty to say that all people should be washing vegetables before cooking?

C3, C4, C9: No, it wouldn’t be difficulty.

M: What if there is a rule that from now on, everyone will be using the toilet and no one will be allowed to go in the bush. Would you manage?

C3: Yes, we can manage (as she smiles).

M: With us who are here, do we use toilets?

All: Yes.

M: What about bathing?

C3: If we do not bath, the openings on the skin get closed, and many may not be feeling well.

M: Others, the goodness of bathing?

C3: Lack of bathing brings diseases.

M: What is the badness of bathing?

*Silence*

M: Would you be allowed to prepare food without bathing?

C3: No.

M: Why shouldn’t you be allowed?

C3: Because of not bathing.

C9: For fear of diseases.

M: What do you say boys? When you maintain cleanliness, would you be preventing?

*Silence*

Children show signs of discomfort suggesting that they are tired

M: At this point I want to thank you for accepting to take part in this study. We have talked about toilets, the goodness of keeping pigs. We have also talked about the disadvantages of keeping pigs. We also talked about having enclosures where to keep pigs. We talked about the advantage of using toilets and the disadvantages of going to the bush.

Now, these pigs would be happy if they were immunised?

C3: Yes.

M: What would be the advantage of immunisation?

C3: So that they don’t die/ to prevent death.

M: Would you be happy if people would be coming to treat pigs?

All: Yes.

M: Well, we have discussed these things. About vegetables, how to deal with vegetables so that you do not contract diseases, how to feed pigs and the disadvantages of those pigs that sleep outside any enclosure.

At this time, we thank you vey much. Maybe you have any questions?

*A bit of silence*

C3: Will you be coming to immunise our pigs?

M: I have heard that question, but we will also come and discuss on that matter with you. Have you gotten me?

All: Yes.

M: Any other question?

*Silence*

M: We thank you once more. We have discussed so many issues about pigs. Thank you.

*Silence*

M stands and goes outside.

M: So, we should be washing our hands and using a toilet, OK?

All: Yes.
