# Supplementary material for: Why Latrines Are Not Used: Communities’ Perceptions and Practices Regarding Latrines in a Taenia solium Endemic Rural Area in Eastern Zambia
Source: PLoS Negl Trop Dis. 2015 Mar 4;9(3):e0003570. doi: 10.1371/journal.pntd.0003570 (PMC4352092; doi:10.1371/journal.pntd.0003570)
Supplement: S1 Dataset — (ZIP) [file pntd.0003570.s001.zip › FGD transcriptions_Zambia-2010/Chimphanje_David Mwanza/Chimphanje-Men.doc]

**Title : Focus Group MEN**

**Date :** 31/07/10

**Site :** Kakiwa Rural Health Center

**Village :** CHIMPHANJE	**Location :** Petauke district, Eastern Province, Zambia

**Duration (total time) :	54min	- Start :**	13h33	**- End :** 14h27

**Participants:** 8 men

**Informed consent:** signed and available

**Moderator :** Mr Emmanuel Mwanza	**Recorder :** Dr Andrew Phiri

**Transcription/translation :** David Mwanza

**Abreviations :**

**Mod : moderator, R : recorder, I: intervener, C : children, W : women, M : men**

**Disposition (from the left of the Moderator):**

**M**

M1

M2

M3

M4

M5

M6

**R**

M7

M8

Age composition:

M2: the oldest

M5: old

Typology:

M4-M8: shy

M1: Deacon (religious)

**Comments about participants :**

**Group dynamics :**

**Text:**

*Introduction:*

R: If you are not happy, you can go outside and never come back. No one will ask you. Any questions?

M1: I ask that we pray first.

R: Yes, it’s fine.

M1 prays for concentration and for success of the study, safe journey for those coming from distant places.

Mod: Firstly, we should be free. The first thing is that we should know each other. Let’s speak loud enough. My name as you know, my name is Emmanuel Sani Mwanza. I am a volunteer here at Kakwiya.

Participants introduce themselves.

Mod: Let’s be free. The title of discussion is on pigs. We want to see how we live with pigs and all that may follow. Firstly, I want to ask, what is the advantage of rearing pigs?

M7: To pay for school fees for our children and also during funerals.

Mod: Any other one?

M2: Here in the villages we are faced with all sorts of problems and so pigs help us in problems, like when a child has passes exams (for school fees)

Mod: We have looked at the advantages of pigs but what are the disadvantages of pigs?

M6: They bring diseases to people. Sometimes they develop *nsembe*.

Mod: Who can add?

M7: The badness is that when you go to the bush, pigs will follow you and as you defecate, they come behind and they eat at your anal area. That’s very bad.

M5: The other thing is that pigs eat faeces, and when they come back they want to feed from the kitchen utensils. They bring diseases to us humans.

Mod: Who can add?

M1: Pigs suffer from epilepsy, so it’s very difficult to cure pigs.

Mod: What are the benefits of rearing pigs?

M4: Pigs help us in many ways when we have problems we sell the pigs and that help a lot.

Mod: Others?

M3: There are people who only keep pigs. Pigs help for cultivating and weeding at the fleads.

Pigs help for weeding = you hire people to come and weed in exchange for a pig or pork meat.

Mod: What are the threats of rearing pigs?

M3: Firstly, say am sick with a diarrhoeal disease and I go to the bush, the pigs will eat the faeces and come to eat from our pots and when these pots are not properly cleaned we eat from them , so they threaten our lives.

Mod: Other threats?

M7: Sometimes we slaughter the pigs and some pigs have cysts that can bring diseases to us.

Mod: How do we keep our pigs? Do we keep them in houses or we just leave them freely roaming, how do we generally keep them in the villages?

M6: Other pigs are kept in enclosures while others roam freely and that’s why they are exposed to bad things and eat bad things so if they are kept in a fence maybe that can be better

Mod: Why don’t we build enclosures? Why don’t we have enclosures for the pigs to be freely roaming?

M1: The major reason is that we depend on the bush, here in villages we are surrounded by bush and we think we are free and can just go the bush and defaecate for the pigs

Mod: When you leave for the pigs what do they do?

M1: So that pigs can be eating without restrictions.

Mod: Pigs should be eating without restrictions?

M1: That’s our thinking here in the villages. We think because we have a lot of bush around us and we think that keeping pigs in enclosures then we are punishing them and we don’t know that pigs also need to be in fences.

Mod: Others about the way pigs are kept?

M4: The other reason is inadequate feed. We fear that if we enclose pigs, they may die. So, it’s better to allow them to be free so that they can be free and eat anything they find.

Mod: What are the problems associated to pigs roaming freely? Have you seen anything wrong with that? What other problems are associated with free roaming pigs?

M2: Those pigs which roam anyhow, sometimes, you just find that it has died on its own since it’s not housed.

Mod: Others?

M7: They also destroy other people’s crops in the field.

Mod: Since pigs roam freely, how do you identify your pigs? Since you just let them go and they mix with other pigs, how do you get to identify your pigs from amongst a lot of others?

M8: We Know, we make notches into the ear to mark them so I can identify them.

Mod: What if other people also give the same identification mark?

M8: I can still identify them. If pigs are mine, I cannot fail to identify my own pigs. When it’s feeding time, even when I have ten I would know that they are mine because they will come to feed.

Mod: Aha! (*Pointing to M1 who had his hand raised*).

M1: Even when you give marks, you do it in such a way that they all look different and you would know that they are yours, even when you make ear notches you put them on one side so you know your pigs.

Mod: Others? How do you know your pigs since they not enclosed?

M7: Sometimes we cut the tail to leave a short one.

Mod: Don’t you think there is a problem there since the tail could be useful to the pig? The tail is not useful to the pig?

M7: You don’t need to cut the whole tail. You just do it a bit.

Mod: Bit by bit?

M7: Just a bit and you will be able to know that these are your pigs.

M5: You will know that they are yours like that.

Mod: Others? (*Pointing at M6*)

M6: For me, I mark the letter ‘W’ on my pigs. That’s how I identify my pigs because I am the only one who marks the pigs that way.

Mod: Aha, (*Pointing at M2 who had his hand raised*).

M2: When you build an enclosure, it becomes the surest way of identifying your pigs because if you depend on marks like ear notches, people can kill your pig and burn the fur, how else do you identify them? We just need to build enclosures and keep them in side, and then one would know that they are his pigs not when they are kept outside, one wouldn’t know.

Mod: Any other person?

*Silence*

Mod: Are there relatives who help caring for your pigs? If you have pigs is there any relative that helps looking after the pigs?

M7: There is no one; it’s just your self.

M3: Sometimes you do not have enough food so relatives or friends can be bringing the feed like maize bran and that when the pig has piglets I will give them a one.

Mod: Relatives?

M3: Yes.

Mod: Anyone who can add on the keeping of pigs?

M6: Yes, sometimes someone from another village can inform you that they want to come and buy a pig from you. They would be bringing some maize bran to feed the pigs so that they are healthy.

Mod: We have discussed that pigs are sometimes housed and may times freely roaming, how do you know that the pig is sick? Maybe a pig is not looking very well, how do you know that it is sick?

M6: You would know, when it comes to feed it will look very different from the last time it came, it will look weak unlike the last time when it was very active, and it will be walking slowly so you will know that it’s not ok. That’s how we know.

Mod: Others? (*Pointing at M7 with a hand raised*)

M7: Not eating, a pig is an animal that never refuses feed so when you give it feed and it’s not eating then you know that it’s sick.

Mod: Not eating right? Others?

M5: Sometimes, it is the tail. The tail will be lowered; sometimes it will lower its head and face down and remain still for a long time and you will know that definitely it’s not well

*They all laugh*

Mod: Others? How do you know a pig is not well? Who can add?

M3: Sometimes, when you are calling the pig to come and eat but it is not coming and maybe your children tell you that it’s somewhere then you know that it is sick. All days when you call it comes to eat so how come it’s not coming? And you know that your pig is sick.

Mod: In a household that keeps pigs, who has the role of feeding the pig? How do you share the role of feeding the pigs?

M6: Mainly, that role is by the woman/ wife. But when the wife is sick, that is when we help.

Mod: Others, who feeds the pigs?

M1: Sometimes, it is the children.

Mod: Children?

M1: Yes

Mod: So you have said that the pigs are fed by the wife or by themselves while others it’s the children, how many times do you feed the pigs?

*Silence*

Mod: How many times do we feed the pigs in a day?

M6: Others, it is in the morning, others it is in the evenings when the pigs come back from scavenging so that it eats and is satisfied.

Mod: Others?

M3: Others especially the ones that don’t have fences just feed it when it when appears. So if it appears once that’s when they feed them

M2: Some pigs are not fed at all. Some people just claim ownership of the pigs when they want to sell them even when they don’t feed them. They would maybe last seen it when it was piglet and only remember it when it’s already grown and they want to sell. Most are alike that.

Mod: You have said that some pigs are not fed, what would be the effect of not feeding the pigs?

M1: What’s needed is making fences, and putting them in the fence and feeding them.

Mod: How about those people, who just claim ownership even when they feed them, what would be the effect of not feeding them?

M3: They will get lost, all of them.

Mod: They’ll get lost?

M3: Generally, a pig will always go where it is fed so those that are not fed will start going to where they are fed and the person feeding it may end up possessing that pig.M1: When a pig is fed lest say once or twice or even three times, it will rarely go out roaming, it will be always be at home

Mod: Is it possible to feed thrice a day in our villages?

M2: Yes, it is possible.

Mod: It’s possible?

M2: Yes, some people manage to feed them three times while others feed once, others feed once in the morning and others once in the evening like that, but others mange to feed them in the morning, at lunch time *(12 hours)* and also in the evening. It is these pigs that are fed well that will not be roaming anyhow. Even when you go to the field, they will just be at home sleeping waiting to be fed. But those that are not fed you really have to call out for them to come and feed.

M1: *(Interrupts),* even the way you call them, you have to make a loud noise for the pig (that is not fed) to come. As for me, I have a pig and I feed it three times a day. It is always at home, can even come very close to where you are sitting and never goes very far from home.

Mod: If you don’t feed a pig, what would follow? What will the pig eat to survive?

M7: Human stool.

Mod: Human stool, Is that true?

*All agree.* It’s true.

Mod: What is bad about a pig eating human stool?

*Silence*

Mod: The disadvantage of pigs eating human stool, is there anything bad about that?

M4: Since it eats stool from different people, it can contract diseases and die.

M7: The bad thing is that when it eats our faeces, it then goes to eat from our cooking utensils and that’s a bad thing. Mod: Ok. In a household, who has the authority over what should be done on pigs? Who is the decision maker?

M2: It is the man.

Mod: It’s the man? Others?

M6: It is the man.

Mod: It’s the man, and you brother? (*Pointing at M7*)

M7: It’s me the man.

M1: A man.

Mod: How about children, since they take the role of closing up the pigs, do they have any authority to say you need to slaughter a pig?

M7: No, it is the man who tells the wife that we need meat so we should kill a pig. A child cannot decide.

Mod: What about the wife?

M8: No, she doesn’t have the authority. It is only a man that has authority. A wife fears the husband.

Mod: The wife fears the husband?

M8: Yes.

M1: When you are married, it means you are one. So the wife can suggest but the husband makes the final decision.

Mod: Ok, she becomes free with the husband?

M1: Yes.

Mod: When do we usually eat pork in a year?

M1: At weddings

M7: After we have harvested, we eat pork a lot, especially the fried one.

Mod: *Shokas*?

*Everyone laughs*

Mod: Any other time, others?

M8: When there is a football match.

Mod: You call friends?

M8: Yes, and enjoy the pork.

Mod: Enjoy the pork?

M8: Yes.

*All laugh*

M6: During church choir practice and religious camp meetings, that’s when we eat pork

Mod: Aha, aha *(pointing at M1 with a hand raised)*

M1: And in public gatherings, there is a lot of pork.

Mod: Do you pay any particular attention when a pig is slaughtered? Do you pay any particular attention how it is slaughtered?

M8: (*Whilst laughing*), Yes we pay attention.

Mod: What is it that you pay particular attention to?

M8: That is just relish.

M7: I care about the meat from the neck area.

Mod: The neck area?

M7: It is delicious.

*Laughter*

Mod: It shouldn’t be tempered with?

M2 and M8 together: No.

M1: When slaughtering a pig, it’s important that it is adequately flamed to become brown so that it looks good and clean.

*Some people laugh while others smile*

Mod: So that’s what you pay particular attention to, right? When the pig is slaughtered, is the meat inspected?

M2: No, it is not inspected.

M5: Never! *(Interrupting M2)*

Mod: Why is it not tested ... Yes my brother? (*Pointing at M6*)

M6: I have the same question because that is how we contract diseases. The pig is not inspected or treated and it’s just slaughtered even when it is has diseases we just eat and that’s why we have disease.

Mod: What about those people who move door to door selling pork? Do you take time to check the worth of the meat? Is there anything you check out for in the meat?

M1: Yes, we do. Many times, especially for those that move door to door, they just get a pig that was found dead, they just clean it and start selling. That meat usually appears redder than normal and you can easily tell. The appearance can tell that it died on its own.

M8: If it has patches of hair all over the carcass due to inadequate flaming to remove the hair, you know that it is not good.

Mod: The meat is not ok?

M8: Yes.

Mod: When a pig dies on is its own, do we still eat? What do we do with a pig that has died on its own in our villages?

M7: When a pig dies on its own, some of us don’t eat some eat depending on how the meat appears because sometimes the pig is sick for just two days and dies, such a pig is eaten. But one that has been sick for some time and loses weight is not eaten, it is just thrown away.

Mod: I saw a hand raised, M4?

M4: Yes I had raised my hand.

Mod: Meat that is not looking very good, looks diseases, why is that even when it is thrown away some people go and get it?

M6: People eat that kind of meat because of the great craving for meat.

*Men smile, others laugh*

Mod: Would you be happy if this same meat is inspected before eating in our villages?

All: Yes, we would be happy.

*All men nod their heads and smile*.

Mod: Very good. Have we ever heard of *masese* or maybe *nsembe* in pigs?

M1: Yes, we have. That residue, masse, from beer brewing, if you feed it to the pigs they get the *nsembe*.

Mod: What are you seeing?

Mod passes a picture of meat laden with cysts for the men to see: Have you seen this picture? What can you see in the picture?

All: *Nsembe*

Mod: That’s why am asking that what particular attention do you pay when meat is brought for you to buy? Is that meat ok or it’s not good?

All: Yes we do.

Mod: What is that you see on the picture?

All: *Nsembe*.

Mod: So if someone is going round on bicycle selling the meat in the village, do we check for these?

M7: Yes, actually, it is the first thing we check for. When the one selling brings the meat, we check. Like the Mod has said that maybe the meat looks red, but when it has nsembe you would easily see it, that the meat has *nsembe*.

Mod: M1?

M1: Sometimes, before a pig is even slaughtered, you can see them. Those people that buy pigs to slaughter, before they even buy they check in the mouth of the pig and if it has nsembe they see them, in a live pig.

Mod shows a picture of a pig’s tongue with cysts: Have you seen this picture? What can you see there? Is the pig’s mouth open or not?

M1, M2 and M3: Its mouth is open.

Mod: Why do you think its mouth has been opened?

M1, M2 and M3: So that they see cysts.

M7: *Nsembe* are seen under the pigs tongue and eyes. You reflect the eyelid and you see the *nsembe*.

Mod: M8?

M8: *(mumbling)* This is the *nsembe*.

Mod: All the meat in the village is checked to make sure it does not have the nsembe, is that so?

All: Yes

Mod: What would be the biggest threat when you find pork meat with cysts?

*Silence*

Mod: What threat would be there if you found meat with nsembe, threat to someone’s health?

M1: Diseases.

Yes: M2?

M2 is silent.

Mod: The threat to people’s health is associated with meat that has nsembe?

M2: You just stop buying the meat.

Mod: Stop buying the meat?

M8 :*( raising hand)* Do not eat the meat.

M5: Just take the meat and throw it away in the toilet...

Mod: Ok.

M5: ...Because the *nsembe* is bad, they have water inside them.

M6: Another thing is that you just throw away the meat since it is infected with a disease and we can’t eat meat that is infected.

Mod: Anyone to add on? You have seen meat with which is infected, who can add on what you do with that meat?

M7: The best thing is to throw it away. If you see it either before you slaughter or after, the best is you dig a hole and burry it.

Mod: Do we know worms?

All: Yes.

Mod: How about the disease of fits (epilepsy)? Do we know fits?

All: Yes.

Mod: Is there epilepsy in our villages?

All: Yes there is

Mod: How does epilepsy come about?

*Silence*

Mod: This person is fitting, another one is fitting; this disease, how does it come about?

*Silence*

Mod: This disease of fitting let us all contribute on how this disease comes about.

M3: Sometimes, I hear that, when fever gets severe, body temperature goes very high; a child starts fitting since the temperature has gone beyond the normal.

M8: Some people say, it depends on the time of the month.

Mod: M7?

M7: Like M3 has said, it is high body temperature.

Mod: The threat that is associated with epilepsy, and also this disease, where does it come from most of the time? How many ways can you list on how this disease comes about? Where does it come from?

M7: I don’t anything on where this disease comes from.

M6: For me, I know that it is malaria. When someone gets malaria with a high fever, they start fitting but when they are treated even the fitting stops.

Mod: Anyone else? Maybe someone does some black magic and a person starts fitting, does this happen?

All: *(Amid laughter)* Yes, it happens.

Mod: So, Isn’t that way in which someone can get epilepsy?

All: Yes, it is a way.

Mod: Are there other ways that epilepsy can come about?

M7: Thieving.

Mod: Thieving, ok.

Mod: Is it possible to find the cure for epilepsy?

*Silence*

Mod: Is there medicine that can cure epilepsy?

*Silence*

M7: We can find it from the African doctors (traditional healers).

Mod: We can find it from traditional healers?

M7: Yes

Mod: OK, M7 says we can find the medicine from the African doctor.

R: Do we have people in Chimphanje village that suffer from epilepsy?

Mod: In the village of Chimpange, are there people that fit?

M4 and M6: Yes, there are there.

Mod: Do they fit on a daily basis or they fit during specific times?

M4 and M8: They fit at specific times.

Mod: Are there diseases that are more severe as compared to epilepsy?

*Silence*

Mod: Other diseases that we think are more severe than epilepsy in these villages where we live?

M6: A disease that would be more severe would be malaria. This is a very bad disease that may even affect your neck and back when there is no treatment. And this is bad for someone’s health.

Mod: And one can die?

M6: Yes one can die.

Mod: Any other to add on?

*Silence*

Mod: Have we ever seen worms in our villages...worms in stool, have we seen them, or do we know worms?

M7: mmmmm! *(Signifying yes)*

Mod: How do they look like?

M8: It looks like a snake, a long thing. It moves in both directions, forward and backwards.

Mod: What makes you able to see the worms, where do they come from?

M7: They come from the abdomen.

Mod: They come from someone’s abdomen, ok.

Mod circulates pictures of stool with proglottids of a tapeworm for the participants to see.

Mod: This is how they look like in someone’s stool. So if someone has these worms, how do they get to acquire these worms in their abdomen?

*Silence*

Mod: Have you seen the worm in that picture?

Most: Yes

Mod: Have you seen something that looks like that in human stool?

M3: For me, I have never seen them.

Mod: You haven’t seen them?

M3: Yes

Mod: Have you seen something like that in the pictures?

Participants exchange the pictures to have a closer look.

Mod: Have you seen a worm that looks like that in human stool in the village?

M7: No.

R: They look like snake skin.

Mod: They look like the skin of a snake.

All: No, no, we haven’t

Mod: How do we know that this person has worms?

M6: Abnormal pain.

M3: Eating at short intervals.

M4: Pot belly.

M8: Sometimes, worms produce sounds

*Laughter*

Mod: Any other?

M5: A pot belly.

Mod: Can a patient be energetic?

All: No.

Mod: Can we find cure for worms?

M8: Yes, they are found at the clinic.

Mod: What about herbs?

M6: I have never heard about that.

Mod: What about the benefits of toilets?

M7: When you have diarrhoea, it is quicker to access the toilet.

M3: During cholera, toilets help.

Mod: What about when you receive visitors?

M3: When a visitor comes, he/she will ask where the toilet is. If you have, you will point at the toilet and it is embarrassing if you don’t have.

Mod: What are the disadvantages of defecating in the bush?

M3: As you defecate, people may see you doing the same. It is embarrassing.

M2: You can even meet with your in laws; it is embarrassing.

M8: You may want to go to the toilet even at night, so if you don’t have, it’s bad.

Mod: What are the benefits of washing hands and when do we do that?

M6: After defecating we need to wash hands to prevent diseases.

Mod: Do our children and we wash our hands strictly after toilet?

M6: No boss, we don’t follow. If we are working, we just continue with our work after coming back from the toilet.

The toilet here means bush.

M1: This is followed if an adult is around. Children normally don’t wash.

M3: We do not follow strictly.

Mod: Why don’t we follow?

M5: Lack of rational thinking.

M7: Ignorance of the fact that we may have germs that may give us diseases.

Mod: What are the benefits of washing hands?

M1: To prevent oneself from diseases.

Mod: Is it possible that vegetables can have diseases?

M1: Yes, more especially in the rainy season. Women should be mindful.

Mod: Let’s suppose that we have a garden at our home. As pigs are roaming, they will end up in the garden. Now, if we do not wash those vegetables when we want to cook, what do you think is the problem with that?

M1: It’s ignorance; it’s dangerous. We do not wash and it’s not good.

R: Would it be a problem if we are given a rule that we should wash vegetables before cooking?

All: There is no problem.

M1: There is no problem because we protect ourselves from diseases

Others agree with him

M7: That’s cleanliness and no one can hate cleanliness.

M3: Good looking.

Mod: Can we be happy if there were vaccines for our pigs or livestock in general.

All: Yes, very much.

M3: We would be very happy.

Mod: Would be ok, if our meat was to be tested each time before killing or eating?

All: It would be very good.

Mod: What about having enclosures for our pigs? Would it be good or bad?

All: It would be very good.

Mod: What are the benefits of having enclosures for pigs?

M1: It is very useful in many things.

Mod: Others?

M7: It would be helpful because pigs would not be eating anything. That would be good.

Mod: I think we have discussed, firstly, the advantages and disadvantages of rearing pigs, how to care for pigs, about the benefits of having toilets and the disadvantages of not having toilets. We have also talked about the dangers of roaming pigs, the dangers of cysts. Also about our cleanliness and how to mind our vegetables, if you have questions, you can ask.

M3: Out of all these things we have discussed, are we going to get any help in the future?

Mod: Well, we will have another meeting in the future to discuss the way forward.

M8: It is about the question we asked at first, can we now have the chance to open and watch that *(pointing somewhere to the machine not visible)*

Mod: Ok, they will come to explain since you have the right to see.
