# Supplementary material for: Why Latrines Are Not Used: Communities’ Perceptions and Practices Regarding Latrines in a Taenia solium Endemic Rural Area in Eastern Zambia
Source: PLoS Negl Trop Dis. 2015 Mar 4;9(3):e0003570. doi: 10.1371/journal.pntd.0003570 (PMC4352092; doi:10.1371/journal.pntd.0003570)
Supplement: S1 Dataset — (ZIP) [file pntd.0003570.s001.zip › FGD transcriptions_Zambia-2010/Chimphanje_David Mwanza/Chimphanje-Women.doc]

**Title : Focus Group WOMEN**

**Date :** 31/07/10

**Site :** Kakiwa Rural Health Center

**Village :** CHIMPHANJE	**Location :** Petauke district, Eastern Province, Zambia

**Duration (total time) :**	51min	**- Start :**	12h00	**- End :** 12h51

**Participants:** 8 men

**Informed consent:** signed and available

**Moderator :** Ms Ruth Chiwa	**Recorder :** Dr Andrew Phiri

**Transcription/translation :** David Mwanza

**Abreviations :**

**Mod : moderator, R : recorder, I: intervener, C : children, W : women, M : men**

**Disposition (from the left of the Moderator):**

**M**

W1

**R**

W2

W3

W4

W5

W6

W7

W8

Age composition:

W2-W6: oldest

W1: the youngest

All the rest belong to the middle age category

Typology:

W8: shy

W5: very dominant

W6: expert, dominant

**Comments about participants :**

W8: left her baby outside, worried. Four women don’t keep pigs.

**Group dynamics :**

**Text:**

*Introduction:*

M: Firstly, I will ask you in advance that you be free, there is nothing scary and you do not need to be afraid. I want to welcome you, you need to be free, there is wrong or right answer in our discussion and nothing difficulty. Ok?

All: Yes

M: Before we get into details, we need to know each other. I will start by introducing my self even if you already know me. I am Ruth. OK!

All: Yes

All participants introduce themselves

M: I believe you know these people *(speaking about those not in the picture)*

All: Yes

*Discussion*:

M: Ok, the major discussion is on pigs. We all know pigs. Do you think it’s necessary to keep pigs?

All: Yes, they are helpful *(while others are smiling)*

M: How?

W1: They are helpful during funerals and when we want to send a child to school, we sell a pig/pigs to raise some money.

W5: Also when we are hungry, we kill a pig and we get helped with children.

R stands and goes away

M: Who can add? What is the advantage of rearing pigs?

W6: Pigs produce in large numbers. They easily increase in numbers such that we can sell some and still remain with some.

M: Who can add on the goodness of keeping pigs?

R comes back and takes his sit.

W7: Pigs are very helpful because we sell and we get helped at home.

W5: Sometimes, we hire someone to cultivate my field and pay him two pigs and I will have done better.

W8: For wedding we hire people and they come to weed and that way, we get helped.

W2: We also use to pay someone I would hire to ferry my maize from the field to the village. This means that I have been helped

M: What about the disadvantages?

W7: Pigs are not good because they eat anything, more especially if there is no toilet. Because of that, some people do not eat pigs because of what pigs do or eat.

W6: The other thing is that when pigs are to die, they all die and you remain with nothing.

W5: Sometimes, pigs have diseases and one can contract the disease after eating pork.

M: What sort of diseases do you suffer from?

W5: As for me, I normally develop diarrhea once I eat pork.

M: Anyone who can add on the disadvantages?

W7: Sometimes, the reason we don’t eat pork is that if a child eats such as of this age *(pointing at her own child on the laps of breastfeeding age)*, the child can develop epilepsy?

M: Anything else?

W5: Sometimes, I can not eat pork with a free mind because you sometimes see a pig with a thread worn on the mouth and its eating.

M: Ok, so what are the threats of pigs?

Babies make noise

*Silence*

M: What are the threats that pigs pose to people?

W1: Pigs eat anyhow and they are very scary in as far as the spreading of diseases is concerned.

W5: As for me, the other thing is that pigs defecate on the yard any how, so they dirten the environment and bring a lot of diseases.

W6: The other thing is that, pigs would eat feces sometimes and when they come home, they would feed or rush to drink water from our buckets. Our children would come and drink the water from the same bucket. That’s who we see persistent diarrhea every now and then.

M: What do pigs do?

W7: They dirten the environment.

W5: As for me, pigs bring different things at home, diseases and sometimes death.

M: Are there other livestock that you keep as women?

All: Yes, cattle, ducks, chicken, goats.

M: Why do you rear them?

W8: Cows are helpful for cultivating and to ferry our harvest to our homes, ploughing. They make our lives easier.

M: What role do you play in rearing pigs? What part do you take in the rearing of pigs?

W7: We sell and get helped…

W1: *(before W7 could finish)* … We sell and get helped.

W5: With school, I would sell three or four pigs and then I would pay for my children’s school.

W3: They are also helpful during initiation ceremonies.

Slaughter and eat during initiation feast

W4: We also slaughter and eat.

M: Ok, now, what role do you women play in keeping pigs?

Selected voices: feeding them, giving them water

M: Do your relatives help you?

W8: Yes, the greatest help is when one has given you the feed. That way, the person will have helped you.

W1: They would help you with the feed while you look on your own as well.

M: Women, do you build enclosures on your own?

W1, W3, W5: No. it is men who do that.

W2: If it proves difficult, I can hire someone to come and build an enclosure for me and I give him a pig.

W2, W1	: … And you pay the person a pig, even a goat.

M: Who else apart from your relatives help you rear pigs?

W1, W4, W5: Just alone and four children.

M: Who makes decisions on whether to kill or sell a pig?

W3, W5	: It is men/husbands.

W6: For those who are not married, we just decide on our own.

M: So, those who are married do not make decisions?

W6: Well, that depends on the household. Men are more powerful than wives. Sometimes, a wife can not point at anything unless there is approval from the husband. However, other husbands respect their wives’ decisions. It depends on the households.

M: What about children?

W1: A child? Can a child make a decision?

All: No, a child can’t (amid smiles)

M: Who takes care of the pigs?

W1, W4	: Children and the owners.

W5: Sometimes, when you are at the hospital, the children will be feeding the pigs.

M: Do pigs eat human feces?

All: Yes

M: What is the advantage of pigs eating feces?

All: It’s bad; there is nothing good about pigs eating feces. It’s very bad.

W5: It will eat your feces and come to defecate on your yard again.

M: There is nothing good?

All: No, no, there isn’t.

M: What is bad about pigs eating human stool?

W6: When you go to the bush to defecate, it means you don’t need those things (feces) but pigs will bring those things back. They eat your feces and we eat the pig together with our feces.

M: What do pigs eat?

W6: Pigs eat anything, they do not choose, grass, meat, snakes, feces, pumpkins.

All: Everything

M: How often do we eat pork?

W5: No, we don’t eat that often.

W1, W3, W4, W8: We eat it very often, as we are talking here, people are chasing pigs to slaughter *(they all smile).*

W8: We eat very often.

M: During which occasions/seasons do we often eat pork?

W4 says something, they al look at her.

W4: They are eaten at any other time. There is no specific time or season or occasion.

W6: When there is a disaster we eat even if we know they have died of an illness.

Disaster = when pigs die in numbers on their own due to probably African Swine Fever

M: When else do we eat pork very much?

W5: This time when we have harvested. People pass through with pork in exchange with maize.

W1: That’s the one they prefer, they say it is very good.

M: What about at funerals?

All: It is pigs that we slaughter.

M: And at marriage ceremonies?

All: Pigs

M: Why do we specifically kill pigs on funerals, weddings, why do we kill pigs?

W6: It is said that it’s the kind of meat that when you slaughter you get more meat.

W5: When you are many, it will be enough for a lot of people.

W6: The other thing is, it is delicious and people would want to taste something nice. Again, pigs are a common livestock as opposed to cattle.

As she is speaking, the microphone drops from the table and they put it back

W5: The major reason is that it gives more meat. That’s why it is killed most often.

M: When one is selling pork, a young man is passing with a bucket of pork for sell, what do we consider before we buy?

W1: We see if it looks good, that’s when we choose to buy.

M: What else mothers? What do you mean “not looking good”?

W5: Not having fat.

W2: The way the pieces have been cut (either big or small).

W5: When they kill a non-castrated male pig, some pigs are bad, it’s not good. It may also have some *nsembe*.

M: Now, these pigs, when we kill them in our villages, are they tested before killing?

All: No *(smiling)*

W1: They just weigh the kilograms

M: If there was a rule that a pig should be tested before killing, what are your views?

All: It can be good.

W8: But can be difficult to some, because some business people are just interested in money.

W5: Testing is good to see if it is sick and can infect you, just like they do with cattle.

M: There is payment for having your livestock checked. What do you think?

They all speak and it results into noise.

W5: It is good, since it is to make sure that we eat health meat.

M: Now, we have reached on the preparation of pork. How do we cook our pork?

W5: As for me, when I buy, I get dish, I wash it, I put it in the pot, I fry it and I boil it a lot considering that pigs eat different things.

M: What about you, W3?

W3: It’s the same. I put it on fire, add tomato and fry it properly.

The child of W7 cries and makes noise.

M: What about you, W4?

W4: It is the same, you buy, you wash, you fry it and you start boiling. You just add water until it is ready

W7’s child is crying as W4 is talking.

M: Anyone with a different way of cooking pork?

All speak at the same time and it results into noise.

W1: If you are not very happy with the kind of meat that you have bought, maybe it has some *nsembe*, you boil it a bit, dry it and then cook.

W5: When you boil it too much, even the germs that would have been there will die.

M: What about the pork they sell in very small pieces at “*shokas*”?

W6: No, those are bad; they do not get cooked inside.

W5: No, that’s bad. Sometimes, I vomit once I start eating it.

M: What about roasting of meat what do you say about it?

W6: Roasting is not good. Boiling is better. Pork is never ready when roasted more especially inside.

They all laugh

M: Do you see some *nsembe* in pork meat?

M passes pictures of meat laden with cyst for the women to see.

All: Yes

W5: We see them very much.

M: What are your views on these *nsembe* in pork?

W6: Some of us think that those are eggs produced by worms.

M: What else?

W5: When you are eating pork that has the *nsembe*, they burst in your mouth, then you develop nausea and want to vomit and sometime you actually do vomit.

W2: Those things go in the stomach and they cause diseases.

M: What about you, W3?

W3: It is the same. They get into the mouth and burst and that is not a very good thing.

M: Have you ever eaten pork with *nsembe*?

All: Yes

W8: They are bad.

The women pass the pictures from one to another to have a good look.

M: When you find pork which has *nsembe*, what do you do?

All: We throw it away.

W7: But some will dry it and still eat it.

W1: Some people who sell pork meat do sell this kind of meat.

All agree with what W1 said by saying “yes, yes”.

M: Do you have toilets in your villages?

All: No, we don’t *(amid laughter)*

W4: But some have.

W5: Yes, some have, but not all of us.

M: For those of us who have, do we use them?

W5, W8	: Yes we do use them.

M: Every time?

All: Yes

M: What about those who do not have, what do we do?

All: They go to the bush, to the bush, as pigs follow us behind

They all smile and laugh

M: Why don’t we all have toilets?

*Silence*

M: Is it good to have toilets?

All: Yes

M: How is it good?

W5: We prevent diseases, since pigs can’t get into the toilet to eat our feces.

M: Who can add on?

W5: If you go to the toilet, pigs will not have access to the stool.

M: What are the advantages of not having toilets?

All: There is nothing good about not having toilets.

W7: Sometimes, we have husbands but they do not want to dig pit latrines and build the toilet.

W5: Sometimes, they will dig but when it develops a fault, they will not do anything until the rains come and submerge it.

M: With me, I thought that it is to help the pigs since we said that pigs eat human stool?

They all laugh.

All: No, no, no

W6: Most of the times, it is because of men’s laziness. And for these who are not married (*Pointing at some of the women*), sometimes, they do not have anything to pay a person who would be hired to do the job for them.

M: What about cleanliness or personal hygiene? Do we know cleanliness? What do you say?

W7: We need to have toilets. At least every household should have one.

W2: We need to have a dish where people can wash hands after using the toilet. We also need to construct a stand where we can be putting plates and pots, at least where pigs can’t reach.

W5: We need to sweep and clean our households and surroundings properly. Wash for the children; wash for the entire family and so on.

M: Talking about children, who cares for the children?

All: It’s yourself, it is you the mother.

W7: We need to see to it that children are clean, that they bath.

W1: Some people are clean but they don’t care about their children, they just care about themselves.

M: Is it good to take care of the children?

W5: Yes, we protect our children from diseases if we clean them and ourselves.

M: What else?

W7: Malaria and other diseases will be far from them. They will not usually get sick. We need to take good care of our children.

W5: Even if you don’t have soap, you need to bath the child, the child will look clean.

W1: You get surprised to see clean mothers but their children are dirty. It is unbelievable.

W2: The children are really dirty *(In agreement with W1*)

W6: You find a situation where you can clearly see the dirt on the children’s faces and the dirt looks as if it is beards.

*Laughter*

W5: That’s why these diseases will start from children to adults. We need to care for our children.

M: How about washing of hands?

W6: It is very important. But to be honest, for us women, sometimes we do and sometimes we don’t.

W1, W2, W5, W8: We don’t, sometimes, one bucket of water, even ten people can wash in there without changing the water.

M: What about when we come from the toilet?

W1: We need to wash hands.

M: Why do we need to wash hands after using the toilet?

W2: It is important

W6: Sometimes, the tissue or whatever we are using to clean ourselves after defecating will get turned; so we need to wash our hands.

W5: Even the scent in the toilet is bad. We just need to wash hands.

M: What about when eating?

All: We need to wash our hands.

M: Why?

W5: Because you may have germs in your hands, so it’s important to wash them.

M: Do we know about worms?

All: Yes, we know them. We have actually seen them before.

M: Where did you see them?

Moderator passes pictures of human stool with tapeworm proglottids for the women to see.

W6: From children.

M: How do they look?

W2: They look white, others are brown.

W6: Others look like cotton thread. Others are like flattish, like those we see in chickens.

M: Have we even seen things that look like this? *(Referring to the picture)*

R: Pieces, they look like the skin of a snake, but they are not as long as snakes.

M: Like maggots, do you know what maggots are?

All: No, no, no, we haven’t seen them before.

M: You haven’t seen them before? Even after a child has defecated?

All: We haven’t seen them before.

W6: There are others that look like mucus, they are white in color. We have seen these before.

M: What do you think about those? (*Pointing to the picture*)

W6: We think that those are just poisonous but not as worms. What we know as worms are those long ones. We just know thread worms.

W2: They have eggs *(ending by producing a sound which connotes fear).*

M: Do we know people who have removed worms before?

All: Yes

M: What were they complaining about? What symptoms did they show?

W6: If it is my child, she developed a potbelly, when she went to the clinic, she was given the medicine and she expelled three bundles of worms.

Potbelly = bulging tummy

W5: Even my child, she had stomach pains and had a potbelly, she became fine after medication.

M: W2?

W2: Whatever the child eat, he could cry. Every time he eats, he cries.

M: Where do these worms come from?

W6: They come as a result of eating soil, secondly, eating unwashed food such as sweet potatoes, hoppers, and not cleaning hands after using toilet.

W5: Even left over foods that were not covered, if they are given to children without warming them.

W5: We need to adhere to health tips from doctors. When we do that, we will find that even the children will not get sick.

M: Do you think that these diseases of worms can be prevented?

W3 stands to put her baby on her back

W6: Yes they can be, if we follow health rules.

M: Like which ones?

W6: We need to mind what to eat, immunization and following what health workers recommend.

M: Do clinics have the medicine for this disease?

W5: Yes, the medicine is there.

M: Do you think that having those kinds of disease is dangerous?

All: Yes, it’s very bad, they even kill.

M: Are there diseases that are worse than this disease? Which diseases do you think are more dangerous than the ones for worms?

All: Yes

M: Like which ones?

W5: They are there, like diarrhea, malaria, headache, dehydration and anemia.

M: Others! What do you say?

*Silence*

M: Are there diseases that are minor, less severe than this disease of worms?

*Silence*

All: There is no minor disease. All diseases kill. All are painful. Since they all affect someone’s body parts.

M: Is this disease very dangerous if you have it?

All: Yes

W3: The outcome of any diseases is death

It means that nay diseases posses a sing or is a prerequisite to death

M: What would you do if someone you know has worms?

W5: I can advise the person to go and see the doctor at the clinic so that he or she is checked in case he/she has worms because you may die if you do not do anything.

M: What are the symptoms of worms?

W5: Body weakness and the hair lighten.

W6: Others, they develop too much appetite and others, it is loss of appetite.

M: We have seen *masese* in pork meat, right? What do we think is the relationship between the *masese* found in pigs and epilepsy?

*Silence*

R: Do we know epilepsy?

All: Yes

W5: I have a grandchild, he used to have epilepsy, when I stopped giving him pork, and epilepsy also disappeared.

M: Others?

W7: I also have two children who had epilepsy when I stopped them from eating pork. They have not been fitting for 4 years now.

M: Where does epilepsy come from?

All: From pigs.

The door opens and all turn their attention to the door.

M: What are the symptoms of epilepsy? How can we know that this person has epilepsy?

W7: Fever when he starts complaining of fever, soon you see the person fitting (*amidst approval from other women*).

W5: Sometimes, it’s cold, and in a few minutes the person would be fitting.

M: Do we know people who have this disease?

All: Yes

W5: For me. I have a grandchild who has this disease.

M: Where do you think he got this disease from?

W5: For me, I think its pigs because once I stopped him, epilepsy all ended.

W6: I have a question, what about those who do not eat pork but have epilepsy? I am behind on this one, I wanted to ask.

M: That is a question women, who can answer? She is saying that you will find someone who does not eat pork but they still have epilepsy. Let’s answer.

W5: For me, I think acute malaria can lead to fits.

M: Others?

W7: It is malaria, because a child would be fitting but when we come here, after they are treated for malaria, the child would stop fitting. Even us adults, it’s the same. When malaria is too much, we start fitting.

“Come here” = clinic

M: Do we think we can we prevent this disease of epilepsy?

W7: We need to be flexible and follow the doctor’s advice. We need to follow what they tell us because if we don’t, we would get into trouble.

W5: We need to adhere to the health tips that we are given.

M: Who give these health tips?

W6: Sometimes, health workers come to give talks, but because of ignorance, we say I am very busy and we don’t go to such meetings when those people have come for us. In that way, we miss a lot, now when diseases have come, they will affect those of you who did not go for the talk.

M: Is this disease curable? Are there medicines that can cure this disease?

W5, W6: Yes, they are there.

M: Do we know them? Can we find them at the clinic?

W2: Health personnel are the ones that know better.

M: Any other?

W6: Some go to herbalists, some go to the clinic.

R: Do people recover?

All: Yes, they do.

W5: But others die.

M: Have we ever seen those who have recovered?

All: Yes

M: Where did they get the medicine from?

W5: Others got cured from the clinic, others at the herbalist.

M: How scary are this disease?

W7: If it is persistent, the child will eventually die.

W5: You might think that the child is just fitting; the next thing you see is death.

W6: This disease is not respectful. Sometimes, it might attack the person so unexpectedly and one can even have seizures on the road. One can fall even at night on fire.

W5: Even in the pond as she draws water. We have seen such kind of deaths especially of elderly people.

M: Are there more dangerous diseases than epilepsy?

All: Yes

M: Like which ones?

W5: Insanity.

W6: In fact, insanity and epilepsy are related. Dying is easier.

M: Which ones are minor diseases to epilepsy? W3, what do you say?

W3: There isn’t. All illnesses are painful and bad.

W5: Yes, even when you hurt yourself on the finger, you complain. All illnesses are bad.

M: Do we have a lot of people in our village who suffer from epilepsy?

W5: These are there but they are few because of clinics. Others are recovering even now after getting medicines from the clinic.

M: Is having this disease dangerous or very serious?

All: Yes, it is.

M: Why?

W5: Yes, one can die at any time.

M: What is the effect of this disease on everyday life of a person?

W5: If we don’t take the person to the clinic, the person may die if we do not take the person to the clinic. It’s a very bad problem.

W7: The problem is that it makes someone behave like a mad person.

W5: Sometimes, a child/person can undress in full view of everybody. That’s very bad.

W7:…They undress, it’s actually madness.

M: Anyone who make any addition?

W5: On the well, one can fall into the well and one can also be hit by a car.

M: If there was a rule in an attempt to prevent diseases that all pigs should be closed and left in an enclosure. What would be your reaction?

W6: No, it wouldn’t be a good idea. For example, if we can close pigs for five days, they lose balance, become lame and they will be very unhappy, very weak. But if you allow it to go out even for three days, you get surprised and say, is this the pig that was so weak? So, maybe we need a different breed of pigs not these that we have, they are difficult.

W5: They wouldn’t be happy.

W1: They wouldn’t be happy and comfortable. They will just want to go outside.

M: Anyone with different opinions? Would it be good to enclose pigs so that if it is to walk, they should be roaming within the fence?

W6, W7: Yes it would be good.

W5: Even if they lose weight, as long as our lives are safe and we remain alive.

W1: I think a fence is better not a small enclosure, just a large fence so that pigs have a change to maneuver, without that, all our pigs may die.

W5: That’s better, because in a small enclosure, they can all die.

M: What if there was a rule to say that every household should have a toilet and that no one should be going to the bush?

All: That would be very good.

M: If pigs are kept outside, what would be the problem?

Most: Diseases.

R: What about an enclosure. What challenges can it possess?

All: Yes

W5: Food, we may need to provide food.

W1: Yes, because food is also a problem more especially in the rain season.

M: What if the rule was about boiling the pork meat; that everyone should be boiling pork very much. Would there be problems with such a rule?

All: It will be good.

W5: It can kill germs. All the germs would die and our lives would be protected.

M: What about roasting? What about those of us who like roasting pork?

W2: It will be bad. No diseases can be killed.

M: What about calling Veterinary offices to check pigs every time one wants to kill a pig? If they said that all meat should be inspected before eating, what would be your reaction?

W7: It will be good because our lives would be protected.

W5: That would be good, because that’s when we can be seeing how diseases can be coming to the village.

M: There won’t be any problem?

W6: There will be a problem. Our husbands are very stubborn; they can’t adhere to that because they say they have authority over their livestock and that there is no one who can tell them what to do. That’s why; there is need for education campaigns and sensitization in the villages. Headmen should organize for these lessons, lessons are very important because if you are not informed, it is very difficult.

M: What about you W3?

W3: It can be good.

M: If it is found with diseases, what would it be?

W3: It would be good for our health.

M: In checking the meat, the Vet Officer needs transport and payment for vaccines. What do you think? Do you think you can manage to be paying even just a small contribution?

W5: It will be good. I can be selling one pig to pay so that the rest of the pigs are protected. I mean, we even bring our children for immunization in the same vain.

M: What about vaccines for pigs?

W5: It is just okay, because if we do that, it means that even when we eat, I will know that it is safe and can take my health will be protected.

M: These things are paid for, so you can be paying for the vaccines?

W5: Yes, I have a lot of pigs; I can sell some and use the money to pay for some because I know that pigs produce in large numbers, sometimes, ten, fifteen, and seventeen and so on.

M: How much do you think you can afford per pig?

W6: I have to pay what it costs per pig and I have to pay knowing very well that pigs are helpful to me.

M: What if we said that everyone in this village should thoroughly wash vegetables before cooking?

W7: It would be good.

W5: Others don’t wash, sometimes; you find that there is a snail on the vegetable. We should be washing.

M: Others?

W5: Sometimes, maybe the vegetables were sprayed recently; you need to wash lest we suffer the effects of spraying.

W8: Even the dirt would be there.

M: What if the rule was about compulsory cleanliness? That whether you are young or old, we should make sure that our bodies are clean. What would we say on that, knowing that we don’t bath every day?

W3: Yes, we do not bath every day. Sometimes, we skip even two days, more especially with this kind of cold

*Laughter*

W7: It would be good to be clean.

W2: It is for our own good to be clean.

W5: We need top even wash clothes because if we bath and put on dirty clothes, then we would have done nothing.

W6: We need to bath everyday, so that even if we skip three days, we still look clean.

W5: But for clothes, they will look dirty. People will see the dirt and will say that the person does not wash and that diseases can easily get to such kind of a individual.

M: What if you have clean clothes but you do not bath?

W6: Oh, it’s easy to see the dirty on the throat, hands, and legs

*Laughter*

W5, W6: Even the scent will suggest that the person hasn’t bathed.

W6: Even the skin looks so thick, even the body looks weak. You can even know when someone hasn’t bathed and has just applied lotion, the dirty will be evident.

W5: Even malaria, it is easy to contract it.

M: What if it is commended that all the people should go for tests on worms and all those with worms should be treated?

All: It will be good.

M: Why would it be good?

W5: As for me, if they find it, I would get the medicine and I would get well.

W6: Even diseases will be very far from us. That’s when people will say that, ‘these people do not get sick, so people will even suspect that you used some magic. But those who do not get treated, they easily get sick. You find that in no time, they have diarrhea, malaria, this and that all because of not taking care of one self.

W5: We need to take our children to the clinic for vaccination so that our children can be safe. Even if we adults don’t want to go there, we need to take our children there so that they are protected and their lives go ahead.

M: Well, I believe we have reached the end of our discussion. We have talked about the advantages and disadvantages of having pigs, those who feed pigs, until we reached on the advantages and disadvantages of toilets, the advantages of bathing and the disadvantages of not bathing. And we reached a point where we suggested control measures, that in an attempt to avoid illnesses, what our reactions would be, if we had toilets, enclosures for pigs, taking all our pigs for vaccination, and that all our pigs should be checked before we kill them, and that all vegetables should be washed. This is the end. In case there are questions, you now have the opportunity to ask.

*Silence*

M: If there are questions, you are free to ask.

S*ilence*

M: There are no questions?

All: No questions.

M: Ok, thank you very much. OK!

All: Alright, thank you.
