# Supplementary material for: Why Latrines Are Not Used: Communities’ Perceptions and Practices Regarding Latrines in a Taenia solium Endemic Rural Area in Eastern Zambia
Source: PLoS Negl Trop Dis. 2015 Mar 4;9(3):e0003570. doi: 10.1371/journal.pntd.0003570 (PMC4352092; doi:10.1371/journal.pntd.0003570)
Supplement: S1 Dataset — (ZIP) [file pntd.0003570.s001.zip › FGD transcriptions_Zambia-2010/Interviews/Medical officer Interview_07-08.doc]

**Title : Interview with MIDWIFE**

**Date :** 07/08/10

**Site :** Vulamkoko Rural Health Centre

**Location :** Katete district, Eastern Province, Zambia

**Duration (total time) :** 47 min	**- Start :	- End :**

**Informed consent:** ??

**Moderator :** Dr E. Kabemba

**Transcription:** Dr E. Kabemba

**Abreviations :**

**M:** moderator**, Res:** respondent**, I:** intervener

**Comments about participant :**

**Text:**

*Introduction*:

**M**: Thank you sir for according us this opportunity to talk to you about pigs and your views and perceptions about a disease that they transmit the pork tapeworm. I welcome you to this small discussion...

**Res:** Thank you.

**M:** ...and ehhh, I ask that you feel free, say anything you want according to what I will be asking you, whatever you say there is no right there is no wrong answer, ok.

**Res:** Thank you very much, you are welcome.

*Discussion*:

**M:** We begin by looking at the positive and negative aspects of pigs, you have worked in this area for quite some time, what do you think are the positive aspects of people keeping pigs?

**Res:** Ahhh, the positive aspects of keeping pigs in this area is that it’s one of income generating activity to the community. That could be the major thing.

**M:** Ok, for income, anything else?

**Res:** They use it as meat.

**M:** As relish?

**Res:** Yes as relish.

**M:** So this income is household income or for some special purpose?

**Res:** Household income especially. For example if one has a very big field to cultivate, they can hire some people to do that job for them and pay them a pig.

**M:** Do they use pigs for funerals as well?

**Res:** Yes, sometimes they do so. Actually most of the times, because the ox or the cattle is rarely killed during funerals

**M:** Rarely slaughtered.

**Res:** So they use pigs and goats.

**M:** Ok, which one do you think people here prefer goat or pig?

**Res:** They prefer pork because pork is said to have a lot of meat than goat.

**M:** Ok. What do you think are some of the negative aspects about pigs?

**Res:** Ahhh, the negative aspects about pigs that I have observed in this area is that they are not kept in houses. They are just left out to move freely, like that.

**M:** How is that a negative aspect, the free roaming?

**Res:** It’s negative because, a lot of people don’t use pit latrines and hence the pigs go and eat that human waste.

**M:** Are there ways in which you think pigs are dangerous to humans here?

**Res:** Of course yes, the possibility of transmitting the worms is so high.

**M:** Ok.

*Silence*

**M:** That’s the only dangerous part?

**Res:** And, from what I heard sometime back, it’s like a lot of epilepsy is connected with pig related issues.

**M:** (*Laughs*). Interesting, ahhh, how do you think the people feed their pigs?

**Res:** They use the husks from the maize, when they pound maize the husks are used by.....

**M:** Maize bran?

**Res:** ...yes, they soak them, sometimes they take water and mix with the husks then they feed the pigs.

**M:** Ok. Do you think everybody is able to feed their pigs?

**Res:** That is not so, many times during the rainy season a lot of people have no maize and pigs are left like that, they don’t feed them in the way they are supposed to be fed.

**M:** So the pigs are left to scavenge?

**Res:** Yes.

**M:** Do you see any danger, in people’s health, of pigs feeding on faeces? Is there any danger?

**Res:** Of course there is because human are also harmful and for example if there is ahhh, an outbreak of a disease like cholera, the possibility is there that a pig can also become a carrier of the disease and spread it to other areas.

**M:** Any other danger apart from......

**Res:** Then other is possibility of transmitting the ahh, the worms themselves.

**M:** The worms themselves?

**Res:** Yes.

**M:** When do you think people consume pork a lot?

*Silence*

**M**: Is it on an everyday basis or there are special occasions when they eat the pork?

**Res:** Like this area it’s on daily basis. If you have come across, as you come along the road you have seen the market at Azele, there is always a pig hanging downwards meaning that it’s on daily basis.

Azele = It is a market situated at a village called Azele Kacheka, about 6kms from the health centre

**M**: But are there special occasions when pork meat is consumed more?

**Res:** Ahhh!

**M**: Are there special occasions?

**Res:** When there is a funeral there is a pig slaughtered and in such circumstances pork is not properly cooked and hence transmit the disease.

**M**: So, for, say a funeral, where do you think the pork comes from?

**Res:** They buy it from people within and if the owner of the funeral has pig they just get some and slaughter.

**M**: Do you eat pork yourself?

**Res:** No I don’t.

**M**: Why?

*Laughter*

**M**: You were stopped or you used to eat before?

**Res:** I stopped some maybe fifteen years ago after learning how the pig was being kept, because where I was in Lundazi pigs don’t go outside and....

Lundazi = a district about 285kms from Katete, Eastern Province

**M**: They are housed nicely?

**Res:** ... feed on human waste...

**M**: Ok.

**Res:** ... so, I used to eat but when I came to Katete, I stopped...

**M**: You saw the free roaming pigs?

**Res:** ...just because it’s like a stray dog going around like that, so I stopped, my entire family don’t eat.

**M**: Do you think is there anything that people should look out for when they are buying the meat? If someone comes and they are selling meat in a dish, is there anything you think people should look out for?

**Res:** I think there is something they should look out for, there are times when this pork is sold it has some particles in the flesh, those particles look like blisters and some call it as *masese* which I think is not actual *masese* because that could be the start of worms, something or so. So they have to look for those things and see if they are buying right meat and.......

**M**: So if they look at that meat and it has....., if they look at that meat and it has those cysts, should they buy or just leave it?

**I (Séverine)**: ????????....

**Res:** I think that meat should be discouraged; people should not buy that meat because that is a possible source of infection.

**M:** You have seen these cysts before, in the meat?

**Res:** Yes I have. And it’s not only in the flesh, but also in the lungs and the liver, they are everywhere sometimes.

**M:** Do people in these parts clearly eat meat that is infected with these cysts?

**Res:** Am sure there is.

**M:** You think they eat?

**Res:** They do.

**M:** You have seen a pig hanged in one of the villages and it’s clearly infected with cysts and people are buying?

**Res:** In the villages it’s not hanged but they put in a basket and move around door to door selling that meat.

**M:** Ok. So according to you, what is the main risk behaviour performed by people in these parts, what do they do that puts their health at risk, the people?

**Res:** The high risk that they....., those pigs are not housed, so they are left to move freely and by so doing they get a lot of dangers.

**M:** Are there any health related issues associated with pigs that you know of?

**Res:** I have heard of one to be epilepsy, some are believed to be having feats because they consume a lot of pig meat or pork and there is actual worms sometimes, those round worms and these are few things I know about.

**M**: How do you think those health related issues can be reduced?

**Res:** They can be reduced if only some measures are taken, one, if people can consistently use toilets that can be one of the achievements, two if the meat itself when it is slaughtered, they should see or seek for a medical personnel to examine that pork so that it is declared free if infection and if they can burn all the meat that contains *masese* in the flesh or in the lungs, that could probably reduce the burden.

**M**: Have you ever heard of taeniosis?

**Res:** Taeniosis, yes.

**M**: Have you ever observed proglottids in human stool?

*Silence*

**M**: As you practice, has anyone ever brought stool or proglottids to you?

**Res:** Yes, sometimes they have but we are not very sure 100% because at this facility, we do not screen the stool very well and that type of specimen under the microscope to determine the actual cause of.

**M**: But you have heard of taeniosis tapeworm infection?

**Res:** Yes.

**M**: What do you think are the symptoms of tapeworm infections in humans, in children, in adults?

**Res:** Usually there is........in human beings a tapeworm can reduce appetite, it can ...sometimes someone can have a bit of fever, a cough and sometimes someone can have even the tapeworms seen in the stool.

**M**: Ok. How do you think humans get infected with this tapeworm?

**Res:** Ahhh, the human beings can be infected through, one, maybe if they walk on the ground without putting on the shoes and the legs get contaminated with the faeces, they can easily penetrate through the toes, even consuming it through the meat its.

**M**: Which meat? Pork meat?

**Res:** Yes.

**M**: How do you think this disease can be prevented, these worms, these tapeworms?

**Res:** Prevention basically is on good sanitation and hygiene.

**M:** Can it be treated, you think?

**Res:** Of course yes, it can.

**M:** You have been treating people?

**Res:** It can be treated with Vermox® or mebendazole.

**M:** You use mebendazole mostly here?

**Res:** Mebendazole, yes.

**M:** Not albendazole?

**Res:** Albendazole we have as well.

**M:** So when do you get to use mebendazole and when do you get to use albendazole?

**Res:** Specifically it’s like these drugs work almost the same, so it’s....

**M:** You treat with whichever is available?

**Res:** Yes whichever is available, but at the moment we have both drugs available.

**M:** Is there... you think there are diseases here that are more severe than the tapeworm, more than worms?

**Res:** More severe diseases........public health is a lot of malaria, there is a lot of this respiratory tract infections...

**M:** Like TB?

**Res:** Yes, TB, we have had a lot of it reported from certain villages, like at the moment we have a few patients who still have sputum positive at zero, two moths and the other months still positive despite taking the drugs and that causes a threat.

**M:** Any disease that you think is less severe than the tapeworms, than the worms.

**Res:** Less severe?

**M:** Yes.

**Res:** We have a bit of.....Maybe these other skin infections like scabies; those are very rarely seen....

**M:** So they are less severe?

**Res:** ...a bit of bilharzias because maybe there not many streams, they are not there. Those are less common.

**M:** The villages that you cater for at this clinic, do a lot of people suffer from the tapeworm?

**Res:** I should believe so because sometimes they can easily bring sometimes roundworms, tapeworms they are very difficult for some to identify to say this is a tapeworm, though sometimes when you go out there carrying out mass sensitisation, you can’t tell them how the tapeworm look like.

**M:** But you get a lot of worms?

**Res:** Worm infestation, the common one is roundworms yes, especially Ascaris*,* yes*.*

**M:** Do you think being infected with a tapeworm is a serious health problem?

**Res:** To me yes, I think to me it is. (*Laughs)*

**M:** Why, why do you think it’s a serious problem?

**Res:** Because it’s like a foreign body in your body and it’s a health hazard to your health.

**M:** What are the consequences of being infected with this worm?

**Res:** The consequences could be growth retardation, there is anaemia and even malnutrition comes in because some of the food is being consumed by the worm itself.

**M:** How does the presence of the worm impact on someone’s daily life? Does it have any impact on someone’s daily life?

**Res:** I feel it has because the worm itself can cause somebody not to feel well and if someone doesn’t feel well then les productivity and health wise as well that person will not feel free to do other things and have a normal life that is expected of him.

**M:** Do you know the disease called cysticercosis?

**Res:** Cyst...........?

**M:** Cysticercosis.

**Res:** No, I would say no.

**M:** But you have heard of epilepsy?

**Res:** Epilepsy yes.

**M:** What are some of the symptoms of epilepsy?

**Res:** Symptoms, first I would say signs of epilepsy, for example you are standing in a queue you may find somebody goes down and starts jerking, convulsing, there is froth on the mouth and when they stop convulsing they will go into a deep sleep and sometimes there is incontinence, many times not sometimes, but many times they have incontinence and its either of urine or faeces. That is a sign; something that one can see with their eyes but symptoms is something that someone can feel.

**M:** You have seen cysts in pork meat?

**Res:** Yes.

**M:** Do you think that’s a human or an animal disease?

**Res:** Ahh… That could be both.

**M:** Could be both?

**Res:** Ehhh, to an animal I would say an animal can be a carrier, because I have never seen an animal having feats or convulsions (*laughs)* so it’s like a carrier while a human, when it goes to a human it becomes a disease.

**M:** So you think you can see those cysts in humans?

**Res:** I think I can, I think so.

**M:** How do you think this would come about? How do you think a human being would get infected with the cysts?

**Res:** When one consumes that (meat with cysts) it will be digested, maybe it’s not well cooked, it will be digested, and from there it will go to the liver, then in the liver they will multiply maybe and then go to the different parts of the body and form those cysts as well.

**M:** How do you think people get infected with...... how do people get to have epilepsy?

**Res:** There is a wide range of some beliefs, some people say it’s inherited but some say its witchcraft, but with the current trend that I have seen, I think the tapeworm is a contributing factor, the worm infestations and also the fact that people consume a lot of pork

**M:** Do you know of people that have epilepsy?

**Res:** Having epilepsy any how like that?

**M:** Yes, any one that has ever had seizures and you think they have epilepsy?

**Res:** Not really.

**M:** You don’t treat people for epilepsy here?

**Res:** We do.

**I (Severine):** And how do you do it?

**Res:** Ahhhh!

**M:** How do you treat them?

**Res:** Treatment of an epileptic patient, if the patient is convulsing you don’t need to fidget with that patient, just move any dangerous items that might be near that person so that he or she doesn’t hurt herself. Ahhh, later on if you ......sometimes they jerk and try to grind teeth, if there is protection that can protect teeth, try to put a soft cloth in the mouth so that they don’t bite their tongue.

**I:** You have seen an epilepsy crisis?

**Res:** Yes, I have.

**I:** Is a lot of it happening here or in the field?

**Res:** Here, it’s not so common, but the ones that I have seen it when I was working at the hospital (in the centre of Katete) and sometimes when you are in the village, and so from there I have encouraged a few of them to come to the hospital and get medication which can prevent the feats from happening.

**M:** So it’s more of prevention than curing?

**Res:** I think on curing it’s ... I don’t know if it can be cured because it’s something that I don’t how it can be cured. Though prevention is better that cure, so they have to take the drugs on a daily basis to try and stabilize whatever provokes the convulsions.

**I:** So are you able to identify what ehhhh, give the origin of those crises?

**M:** Are you able to quantify?

**I:** No, no, not quantify, find, what are the origin of the crises?

**Res:** Ok.

**M:** In the cases that you have seen, is it possible to identify what could have caused the convulsions?

**Res:** No, **at many times the causes are not very well known.**

**M:** Do these people use traditional healers for such?

**Res:** Of course they have done that several times and when they fail they come to the hospital.

**M:** So they try with the traditional healers?

**Res:** That’s the common thing which I have seen here, even a child who has malaria and that malaria is complicated to cerebral malaria and the child starts feating, they will first go to the witchdoctors and seek treatment, they come here later.

**M:** Ahhhh!

**Res:** So (*Laughs*) that is one part which is very much challenging, how to overcome that.

**M:** You have said that cysticercosis can be in animals and in human as well, do you think it can be treated, if say it’s in a human being?

**Res:** I feel it can be treated.....

**M:** And it can be prevented?

**Res:** ...and it can be prevented, yes.

**I:** But how?

**M:** How do you think it can be prevented?

**Res:** Ok, first thing, when you treat that thing, ahhh, the prevention is, maybe that person will have to go on some....ehhhh....on....either you’ll have to educate them not to start eating maybe the infected meat or the pork itself to stop, that can help because the causative organism is in the pork itself.

**M:** So if someone has these cysts, how do you think you treat them? Is it possible to treat them?

**Res:** I feel it could be possible because the modern world has a lot of research and hence drugs could be there.

**M:** Are you afraid of getting these cysts, of getting epilepsy?

**Res:** Oh yes, it’s something that is frightening, when you see one is doing that (feating) and feel that problem is on you....

**M:** Are you afraid of getting the cysts?

**Res:** .... it’s a threat yes, it’s a threat to some human beings because I feel when one is convulsing there is a bit of brain damage and at the end that person will not be ehhh... a full...ehhh....a well.....what should I say?

**I:** Normal minded?

**M:** A normal, normal minded person?

**Res:** Yes, exactly. So there are some disabilities, developmental, in the brain.

**M**: So we have talked about this cysticercosis and epilepsy, can you mention a disease that is more sever than epilepsy.

**Res:** More severe than epilepsy? Ok, in .......more severe than epilepsy?

**I:** And, and why?

**Res:** Which is caused by what?

**M:** Any disease, any, I mean you have come across a lot of diseases here..

**Res:** Oh, ahhh.

**M:** ... according to you is there a disease that is more severe than epilepsy?

**Res:** More severe cases, we have had meningitis, ahhhh, but it’s not so common.

**M:** Would you consider HIV to be more severe than epilepsy?

**Res:** HIV is not so severe to me because HIV does not kill actually, it does but in the other way round, so the severity of it is gradual while these other conditions are so quick.

**M:** Like epilepsy?

**Res:** I don’t know how long epilepsy can take to...

**M:** We are comparing with epilepsy, if you compare HIV and epilepsy, which one do you think is more severe?

**Res:** I think epilepsy is more severe because it damages the brain.

**M:** And HIV doesn’t?

**Res:** HIV doesn’t.

**M:** Any disease that is less severe than epilepsy?

**Res:** Maybe syphilis ehhhh, and these others.....I cannot mention some of these diseases because they are running away from my head (*laughs*).

**M:** Do you think a lot of people suffer from epilepsy in these villages?

**Res:** I believe so. Only that they are neglected and not taken to the health institutions for treatment. I think there are many out there who are not coming to access treatment.

**M:** Do you being infected or having epilepsy is a serious health problem?

**Res:** It can be and as I said earlier on, if one has repeated convulsions that person ca have brain damage.

**I:** Do you think an epileptic person can give epilepsy to another person?

**Res:** Ahhh, that is not true, though traditionally they feel it can be transmitted in such ways but it is not that way.

**M:** But how do you feel yourself, can it be transmitted from one person to the next?

**Res:** It cannot be.

**I:** And, how do people here see people who have epilepsy, do they consider them?

**Res:** Ahhhh.

**M:** Do they stigmatise them?

**Res:** Of late they have interacted very well, I don’t know of the previous year’s how it was but long time ago where I had stayed in Lundazi, these people were neglected and if one has epilepsy they thought that if one starts convulsing they would shun away from that person for fear of contracting the disease. They thought that if one farted, they would say that the smell would make one contract the disease but it is not true.

**M:** Do people who have convulsions? Who have epilepsy before they get married, do they get married?

**Res:** Oh yes they do marry and get married.

**M:** Ok; any impact that this disease can have on one’s daily life?

**Res:** Ahhh, on daily life, the only thing is that they may experience a bit of threat because those people are vulnerable to maybe when they go to draw water, to have bath or play sometimes they can drown due to the convulsions or the attacks or get burnt or hurt themselves with dangerous objects due to the convulsions.

**M:** Imagine we came and proposed different measures to control the tapeworm and also the cyst in both humans and pigs, according to you, you have worked in this area for quite some time, what would be some of the barriers that we would encounter? If say we came and said, can everybody confine their pigs, build fences and houses for their pigs, any problems that we would encounter?

**Res:** Problems might be there, first thing would be what are they going to feed the pigs because pigs we know they need, they demand a lot of food for them to thrive, and for them to be house sometimes it will be a burden to the ones who look after them because they may not have enough food.

**M:** So because of that people wouldn’t take kindly to such a control measure?

**Res:** Maybe with education, they may accept such suggestions and idea because what matters is when you educate somebody they get or they see the dangers and benefits from the suggestions and there might be a change.

**M:** So with education they would comply and start confining their pigs?

**Res:** I think so; they would change.....

**M:** Are they receptive to....

**Res:** ... though change does not happen there and then.

**M:** ...do they listen and get educated to you in your various health talks, education campaigns and....

**Res:** Ahhh, many times when you are giving health education, they feel sometimes you are discouraging them from doing things they think are still good to them, like for example when you say, when you slaughter an animal and it’s found to be infected, that animal has to be burnt, they say no. (*Laughs*)

**M:** What if we came and said every household should have a pit latrine or a toilet and not just have them but use them.....

**Res:** (*Laughs*) Use them!

**M:** Are there any problems or barriers that we would face?

**Res:** Ahhh, barriers would be there and there is one thing that someone should know. Firstly, they have to know what is the importance of that thing you are telling them to do. You may say...ok, when you are imposing something on someone, they don’t feel it’s good to them, they can do it but later on they won’t use that thing. The best way that I feel it could be done is educate the civic leaders, I mean the....

**M:** The traditional leaders?

**Res:** ....the traditional leaders the importance, later on those should educate their subjects. And I have seen. There was a place where I went, they had built some very nice IVP latrines but when I went back there I found that it was a storeroom for grain. (*Laughs*)

IVP = Improved Ventilated Pit

**M:** What if we came and said all those that eat pork should not roast it but they should eat it boiled.

**Res:** Ahhh....

**M:** You think we would face problems with such a control measure?

**Res:** Sometimes you may have difficulties to overcome them but some people, I have heard them say the roasted meat is better than boiled but in actual sense the boiled meat is better than the roasted one because the roasted one does not... is not well prepared, inside its still fresh and they eat something like raw meat.

**M:** So we would face problems changing their mind sets to start boiling the meat?

**Res:** Ahhh, eventually they can change because when you educate them you change them bit by bit.

**M:** And if we came and said that all those that slaughter their pigs, the pig should be inspected before slaughter and the meat should also be inspected, can we face problems with such a control measure?

**Res:** That cannot be a very major problem. There are times when the EHT (Environmental Health Technician) is called upon when an animal is slaughtered. Those are the rolls of the EHT to inspect the meat to see that it is free from these diseases and infestations and recommend it for human consumption.

EHT = Environmental Health Technician

**M:** He does that?

**Res:** Ahhh, the current EHT has not been able to do that, ahhh, they rarely tell him that they are slaughtering an animal for sell or for consumption so that he can inspect. You find that sometimes they are just going round selling that meat uninspected.

**I:** Which works better, if the technician goes to their houses or if they come with the meat here (clinic)?

**Res:** Normally what it is.....ehhhh, like at the Katete stores they have a place where they slaughter these animals and are taken there for examinations. But it’s very different from our setting here in, an animal can be slaughtered at the village, they will start selling it there before the EHT even knows that they have killed an animal and it’s on sell and they don’t....

**I:** They won’t call him?

**Res:** ...they call for meat inspection.

**M:** Imagine they called and he inspected and found that the meat is not fit, would they comply with destroying that meat?

**Res:** That much, I don’t think they would because sometimes these people here, they eat even an animal which has died on its own.

**M:** Or they would beat up the EHT for destroying their meat?

**Res:** No, it would be a controversial issue because they would feel that he has not done the correct thing.

**M:** How about washing of vegetables? If we said lets all wash our vegetables before cooking?

**Res:** That would not be a problem and a good washing.... practises of washing vegetables, I think if they are taught and I have talked to women when they come for ante-natal or when we are doing under five clinics, the good way of washing vegetables is washing them before they cut them so that all the dirt is washed out nicely.

**M:** Do you think they practice what you have taught them?

**Res:** I think they do.

**M:** Ok. And good body hygiene? If we said they should bath, please wash your hands after using the toilet, before eating anything?

**Res:** That cannot be a problem and can help yes, there is a belief that I have heard many communities that when they handle faeces or waste of the baby, it is not harmful. (*Laughs*)

**M:** So they don’t wash their hands after handling or changing their babies?

**Res:** With this current situation they are changing.

**M:** They are changing?

**Res:** They are changing, yes.

**I:** Is there enough water available to do that?

**Res:** Enough water is not there because sometimes they have to walk long distances to fetch water and that a very big challenging issue.

**M:** What if we brought medicines; we brought treatment for the tapeworm and said all should come and get treated, you think we would face problems with such a control measure?

**Res:** I don’t think that can be a problem; I think everyone can accept and access treatment.

**I**: They are not afraid about vaccines and vaccination?

**Res:** No.

**M:** And if we came and said we are going to treat or vaccinate all their pigs, you think we would face problems?

**Res:** I think that can also be a welcomed thing or idea, especially that when you educate them they want their animals to be healthy; I don’t think you can have any problem in that area.

**M:** What if we said, ok we have a vaccine for the cysts but people should contribute something?

**Res:** They might contribute since people who rare cattle sometimes they are able to give something so that they (Vet officials) give vaccines to their animals.

**M**: But that cattle, would it be the same with pigs?

**Res:** I don’t think there would be any......any.....

**M:** Any barrier?

**Res:** ...any barrier because these small animals are also wealth to them and they feel if one has no animals it’s like you are nothing. For example if I have ten chickens and I need my chickens to reproduce, I don’t want them to die, I would easily access the vaccine for my chickens.

**M:** (laughs), thank you very much sir for according us this chance to talk to you. So we have briefly looked at the positive and negative aspects about pigs, your perception of risk behaviour with regards to raising pigs, your knowledge and perceptions of the diseases that are transmitted by pigs namely taeniosis and cysticercosis, and also your perceptions of the control measure and what barriers we would face if we introduced these control measures. Ok. Thank you very much.

**Res:** Thank you.

**I:** Thank you.
