# Supplementary material for: Why Latrines Are Not Used: Communities’ Perceptions and Practices Regarding Latrines in a Taenia solium Endemic Rural Area in Eastern Zambia
Source: PLoS Negl Trop Dis. 2015 Mar 4;9(3):e0003570. doi: 10.1371/journal.pntd.0003570 (PMC4352092; doi:10.1371/journal.pntd.0003570)
Supplement: S1 Dataset — (ZIP) [file pntd.0003570.s001.zip › FGD transcriptions_Zambia-2010/Interviews/Vet. Ass. Interview_05-08.doc]

**Title : Interview with VETERINARY ASSISTANT**

**Date :** 05/08/10

**Site :** Kakiwa Rural Health Center

**Location :** Petauke district, Eastern Province, Zambia

**Duration (total time) :** 33 min	**- Start :	- End :**

**Informed consent:** signed and available

**Moderator :** Dr E. Kabemba

**Transcription:** Dr E. Kabemba

**Abreviations :**

**M:** moderator**, Res:** respondent**, I:** intervener

**Comments about participant :**

**Text:**

*Introduction*:

M: Good morning.

Res: Good morning.

M: I welcome you to Kakwiya Rural Health Centre where I want to talk to you about pigs, what you think about the way pigs are kept and the disease that are associated with pigs. What you say is in confidence and we’ll record it on a video but for the sole purpose of us going translate and transcribe the interview. It will not be taken to any radio station or TV station, nothing of that sort. Ok?

Res: Ok.

M: You have any questions?

Res: I think as at now, I think you can start with the interview.

M: Ok. We’ll begin by looking at the positive and negative aspects about pigs. Do you think pigs are important in these villages?

Res: Pigs are very important in these villages because they are; they are like the easiest animal that people can slaughter in case of a funeral, in case of a wedding ceremony, in case of the traditional functions (relish), I would say, it’s very important.

M: Very important, ok. Anything more, apart from the funerals, and weddings, and relish and...

Res: School fees.

M: School fees? They use that for school fees as well?

Res: Yes, they use it for school fees as well. They sell the meat to raise some funds to pay for their children’s school fees.

M: Ok. Those are the positive aspects. Are there any negative aspects about keeping pigs in the villages?

Res: Ok, the way they keep the pigs, they keep them on free range.

M: On free range, yes.

Res: Yes, that’s their main problem and the reasons why is that they say they can’t manage to keep them in houses, that is, in pig houses. They say they can’t afford to feed them throughout the year.

M: Ok, ok.

Res: That’s the problem which they have.

M: So they would rather leave them on free range so that they scavenge?

Res: So they scout on their own, yes.

M: So for you what are the positive aspects about pigs? For you, that was about the villagers, now for you, you think there are any positive aspects about pigs?

Res: Ok, the positive aspect is that eeuuu! Pigs when they are kept like in house, production is very high.

M: Very high, yes.

Res: There is good profit at least they produce a good number of babies I would say. (*Laughs*)

M: They are prolific? (*Smiles*)

Res: Ya, ya, ya, they produce a good number of litter eeeh! So they are ahhh! (Laughs) what can I say?

M: They are a good, what can I say ehh! They are eeh! A good return in someone’s investment?

Res: Yes they are a good return in someone’s investment, when you are rearing pigs at least you are assured of having twice the number of litter that you are going to produce and you are going to have enough money to raise for the problems you are encountering in a home and when you leave them on free range...

M: Ya.

Res: ...there are higher chances of them being attacked by African swine fever. That’s the major disease that we have in this place.

M: Any, any negative aspects?

Res: As in?

M: We have talked about the positive aspects, that when someone keeps them nicely then they’ll have a number of them within a short period of time. Any, any negative aspects according to you?

Res: About when they are kept in house?

M: About the pig in general.

Res: The negative aspect that I know is, on the preparation of the meat itself....

M: Ok.

Res: … Because usually the farmers would just want to like ehh! *kushoka*

kushoka = roasting

M: Roasted?

Res: Yes, they would rather roast it.

M: Than have it properly cooked?

Res: Yes, and roasting it is a problem because that meat is not ok...

M: Not ok!

Res: … Since they scavenge, they pick a lot of things as they scavenge and they are found with worms and then people would just roast it for one second and eat. But I would recommend that if they want to eat that meat they should at least boil it for one hour...

M: Ok.

Res: … yes, before they fry it.

M: Are there any aspects about the pig that would make it a dangerous animal to people?

S*ilence*

M: Anything that you can think of that would mean that the pig is a dangerous animal to people?

Res: It’s not dangerous.

M: It’s not dangerous, ok. You have already talked about why villagers raise pigs eehh! Mostly for helping in the family when in need of money, ok. How do you think pigs are fed in the villages?

Res: How they are fed?

M: How they are fed, yes. Are they fed at all?

Res: Actually they are not fed.

M: They are not fed!

Res: Yes. The only time that maybe you can say they are feeding them, maybe it could be one or two farmers, not, not the majority.

M: Not the majority.

Res: Yes, maybe one or two farmers when they harvest, they would like after milling their maize meal they take the maize bran and give it once in a while.

M: And that’s not by everybody?

Res: No it’s not everybody who does that.

M: So pigs would feed on a lot of other things...

Res: Yes.

M: ...if they are not fed?

Res: eehh eh!

M: Its common knowledge here that pigs feed on human faeces?

Res: Yes they do.

M: Do you see any danger in pigs feeding on faeces?

Res: On the health of the pigs themselves?

M: Yes, yes.

*Silence*

M: Is there any danger?

Res: There is no danger I would say.

M: Ok. Ahh! Do you eat pig, pork meat yourself?

Res: Yes I do.

M: When do you normally eat the meat?

Res: Yes I do.

M: Ok. When do you often consume the pork?

Res: Ok, me I know the preparation for it...

M: Ok.

Res: … Not to bring an effect to me, so even if I buy it for my family I make sure it is at least boiled for one hour, yes.

M: Ok. Are there special occasions when you eat pork or you just eat it on an everyday basis?

Res: No, there isn’t.

M: Ok. Where do you get this pork meat from?

Res: We buy it from the butcheries.

M: You buy it from the butcheries, from the Boma?

The Boma = the centre of Petauke town

Res: Yes.

M: Ok. Is there any special attention you pay, you pay or before you buy the meat is there anything you look out for in the meat?

Res: When we buy it from the butchery we take it ehhh. There is someone from the Heath department (from the District council) we believe checks the meat. Yes, so when it’s bought, we just prepare it.

M: So before you buy it from the butchery you have that conviction that someone from health has looked at it.

Res: Yes.

M: So you have no special things that you look for, yourself, in the meat?

Res: No, no (*Laughs*).

M: Ok, ok. Do you ever eat meat that is clearly infected with cysts?

*Silence*

M: You know cysts ahh? They call it *nsembe* here.

Res: Mmmmm, I have seen them, yes.

M: You have seen them, yes!

Res: Yes.

M: Do you buy meat or consume meat or eat meat that is clearly (*with emphasis*) infected with the cysts?

Res: No we (family) don’t.

M: Ok. Ahhh, according to you what is the main risk behaviour performed by pig owners in this region?

*Silence*

Res: The most risk?

M: The, the thing, the way they raise their pigs. What is the most ah ah ah, the most, what is the...about the keeping of pigs what would be of great risk to people’s health or to pigs’ health?

Res: The way they keep them?

M: Yes, the way they keep them.

Res: The security of the pigs is not there, that is the side of the pigs, they can be stolen at any time. They mix (with other pigs) at any time, they are caught by wild animals at any time...

M: So the free range kind of husbandry!

Res: … Ah ah, it’s not safe for the pigs themselves. And ah, on the side of the humans themselves, I think it’s the preparation which they, they usually don’t follow.

M: Don’t follow!

Res: Mmmmmmm.

M: What are the main health issues related to pigs? What are the main health issues that you are called to attend with regards to pigs?

*Silence*

M: Do people call you often that “my pig is sick or our pigs are sick”?

Res: I would say the people don’t care about their pigs...

M: They don’t care!

Res: … They just raise them; eat them when the time to eat then comes. They just come across a case, not that they can come and call you (the vet). If they call you it means they have, they are keeping these, what...

M: Cows?

Res: ...no, the what, what can I say? The improved pigs.

The improved pigs = the pigs raise in doors by wealthy farmers

M: Ah, ok.

Res: The cross breeds

The cross breeds = the cross between large white and landrace

M: The cross breeds?

Res: Yes, so they manage, those that keep these they manage to keep them in-house...

M: When they have a problem, they call you?

Res: ... So those are the only ones that can call you when they have a problem. Those that keep on free range, they don’t consider us important.

M: So the ones that keep in houses and call you, what are the main problems that you find with their pigs?

Res: They usually have “Mange”...

Mange = pig skin disease

M: Mange, yes.

Res: Yes, when they have mange.

M: How do you reduce these health related issues with pigs, if someone calls you and you see the pig has mange, how do you manage that?

Res: We treat them.

M: With?

Res: I treat with Ivermectin.

M: Ivermectin.

Res: Yes.

M: Have you ever observed proglottids in human faeces?

Res: What?

M: Proglottids, these…

Mod shows and gives pictures of human faeces with proglottids

M: You know a tapeworm? You have heard of a tapeworm?

Res: Ahhh, yes I have heard of a tapeworm.

M: So a tapeworm has a number of proglottids and usually if someone has the proglottids, they will pass them in the stool. Have you ever observed that?

*Silence*

M: Like in children or anyone else.

Res: No I haven’t. (*Shakes head*).

M: Would you know what those are? (*Pointing on the picture*)

Res: Mmmmmm, no (*shakes head*).

M: Having looked at them, would you know what they are?

*Silence and Res goes through the pictures*

Res: They are all the same?

M: That is from someone’s stool where we found those proglottids.

*Silence*

M: You’ve mmmmm, do you know about taeniosis, tapeworm infection in humans?

Res: I don’t know.

*Silence*

M: But you have heard about common helminths in humans?

Res: Mmmmmm, (*nods head to signify a yes*).

M: Ok. Ehhh, do you know of any symptoms that are related with general helminthiasis?

*Silence*

M: When children have worms...

Res: In humans?

M: ...yes, what would be the symptoms?

Res: It’s just a big tummy. (*Laughs)*

M: A big tummy (*laughs with respondent*). Just a big tummy, nothing more?

Res: Mmmmm (*nods head*)....Some weight loss with a big tummy.

M: Do, ehh, do you know where these worms come from? How can someone get worms?

Res: Adults get them from pigs, some eat soil. Some get it from the soil; some get it from the pigs.

M: Eeeh mmmmmm, and children?

Res: Children get it from the soil...

M: From the soil!

Res: Yes

M: Do you think such diseases, like having worms, can be prevented?

Res: As in by not deworming?

M: Are there ways that you know of that we can use to prevent people from having worms?

Res: I think on the side of adults that don’t eat the soil...

M: Don’t eat the soil!

Res: We just have to teach them how to prepare ah ah, the meat.

M: Can worms be treated, you think?

Res: Yes. (*Giggling)*

M: They can be.

Res: Yes.

M: Witch doctor? At the hospital or at a witch doctor’s ‘clinic’?

Both moderator and responder laugh softly.

Res: Worms can be treated at the hospital.

M: Do you know of any disease that you think is more severe than worms?

*Silence*

M: Are there any diseases that can be considered to be more severe than worms?

Res: Mmmmmm! Malaria...

M: Malaria. Just malaria?

Res: And TB.

TB = Tuberculosis

M: And TB, ok.

Res: Yes! (*Nods head*).

M: Any disease that you think is less severe, in theses villages? As compared to worms?

Res: I think its flu (Common cold).

Flu = Common cold

M: Flu is less severe than worms.

Res: Yes mmmmmm!

M: Do people in ....You have interacted with a lot of people in these villages?

Res: Mmmmmm! (*nods head*).

M: Do, a lot of them suffer from worms? You think.

Res: Mostly I think the children.

M: The children ok. Do you think being infected with worms is a big health problem or is a serious health problem for someone?

Res: For the children, yes.

M: For the children, ok. For the adults, no?

Res: For the adults, no, I don’t think so.

M: Do adults also get big tummies?

Res: No they don’t.

M: So they don’t get to know that they have worms?

Res: They just see them.

M: When they....

Res: By chance.

M: By chance! When they pass stool?

Res: Yes.

M: Are there any consequences that you might think of that could be related to being infected with a worm?

*Silence*

M: If someone has a worm, any consequences to their health?

Res: If they are heavily infested with worms, I think they can be anaemic.

M: Anaemic, ok.

Res: Yes.

M: Ok. Ahhh, are there any impacts that being infected with worms can have on one’s daily life?

*Silence*

M: Like if I have a tapeworm or worms, are there any things that I could not be doing because of the worms?

*Silence*

M: Can I go on with my normal daily life no problems, even if I have worms?

Res: That is, that would depend up on the number of worms that are in you.

M: So if I have a lot?

Res: If you have a lot of worms, then definitely they’ll have an effect, because they feed on what you are eating and they destroy what is inside.

M: So what’s that effect?

*Silence then laughs*

M: How will that affect my daily life? Say this man grows a lot crops, he plough a big piece of land, would that affect him, I mean having worms?

Res: I think there can be an effect.

M: Ok.

Res: Yes.

M: You have heard of cysticercosis, right?

Res: Mmmmm (*nods head*).

M: The disease cysticercosis. Do you think it’s an animal disease or a human disease?

*Silence*

Res: Cysticercosis! *(Mumbling)*

M: The cysts, ahh!

Moderator shows picture with cysts

M: You have seen those, right?

Res: I have seen them in pigs.

M: In pigs yes. Do you think that’s purely a pig disease or it could be a human disease?

Res: I think it’s a pig disease because I have seen it in pigs.

M: It’s a pig... you have never seen it in humans?

Res: No (*shakes head*).

M: Do you know that it can occur in humans?

Res: No.

M: You have heard of epilepsy...

Res: Yes.

M: ...or “*Khunyu*”.

Khunyu = local term for epilepsy

Res: Yes.

M: What are the symptoms of *Khunyu*?

Res: I think it’s the convulsions and saliva that comes out from the mouth and the eyes will show to be upside down.

M: Is it an animal disease or a human disease?

Res: Epilepsy?

M: Epilepsy, yes.

Res: It’s a human disease.

M: It’s a human disease, ok. Do you know of anyone infected or anyone who has epilepsy?

Res: There is someone I know yes.

M: How do you think they got infected? Or how do you think someone can get infected or can get epilepsy.

Res: From what I gathered, the owner of the child said she just got a temperature (fever) and they started treating for malaria but the problem still continued.

M: Even after the malaria was treated?

Res: Yes.

M: So the only way you think you know of that someone can get epilepsy is from malaria?

Res: It’s from malaria, yes.

M: Can this disease, epilepsy, be prevented? You think.

Res: No, I don’t think so.

M: Can it be cured?

Res: No (*laughs*).

M: It can’t be cured! Why not? Why do you think it can’t be cured?

Res: Because the cases I have heard of, they have tried this traditional and hospital and they haven’t been cured, they are still sick, the two of them.

M: The two that you know, are they adults?

Res: No they are children

M: Are you afraid of getting epilepsy yourself?

Res: I don’t know how it can hurt me but I wouldn’t want to suffer from it. (*Laughs*)

M: So you are afraid of it?

Res: Yes.

M: Why?

Res: Mmmmmm?

M: Why would you be afraid of it?

Res: Because there are so many accidents that they encounter.

M: So do you think that in your daily activities as a Veterinary Assistant, are you exposed to the possibility that you can get it?

Res: I don’t know how I can get it and that’s why I can’t know if it’s possible that I can get it.

M: Do you know of or can you mention a disease that you think is more severe than epilepsy?

*Silence*

M: According to you is there any disease that you think is more severe as compared to epilepsy?

Res: A disease which is more severe that epilepsy!

M: Yes.

*Silence*

Res: Its only TB

M: Only TB, ok. So epilepsy would be more severe than malaria? You think.

Res: Epilepsy is more severe yes.

M: More severe. Any disease that you think is less severe than epilespy?

*Silence*

M: Than *Khunyu*?

Res: *(Laughing)* Plenty

M: Plenty of them? Like?

Res: Come again?

M: Like which ones?

Res: We have....Is headache a disease?

M: It’s an illness, so...

Res: Abdominal pains.

M: So those are less severe?

Res: Yes they are.

M: Can epilepsy be prevented?

Res: That one I don’t know.

M: So you don’t know if it can be cured either!

Res: Yes.

M: Do a lot of people suffer from it in the villages where you have been?

Res: No. *(Shaking head)*

M: They have never told you that, “in this village we have a number of people with epilepsy or *khunyu*?

Res: They would say that there is only one person in the whole village.

M: The whole village!

Res: If they are many they should be just two.

M: Ok.

Res: It’s not very common.

M: Do you think being infected with this disease is a serious health problem?

*Silence*

M: Being infected with epilepsy or someone having epilepsy is a serious health problem?

Res: It’s a very very serious health problem.

M: Why?

Res: Because they can get burnt.

M: They can get burnt?

Res: Mmmmmmm.

M: Are there any other consequences that people can have because of epilepsy apart from getting burnt?

Res: They can get drowned...

M: They can drown, yes.

Res: ...they can be hit by cars.

M: Ok.

Res: Yes

M: Do they marry?

*Silence*

M: People with epilepsy?

Res: Mostly the marriages fail.

M: The marriages fail, why?

Res: The moment the....ok, they can marry before that person knows the condition of the other partner, but the moment that (seizures) happens in the house, that’s the end of the marriage.

M: And if someone has or someone already shows that they have epilepsy, can they get married? Before they even get married?

Res: I don’t think so.

M: Are they stigmatised? People with epilepsy?

Res: They are, yes. I have met one, she was just complaining to me that she has epilepsy, she was married, and then when it happened, the husband left her and left her with a baby and she is living with her mother now.

M: Ok. So are they kept at a distance by the community, like no hand shaking you know general stigma?

Res: Generally it’s not there but when a person feats, people will like scatter...

M: Ahhh!

Res: ...and maybe try to avoid so much being with that person in case that happens again.

M: So does the disease have any impact on their daily life?

Res: It has. From the person that I saw it has because she is not happy.

M: So she can’t do a lot of things freely?

Res: No she can’t.

M: Ok. Ahhhh! Imagine that we propose different control measures for taeniosis and cysticercosis, both in humans and in pigs, taeniosis in humans and cysticercosis in both pigs and humans. According to you, ok, which main barriers do you think applying these, which are the main barriers that would appear if we applied these control measures and why? If we came and said everybody in the villages confine their pigs, build houses and fences and keep their pigs in fences, are there any barriers or any problems that we could encounter from the villagers?

Res: They will tell you we don’t have food to give the pigs so how are they going to survive in the houses.

M: So they wouldn’t grasp such a control measure?

Res: They would make that thing work, but for how long. The moment they lack something (feed) to put in there, they release them.

M: Release them!

Res: Yes, meaning it won’t be a long term programme.

M: Won’t be a long term programme! So as long as they don’t have the feed they release them.

Res: They release them.

M: So that would be a big barrier ehh!, feeding.

Res: Yes that would be a big barrier.

M: What if we came and said every household should have a pit latrine? Would we meet any barriers? Or are there any problems that we would encounter?

Res: Nowadays it has changed (*laughs*). It’s like they consider pit latrines useful.

M: They consider them useful?

Res: Yes.

M: But do you think they consider human stool as feed for pigs?

Res: They don’t.

M: They don’t.

Res: Mmmmm! They just go to the bush because they don’t put making a pit latrine as important, because the house has first priority so they would rather go in the bush.

M: Why do you think they would rather go to the bush?

Res: … *(Laughs)*.

M: I mean, it’s easier to put up a pit latrine and easier to do everything in an enclosure than going to the bush, you don’t know who is going to see you.

Res: You see, they don’t want to be seen going into the toilet (*laughs*). So they could rather disappear and come back.

M: ...and come back.

Res: ...Yes, than be seen going to the toilet maybe 5 minutes there and come out.

M: So that would be a barrier, it would still be a problem?

Res: No it can’t be a problem because I think they now understand why toilets should be there.

M: Ok. So we would need a lot of education campaigns so that we educate them further on the importance of toilets?

Res: … Toilets yes (*nods head*).

M: What if we came and said that all those that are eating pork should boil it? Would there be any problems? You think?

Res: … *(Laughs).* The problem would be adopting (this cooking method) because they think they are wasting time by boiling it. Its soft meat and then they boil it for one hour when they believe in just put it for 5 minutes and it’s ready.

M: So it would be difficult changing their mindsets?

Res: Not with, not with reasons, I think they can change. You give them the actual reasons

M: Do you think in the households, ehhh! Boiling meat is not practised?

*Silence*

M: Like, by women?

Res: They dot boil it with water, they just wash it and put it in the pot and when that water finishes they fry and eat and it’s not enough (the water) for one hour, it can’t.

M: What if we came and said, ahhh, anyone who slaughters a pig, before they eat the meat it should be inspected?

*Silence*

M: Would we face any problems? Or would we encounter any problems?

*Silence*

M: You have worked with them...

Res: Yes.

M: Do they call you to inspect the meat before they eat?

Res: The health personnel, that’s the person they use, they don’t use me.

M: And he inspects the meat?

Res: She inspects the meat, yes, she is a lady also.

M: So they can be receptive to such a control measure, you think?

Res: Those that sell at an open place wouldn’t be because they sneak around.

M: Because ahhhh, the slaughtering of pigs is very common in people’s backyards...

Res: Yes.

M: ... They just tell their children, bring that pig we slaughter.

Res: I know, and after slaughtering, if it’s for the family they just eat it without calling anybody, and if they want to sell it like in the village they will just go round in the village selling the meat.

M: So would we face a problem if we put a rule and said everyone that slaughters a pig lets have the meat inspected, you think that would be a problem?

Res: I don’t know, I can’t be very sure.

M: Why do you think some people don’t call the meat inspector to inspect the meat?

Res: Why they don’t call us? (*Laughs*).

M: Why they don’t call you yes.

Res: Because they are scared we can condemn the meat and when we condemn sometimes we condemn totally, sometime we condemn half, ahhhha.

M: So for meat that is like that?

Mod points at the picture of cyst laden meat.

Res: It has to be condemned totally.

M: So do they take kindly to that condemnation, because after condemning it has to be destroyed right?

Res: And we have to dispose it with them, you burn it with them.

M: Do they take kindly to that?

Res: They don’t.

M: They don’t?

Res: Mmmmmm, (*nods head*).

M: So that would be a big barrier?

Res: But it’s supposed to be done (*laughs*). Yes it’s supposed to be done whether they don’t want it or not.

M: You know there are some worms that are passed through vegetables? The non-washing of vegetables before they cook?

*Silence*

M: You know that ahh?

Res: Raw vegetables maybe, not when they are cooked.

M: If we came in the villages and said anyone cooking vegetables they should wash them before cooking.

Res: If you cut them and wash them then you cook can they have worms?

M: Yes it’s possible.

Res: Then I don’t know.

M: Would people take kindly to such a rule?

Res: … *(Laughs)*

M: Would people agree to such a regulation that all vegetables should be washed?

Res: They wash.

M: They wash?

Res: Yes.

M: Do they wash “*chibwabwa*”?

Chibwabwa = Pumpkin leaves

Res: Ahhh!

M: *Chibwabwa*, do they wash it?

Res: *(Laughs).* *Chibwabwa*, they...........

M: Fresh *chibwabwa*?

Res: ...C*hibwabwa*, some wash others don’t.

M: Ok, so they will take this rule seriously and their vegetables?

Res: They can wash, yes.

M: Ok. What if we came and said, we demand good body hygiene, people should be bathing and putting on clean clothes so we minimise on a lot of diseases not just worms?

Res: Mmmmm!

M: Would it work?

Res: It can’t… *(Laughs)*.

M: Why?

Res: Because it’s temporal, after harvesting they have money then they use that money, when that money gets finished then they are finished.

M: Then they don’t bath?

Res: They bath but it’s not as regular as they would bath when they have everything.

M: And this “everything” means?

Res: When they can buy soap for washing and bathing soap.

M: So the bathing, the good body hygiene is seasonal.

Res: It’s after harvesting, that’s when you would find that most of them look smart and after that time, then they start changing bit by bit. Then when they start cultivation, it’s something else.

Mod laughs together with respondent.

M: What if we brought a regulation that everybody should be treated for tapeworms? Everybody should go to the health centre and get medicine for tapeworms.

Res: I think they can come.

M: They can come; you think we would encounter any problems, barriers?

Res: No.

M: Are people receptive to these mass treatments for anything in the villages?

Res: Yes they are.

M: Ok, they wouldn’t say “no me am not even sick why should I go”.

Res: *(Laughs)*. No they can’t.

M: What if we brought a vaccine for the cysts...

Res: For the humans?

M: No for pigs. We want to treat them and carry out vaccination campaigns for cysticercosis, do you think people would be receptive to such a control measure?

*Silence*

M: Would they bring their pigs for vaccination?

Res: Not all of them.

M: Why?

Res: Because they don’t consider pigs being taken to Veterinarians that much unless we sensitize them on the importance bringing their pigs to us, mmmmm, but the little that we (Vet Department) have done, the response is not so good, like in cattle.

M: Ok. If we, say, brought this vaccine and said brig your pigs but you are supposed to pay a small fee, would that work?

Res: Mmmmm, that’s the problem we have on the cattle (*laughs*). Because they are paying a K15,000 ($3) for each animal, so some would stay back, some will pay, just like that.

M: Others wouldn’t?

Res: Yes, other wouldn’t.

M: Are the ones paying the majority or the ones that are not paying are in the big majority?

Res: Those that are paying are in the big majority because we have done it for quite some time now so they have understood now that the vaccine is important...

M: Important!

Res: ...Because where the animals are dying, those that were vaccinated you find that the animals are still there, those that were not are all gone...

M: They are dying?

Res: ...like its working on this one.

M: So that’s for cattle...

Res: Yes.

M: it seems to be working relatively well...

Res: Yes

M: so you don’t see it working as well for pigs?

Res: It can work.

M: It can work? With? How would it work?

Res: The same way that we are trying to make them work with cattle.

M: With cattle.....like with sensitisation? Ok. Thank you, so in our discussion we’ve looked at the positive and negative aspects of pigs, how people recognize their pigs and what problems they encounter, we’ve looked at the risks associated with pigs and we also talked about how much you know about the disease taeniosis and cysticercosis and also the possible control measures that we would adopt and the possible barriers that we would encounter with regards to controlling this diseases in pigs and humans. Unless you have any questions.

Res: I just thank you for giving me this opportunity to talk to you.

M: Thank you very much.

Res: You have made my birthday (*Laughs*).

M: Ahhh! Ok, it your birthday today?

Res: Yes.

M: Happy birthday then and thank you very much.
