# Supplementary material for: Why Latrines Are Not Used: Communities’ Perceptions and Practices Regarding Latrines in a Taenia solium Endemic Rural Area in Eastern Zambia
Source: PLoS Negl Trop Dis. 2015 Mar 4;9(3):e0003570. doi: 10.1371/journal.pntd.0003570 (PMC4352092; doi:10.1371/journal.pntd.0003570)
Supplement: S1 Dataset — (ZIP) [file pntd.0003570.s001.zip › FGD transcriptions_Zambia-2010/Men_pretest_29-07-10.doc]

**Title : Focus Group MEN (pre-test)**

**Date : 29/07/10**

**Site : Kakiwa Rural Health Center**

**Village : multiple	Location : Petauke district, Eastern Province, Zambia**

**Duration (total time) :	- Start :	- End :**

**Participants : 6 men**

**Informed consent : verbal agreement**

**Moderator : Mr Benjamin Mvula	Recorder : Dr Andrew Phiri**

**Transcription/translation :**

**Abreviations :**

**Mod : moderator, R : recorder, Intervenant : I, C : children, W : women, M : men**

**Disposition (from the left of the Moderator):**

**M**

M1

M2

M3

M4

M5

M6

**R**

Age composition:

M2: the youngest

M6: the oldest

**Comments about participants :**

Even they belong to different age category, all of them have the same statut. All of them are headmen.

**Group dynamics :**

**Text:**

**Mod :**
