# Supplementary material for: Why Latrines Are Not Used: Communities’ Perceptions and Practices Regarding Latrines in a Taenia solium Endemic Rural Area in Eastern Zambia
Source: PLoS Negl Trop Dis. 2015 Mar 4;9(3):e0003570. doi: 10.1371/journal.pntd.0003570 (PMC4352092; doi:10.1371/journal.pntd.0003570)
Supplement: S1 Dataset — (ZIP) [file pntd.0003570.s001.zip › FGD transcriptions_Zambia-2010/Mkopeka_Mr Sakala/Children_Mkopeka_03-08-10.doc]

**Title : Focus Group CHILDREN**

**Date :** 03/08/10

**Site :** Kakiwa Rural Health Center

**Village :** MKOPEKA	**Location :** Petauke district, Eastern Province, Zambia

**Duration (total time) : 44 min (video 22 min)	- Start :** 10:56	**- End :** 11:40

**Participants:** 9 children

**Informed consent:** signed and available

**Moderator :** Mr Benjamin Mvula	**Recorder :** Dr Andrew Phiri

**Transcription/translation :** Dr Sakala

**Abreviations : M: moderator, R: recorder, I: intervener, C: children, W: women, M: men**

**Disposition (from the left of the Moderator):**

**M**

C1

C2

C3

C4

C5

C6

C7

C8

**R**

C9

Gender distribution:

**C1-C2-C3-C8**: females

C4-C5-C6-C7-C9: males

Age composition:

C4-C5-C6-C7: 18yrs

Typology:

C1: shy

C9: dominant/expert

C3: dominant among girls

**M**

M1

M2

M3

M4

M5

M6

**R**

M7

M8

**Comments about participants :**

**Group dynamics :**

**Text:**

*Introduction*:

M: I want us to discuss on the rearing of pigs, so what I will be happening is that I will be asking you questions and all of you should be free to answer, I do not want to discuss with one person but all of you should participate. First of I want us to know each other.

*All children introduce themselves.*

*Discussion*:

M: Like I have already introduced the subject we are discussing about pigs. What is good about pig rearing?

C2: It is slaughtering them during a funeral.

C4: The other good is that for us who go to school, a pig can be so that we pay our school fees.

C8: In addition you can sale a pig and hire some people to help with the field work.

C7: You can slaughter a pig for your relish at home.

C6: I was making the same contribution as the last speaker.

M: What is badness about a pig?

C4: Pigs eat human feces.

M: Anymore?

C2: When it eats feces it comes to eat from kitchen utensils.

M: C3?

C3: When swine fever strikes they all die.

M: Anymore?

C8: They are not strong when the fall sick.

C7: A pig is a carrier of germs.

M: Who can add what C7 has said?

C6: The disease can be contracted by people.

M: How?

C6: If you eat the meat.

M: Do we rear pigs in our villages?

All: Yes.

M: Why do our parents keep pigs?

C5: To help them.

M: In what way?

C5: If they have a problem.

M: Others?

C3: Help as for relish when they slaughter it.

C6: They help send children to go to school, if they sale one pig they would have enough money to send children to school.

M: Anymore?

C3: When there is a funeral they would slaughter a pig to assist feeding people.

M: Anyone?

C2: Use the money realised from sale of a pig to help weeding in the field.

M: Anyone else?

*Silence*

M: How do we keep our pigs, do we keep them in the kraal?

C2: We keep them in the Kraal.

C9: Some are just kept outside.

C3: Our parents do not build kraals so some pigs are just kept outside.

M: What is good about keeping pigs outside?

C4: There is nothing good.

M: Why is it bad?

C3: Some of us do not have toilets, we go and shit in the bush and the pigs which just roam about would feed on the feces and in turn eat from kitchen utensils.

C9: When they come back to the village the same pig would feed from our utensils.

M: Any others?

*Silence*

M: C1?

C1: Some do not have toilets and the pigs would feed on the feces.

M: Anymore?

C5: The pigs would wonder into people’s fields and eat their crops and this would bring problems to the owner of the pigs.

M: What is bad about leaving the pigs roaming about?

C9: They would contract diseases.

M: What type of diseases?

C9: Like swine fever.

C7: Also like diarrhoea.

M: Any other?

*Silence*

M: How do we keep these pigs?

C7: We need to build them kraals.

M: How do we keep them?

C9: We give them food.

M: Anyone else?

*Silence*

M: Since these pigs are kept outside how do we know that these are our pigs?

C3: Some cut a piece of the ear lobe.

M: Anymore, C8?

C8: Like she has said I only know the ear mark.

C2: Some cut off a bit of the tail.

M: Anymore?

*Silence*

M: What problems do we meet in the rearing of our pigs?

C2: Some fall sick after eating pork.

M: We are talking about the problems you meet in the rearing of pigs?

C3: The problems we face are that the pigs would go and feed in the nearby people’s fields and therefore bring us problems to pay for the damages they would have caused.

M: Anything more?

C7: We are told that a pig carries some germs, and after eating the meat you would get sick.

M: Anymore?

C3: When they fall sick we do not know the medicine to be used in treating them.

M: What problems do we meet in the rearing of pigs?

C4: Sometimes they have boils on their bodies.

C8: Sometimes they have *nsembe*.

M: Anyone else?

*Silence*

M: Now, when we meet these problems is there anyone who helps us?

C8: Nobody.

M: How do we know that this pig is sick?

C4: You would notice that it is not very active and even when you give it food it would not eat.

C8: It would loose weight.

M: Anyone?

C7: It does not feed well.

C9: It would be foaming from the mouth.

M: Anymore?

C3: Sometimes it would not be able to move because the legs would be paralysed.

C2: I wanted to give the same answer.

M: What do we feed the pigs?

C4: Maize bran and salt.

C8: Pumpkins.

C1: Maize bran.

M: C2?

C2: Maize bran.

M: Anymore?

C7: They mix fertiliser with the bran so that it can help in their growth.

M: Anyone else?

I (Dr. Mwape): What type of fertiliser do they mix with the bran, the white one?

C6: Yes, the white one (urea).

M: Who feeds the pigs at your homes?

C9: In our home I am the one who feeds them.

M: C2?

C2: They are fed when they feel hungry.

M: Who gives them the feed?

C3: Anyone else who is at home can feed them when they notice that they are hungry.

M: Can your parents feed them?

All: Yes because if you have gone to school and they are at home they would give them feed.

C6: Yes because if I go to school my parents should feed them.

M: The food that we feed the pigs where does it come from, is it our own or it comes from somewhere?

C9: We collect from the maize mill.

M: Do we all manage to collect from the maize mill?

All: Some just pound their own maize.

M: So there is no other supply of the feed we just feed them our own feed?

All: Yes.

M: Do you take a role in the rearing of pigs in your homes?

C9: They are helpful also to us.

C4: Like we have already said the pigs assist us in the payment of school fees.

M: I am saying what role do you play in the rearing of pigs?

C3: We give them feed.

C4: We also pen them in for the night.

M: What is your job in the rearing of pigs?

C6: Build them a kraal and give them feed.

M: Anymore?

C3: Pen them in for the night.

M: Are you happy to do those jobs?

All: Yes.

M: Why?

All: Because they help us and they are ours.

M: Apart from your family members in you home, are there any other people who help in the rearing of pigs?

All: None.

M: There is nobody except the people at our homes?

All: Yes.

M: Is there any where, where we collect anything to help us rear the pigs?

All: Nowhere.

M: So, everything that goes into the rearing of pigs comes from us?

All: Yes.

M: Who make a decision that this pig should be slaughtered today?

All: Our Parents

M: You do not take any part in the decision making?

C4: We can not make that decision, but we can suggest.

C9: But if our parents say no then we can not go ahead.

M: So we cannot say anything?

C4: We can say something, we can ask that we would like to slaughter a pig but if they do not allow us according to what we are asking for then we cannot proceed.

M: What if these pigs belong to you?

C3: Yes you still have to ask.

C9: Yes, you have to ask them and if they say no then they would suggest an alternative.

I (Dr. Mwape): What if you did not have books and you asked your father that can we slaughter a pig I would like to buy books. Do they agree?

C9: Yes they agree.

M: How many times do we feed our pigs per day?

C6: Three times

M: C9?

C9: Once.

C3: Twice.

C4: There are those which stay in the kraal day and night, these are fed three times a day.

M: What is good about feeding the animals?

C8: So that they can get fat.

M: Anyone else?

C4: So that they are not kept hungry otherwise they would die.

C3: They should not be kept hungry because if they are kept hungry they would start eating feces.

M: What is good about giving them feed?

*Silence*

M: What is bad then of giving them feed?

C4: There is nothing bad.

I (Dr. Mwape): In your village, are there any people who keep their pigs in the kraal day and night and do not let them out?

C9: Most people keep them in the kraal at night then let them out in the morning.

I (Dr. Mwape): Do they manage to feed them three times a day?

C9: They feed them only once in the day.

C5: They are fed twice when letting them out in the morning and when locking the up for the night.

M: Anyone else?

C3: They are fed three times in the morning, mid day and in the evening.

C6: The ones which are fed three times mostly are those which are not let out.

M: Do we regularly eat pork?

All: Yes.

I (Dr. Mwape): You do not eat?

All: No we eat, it is good meat.

I (Dr. Mwape): Who does not eat pork?

All: There is may be one.

C3: There are two of us.

M: When do you slaughter pigs?

C9: During Christmas.

M: Anyone else?

C6: Some slaughter pigs for business.

M: Any other time?

C9: During Independence Day.

C5: If you receive visitors.

C3: During a funeral.

C6: At a wedding.

C3: When you do not have money.

C1: During the ploughing season.

M: Is the meat examined?

All: No.

M: Would you be happy if pork was examined?

All: Yes.

M: Why?

C6: We would like know which ones have a disease and which ones are fit for human consumption.

M: How about others?

C9: When the pig has *nsembe* and was examined and they told us that it has a disease and should be thrown away then we would throw it away.

M: Would you manage to come and see the person to examine the meat?

All: Yes we would be managing.

M: Do you know the person who can examine the meat for you?

All: We do not know him.

M: If you knew him would you becoming to tell him?

All: Yes.

I (Dr. Mwape): What if the person came and examined one of your biggest pigs, that it had a disease and recommended that the meat should be thrown away, can you agree?

All: Yes, because he has examined it and found the meat not suitable for human consumption.

C3: But we still feel bad.

C4: Even when we are throwing it away we would feel bad.

*Laughter*

C6: Some times we can go and sale it else where.

M: A diseased animal?

C5: Well sometimes it happens that way.

M: Now we are talking about ourselves?

C6: Those who would be passing by and who would not have known what transpired.

*Laughter*

I (Dr. Mwape): What about when it is sick, what do you do with it?

C9: We slaughter it.

I (Dr. Mwape): Do you eat the meat.

C9: Yes we eat the meat, without knowing what it was suffering from. Even if it died own its own people just eat.

M: Which type of cooking do you like boiled or roasted, then why?

C4, C5: Boiled.

M: Why?

C5: Because it is thoroughly cooked.

M: How about roasted?

C4: We do not like roasted meat.

C8: Sometimes people like to buy already roasted meat because may be there is no relish at home so that when they reach home they just start cooking *nshima*.

M: Why do you not like roasted meat?

*Silence*

M: How do your mothers cook pork?

*Silence*

I (Dr. Mwape): How do your mothers cook pork at home?

C9: She cuts the meat up put it on a fire and thoroughly boils it and then takes it off.

M: Anyone else?

C7: It feels good.

*Laughter*

C9: You wash it and put it on a fire.

M: They do not roast some of it?

C5: They roast.

M: Roast some for your dad?

C9: They roast.

M: Now why are you not saying so?

C9: Now it depends on the person some would like to roast and some would like cooked.

End of the video recording

*Notes from the recorder:*

M: Have you ever seen *nsembe* in pigs or pork meat?

Picture showing pork meat laden with cysts is passed around.

All: Yes we have seen them

M: Do you know what they are?

C3: Some say that when you feed pigs maize bran then they can have the *nsembe*.

M: What do you do with such meat?

C9: Some people dry it if it has *nsembe*.

M: Do you or have you eaten such meat before?

C3, C4: Yes we have eaten such meat before.

M: Do we have toilets at our homes?

C5: No we don’t have.

C8: Some people have while others don’t have. And some toilets don’t have a door so it’s difficult to use them. Others don’t use toilets because they don’t want to wash hands and so they go to the bush.

M: You do not wash hands after using the toilet?

C9: Sometimes we just forget.

*Laughter*

C4: Those that go to the bush don’t wash hands.

C9: ....and many people use the bush.

M: Why do many people go to the bush?

C3: When we grow older, we are prevented from using the same toilets together with our parents, so we go to the bush.

M: But why not just build another toilet?

C5: We fail to have two toilets at home so we go to the bush.

M: And you don’t meet in the bush with other people?

C9: When we meet in the bush it’s ok, it’s better than meeting in the toilet where you can find your father or mother with their pants down.

*Laughter*

M: Do you wash hands before eating anything?

All: We wash hands before eating *nshima* but we don’t wash hands before eating groundnuts or fresh maize.

M: Have you seen worms before?

*Silence*

Picture of human stool with tapeworms is passed around.

C9: I have seen them but they were round worms.

*Others say they have not seen worms before.*

M: What do you think are some of the symptoms when someone has worms?

C8: People with worms vomit.

C3: Others have enlarged abdomen.

C9: Others eat a lot.

C4: They eat very frequently without getting satisfied.

C1: Vomiting.

C9: Also abdominal pain.

M: How do you think people get worms?

C9: From foods such as pork meat.

C8: eating soil.

M: How can people prevent themselves for getting worms?

*Silence*

C9: By going to the clinic.

C3:....and get vaccinated.

C5: Eating rape a lot helps prevent worms.

C7: Eating cabbage also.

C6: And also not going to the toilet with bare feet, i.e. without shoes.

C9: And also not going to the toilet while eating something.

M: Is having worms a dangerous thing?

C3, C6: It is a dangerous thing because if you have worms you can die.

M: Are there diseases that are worse than worms?

C9: Headache.

C6: Diarrhoea.

M: What are some of the consequences or effects of having worms to someone’s everyday life?

*Silence*

M: Have you heard of epilepsy?

*Reluctance to respond*

M: What are some of the causes of epilepsy?

C3: You can get it from the air.

C2: When body temperature rises.

C8: You can also get bewitched.

M: Is there treatment for epilepsy?

C9: Yes, there is at the clinic.

C4: Some people go to traditional healers.

C9: For *nsembe* in both humans and pigs.

M: Are there people in your village with epilepsy?

C4: There is someone with epilepsy in our village.

M: Are there diseases that are worse than epilepsy?

*All: No.*

M: If there was a law that say all pigs should be confined, can there be a problem?

All: No, there wouldn’t be a problem.

C5: But people who fail to feed their pigs may a problem with that.

M: And if the law said all households should have toilets?

C3: That will be tricky because we have freedom to do what we want. Others feel free to go to the bush than to use latrines.

C7: Some people are lazy to build toilets by themselves and hence continue going to the bush.

M: How about if we said all pork meat should be inspected before eating it?

All reluctantly: There would be no problem.

M: And washing vegetables?

C9: There some cultural preferences on that one. Some women fear that if you wash vegetables especially *chibwabwa*, it loses its taste.

*Chibwabwa*: Pumpkin leaves commonly eaten in Zambia as a vegetable.

M: If the law said all people should observe good personal hygiene, would there be a problem?

All: There wouldn’t be a problem.

M: How about treatment for worms?

All: There wouldn’t be a problem.

M: And if the regulations said all pigs should be vaccinated against *nsembe*?

All: There wouldn’t be e problem.

M: Even if the people were asked to contribute or pay something for the vaccination?

C1: If the vaccine is costly some people might not manage while others may manage.

M: Thank you everyone, we have come to the end of our discussion. We have talked about pigs, their importance, and their dangers to us and their benefits. We looked at how we keep the pigs and how we get to recognise our pigs even when they are sick. We went on to look at some diseases like worms and epilepsy and how they can be treated and prevented. Finally we looked at various proposed control measures and how willing the people can accept them and what challenges can be encountered in their implementation. Thank you very much for coming her at Kakwiya and if you have any questions you can ask now.

The end
