# Supplementary material for: Why Latrines Are Not Used: Communities’ Perceptions and Practices Regarding Latrines in a Taenia solium Endemic Rural Area in Eastern Zambia
Source: PLoS Negl Trop Dis. 2015 Mar 4;9(3):e0003570. doi: 10.1371/journal.pntd.0003570 (PMC4352092; doi:10.1371/journal.pntd.0003570)
Supplement: S1 Dataset — (ZIP) [file pntd.0003570.s001.zip › FGD transcriptions_Zambia-2010/Mkopeka_Mr Sakala/Men_Mkopeka_03-08-10.doc]

**Title : Focus Group MEN**

**Date :** 03/08/10

**Site :** Kakiwa Rural Health Center

**Village :** MKOPEKA	**Location :** Petauke district, Eastern Province, Zambia

**Duration (total time) : 74 min	- Start :** 14:00	**- End :** 15:14

**Participants:** 8 men

**Informed consent:** signed and available

**Moderator :** Mr Emmanuel Mwanza	**Recorder :** Dr Andrew Phiri

**Transcription/translation :** Dr Sakala

**Abreviations : Mod: moderator, R: recorder, I: intervener, C: children, W: women, M: men**

**Disposition (from the left of the Moderator):**

**M**

M1

M2

M3

M4

M5

M6

**R**

M7

M8

Age composition:

M5: the oldest

M4: the youngest

All the rest are middle age

Typology:

M6-M4: shy

M8: funny

M3: expert

M2: dominant

**M**

M1

M2

M3

M4

M5

M6

**R**

M7

M8

**Comments about participants :**

M5 is the headman and oldest, others fairly young men. Level of living is poor.

Used local language (Nsenga)

**Group dynamics :**

Level of participation was excellent. M6 was quiet. Discussion was free and frank. Level of interest was good for all topics.

Laugher: Description of open-air defecation and its challenges, concerning feeling disgust about consuming pork which feeds on feces, concerning pork preparation methods, use of latrines

Silence: on tapeworm infection

After meeting: Acknowledged the knowledge they shared in the focus group discussion, requested to watch video that was recorded (they were very pleased with it).

**Text:**

*Introduction*:

R: Do you eat pork?

All: Yes. *(Lifting their hands)*

R: Where do you get the pork that you eat?

M7: We just buy.

R: Have you ever kept you own pigs?

M2: No.

R: But you see how your friends keep them?

M2: Yes.

R: Do you all come from the same village? Which village?

M1: Yes, Mkopeka village.

R: Are you the headman, do you have only one village headman?

M1: No, it’s that man *(M5)*.

R: Ah, so you are headman Mkopeka?

M5: Yes.

R: What is the meaning of Mkopeka, I asked the women and they could not answer, so I want to ask the patriarch?

M5: I do not know. It is a name from our forefathers.

I (Dr. Mwape): What can I do to become an elder in you village?

R: Can a person who just comes from somewhere become one of the elders in your village?

M1: No, that is impossible.

I (Dr. Mwape): Impossible?

R: How about me?

M1: You can stay because sometimes it happens that you come with your family then you ask the headman to give you an area where you can stay with your family.

R: What if I stay for years and years?

M1: Still you would not become the headman.

I (Dr. Mwape): What if I come and go to chief Nyamphande and tell him that I want to be a headman?

M1: No, you can not, unless you go to the chief that you were a headman in where you come from. Only then he can give you an area where to settle with you family and then you would be the headman.

I (Dr. Mwape): What if where I come from I am not a village headman?

M1: It means even here you can not be a village headman.

I (Dr. Mwape): Oh, then it is better in Tongaland.

M5: What happens here is that in the village where you arrive and stay. That is where the village headman will give a place to build your house and a field.

R: if I come to Mkopeka village would give me a place?

M5: Yes we give.

R: Ooh! Okay.

M1: Do not forget to see the chief.

R: How about a single woman?

*Laughter*

M1: That you have to find on your own.

*More laughter*

I (Dr. Mwape): I thought that when you arrive, you are given a full package of everything?

M5: Aah no, *(amid laughter)*.

M8: Single women are everywhere even on the road you can get one just now, but first you have to ask the headman.

R: How much is a dowry here?

M1: You would pay about twenty thousand kwacha.

I (Dr. Mwape): We heard that single women are very reasonably priced just five hundred kwacha.

They all said that money may just allow you to talk to her and establish friendship.

Mod: Thank you very much, you are all welcome here at Kakwiya clinic, like it has already been said by my friends what we need to do is to participate in this discussion very free. We have come here to share knowledge on how we rear pigs in our villages. But before we start our discussion the most important thing is for us to know each other, so I will start by introducing myself although most of you know me, I am Emmanuel Mwanza one of the helper here at Kakwiya.

M1: I am Magilasi Tembo, I come from Mkopeka village, Chief Nyamphande.

M2: I am Isaac Mphande, I come from Mkopeka village, Chief Nyamphande.

M3: I am John Tembo, I come from Mkopeka village, Chief Nyamphande.

M4: I am William Zulu, I stay at Mkopeka village, Chief Nyamphande.

M5: I am Mike Tembo, I stay at Mkopeka village.

M6: I am Christopher Tembo, I stay at Mkopeka village.

M7: I am Timoth Daka, I stay at Mkopeka village

M8: I am Samson Daka, I stay at Mkopeka village, Chief Nyamphande.

*Discussion*:

Mod: The first thing we want to know, are pigs needed in our village?

M1: Yes they are needed.

Mod: Why are they needed?

M1: They are needed because if you have a job at the field you can sale a pig and hire some people to help you. When there is a funeral you slaughter a pig and you have a lot of relish. If you had to slaughter a goat you need to slaughter two instead of one pig. Also pork has got very good fat always. Pork has a lot of fat that is why find it good.

Mod: Good, any other thoughts why pigs are good?

M2: In addition pigs help us in the education of our children. For example if a child passed to go to secondary school if you had five castrated boars you are sure you will be able to send your child to school. The price of a pig is much more than that of a goat. A pig would sale for two hundred thousand kwacha you can therefore not fail to send a child to school.

Two hundred thousand kwacha = about 20 USD

M3: In addition, we are now going towards the farming season and if you had two pigs and sold them you would be able to finish the whole field no matter how much weeds were in your field. That is the other goodness of rearing pigs.

Mod: Any more additions?

M8: Like in my case. For me to come here I had four castrated male pigs which I sold to carry all my belongings to come to Chief Nyamphande area.

Some men: Where are you originary from?

M8: I came from Chief Kalindawalo.

Mod: Any more additions?

M7: Yes I can still add. Say if you had no money to grind your maize, especially during the rain season when money is very scarce. You just sale a pig and there is your help. Pigs assist in many numerous ways. That is the beauty of rearing pigs!

Mod: Like you have explained on the goodness of pigs, would there is bad things that you would associate a pig with?

All: there are bad things that are associated with pigs.

Mod: What bad things would you associate with a pig, M5?

M5: Ah, a pig can be associated with many bad things because it does a lot of bad things. But if we put it in a fence, feed it with natural foods and care for it then it would not have any problem. But if we left free range to scavenge then there are a lot of problems.

Mod: M2, what do you see as a bad thing about a pig?

M2: The worst thing about a pig is that sometimes for those without toilets if he wants to go to the bush to help yourself, you find that the pig has run before you and has already allocated a place for you where you are going to sit and is waiting for you.

*Laughter*

M2: Some times if you are not finishing fast you find that it pushes its snout between you legs, jerking you forward so you just move forward a bit. That in my view is the bad thing I find about pigs.

*More laughter*

Mod: Any more additions on why pigs are bad?

M3: Like we have already heard that it would follow you in the bush and eat the human excreta and the same pig would run home and it in your cooking pots.

Mod: Ah please go on?

M1: Sometimes it happens that women would go and burry a still born child at the graveyard. Pigs manage to dig up the child and eat it.

Mod: If a pig gives birth and you do not feed it with bran, what would happen?

M3: I would eat all its children.

M7: If you do not feed it when it gives birth or the food you give it is not adequate it will feed on its own babies.

Mod: You have explained the bad things about a pig, what threats do they pose to our daily life?

M3: You would have to make a fence around its kraal and give it food in there so that it does not go out to scavenge.

Mod: Any other additions?

M2: Like someone said. A pig would eat human excreta and come to eat in the plate and some women do not clean the plates thoroughly well. So when they put food in that plate, we could find ourselves suffering from diarrhoea.

Mod: any more additions?

M8: Now like others have explained you would make a fence but there must be a kraal inside the fence, because the fence is to help the excise. But if you do not feed them enough food you will find that hey start dying. Now if you examine the meat you find that it has grain like structure in the flesh. Then you start telling whoever is responsible for the feeding of pigs that you are not a good rearer of pigs. Then you begin to search for answers. Really there is greater need to have a veterinary Dr. among us who knows livestock diseases.

Mod: Anyone who can explain, from the point which has been raised?

M1: The pig can be compared to a house fly which goes to the toilet and comes back to the kitchen. These two display the same characteristics.

Mod: How do we rear our pigs, are they free range?

M1: Yes there are few who make fences for them but the majority just leave the pigs to scavenge, without due care. That it may bring us diseases. People buy a pig as long as their wives give the pigs bran. That is it. All they know is that they have a pig.

Mod: What is the major problem why you do not build fences?

M7: In most cases it is possible to build a fence but you might find that you do not have enough food to find it in the fence. For fear that it may starve to death; you open the fence to let it scavenge around.

Mod: M2, what is a bad thing a bout a scavenging pig?

M2: The problem of scavenging pig is that a person goes to the bush to help himself, and then the pigs follow and feast on human waste. And also a woman has pre-mature birth and the baby is still born you find a pig digs up the baby and feasts on it.

Mod: Any other additions?

M8: You would take precautions your self and dig a toilet, and sometimes make a fence around your kraal so that they are kept inside. Then a situation arises when you run out of feed. You let the pigs out then the pigs run into the bushes and wait whoever is going to come to help themselves because some of your neighbours would have no toilet. So the pigs spend the whole day in the bushes waiting for whoever comes to help them. Sometimes the child would call the pig and it would not show up. Why? Because during its scavenging time it may have eaten human excreta with a disease that mighty kill it in the bush, sometimes pigs are lost that way. In all honest in the villages we fail to care for the pigs, because we are not able to feed in the manner pigs should be fed.

*Laughter*

Mod: These pigs which just roam about, how do you know that this is yours since another person would also have his pigs roaming about?

M3: Aaah we know them, because when they are born you see them grow up and in most cases pigs do not just move about the whole day, there are times when back home and take a nap then you see them and know that these are my pigs.

Mod: Any more contributions?

M4: Sometimes we cut an ear lobe to help in the identification.

Mod: Any one?

M1: Some pigs are born with some spots and then you would know that these are my pigs.

Mod: Anyone else?

M5: Also pigs easily get used to the home where they stay.

Mod: Now, what challenges do you meet in the rearing of pigs in the villages?

M2: You know that pigs multiply very quickly. Within a short time you find that you have twenty pigs, but then when swine fever strikes, you would find that in one week the whole flock is wiped out.

Mod: Can any one explain other problems that you find in the rearing of pigs like he has just explained?

M3: Like the last speaker has said pigs multiply very fast. You may find that in a short time you have twenty. Now to find feed for twenty pigs is a challenge, so you find that because you can not provide adequate food, they would start dying one by one.

Mod: In the rearing of pig how do you share the roles? Who does what?

M1: Usually the man’s role is to build a kraal and feeding is done by the women and the children. But if the man is lazy he will not go to the bush and cut trees to build a fence around the kraal. So us, the men, are the ones who cause this problem of pigs just roam about.

M5: In the past it was okay because people used to build kraals, but now people have stopped because they say there are no hyenas. The pigs just wonder about when in fact we are bringing diseases to ourselves because we are not taking care of the pigs.

Mod: The same pigs which just wonder about, how do you know that today my pig is sick?

M3: When they return from feeding you would notice that it is not very active and the appearance of the hair on the skin would be standing up.

M5: It usually refuses to feed, it looses appetite.

Mod: Any other signs that would show you that the pig is sick?

M8: Those who rear pigs have several methods of calling pigs to come and feed, some times you just take a stick and tap the trough where you put gently. The pigs would usually come running knowing that there is some food. A sick pig would run behind the other pigs in a staggering fashion then you would know that things are not alright. Also you would notice that the pig snout appears dry, because when pigs smell food their snout is usually wet and very active tyring to smell the food. Sometimes some pigs present very different signs, because of the numerous diseases that affect them some would not be able to stand when they try the fall down, and some would not be able to run. They would fall down if they try to run.

Mod: What do you feed your pigs and how many times do you feed hem in a day?

M6: Pigs are feed twice in a day and we feed them maize bran.

Mod: Anybody else, M7?

M7: This time of the year, there is a lot of food. They would be fed twice and there is variety of food types. They would be fed on left over *Nshima*, pumpkins and maize bran or any other foods that are not required by human beings.

Mod: Any one also what do you feed them?

M3: Pigs can be fed several times per day, just like human beings in the morning when you let them out and in the evening when you put them in a pen. But in times of plenty you can feed them also at mid day just like people. But mainly pigs are only fed twice in the morning and evenings.

Mod: Like, here in the village do you manage to feed them twice or three times in a day?

M3: Yes, twice is possible.

M7: Yes, twice but many people manage only once in the evening, but if it had not eaten where it is it will come back and start to give problem. Then you know that it has not eaten where it came from then you tell the children to give it food and you would see it get into the kraal.

Mod: On the aspect of feeding who feeds the pigs at your homes?

M1: It is the women and the children who stay at home.

M3: Us, men are always moving about.

M1: The men are usually away from home.

Mod: You have said the pigs just wonder about. What do you think they eat when they move about since you said they are fed on once or twice in a day?

M1, M2: They eat human excreta, pumpkins, potatoes and many other crops that remain in the fields after harvesting the crops.

Mod: Are there any family member apart from you immediate family members who come to help you in the rearing of pigs?

All: There are no other family members who come to assist in the rearing of pigs.

M3: Family are your own children who assist with feeding.

*Laughter*

Mod: When the time comes for you to slaughter a pig, where do you slaughter them?

M3: When we want to slaughter a pig, we would catch it from the kraal and take it on the side in shade to slaughter it.

Mod: Why take it aside?

M3: We try to avoid the other pig to come and eat the blood of the other pig. After slaughter we make sure we burry the blood.

Mod: Now, who makes a decision either on the rearing of pigs or when you want to slaughter in your home?

All: it’s us.

M3: The man of course, but in consultation with your wife, because sometimes you would have no soap at home then you would decide to slaughter a pig.

Mod: Others?

M3 mobile phone rings and he walks out to answer the phone.

M7: Yes like it has been stated, it is the man who decides and who has authority to decide. If he refuses nothing can be done, because he is the one who has programmes but also your wife should agree.

Mod: How about the children, can they not complain that it is time they eat some meat?

M1: Yes the children can also participate in the decision making.

M5: yes because they also know that these are their father’s pigs.

Mod: Is that a possibility?

M2: The parents who are open to suggestion listen, but if the parent does not understand, that is not a possibility.

Mod: What is good or bad for a pig to eat human solid excreta?

M1: What I find bad is that you go into the bush and shit then the pig eats the shit. The same pig comes and eats in your plates. It means the pig is feeding you with your own shit.

M3 walks back into the room.

*Soft laughter in the room*

Mod: Any one else?

M5: The badness is that it would bring diseases.

M2: The pig would eat the human waste which I have shitted in the bush and rush home where my children are eating *nshima*. Since children may not be able to chase it away, with the shit still visible on its lips it will come and eat in the same plate where the *nshima* is. This would mean that my children also are feeding on my shit. It would therefore be bring a disease in my family.

M5: If person comes and he wants to buy a pig, you would catch the same pig with human shit still on its lips and slaughter it and start selling the meat.

R: Don’t you feel like vomiting when you eat a pig which does that?

M5: No.

*Laughter*

I (Dr. Mwape): Is shit pig food?

M8, M3, M8: No that is not pig’s food. If it was pig’s food then there would be no toilets.

Mod: So what is the threat to human life in a pig eating human shit?

M1: We do not have equipment to measure that this pig is diseased, but from our own observations a pig goes to the bush and eats human shit and it would go to the graveyard dig up a person and eat. Then we would slaughter the pig and eat the pork. It would appear that we are feeding on our own shit and our relative who the pig ate. Therefore even if we said we do not eat a person but we eat him through eating the pig.

M3: Even snakes which we say we do not eat, the pigs eat snake and in turn we eat pork.

Mod: So, graveyards when they are near the village is that a very big danger if you keep pigs?

All: Yes.

Mod: Among the livestock you keep in the village which one do you eat regularly?

M2: Pork.

Mod: What is the reason for that?

M7: A pig usually gives birth to a lot of piglets, may be ten at once. Because of this they multiply very quickly and in abundance.

M1: In addition they also grow very fast. You find that in six months they would be ready for slaughter.

I (Dr. Mwape): How about the taste of the meat which is better, beef, goat’s meat or pork?

M3: If it is well prepared, pork is the best my brother.

*Laughter*

I (Dr. Mwape): Roasted?

M3: No, cooked.

M2: The other goodness of pork is that you do not need cooking oil, it already has its own cooking oil. So if it is being fried here everybody in the windward side would feel the smell.

*More laughter*

Mod: What do you check for if you want to buy pork?

M2: We check if it has sand like grains in the flesh or if it has fat or not.

M1: If it has no fat then ah we do not think it is good meat.

M5: If it has no fat it means it died from swine fever infection.

M3: Or it is a very old pig.

M8: The first thing we examine is the thickness of the fat layer. If the layer is small then we will not buy the meat. But if the fat is thick and shinny then we buy the meat.

*Laughter*

Mod: The pigs we slaughter in the village are the meat examined, M7?

M7: No.

M5: No, there is no equipment for examination.

Mod: Why do we not have the pig which we want to slaughter?

M7: We have no equipment to examine the pig before slaughter or after slaughter. We just eat the meat. Sometimes we check if it has fat, if it has grains in the flesh and the general appearance of the meat, if it is reddish or the colour of the blood. So we use our eyes and experience to examine the meat.

Mod: You said you use your eyes to examine the meat, how would you know that this pig was sick?

M2: Lets say two people brought meat, one pig was sick the other one was healthy. The meat from a sick pig would appear more red and with thin layer of fat. The healthy pig its meat would be white and with a good layer of fat. Most people sitting here would definitely be able to tell, and if you ask the owner very carefully he would admit that the animal was sick and we just finished it off.

Mod: Would you be happy if the meat of the pigs you slaughter was examined?

All: We would love that.

Mod: What are reasons, for you, to want that your meat should be examined?

M1: The first reason is that yes, we examine with our eyes but we are not able to determine whether the meat is contaminated or not. Now with the advent of technology we may be very happy if the meat was examined and if we are told that this meat is infected we would throw it away and we would comply because we would know that you have examined it and found it not fit. Thais why we are here so that you can educate us and government should help us examine the meat we eat.

Mod: How do you want you meat prepared, roasted or boiled?

M7: That depends on the person some like roasted, some like boiled. In my case I like pork meat which has been thoroughly cooked and I do not have to struggle when eating it.

M5: In most cases meat which is cooked at the funerals is not thoroughly cooked. If you want to eat it you have to struggle with it, almost heating you head against a wall.

*Laughter*

M3: Or when wanting to tear a piece from a served piece you even slap a friend.

M5: Pork must be thoroughly cooked that is when you enjoy it!

M8: I do not enjoy fresh pork. I like my meat dried and then thoroughly boiled, then I can even steal from the pot in the house.

*More laughter*

M3: Everything being said here and some of us like the fat around the neck and if it was roasted, ah my bother! That is the meat I like myself even if I was given a dishful.

*More laughter*

Mod: Are there times when you see that the meat has not been prepared well, and you are not happy with the preparation?

M8: I usually refuse to eat that meat, but I do not say many things because I do not want the children to stop eating.

Mod: Have you ever seen *nsembe* (cyst) in flesh of pig’s meat?

All: Yes.

Mod: What do you think these grains are?

Mod passes picture around.

Mod: When do you see grains?

All: Grains in pig meat.

I (Dr. Mwape): This name of *Nsembe*, where did it come from?

M1: It came fro the Nsenga’s, that is the name we know, solids from locally opaque beer.

I (Dr. Mwape): Why do you call them *Nsembe*, what is it?

M1: When you go to a grinding meal and the mill is not efficient enough to completely grind the maize into powder, those small granules of maize are not ground and that is *Nsembe*. Those are the same things that we find in the pig.

M7: That is how the Nsengas call them.

M5: Even in the past when people used to brew local beer, at the end of the drinking they would squeeze the solids that remains in the pot. They would drink the liquid and they would feed the *masese* to the pigs and when they saw the granules in the flesh of the pig they would say the pig has developed *masese*.

Mod: So do you think those things from the local beer were the cause of the granules found in pork?

Several men: yes, in our own Nsenga understanding we thought those were the things responsible for the condition.

Mod: Now, how about us sitting here today would you say the same about these masese?

M1: No, I think it is a disease affecting pigs, because in our area nobody brews the local beer anymore, but these *nsembe* are still found in pigs flesh.

M2: In addition you find that there grandparents who were brewing local beer are long dead but the *nsembe* are still found in pigs flesh.

Masese: maize bran; nsembe: bigger granules that remain during the making of maize meal.

Mod: Now, let say you slaughter a pig and you find these *nsembe* in the flesh, what are you supposed to do?

M3: If you find the *nsembe*?

Mod: Yes, What are you supposed to do?

M3: That is difficult decision whether to throw it away but most times we decide to dry it on a fire thinking that some of them will burst open and therefore reduce the danger.

*Laughter*

M8: I had raised nineteen pigs then someone came from the Boma. He bought one and took it to the Boma. After slaughtering he found the *nsembe*, he returned the following day with the meat and we agreed because he was not going to be allowed to sale meat at the Boma because he would be arrested. I give him fifty percent of the money and he sales the meat in the villages. But it should actually have been thrown away.

Mod: So when he got that meat, did he sale in the village?

M8: I do not know whether he sold or threw it away.

Mod: Now when you were still with the pig, was it a very fat castrate or did it give any picture that it had *nsembe*?

M8: No, it was feeding very well and very fat. Now each time the young men would come to buy they would check the mouth, the tongue and other parts to check if the pig had *nsembe*. I would assure them that it was only that pig which had the disease, even to day if you came to check my pigs they are clean. There is need to find a remedy to this problem because for us who rear pigs it is a constant worry.

Mod: It is eh?

M8: These are local pigs that we rear in the villages.

Mod: Now on the *nsembe* that we are speaking about it is a huge problem. What do we think about and what can we do about it, M2?

M2: Well to be honest with you our heads are locked. You know this problem has been with us for long time. Some have tried to use traditional herbal medicine with very little success, the problem still persists.

Mod: This traditional medicine how do you administer it?

M2: Some pound it and mix it with the feed and the pigs eat as they eat the feed, but the success is decimal.

Mod: And the animals continue having the granules?

M2: Yes.

Mod: Fine.

Mod: Do you use toilets in the villages where we come from?

M2: Yes some people do and some do not, they just go four hundred metre.

*Laughter*

R: four hundred metre away.

*More laughter*

Mod: What are the advantages of having a toilet?

M1: Like now you can see all the bush has been burnt so if you suffer from diarrhoea, for you to find a small bush where you would help your self, you find that you shit on yourself. So if you have a toilet you just go round your house and help yourself.

M2: The other advantage of having a toilet is that sometimes you run to the bush to go and help yourself then you find that your mother in law, who had gone before, is just squatting a few metres away from you. Because you are in such hurry you do not see her and you help yourself but even if she has finished she will not leave until when you leave. In the case of a toilet at home people will see that a person has entered the toilet and nobody will come until when you are through posting your mail. That is when they also can write their letters and come to post them.

They are expressions to say that you are going to defecate.

*Laughter*

Mod: Any other person who can explain the goodness of a toilet?

M8: The goodness of a toilet is that some time without knowing you give yourself times. Early in the morning the children will hear you door opens and they know that you are in the toilet, they will not come and they will wait. That shows respect. But if you take an axe and pretend that you are looking a handle, then you just cut once and decide to start helping yourself then you hear a branch breaks from the stepping on of a person and then you stop without cleaning yourself.

Mod: Say at you house you have a toilet and children some of which are married, how do you use the toilet?

M1: Yes, it is a problem, because your daughter in law would find that may be you miss the hole and shit on the side or she would find a very big heap and start say this man really shits. In our communities that does not show respect it is always a good idea in that case to have two toilets.

M5: She would say, “my father in law’s heap of shit is like that of an ant eater”.

Ant eater = fourmillier in french

*Laughter*

M8: Yes there are problems when you have very old daughters, because suppose you went in and found that she has shitted on the side, you can not go and start shouting at her. But when you make a toilet you must set rules which guide the use of a toilet. So that even your children, if you are not there, would admonish eat other. That way you do not shout at any one. The toilet must also be kept clean.

M7: Yes, like it has been said to you, the owner and your family would take care of the toilet, but you know that in the villages usually there are very few people who have toilet. A lot of people would come and use your toilet and would not take as much care as yourself and your family. They would mess the place. You would be forced to lock the toilet and keep the key so that whoever comes to use it would come and collect the keys from you just to maintain sanity. The goodness of the toilet is that when you suffer from diarrhoea you straight go to the toilet behind your house. That way the toilet is a very good thing to have at each house hold.

R: Who keeps the keys to the toilet?

M7: I keep the key or if I leave home I give it to my wife. I can not leave it with the children otherwise they may give it to their friends.

R: How about at night?

M7: The children will come and ask for it.

Mod: M2, you have young adult daughter? In our own culture would they come and ask for key from you if they want to use the toilet?

M2: They can not come they feel shy.

Mod: They would feel shy, why?

M2: Ah, if she is married and she has her own home she can not come to me to ask for keys from me, may be the mother.

Mod: If she feels shy then what would follow?

M1, M2: Then she would resort to go to the bush.

Mod: If the keys were put at a much higher place whee children can not reach and all of you are outside, what would follow?

M1, M2: They would run to the bush.

M: Why are you not free yourselves?

M1: it is not possible for your daughter who is a young adult come to the father and say father I want to go and shit in the toilet. That is against our norms and customs and it would never happen, in our community.

M: Are we ourselves? Are we free to go to the toilet even when our mothers in law are seeing us?

M1, M3: Yes we are free, because they know what we are going to do.

I (Dr. Mwape): Your daughter in law, the wife of your son is seeing you, are you free to go to the toilet when she is looking?

M2, M3; Yes we are free because that is my house. In our culture as Nsengas when my son in law comes to marry my daughter and comes to live with us he must dig his own toilet at his own house so that he can be free but I can not go to his toilet.

I (Dr. Mwape): If you daughter in law came to visit you at your house and she is sited with your wife, would you be free to go to the toilet when she is there?

M2: Yes it is my house I can be whistling when going.

M1: If I sit, I may shit in the trousers.

Mod: How about your sons are they free t use your toilet, in you presence and the presence of their mother?

M7: Usually, your sons are very free even in the presence of their mother. Your daughters are too shy to do something like that.

Mod: Now let us talk about personal hygiene, what benefits do we derive from personal hygiene?

M2: Well it benefits us because if we do not take a bath and our clothes are not washed, we would get some diseases which are preventable by these simple and easy things to do. Also you may develop lice so that wherever you sit you would be scratching and if it is here you would not even listen to the lesson because you would be disturbed by the lice bits.

M1: They (lice) my friend carry me, you are the food and warmth and blanket.

Mod: When do we need to wash our hands?

M3: When I go to the toilet, I must be given water before I touch anything or before I start to eat anything.

M8: there are many reasons why we need to wash our hands. It is the hand that touches all things, greeting people if a visitor came home and you greeted him he would look at your hand and say does this man wash his hands. If for example you do not wash your body for three days you would find that the body is itching, sometimes you feel like you have malaria.

Mod: Are children bathed every day?

All: Yes.

Mod: What care do be take of vegetables?

*Silence*

Mod: Can vegetables have diseases if they are cooked before being washed?

All: Yes.

M1: some people spray their vegetables with medicine meant for spraying cotton, then three days latter they would want to sale. Now if you buy and do not wash that vegetable you would have a big problem.

Mod: What could be done in that case?

M7: In such a case the woman should thoroughly clean those vegetables.

Mod: we have heard of *nsembe* infection and fits from people?

All: Yes.

Mod: Are there people who suffer from fits in your village?

All: Yes they are there.

Mod: how do fits infect people?

M3: We have no idea how this disease starts or what causes it. Most time we just hear that the person has seizures and when we bring the patient to the hospital we are told that it is malaria. We have absolutely no idea what causes fits. If we knew we would try to prevent it.

Mod: What do you think would be problems with fits in a marriage?

M2: Supposing it is my wife who suffers from fits and it is a night and we wanted to make love then she starts to fit, I will have to wait before I proceed and most likely you would feel pity for her. You would stop because you think that if I tried again she would start fitting again.

*Laughter*

Mod: Any other contribution?

M1: If your wife was cooking on a fire she would fall on a fire and everything else. If she was drawing water she would fall into the water and die, then she would put the whole family in a huge problem. This is a disease which has given us a very big problem here in the villages.

Mod: would you find prevention or cure like here in the village?

M3: No there is no help.

M8: The only help is at the clinic.

Mod: Have you ever seen the worms which are found in the parasitic infection?

All: Yes.

Mod: Where do worms come from?

*Silence*

Mod: do you know them or have you ever seen them?

M2: Yes we have seen them and know them.

M3: They come from the stomach. That is all we see.

M5: You just notice a swollen stomach.

Mod: how would you know that this one has worms?

M1: If it is a small child you would see it in the stool when it goes to the toilet, then we bring it to the clinic.

Mod: Now those things which appear like a wood borer and like the shaded skin of a pig, have you ever seen them?

M5: Yes.

Mod: Where have you seen them?

M1: In the child’s stool. If a child passes stool you see it in the stool trying to move about.

Mod: Any one else have ever seen these?

M3: Yes we have seen them in the stool of goats and pigs.

Mod: What prevention measure would we put in place to protect pigs?

M1: To examine the meat.

Mod: Would you agree to have the meat examined?

All: Yes.

Mod: What if a law was passed that all house hold should have toilets would that be a problem?

All: We do not think so.

Mod: What if a law was passed that the meat should be boiled thoroughly what problems would you face?

All: None.

Mod: What benefit would you get from thorough washing of vegetables?

M7: Benefits are there, because even vegetables carry some eggs, which can be cleaned by a simple task of using hot water which ably removes them.

Mod: If a suggestion was made to immunise all the children against worm infection would that be fine with you?

All: Yes it would be good.

Mod: What would be the problem if it was suggested that all pigs should be immunised?

M8: I do not think there would be a problem, but there would be need for sensitisation, then our animals would be more health.

Mod: what if the government said that ah now you should also contribute towards the price of the medicine would you agree M2?

M2: yes there would be no problem.

M3: I do not see any problem because we contribute to cattle vaccination.

Mod: We have come to the end of our discussion, we looked at how we can rear pig, the goodness of rearing pigs, the badness of pigs, we discussed personal hygiene, the benefits of rearing a pig, we discussed about worm infection, the hygiene of washing vegetables, we discussed the immunisation of people against worms, immunisation of pigs against worms, I would like to thank you for coming. But if there is anybody with a question they can ask.

M8: we would like to thank you because this is the first time that we have been taught on the rearing of pigs and also the discussion on personal hygiene was very enlightening, we were very happy and thank you.

Mod: Any more additions?

Dr.Phiri’s phone rings and he leaves the room.

M1: It is true that a discussion on pigs had never been held before, which made us believe that we were doing well in the rearing of pigs. I f you want to bring medicine for the immunisation of pigs that is welcome and we shall make a small contribution towards the cost of medicines. All we want is that we rear animals which will give us a good return for our investment. Government saw it fit to teach us we should treat our cattle, why should a pig have no medicine to treat it. We come to the clinic and Mr. Mvula prescribes coartem for our malaria.

Coartem = medecine

M7: I would also like to say thank you for the many things that you have taught us, you discussed about having a toilet in home and personal hygiene.

I (Dr. Mwape): I would also like to thank you for coming. I would also like to say that may be on Friday we can pass in all the villages to discuss further how these diseases are passed on from pigs to humans and humans to pigs. But I would like to state that there is no medicine to prevent or cure granule in pork. Thank you.
