# Supplementary material for: Why Latrines Are Not Used: Communities’ Perceptions and Practices Regarding Latrines in a Taenia solium Endemic Rural Area in Eastern Zambia
Source: PLoS Negl Trop Dis. 2015 Mar 4;9(3):e0003570. doi: 10.1371/journal.pntd.0003570 (PMC4352092; doi:10.1371/journal.pntd.0003570)
Supplement: S1 Dataset — (ZIP) [file pntd.0003570.s001.zip › FGD transcriptions_Zambia-2010/Mkopeka_Mr Sakala/Women_Mkopeka_03-08-10.doc]

**Title : Focus Group WOMEN**

**Date :** 03/08/10

**Site :** Kakiwa Rural Health Center

**Village :** MKOPEKA	**Location :** Petauke district, Eastern Province, Zambia

**Duration (total time) : 59 min	- Start :** 12:18	**- End :** 13:17

**Participants:** 8 men

**Informed consent:** signed and available

**Moderator :** Ms Ruth Chiwa	**Recorder :** Dr Andrew Phiri

**Transcription/translation :** Dr Sakala

**Abreviations : M: moderator, R: recorder, I: intervener, C: children, W: women, M: men**

**Disposition (from the left of the Moderator):**

**M**

W1

**R**

W2

W3

W4

W5

W6

W7

W8

Age composition:

W1: the oldest

W7: the youngest

All: middle age

Typology:

W7: shy

W8: dominant

W5: expert

W2-W4: quiet

**M**

M1

M2

M3

M4

M5

M6

**R**

M7

M8

**Comments about participants :**

4 women are coming from Chimanja village and 4 women from Mkopeka village

**Group dynamics :**

**Text:**

*Introduction*:

M: This is not like a teacher and students; this is a discussion in which all of us are going to participate. But before we start our topic we need to know each other, is that not so?

All the panellists agree.

W1: I am Esther Zulu

W2: Telesa Lungu

W3:

W4: Magret Daka

W5: Sarah Tembo

W6: Evelyn Njobvu

W7: Mumba

W8:

M: Please speak up.

I (Dr. Mwape): Speak like a real Nsenga woman.

M: I will beg you to speak freely and without fear, on what you know and what your role is regarding today’s discussion. Our major topic of discussion today is the pigs.

M: You know pigs?

All: Yes!

M: And you rear them?

All: Yes.

M: Are pigs really needed in our village?

All: yes.

M: why?

All: Because they help us.

M: They help us in which way?

W8: They help us take our children to school, when there is a funeral and many other problems that would befall us. You just sell a pig and solve the problem.

W1: In addition you can use the money to hire people to help you weed the field.

W6: If you sell a pig, you sell it for much more money than a goat.

M: Any other contribution?

*Silence*

M: What is good about a pig?

All: As it has already been said they help us solve a lot of financial problems that have already been stated.

M: What is bad about a pig?

W3: Following a person to the bush.

M: Now, are you saying that the pig just sees a person and follows them to the bush?

W1: Yes when you want to help yourself in the bush.

W5: Yes, because most people in the villages do not have toilets, the pig would follow the person to the bush. After they help themselves the pig would come and eat their stool. That is what I find bad about a pig.

M: What are the other bad things about a pig?

W8: Sometimes you would find that a pig comes directly from eating stool and finds a child eating something, it would go to the plate and eat from the plate and the child without realising would just continue eating the food. Therefore, it is difficult to prevent diseases that are transmitted by a pig.

M: Others, what are your thoughts about the badness of a pig.

W5: Sometimes when a woman has a miscarriage, because they say that a still born child should not be buried very deep, the pig would go to the graveyard and dig out the foetus and start to eat it. Then we slaughter the same pig and eat the meat, which is very bad.

W5: The reasons are many, sometimes if you have not taken good care of the kitchen utensils, the pig would come and eat from your plates and pots.

W1: Sometimes when they want to slaughter a pig they would just catch it, slaughter it and someone would just buy the head with feces on its mouth, cut it up and cook and they would say the head tastes better.

*Laughter*

All women: They would even cook the intestines.

M: do you think pigs are useful?

All: Yes.

M: what use?

W1: They help.

W6: They help financially. If you sell a pig you get some money.

M: Any more thoughts?

W5: Yes it is useful in many ways, like here in the village where most parents do not work and if they had a bad harvest and their child passes grade seven to go to grade eight, the parents would sell a pig, get money and send their child to school. But it has also disadvantages that we have already discussed.

M: What problems do you find with a pig?

W5: Most problems have already been discussed that they would eat human stool which we detest, and we in turn would slaughter the pig and eat the meat.

M: What threats to human life are associated with a pig?

W8: The threat is that of diseases, if you cook pork and it is not fully cooked the gems in the pork will not die; it would mean the disease will be transferred to the humans.

M: Any one else?

W4: To minimize the pig from scavenging and eating dirt, it would be best to make a fence around the kraal.

M: Any thing else?

*Silence*

M: What if one pig is sick what would you do?

W1: If one pig is sick, you catch those which are not sick and put them in kraal and not allow them to come out.

M: Are pig diseases frightening?

All: Yes.

M: How?

W1: Well, when one pig falls sick all of them will get sick.

M: Do you keep any other animals?

All: Yes.

M: That you women rear on your own, like which ones?

W8: Like, chickens and goats, which us women manage on our own except cattle.

M: Does it mean you do not rear your cattle because you can not look after them?

W8: If you are alone you can keep, but we mostly care for goats, pigs, and chickens even when your husband is not there. The ones who usually care for the cattle are the man.

M: What if he says this cow is for my wife?

W8: There is no problem; if the animals come I will pen them in when my husband is away.

M: Why do you keep these animals?

All: For assistance.

M: How are you assisted by these animals?

W6: From eating the meat, if you slaughter a goat the children enjoy eating the meals. Sometimes even when you have a problem that requires finances, you sell a pig or any other animal and you solve the problem.

M: Any one else?

W5: I we sell animals and use the money to send our children to school, also if you are sick and come to the clinic and if the doctor tells you that there is no medicine, you can go home sell an animal and use the money to buy medicines. So the animals we keep assists in many ways, even when you want to have your maize ground and you do not have money you sell your chicken and use the money to have your maize ground.

W1: If you receive a visitor, you slaughter a chicken for the visitor as a sign of respect and the visitor would be happy.

R: Then I will be your visitor at your house.

*Laughter*

M: What role do you play in the rearing of pigs?

*Silence*

M: Maybe I should say what work do you perform in the rearing of pigs?

W1: We look for food for them like maize bran.

M: And others?

*Silence*

M: You do not participate?

*Silence*

M: You do not contribute anything to the rearing of pigs

W8: For a pig to grow up strong and healthy you need to feed it three times, in the morning, at mid day and in the evening, so this is the part we play in the rearing of pigs.

M: W7, can you add anything?

W7: We are responsible for the feeding and building a kraal.

M: Anything else?

W6: When your husband buys a pig you know that your role would be to take care of the pig.

M: So your husband buys a pig and say, this is your pig my wife?

W6: Well even if he does not give it to you, you know that your role is to feed the pig. Your husband can not feed a pig.

W1: The pig is usually in the hands of the women.

I (Dr. Mwape): Who constructs the kraal?

All: It is the men.

M: Are there any other people that help you to keep pigs?

W8: There is no one who can help you, when your husband buys a pig it is your responsibility to care for that pig, because it is like you are chained to it.

*Laughter*

W1: Once your husband has built a kraal his job is finished.

W8: He would just be waiting for reports from you, when the pig is sick or this and that.

W1: That now you need to castrate the pigs and so on.

M: So the man is responsible for castrating the pigs?

W8: Yes, so that the castrates should grow strong and healthy so that when you charge, your clients will not argue about the price. You can charge as much as one hundred and fifty thousand Kwacha.

150 000 ZMK = $30

W1: Even two hundred thousand kwacha

200 000 ZMK = $40

*Laughter*

I (Dr. Mwape): Is it the men who build the kraals?

All: Yes.

M: How about the children, what role do they play in the rearing of pigs?

All: They assist in the feeding of the pigs.

W3: We assign the role, to go and feed the pigs.

M: What about other members of the family who are not immediate members of the family, what role do they play?

All: Nothing.

W3: Is it theirs?

W4: They would not play any role, they mind their business.

M: Who makes a decision that this pig should be used for this, this one let us slaughter and this one let us sell or this and that?

W6: The owner.

M: Which owner, even your husband?

W8: The owners, as long as you agree between yourself and your husband, considering the problem that you have before you, the decision is made to sell the pig.

M: Any more?

W3: Yes, like it has been said you agree between yourself and your husband to sell the pig, so that you solve the problem in your home.

Someone from the background: How about if you do not want yourself?

W3 & 5: Well you have to agree with each other because it is only when there is a problem that has befallen you that you decide to sell a pig. Take for example if your friend had a funeral, your husband will call you on the side and ask that you assist them, then you agree because why should you refuse if there is a problem. Unless there is no problem then you would refuse since there is no problem requiring such drastic measures.

M: How about the children they are not part of the decision making?

W8: They can contribute, but they do not have power to influence the decision.

W3: They can ask that “can you slaughter a pig” but if that is not what their parents want, nothing can happen.

W5: Nothing can happen.

M: Who feds the pigs?

W8: If you the mother do not care to tell the children to feed the pigs, then the pigs will not be fed but if you care then the pigs will be fed. If you left your home and stayed away for a week you would be guaranteed that your animals are not fed or will sleep outside because the job of organising the children to fed and pen the animals is solely your responsibility.

M: W4, your thoughts?

W4: It is true that if you went away you would be sure that your animals are sleeping outside and are not being fed.

M: Can the men not feed and pen the pigs?

All: Yes they can.

W5: The men can pen them in if you are not there.

W8: There are men who do not stay at home who would arrive home late into the night, when do they pen the pigs?

*Laughter*

W2: Some men would not care to pen the pig, which is the truth.

M: How about feeding them, how many times are they fed and what type of food do you feed them?

W8: We feed them maize bran.

W1: They should be fed three times.

W8: They should be fed three times, because even yourself, when you wake up in the morning you look for something to eat, so you should feed them in the morning, mid day and in the evening with some bran, the goat is the one which is rarely fed.

M: Does it mean you feed the pig with maize bran only?

W8: Yes, that is what pigs are usually fed on, some times they can be fed on pumpkins but this is only for a short time after the harvest but mostly they are fed on maize bran.

M: Anybody else, how about left over *nshima*?

W8: Yes, all, even scrapings from the pots are all pigs’ food.

I (Dr. Mwape): How about human stool?

W1: They eat.

W8: They pick feces on their own where they go scavenging.

*Laughter*

All: Where they go they eat any thing, the only food you know is what you feed it, anything else only the pig knows what it eats.

*Amid laughter*

W8: But if you feed it regularly, you find that it does not go about scavenging you just find it near the home resting in the shade, because it is satisfied.

M: Now, if we think very deeply what is god about a pig eating human stool?

*Silence*

M: What is good about a pig feeding on human stool, mum (W1)?

W1: There is nothing good because the disease which would come from the person who left the stool would affect the pig.

M: My sister (*W2*)?

W2: It is bad because it may contract a disease.

M: Is it not good because it helps us not to look for its food?

All: No.

W8: Just a small piece of shit, would a pig be satisfied?

*Laughter*

W8: It would not be satisfied because the shit would just remain in its teeth.

*Laughter*

M: What is bad about a pig eating feces?

All: It is bad.

M: How?

W8: It may be that the person who is shitting in the bush is sick, therefore if the pig eats that stool it will in turn become sick with the same disease, that is why we are saying it is not good.

W1: It is bad.

W8: It is bad like we have already said that some say the head tastes better, but they may not realise that in the molar teeth there are some pieces of shit which were stuck there, some one would be telling you to pick the head when they do not know that under the tongue there are some pieces of shit. Even if I speak like this I do not eat pork.

M: Any additions?

*Silence*

M: How about to your life, a pig has eaten stool, what would happen to you?

All: It is a disease.

W8: It is a disease.

W3: You would definitely contract a disease because a pig would have fed on feces which would be contaminated and when you eat the meat you would contract the same disease.

M: Do you eat pork regularly?

W1: This time of the year yes, especially roasted pork that is fried on a pan.

W8: Yes this time the roasted pork is eaten regularly, especially that roasted on a pan before blood finishes coming out you would find people busy eating the meat, which is partially roasted. Most people think they are doing something good when they are actually adding diseases in their bodies.

W1: During some celebration events, like the initiation of girls when they reach maturity.

W5: But in most cases pork is consumed in large quantities during the dry season.

M: How about during the Christmas period.

W5: That is when there is another peak when pork is consumed in large quantities.

W6: Yes people slaughter meat to eat at home.

W3: That is when people slaughter their fattened castrates and eat at home.

*Laughter*

M: These same pigs which are slaughtered, where do they come from?

All: It comes from the surrounding villages.

W2: They buy them from the surrounding villages.

M: Those you rear in Mkopeka village, you do not slaughter them?

W1: We slaughter

M: When the meat comes and there is an announcement, what do you check for before you buy?

W1: We check for the fat.

All: We check for the fat and the appearance of the meat.

W8: Yes you check for the fat and the appearance of the meat, some of the meat when you just look at the skin it appears white and with a lot of fat with very little steak. Then you say yes this is good meat.

M: How about others, what do you check for?

W6: We check for the fat.

W5: We check for the fat, then we check for *nsembe* and when you see *nsembe* then you know that this meat is not good.

W8: Those who know how to buy pigs check in the mouth, and the tongue, if they find that there are *nsembes* in those parts they won’t buy the pig, because they say that is a clear sign that it has *nsembe*.

M: What do others say, we do not buy pork?

All: We buy.

W3: We know that this meat is not good.

M: How do you know?

W3: We know because some of the meat has very little fat and has got *nsembe*.

W8: Some of the meat appear reddish and without fat.

I (Dr. Mwape): Why do you check for the fat?

*Laughter*

W5: Because that is the meat which has no diseases.

I (Dr. Mwape): Do you mean that fatty meat has no diseases.

All: That is what we think

W5: That is what most people think because when you do not see the fat you conclude that the pig was sick, since we have no equipment to measure whether it has a disease or not. But when you see the fat you conclude that the pig is okay and because of the problems of relish in the village you just eat.

W8: Our friends in town are much better off because they eat examined pork.

M: These same pigs when they are slaughtered are they examined?

All: It is never examined.

M: It is not examined?

All: No.

W1: Mr. Mbulo is there to examine the meat

W5: We do not see him

All: We do not see him neither do we know.

W5: We do not know him, because if it was like that there should have been a poster showing his office or other wise he should have been going round to check what is going on.

M: You need to ask for him.

W5: I do not think that is correct, because the inspector should make his/her presence known, go round the villages and try to find out if there are any people slaughtering pigs. I do not think people would go into the fields buy a pig and start looking for the health inspector, that is not possible because those who slaughter pigs are only interested in selling the meat whether it is diseased or not.

M: But would you be happy if the meat was examined?

All: We would be very happy if the meat was examined.

I (Dr. Mwape): Let us suppose that you had three thousand kwacha ($0.80) in the house and went to buy a piece of meat, and later found out that the meat had *masese* what would you do?

All: We would throw it away.

W8: But you check the meat before you buy and if you discovered that it had *masese*, you do not buy and go back with your three thousand kwacha.

W2: If you send a child you take back the meat and get your money.

I (Dr. Mwape): What if you husband brought the meat and says roast the meat for me?

All: You tell him to take back the meat, or throw it away.

W8: That meat is suitable for a drunkard, who does not care what he eats because even if a person killed a cat he would eat.

*Laughter*

M: how do you prepare the meat?

W5: Once we buy the meat, we take it home cut it into smaller pieces put in some water, boil the meat and when the water finishes you fry the meat, put in some tomatoes boil and make some gravy and then it is ready to eat.

M: How do some of you do it?

*Silence*

M: You do not cook pork?

All: We do.

M: Do you all cook the same way?

All: We all cook the same way.

*Laughter*

W1: But it must boil for a much longer period.

W6: Some of us do not eat roasted meat.

M: Why?

W6: Once I eat, the smell of smoke does not finish quickly in my breath.

I (Dr. Mwape): What if your husband is rushing, for example to the chief and asks you to roast a small piece?

W6: My husband does not eat roasted meat.

*Laughter*

W3: You just roast for him

M: So does it mean all of you, your husbands do not eat roasted meat?

W2: They eat; we roast and give them to eat.

M: So what do we think about eating roasted meat?

W5: We do not think roasting the meat is the best mode of eating pork, because the germs do not cook.

W6: The way we have learnt, we have seen that it is not a good method of cooking pork, so we shall stop roasting the pork.

M: How about those who roast in the street?

W3: We do not buy from those people.

W5: It is the drunkards who usually buy from those traders.

M: How about at a funeral?

W5: There those who know how to cook they cook just fine and there are those who are careless.

W3: Some are really terrible.

W8: Some cook well some it is pathetic, the meat would be white and you feel ashamed even to see the pot.

W6: Some would call you to come and eat even before burial, so when did they cook the meat.

M: Have you ever seen *nsembe* in pig’s meat?

All: yes, we have seen them.

M: When we see the *nsembe* what do we think?

W5: We think that it is a disease.

M: Any one else?

W3: We think it is a disease.

M: If we think it is a disease how do we think if comes in the pig?

W6: We know it is a disease but we do not know how it infects the pig, but we have also heard that it the bad hands of the feeder that causes *nsembe* in the pigs.

M: Any more contributions?

W8: In the past they used to say if you feed the pigs with *masese* (residues from the local beer) from the local brew then that is what causes *masese* in the pig, now that is in the past, but now there are no people who brew local beer how come the pigs still have *masese* in their flesh? Maybe you who are educated can teach us what causes *masese*.

M: Have you ever eaten meat with *masese*?

All: We have never.

M: You are sure you have not tasted *masese* meat, be honest ladies?

All: We have never tasted it.

W5: From the time I started cooking on my own I have never eaten *masese* meat, may be when I was young.

I (Dr. Mwape): How does *masese* meat taste like?

W6: We are refusing that we have never eaten *masese* meat; it would be therefore difficult to know how it tastes.

M: But some of you have said you tasted it when you were young?

W5: Even *nsembe* they do not cook they just remain like that after cooking.

W8: Even when you are eating the *nsembe* they just pop.

M: Have you eaten them before?

W8: In the past when I used to eat pork, I ate pork with *nsembe*.

*Laughter*

W8: Yes, even if you over cook the meat to the extent that your finger can go through the skin of the cooked meat, *nsembe* does not cook.

M: What if you roast it, they do not burn on the fire?

W8: No, it does not burn, they cover the whole steak.

I (Dr. Mwape): How about when you dry the meat?

W8: Still more.

W5: Even then.

W4: Even when it is dry it shows that it had *nsembe*.

M: Do we have toilets?

W5: Yes some have got and some do not have.

M: How about in Mkopeka village, which is the largest number, those who have or those who do not have?

W5: The largest number is those who do not have.

M: Where do you think those without toilets go?

All: They go to the bush.

W3: Others go to the bush and still others go to the nearby toilets.

M: What is good about going to the bush?

All: There is nothing good about going to the toilet.

W8: There is nothing good about going to the bush because you would find that when you go to the bush you find that your son in law is coming yonder and comes to sit next to you, and then you just look down in shame.

*Laughter*

M: Others, the goodness of going to the bush?

All: There is nothing good about going to the bush.

M: Not that you reduce the job for the men to dig toilets?

All: No.

M: What is good about having a toilet?

W8: If we had toilets even the pigs would not feed on human feces.

W4: In the village the diseases reduce because you use toilets.

M: Anyone else, my sister (W2)?

W2: It is good to have a toilet.

M: Madam (W1)?

W1: If someone does not go far to help himself, the flies that sit on the feces would come and sit on the food, that would transfer the diseases to you.

Someone from the background: So, you who have toilets, the whole family uses the toilet?

W1: Some go in and even those without toilets use the toilet.

M: We are talking of all the family members, do they use the toilet?

All: No and yes.

W5: There are times when there is one toilet at home if that toilet is for the father then the children would have to go to the bush, if the toilet is for the children then the father has to go to the bush. Otherwise it is taboo for the children to use the same toilet with the father.

M: Others, what do we think?

W6: Like us we have our own toilet and the children have their own toilets.

*Silence*

M: Madam (W3)?

W3: A toilet is still necessary.

I (Dr. Mwape): So every home should have two toilets?

W5: Yes that would be the ideal situation, especially if you have a big family and older daughters. You need to have two toilets because older daughters can not use the same toilet with their father. If it is you and your husband with small children then you would have one toilet.

I (Dr. Mwape): Why can you not use the same toilet?

W5: Sometimes you find that the father is already in the toilet and if child wants to go to the toilet and finds the father in the toilet, the child will go to the bush. That is why we are saying you need two toilets.

W1: What if you have a son in law in the village you would not use the same toilet?

W8: In fact, when a son in law comes to marry he should immediately be told to dig his own toilet, it would not be good to use the same toilet with your son in law.

M: How about personal hygiene what do you think?

W1: It is very good to practice good hygiene, because you avoid some sicknesses in that not every small disease that breaks out you would be the first one to suffer from it. If you practice good personal hygiene you avoid all that.

W4: If one member of the family suffers from diarrhoea you find the whole family suffers from the disease if they do not practice good personal hygiene.

W8: When you practice good personal hygiene you find that it would take you a long time before you fall sick. So a good result of good personal hygiene is very effective preventive measure of any disease.

M: Do you think bathing the children and caring for them is a very good thing?

All: Yes, that is a very nice thing.

M: Why?

W8: That is very good.

W1: It opens up the poles on the child‘s skin and the body breathes better.

M: Any one else, do we all have children?

All: Yes.

R: There is none who is (ngomwa) barren?

All: There is no *ngomwa.*

W6*: Ngomwa* refers to men, for us women the word used is *chumba*.

R: *Chumba*?

*Laughter*

W6: It is very important to bath the children and care for them, because just like you take good care of yourself you should do the same with the children so that you can also reduce the disease burden.

M: When we come to washing your hands, when should we wash our hands?

W8: When you wake up in the morning you should wash you hands, because you do not know where you scratched during the night.

*Laughter*

W5: Before you touch anything you should wash your hands.

M: Anybody else?

W6: When you want to eat *nshima* you must wash your hands, after eating *nshima* you have to wash your hands.

M: Like Mr.Mvula has just walked in then I come from the toilet, i just walk in and greet his hand?

All: No, that would not be very good.

W8: That would not be very good because you may pass a disease to him.

I (Dr. Mwape): Do you wash you hands when you are eating maize or groundnuts?

All: You wash your hands.

I (Dr. Mwape): Do you really wash your hands?

All: Yes.

Someone from the background: When you are in the field digging up the groundnuts is it possible that you wash your hands?

W5: We carry water to the field so if you go to the toilet you wash your hands.

W3: In case you should eat the groundnuts as you are working.

*Laughter*

M: Ladies say the truth.

M: The water you carry is about five litres and you have children who drink so much water, do you think it is possible to wash your hands with this little water when you come from the toilet?

W6: During that time there is always a lot of water in the small streams, so there is a lot of water in the fields and so the only water you carry is for drinking.

M: How about in June when you are harvesting groundnuts?

W6: The running water would have stopped but there would be water in the water holes.

M: Have you ever seen worms?

All: We have seen them.

M: How do they come about or where have you seen them?

W5: We have seen them in feces.

M: Any one else?

W2: Some just vomit them.

M: Where do they come from and how do they come?

W5: Where they come from we do not know because some people say they come from eating the soil and some say they come from rape, we therefore do not have very good information on where they come from. We would be very happy if you can tell us where they come from…

M: Where do these worms come from?

W3: We just think that it is a disease also.

M: The person with worms, what do they complain about?

W6: They complain of stomach ache and if it is a small child the body does look nice and the stomach appear swollen.

W5: The child suffering from worms eats too much.

M: Others, what have you seen?

*Silence*

M: Have you seen those things in the picture, have you ever seen them?

All: We have never seen them.

M: In the stool of someone?

*Silence*

M: That is someone’s feces or vomit.

*Silence*

M: If you saw these things in someone’s feces, what would you think of that person?

All: We would think that such a person was sick.

*Whispers*

M: Those are the young worms before they grow up.

W8: Which ones are they?

M: It is those which appear white.

I (Dr. Mwape): Have you seen something like this before?

R: Those that look like a wood borer or the shaded skin of a snake.

M: Have you ever seen these?

W8: These are like those you find in a chicken’s intestines, when you slaughter a chicken you find things like these.

R: Have you ever seen them?

All: Yes.

R: In a person?

W8: Not in a person but in a chicken.

M: Do you think a disease like this can prevented?

All: Yes.

W6: If the government looks at it, maybe it can be prevented.

M: On our own we cannot prevent it, but we have to wait for the government to come and do it?

All: Yes

W8: Yes, because unless we knew how this comes and where it comes from then we would look for a solution on how to prevent this, but the government with all its resources and trained man power would assist in finding prevention.

M: How about treatment, can this disease be treated.

All: Yes, it can be treated.

W8: Yes, it can be treated there are more powerful diseases which are treated.

M: How is it treated?

W6: If a patient comes to the clinic and they give him treatment and all the worms come out, he is completely cured.

M: Is it frightening to have this disease?

All: Yes it is.

M: How is it frightening?

W8: It is frightening because if you try to vomit the worms and by mistake you bite one, you will immediately die.

M: Have ever heard of someone dying because he had bitten a worm?

W8: Yes

M: Where?

W8: At Chief Kalindawalo.

Chief Kalindawalo=Neighbouring chiefdom about 50Km from Kakwiya RHC

M: Is there a much more dangerous disease than worms?

W8: This is a very dangerous disease than this modern disease.

M: Which disease.

W8: These many diseases.

*Laughter*

M: All diseases are many *(amid laughter)*

I (Dr. Mwape): Which disease mum (W8)?

W8: This disease is bad because an AIDS patient can live much longer than people who suffer from a head ache and die, but an AIDS patient can live for a long time.

M: Do you think worms can kill a person?

W8: When they are in the stomach they can not kill a person but when they want to come out that is when it is most dangerous.

W6: Even when they are in the stomach you would find that a child suffering from worms does not grow up well.

W3: Yes, it does not grow up well. You find that in the morning they wake up with a swollen face and looking thin.

M: Which disease do you consider smaller than worms?

W3: There is no small disease.

W5: There is no smaller disease because any disease, if it persists, it would kill you.

M: Do you consider this disease very serious?

W5: It is very serious.

M: More serious than AIDS?

W5: That is also another problem.

M: We have heard that those suffering from AIDS can survive for a much longer period.

W5: Unfortunately there is no such a thing as a good disease, because all diseases if not treated can kill a person.

M: In every day living, if a person has worms what do you think about that?

W6: You take the person to the clinic.

M: Do you think there is any problem if you had worms in your every day life?

W6: Yes, there is a problem, because you would have frequent stomach pains, even if you are hungry you feel like you have already eaten.

M: *Masese*, Have you ever seen them?

All: Yes.

M: Do we think it is disease for animals or does it also affect human beings?

W6: Even us human beings we could have them but we may not see them.

M: What do others say; does it affect animals or human beings also?

W8: They could affect humans as well

W5: They indeed could be for both humans and pigs, because we eat pigs and pigs in turn eat human feces and other things, so there is a very big chance that it can affect humans as well as animals.

M: Since we are saying that they can be in animals and humans...

W8: In a pig.

M: ...How does the person affected with masese appear?

All: We have never seen how the person looks like.

M: Do you know fits?

W6: Yes

M: Have you ever seen it?

All: Yes

M: Where does it come from?

W8: It comes from a pig.

M: How?

W8: When the medicine man wants to treat a person suffering from fits, he stops him from eating pork that is why we think the spirit which causes fits is found in a pig.

M: And what would others say?

W5: In some cases the person would have been bewitched but in some instances the person would start fitting and if they are brought to the clinic, the doctor would say it is malaria and when they receive malaria treatment they get better.

M: Any more contributions?

*Silence*

M: Do you know anybody with fits?

*Silence*

M: Do you know anybody who suffers from fits?

W5: In our village?

I (Dr. Mwape): In your village, yes?

All: In a murmur we do not have.

M: You do not have fits?

W7: They are there.

W2: Who?

W7: Madam Monica.

W6: There was a case but when the person was brought to the clinic they said it was cerebral malaria and after treatment the person is fine. It is therefore difficult to say whether he was attacked by fits.

W8: Like the seizures in our family is very difficult.

W5: Why did you people not answer when the question was asked if there were any people with fits?

W8: She talked about our village, but me I am discussing the one which is in our family, he would start in the morning and it would go on until day break, usually what happens is that you have to bring the patient to Petauke district hospital for treatment that is when the person gets some relief.

W1: In my opinion fits are there but what we should say is that we do not know the cause, because you would find that an elderly person with grandchildren who had never suffered from fits starts fitting from no where, so what we do not know is the cause.

W8: Some would suffer terrible burns on their hands; especially if he is burnt then he would never get cured.

M: Do you think this disease can be prevented?

W8: Many get help from the hospital, if they go to the hospital and get treatment, sometimes they would stay without seizures for five months as long as they continue to go to the hospital and follow the doctor’s instructions.

M: How about being cured?

W7: Some get cured.

M: Those who get cured where do they get this help?

W7: If you come to the hospital, when you get treatment you would find that the fits do not happen again.

W5: Like it has already been pointed out fits are in many forms, here is the form which comes with malaria, that one when you come to the hospital receive treatment then it is cured, there is another which even if you brought the person to the hospital and are given treatment they do not get well. That is when I have remembered there is a person in our village who has been suffering from fits and he has continued coming to the hospital with no relief.

W1: Yes, even now he has grown old with it.

M: Where else can we get help from this disease?

W8: Some when they mix hospital and tradition medicine and they are completely cured.

M: Have ever seen anybody who got cured?

W8: Yes, they are there, like that wife of our uncle who had a miscarriage, it was so bad that you would think she is dead, but when she came here to the clinic and was also using traditional medicine she is now cured. Even our uncle was suffering from fits but not anymore.

M: Do you think they were stopped from eating anything?

W8: They were stopped from eating pork and chicken during the treatment period but after the course was finished and they came and to do the final rituals they are completely cured and were told to eat these animals.

M: Do you think this disease is very frightening?

All: Yes

W5: This disease can make one be like an imbecile.

M: Is there a disease bigger than fits?

W5: Yes there are.

M: Which ones?

W1: Malaria, all diseases are bigger, even a finger disease can kill you.

All: There is no small disease.

W8: There is no small disease because even if you had a swollen finger, headache, and ear diseases, all diseases are dangerous because if they are not treated they would kill a person.

W3: Even toothache.

M: Is AIDS small?

*Laughter*

M: I have never heard at a funeral where an announcement is made that the person has been killed by AIDS.

All: They usually do not announce directly, they announce in parables.

M: What challenges does a person meet in their every day life?

All: The challenges are many.

M: Like which ones?

W6: If they try to warm themselves near a fire that is dangerous, if they go near water that is fatal for them.

W5: They do not really have peace in there day to day living because even marrying becomes difficult because the men are scared of the same disease.

W6: Even chatting with friends they are not free

W1: Even their whole life changes from how they were before and after the disease, they usually look confused. It is like they loose a bit of their normality.

I (Dr. Mwape): Do they get married or not?

All: It is difficult to say, but they can give birth just fine.

W8: They can give birth just alright better than those who are not sick.

W5: Very much as long as there is someone to look after the children for her.

*Laughter*

M: What if the chief or the government passes a rule that all those with pigs should keep them in a fence and should not allow them out to prevent worm diseases and fits, what problem would we find with that?

W5: There would be no problem because those can be kept indoors.

M: Any body else?

W6: There would be no problem except feeding the pigs.

M: Anymore?

*Silence*

M: What if they bring another law saying that all the people must have a toilet what problems would we have and that all people from a child to adults should all use a toilet what problems would be there?

All: There would be no problems.

W1: If you have a toilet that is a very good thing.

M: How a bout on the cooking method, if the rule was passed that all pork should be boiled thoroughly well what problems would we find?

All: There would be no problem because we want to prevent diseases.

M: You would not complain that the time is too much?

All: No we would not

M: What if they say all the animals before they are slaughter should be examined?

All: That would be lovely.

W3: That is when it would be nice because when you are eating the meat you would be free, because you know that the meat had been examined.

W8: If it is bad it should be thrown away before we eat it.

M: We would not complain to throw it away?

W8: No because the animal is yours.

M: What if they say all the vegetable should be washed before cooking what problems would you find?

All: There would be no problem.

W3: Sometimes the vegetables have insects on them so they must be washed before cooking.

M: How about pumpkin leaves, do we cook it after washing, some say it tastes better if it is not washed?

*Laughter*

W8: I think it should be washed, especially if it is fresh because there are crawling snails that would attach themselves to the leaf.

W5: But many times pumpkin leaves when they are fresh, many do not wash ,they just cook it, especially if it has no mud.

M: On personal hygiene if a decree was passed that all people should bath and wear clean clothes, what problems would we experience?

All: None.

W1: When take a bath and wear clean clothes, that is when you look nice.

M: Can we manage, because you do not need to bath every day?

W1: We can manage.

All: We can manage, because you need to take a bath every day.

M: What if they say all those with worms should be treated?

All: The difficulty would be to know who has worms.

M: If they say all of you should be examined and those with worms should receive treatment?

All: We would be examined.

M: What if they say all pigs should be taken for immunisation?

All: We can bring them.

M: There would be no problems?

All: Yes

M: We can not complain?

W5: No because the pigs are not going to be killed and that is just providing prevention.

W8: That would also be preventing diseases.

M: What if they say that you have to contribute or pay a small fee for the vaccinations?

W8: If the price is fine there is no problem.

W6: You sell one and pay for the vaccination of all the others.

W5: But not a very high price because we are already poor.

M: We have now come to the end of our discussion, from the time we started we discussed about pigs, the goodness of pigs and why they are bad, why we rear pigs, what we feed the pigs, and hoe many times we feed them, we discussed about toilets, about hygiene, we discussed about worms, fits, At the end we discussed how we can prevent worm diseases, we discussed that we would prevent these diseases if we put our pigs in an enclosure and did not let them our to scavenge, we clean our vegetable and boil our meat , have toilets at our homes, immunise both humans and pigs, if there is any other question or additions you can add.

W8: We would just like to thank you for calling us here to discuss the mistakes that we are making in the rearing of pigs and the way we prepare our meat.

W5: I have a question; can the government find the medicine for fits especially that it is in many types?

R: You said it is in many types which type are you talking about?

W5: The one which does not cure.

R: Where do you think the medicine can come from?

W5: That one where the person has been bewitched

R: If you take a person with bewitched fits can they get well?

W5: No

R: The medicine is there if they go to the hospital, they get help and medicines, but the problem with you people is that you do not drink the medicine.

Some from background: You do not follow the course.
