# Supplementary material for: Why Latrines Are Not Used: Communities’ Perceptions and Practices Regarding Latrines in a Taenia solium Endemic Rural Area in Eastern Zambia
Source: PLoS Negl Trop Dis. 2015 Mar 4;9(3):e0003570. doi: 10.1371/journal.pntd.0003570 (PMC4352092; doi:10.1371/journal.pntd.0003570)
Supplement: S1 Dataset — (ZIP) [file pntd.0003570.s001.zip › FGD transcriptions_Zambia-2010/Mtuna_ Dr Phiri/Children_Mtuna_05-08-10.doc]

**Title : Focus Group CHILDREN**

**Date :** 05/08/10

**Site :** Kakiwa Rural Health Center

**Village :** MTUNA	**Location :** Petauke district, Eastern Province, Zambia

**Duration (total time) :**	59 min	**- Start :**	11:04 am	**- End :** 12:03 pm

**Participants:** 8 children

**Informed consent:** signed and available

**Moderator :** Ms Ruth Chiwa	**Recorder :** Dr Andrew Phiri

**Transcription/translation :** Dr Andrew Phiri

**Abreviations : M : moderator, R : recorder, I: intervener, C : children, W : women, M : men**

**Disposition (from the left of the Moderator):**

**M**

C1

C2

C3

C4

C5

C6

C7

C8

**R**

Gender distribution:

**C5-C6-C7-C8**: females

C1-C2-C3-C4: males

Age composition:

C8 : Oldest (17 years old)

Typology:

C7: Quiet/shy

C1: Dominant

C5: Expert; Dominant

**Comments about participants :**

3 eat pork out of 8 children

**Group dynamics :**

Not very talkative, the discussion was quiet, difficulties to make them speak. They answer more easily when the questions are not oriented directly to them (e.g. Do you know people who eat pork like this? But no answer when we ask “Do you eat pork like this?”)

**Text:**

*Introduction by moderator:*

M: Good morning boys and girls

All: Good morning

M: We welcome you all here at Kakwiya

All: Yes *(in English)*

M: Do you all speak English?

*Silence*

M: Can we all start speaking English?

Introductions of moderator, children, recorder, Séverine and Evans are made

M: Do you all learn?

All: yes

M: Then you will need to learn more. Do you know what pigs are?

All: Yes

M: The reason why we have called you here is to talk about pigs; why do you keep pigs?

C1: Help with school when we sell pigs

C4: Agriculture help

M: What about you my brother *(asking C3)*. You had a hand raised

C3: I have forgotten what I wanted to say

C5: When there’s a funeral within household, we can slaughter a pig and thus help ourselves

C6: When a funeral has happened in Lusaka, we would sell a pig and use the money realised to cover transportation cost

M: Others. We need to be free and relaxed and say something. No one is going to charge you. There is no wrong answer here, ok?

All: Yes

M: All the answers are correct, ok

All: Yes

M: What do you think is the badness of keeping pigs?

C5: If we don’t have a proper kraal, for us without toilets, pigs eat feces and then come back to household to eat from cooking utensils and plates

C6, C7: *(Giggle shyly)*

M: Others, what are the badness’s of keeping pigs?

C6: When you are fitting, the disease doesn’t get better or healed if someone continues eating pork

M: Others, what do you think is the badness of keeping pigs? You need to be free. There are no wrong answers. All the answers you make are correct ones.

*Silence*

M: Others, what are the badness’s of keeping pigs?

C6: Others get *Mashabe*

Mashabe = being possessed with evil spirits

M: Others with a different answer?

C2: Not building kraals

M: Others? The badness of keeping pigs?

*Long silence*

M: Do pigs have any use?

*Silence*

C5: Yes (*mildly*)

M: What do you think is the pig’s usefulness?

*Silence*

C1: To be helped at the household when the going is tough

M: Others? My sister? (*Looking at C8*)

C8: Agriculture help, when befallen with funeral we can be helped

M: Others?

C3: If there is a funeral in Lusaka, we can sell a pig to realise money for transportation there

M: What about the dangers of keeping pigs? What do you think?

*Silence*

C1: If a pig is diseased and is eaten, one can get diseases from pork.

M: Others? What do think are the dangers?

*Long silence*

M: What about if one pig is sick, what would be the implications to other pigs?

C1: Others can contract diseases from the affected one

M: Others?

C8: When a pig is diseased, we can slaughter it and sell the unaffected other pigs

C1: We need to build kraals without which pigs can get diseases

M: Do you keep pigs

*Silence*

M: Others?

*Silence*

M: Do we know *Chimumphe*?

*Chimumphe* = pig disease outbreak

All: Yes *(in English)*

M: When it comes, what do you think about it?

C3: We think it can spread disease to others

M: Others?

C1: We need to build kraals because if pigs are free-roaming, it means they would also contract the disease

*Long silence*

M: Do you keep pigs? How many of you keep pigs?

*Silence*

C7: There’s no one

M: Of you present here, how many of your parents keep pigs?

*Silence*

M: There’s no one?

C8: There is no one

M: All of you don’t have pigs?

All: Yes (In English)

M: Why don’t you keep pigs?

*Silence, C5-C8 restless*

M: What type of livestock do you keep if you don’t rear pigs?

C2: We keep chickens, goats...

C1: ...and cattle

M: Why don’t you keep pigs?

C5: Because they have terrible diseases

M: Others, why don’t you keep pigs?

*Silence*

M: But you kept some pigs in the past?

*Silence*

C7: yes

M: Long time ago, you had pigs at home

C3, C1, C4: Yes

M: That’s why I told you to be very free. It’s not a crime, so you need to be responding

All: Mmmmmmm

M: So those in the village, how do they keep their pigs?

*Silence*

M: Are the pigs free range or are confined?

C1: Others have built kraals for confining their pigs, but many people just keep them roaming outside.

M: Uhmmm, others? What do you do in our village?

*Silence*

M: Do the pigs come out some times? Those in kraals?

C5: Yes, they free them sometimes

M: Most of the times they are free?

Several children: Yes

M: They confine them at night?

All: yes

M: What is the goodness of pigs that roam freely?

C4: Nothing

M: What about the badness?

C5: Those things eat a lot of different things

M: What kinds of things do they eat, my sister? *(Looking at C5)*

C5: …Like feces, they eat

M: Others? What do we see about free-roaming pigs?

C3: After eating feces, pigs also eat from household cooking utensils

M: Others?

C1: Once pigs find water they play in that dirty water and mud

M: Others who can add?

*Silence*

M: There’s no wrong answer. All of us speak right answers. Ok?

All: yes

M: So everyone needs to speak out

All: Yes

M: When you had pigs, how were you identifying them?

C1: Ear notching on one side

M: Others?

C7: They used to come to kraal on their own

M: Were they coming at every occasion? *(Looking at C7)*

C7: Hmmmm

M: Others?

*Silence*

M: What about those with pigs? How do you think they identify their pigs?

*Silence*

M: They don’t know them?

*Silence*

C1: Others tie a string on one leg

M: Others, how do we see it?

*Long silence*

M: What kinds of difficulties can be found in rearing pigs?

*Silence*

M: The difficulties that can be found?

C2: Not building kraals for pigs to sleep in

M: Yes, others?

C8: Making them sleep anywhere

M: Others, any difficulties that can be found?

C1: When we slaughter a pig that roams about and eat it, we can get sick

M: Others?

*Silence*

M: What are the symptoms of a sick pig?

C8: Sleeps a lot. It walks a bit and lies around. That’s when you know it is sick

M: uhmmm. Others?

C1: It tends to have froth coming out of the mouth

M: Others?

C3: Getting thin or weight loss as a result of hunger

M: Others?

*Silence*

M: When a pig is sick, how do you know it?

*Long silence*

M: What do pigs eat?

C1: Maize bran

M: Others, what do pigs eat?

C8: Grass

M: Others?

C3: Feces

*C2, C3, C4, C7: Suppressed laughter*

C1: When eating sugar cane and you throw away sugar cane peels, it eats the leftovers

M: Others?

C4: *Miphephe*

Miphephe = Cobs of maize

M: What do pigs feed on?

*Silence*

M: Who feeds the pigs?

C1: Us people

M: Us people? Big people, fathers, mothers or you the children?

C6: Us children

M: What about others, who feeds them?

C5: Our parents

M: Male or female parents?

C5: Male

M: Others?

C6: Female

M: Is that the children who feed the pigs more than parents?

C1: Yes

M: You have said many pigs are free-roaming, meaning that they look for their own food? What kind of food do they look for?

All: Hmmm

M: What kinds of food do you think they look for?

C1: Feces

*C5: Laughs as she covers mouth and looks around*

M: Others?

C6: Pumpkins

Kapanda = Pumpkins

C5: Cobs leftovers

M: Others?

C3: What I wanted to say I have forgotten. I may say it later on

M: When you used to keep pigs what was your role?

*Silence*

M: What was the duty you used to perform?

C8: Feeding maize bran

M: Others?

C2: Giving them water

M: Others?

C1: Calling pigs home, some people do so

M: Others, what kind of jobs were we doing?

*Long silence*

M: Apart from our family members at home, were there anyone help you to raise pigs?

*Silence*

M: Was there anyone helping you? Have you understood the question?

M repeats the question

C1: Anyone who lives nearby can assist by calling pigs home when they know there is no one at our home

M: Others? Who were helping us?

*Silence*

M: Wasn’t there anyone who was giving you maize bran?

*Silence*

M repeats the question

C5: When they pound maize, they would call you to collect maize bran

*Silence*

M: Who makes decisions in the home such as slaughtering the pig? Or selling it?

C6, C5: Parents

M: Others? Your male or female parents?

C6: My dad

M: What about sis? *(Looking at C5)*

C5: That’s what I wanted to say

C1: Even us children, if a pig belongs to you, you can just inform your father when slaughtering or selling

M: Do parents understand?

C1: Yes, they do

M: Why do you think your dads have authority to sell pigs? Why do you think so?

C1: Because he is the head of the household

M: What about your mothers?

*Silence*

M: Don’t they have a say?

M repeats the question

C5: Mothers don’t have authority

M: Who feeds the pigs?

C1: Parents and us children

M: How many times do you feed the pigs?

M repeats question

C8: Three times daily

M: What times do you feed the pigs?

C5: Morning, afternoon and evening

M: Others? How many times do you feed the pigs?

C1: Twice daily,…

M: What times?

C1: Afternoon and evening

M: Others? How many times do you feed the pigs?

*Silence*

M: For those pigs that eat feces, what goodness do you see about that?

M repeats the question

C4: No goodness at all

M: Others, goodness about pigs eating feces?

*Silence*

M: Don’t they help looking for the food?

*Silence*

C1: Pigs help themselves to eat the feces when food is finished

M: So they assist to clean up, then?

C5: Yes

M: What about the badness of pigs eating feces?

C1: They may have worms

Nsokanda = roundworms

M: Others? What is the badness of pigs eating feces?

C5: When the pigs eat feces, they usually come and eat from our utensils and thus transmit disease. As such we cannot prevent diseases ourselves.

M: Others? What is the badness?

*Silence*

M: What about you my brother? Is there any badness? *(Looking at C2)*

C2: It is bad

M: How?

C2: When the pig eats feces.......

M: What is the badness?

C1: When you use a dish to give it water and afterwards not washing the dish for washing our hands

M: Does pork get eaten often?

*Silence*

C1: It doesn’t get eaten

M: Others? Does pork get consumed often? Let us be responding youths.

M repeats the question

C3: Not eaten

M: What is the reason?

C1: Many of us think it has diseases. Mr Mwanza came to educate us at Misolo School about pig health dangers

Mr Mwanza = community health leader

M: Do you learn at Misolo?

C1: Yes

M: Do you eat pork?

C1: *Murmuring in the affirmative*

M: How many of you eat pork?

C1-C4: *Raise their hands*

M: What about you girls? You don’t eat pork?

C5-C8: *make some sounds*

M: Don’t feel shy, you mean you don’t eat pork?

C5: We don’t eat

M asks the other girls singularly. They answer no

C8: I ate a long time ago

C1: When shortage of relish

M: what times do you think pork is eaten often? Whether you yourselves eat pork or not?

*Silence*

M: But we know that pork is eaten, not so?

All: yes

M: So when are times that pork is eaten often?

C1: Anytime, when I am asked to go and by. It can be bought in the afternoon and if that portion is finished can also be sent to buy later in the day

M: Others? We are not talking about only those at home but also the ones we buy. When are the times that people eat pork?

Pork is meat, not so?

All: Yes

C1: In the evenings

M: Others?

C5: In the afternoon when relish is difficult to get

M: Others? What about during times of Christmas, don’t people eat a lot of pork?

C4: They eat

C1: They eat a lot

M: At funerals?

C5: They eat

M: At wedding

C3: They eat

M: At initiation ceremonies

C6: They eat

M: These are the things you need to say. What about this time when crops have been harvested and people are relaxing in the village. Do you think they are eating a lot of pork or not?

C1: They eat

M: Do they eat a lot?

C1: Yes

M: So the pigs that people eat a lot where do they come from?

*Long silence*

M: Do they come from your village?

*Silence*

M: The pigs that are slaughtered where do they come?

*Silence*

C1: Buy from others, those close by or even going far to purchase

M: Do you think these pigs that are slaughtered are inspected?

C8, C3: Not done

M: Why do you think is the reason?

C8: People don’t take the pigs to the clinic

M: Others? What do you think is the reason?

C1: Laziness, they think it is waste of time doing so

M: Do people know where meat inspection is done?

C5: Others don’t know where it is done

M: Others, what do you think?

C1: Others know, but they don’t just want to do it

M: Can you like it if the pork is to be inspected?

C5: Yes

M: why?

C4: Because the pigs have diseases

C1: so that diseases can lessen in humans

M: What about issues of cooking. How is pork prepared so that people eat it?

C4: It is cleaned?

M: Do people such cleaned pork?

C3: It is put in a pot

C2: and cook it

M: Explain properly. We will start with the girl. *(Looking at C5)*

C5: When they buy it they clean it and place it in a pot on a fire. After cooking it for a while, that’s when it is ready for consumption

M: Others? What about you girls, how do you cook pork? Even if someone doesn’t eat pork, but one should know how pork is prepared, not so?

C8: Yes

M: So how do you prepare it? What about you my sis? Have you prepared it before? *(Looking at C6)*

C6: I have

M: Tell us

C6: It is first cleaned and placed on fire. When it cooks, we add cooking oil, tomatoes and onions

M: What about you? *(Pointing at C7)*

C7: Clean it and boil it. Then add cooking oil, tomatoes and onions before frying

C8: We clean it and boil it for a long time in case it has tapeworms so that they are killed

M: What about you boys?

C1: It is cleaned, put in a pot with water and boiled until is cooked. Add tomatoes, cooking and onions and then fried.

M: Others? How do we see it?

C2: When it is bought, it is cleaned and boiled for a short time before being fried

M: Is the only way we use to prepare pork, just boiling in a pot or there is another way of preparation?

C5: Others think boiling it is a waste of time. They take it and place on fire and roast it. After adding some salt, they eat it.

C5, C6 and C7: *smile and Laugh*

M: What do others do? Do we know “shockers”?

Shockers = Mix of frying, roasting

C3, C8: We know it.

M: What are the dangers of eating pork prepared in this way?

C3: After *puphusa* the pig and cutting it in pieces, they place it on a pan without even cleaning it with water.

Puphusa = meat getting burnt on the outside without being well cooked inside

C1: Others eat that meat and sip the soup without washing their hands, no wonder some consider that as unhygienic

M: My sister? *(Looking at C5)*

C5: Others don’t make the meat to be well done

M: Suppose we say, we have been called to a meeting at Kakwiya *(Clinic)* and we quickly place meat on fire and roast it. What can you say about it?

C8: The meat does not get well done

M: What do others say?

C1: You can eat together with dirt from the fireplace

M: Is that meat good?

C5: It is not good

M: Why do you say it is not good?

C5: Many times it is not well taken care of

M: Why do you think people like roasted meat?

C3: Because it cooks fast

C1: Others like the smell that comes from roasted meat

M: Have you seen cyst in pork?

*Silence*

C1: I have seen it myself

M: Have you seen cyst in meat like this?

M hands over a picture of meat with cysts

C1: I have seen it in pigs *(as he looks at the picture)*

M: Pass the picture to others. What do you think when you see meat looking like that?

C1: I don’t think much about it, I assume it is good meat to eat

M: What about you girls, if the meat is looking that, what do you think about it?

C8: I think of throwing the meat away

M: Why throwing it away?

C8: Because it can give us diseases

M: What kind of diseases?

C5: Others fit with it

C1: Worm infection

M: What do others think of such meat?

*Silence*

M: Is it when it is tasty?

*Long silence*

M: Have eat such meat before?

*Silence*

M: Have people you know eaten such meat before?

C5: Yes, others eat such kind of meat

M: My brother here, have you eaten such meat before? *(Addressing C1)*

C1: I have eaten such meat; doesn’t taste very well

M: What about you? *(Looking at and addressing C2, C3 and C4 separately)*

C2, C3, C4: I have not eaten it before *(they answer in sequence)*

M: How does such meat taste like?

*Silence*

M: My brother, you who has eaten such meat, how does it taste like? *(Addressing C1)*

C1: It did not taste good; there was no “sound”

There was no “sound” = it didn’t sound good, it didn’t taste good

M: When they cook, do the cysts finish?

Do the cysts finish? = Do the cysts disappear?

C3, C5: Cysts don’t finish

M: What about if meat is placed on fire to roast? Do they finish?

C1: No, they don’t finish.

M: My sister, do you think cysts finish when meat is dried or grilled?

C8: No, they don’t finish

M: But many people eat such?

C8: Yes, they do

M: Even when such a pig is killed, they just eat it?

All: *Murmur in agreement*

M: There is nothing like throwing it away, not so?

All: Yes

M: Why do you think such meat is eaten?

*Silence*

M: Is it the one that tastes good?

*Silence*

C5: Sometimes, it does not taste good; people just eat because of *mandu*

Mandu = craving for meat

M: What do others think?

C2: Just not knowing that this meat is bad

M: Others?

*Silence*

M: Is there a latrine at your home?

C2: It is there

M: What about at other homes?

C5, C7: There is no toilet

M: How many have latrines at their homes?

C1-C4, C6, C8: *lift their hands*

M: Those that have, do you all utilise them?

All: Yes

M: Why do you utilise them?

M repeats question.

M: Why don’t you go in the bush?

M repeats question

C1: We use latrines to prevent diseases

M: What do other think people go in the latrines?

*Long silence*

M: Just going in there makes feel good?

*Silence*

M: What do you think makes people use latrines

C5: If they wish, can urinate or defaecate in there

M: What about those who don’t have, where do you go?

C7: In the bush

M: What goodness do you find in going to the bush?

C5: There’s no goodness

M: What do others think? Even those with latrines may also go in the bush?

C1, C2: Yes

M: What is the badness of going in the bush?

C1: Goodness of going to bush, one doesn’t get toilet smell

M: Others? My brother? *(Addressing C2)*

M: What is the goodness of going to the bush?

C4: Nothing

M: What do you concerning the goodness of a latrine?

C4: It prevents diseases

M: Anything else?

*Long silence*

M: What about family members, where do they go, in the latrine or the bush?

C5: Others go in latrine, others go to the bush

M: Why do you think they go in the latrine?

C4: To prevent diseases

M: Others?

C1: Others don’t know how to use latrines, that’s why they defaecate in the bush

M: Others?

*Long silence*

M: What about the issue of washing, what do you think about it? Do you see it as a good or bad thing?

C3, C5: It is a good thing

C1: A tidy person looks good

M: Others?

C8: To protect one’s self

C3: When you don’t bathe, you have lice

C5: You may not grow healthy if you don’t bathe

M: Do parents find any difficulties in telling you to bathe now?

All: Yes

M: How many times per day do you bathe? Bath once or thrice

C5: Others once while others thrice

C7: Others twice

M: Do they manage to bathe three times?

C5: Yes, they do

M: Where does the bathing water come from?

C6: From the well or a borehole

M: What about washing hands, how many times should we wash our hands?

C5: Many times

M: Now, what times do you wash hands, before doing something?

C5: After coming from the latrine… washing with soap and water failure to which you can forget that you had been to a latrine and eat foods like that

C4: When you want to eat *nshima* you wash your hands

Nshima = maize meal pap

M: My sister? Before eating groundnuts, can you wash your hands? *(Addressing C8)*

C8: We don’t wash before eating groundnuts

M: Why don’t you eat without washing hands? What do you think you don’t wash hands?

*Silence*

M: My brother? *(Looking at C1)*

C1: Because groundnuts do not have covers; you just break it and throw nuts in the mouth

M: What about those occurs when you break the groundnut and put the nuts on the palms before eating? We do not always put groundnuts straight in the mouth, do we?

C1-C3, C8: yes

M: So why don’t we wash our hands?

C5: Others just ignore and don’t wash hands

M: Others?

C1: We sometimes forget to wash hands

M: Is washing well?

C5: Yes, it is good

M: Why?

C5: Protecting one self

M: Do others think washing hands is good?

C1: it is good because you may not easily contract diseases

M: What do other think about washing hands, my sis? Is it a difficult thing? *(Looking at C7)*

C7: Yes

M: Is it a difficult thing? Even not washing before eating *nshima* is ok?

All: No *(in unison)*

M: What good do we see in washing hands?

*Silence*

M: Do we know the disease of worms?

*Silence*

M: Worms?

C5: Some of us don’t know it

M: What about abdominal *snakes*?

Abdominal snakes = worms

*Silence*

M: Have you seen them?

C1: Haven’t seen it

M: Have you heard about them?

C5: We have heard about it

M: What did you hear?

*Silence*

M Repeats the question

*Silence*

C1: Just told, some of us eat with soil, that eating soil can lead to worm infestation in abdomen

M: Have you heard of anyone expelling a worm?

C5: Yes, we have heard

M: Where did you hear that they came from or how they got out?

C5: When eating

M: What did other hear?

C2: From the nose

C1: Vomiting

M: Where do you think such worms come from?

*Silence*

C5: When one has a vegetable garden and a child defecates nearby, flies can sit on feces then onto vegetables. When a human eat such vegetables, then they can contract worms.

M: Where do others think worms come from?

C3: From termites

M: Others?

C1: From pigs

M: Where do others think worms come from?

M: Of those people that expelled worms whether by vomiting, through the nose or defaecation, what do you think were their symptoms/complaints?

*Silence*

M: What was paining, or what was troubling them?

C4: Abdomen

M: What was happening in the abdomen?

C4: The abdomen was painful

M: Others? What were their complaints?

C5: Hunger

M: Others?

C1: Some of us were feeling something moving in the abdomen and then experience abdominal pain

M: Do you think this disease of worm infestation can be prevented?

C1: Can be prevented if one goes to clinic

M: My brother and sister? Can this disease be prevented? *(Looking around at group)*

C5: They are preventable

M: Do you think they can be treated? Does such an illness get treated?

C5: Yes, they can be treated

M: What are they treated with?

C4: With medicines

M: Where are these medicines found?

C4: At the clinic

M: If you had such an illness, can you be fearful?

C1: I would fear because I may feel I can die

M: What do other think, if you yourselves had worm infestation, would you be fearful?

C5: Yes, may be afraid as I may not have suffered from it previously and I may think worms bite the intestines

M: What do others think? Can you be happy having them?

*Silence*

M: My brother? *(Addressing C2)*

C2: I may not feel good/well

M: If worm infestation were on you, how would you feel?

*Long silence*

M: Hmmmm?

C1: Can have abdominal pain, so can just be suffering from the pain

M: Are there diseases greater or severe than worm infestation?

C5: Yes

M: What kind of diseases?

C1: AIDS

C5: Syphilis

M: Others, what kind of diseases can we think of? Sis? What are the diseases greater or severe than worm infestation? *(Looking at C6)*

C6: “makimum”

M: What was that?

C6: “Makimum”

M: What kind of disease is “makimum”?

*Silence*

M: What do others think are the severe diseases?

C6 says something inaudible, but sounding like malaria

M: What are the less severe diseases than worm infestation?

C5: Malaria

M: What do others think?

M repeats original previous question while addressing C7

C7: Headache

C8: Malaria

C1: Sneezing, flu

M: Are there people in your village who have worm infestation?

C5: They are there

M: Are they there?

C5: In the areas where we live

M: In your daily life what would be the difficulties you would encounter with having worms?

*Silence*

M: Have you understood the question?

*Silence*

M: I said, in your daily life what would be the difficulties you would encounter with having worms?

C1: Death

M: My sis? *(Addressing C5)*

C5: Not getting satisfied as worms are the one that eat food you consume

M: How can others add on?

*Silence*

M: Do you know epilepsy?

C3: Yes

M: Have you heard of it?

C1: Yes

M: Have you ever seen someone fitting?

C1: Yes

M: Do you think epilepsy is a disease of animals or humans?

C3: Others can be from black magic

Black magic = witchcraft

M: Others?

C1: In people

M: Where do you think this disease come from? If someone is fitting, how do you think the disease came about?

*Long silence*

M: Is the question difficult?

C1: Can get from pig if you eat pork

M: What do other think?

*Silence*

M: How does epilepsy come about in humans?

*Silence*

M: How does one get this disease when you see them fitting? *(Demonstrating with hands)*

My sis, my brother? *(Looking around at group)*

M: Do you know of anyone suffering from a disease of fitting?

*Silence*

M: Are they there around your area?

C5: Hmmm

M: Are such people there?

C5: Yes

M: Where do people who fit get the disease from?

*Long silence*

M: You never heard anything?

C5: Hmm

M: They just started on their own? They did not know the cause?

C5: Yes, they did not know

M: Can this disease be prevented?

*Slight silence*

M: Can fitting be protected?

C1: Can be prevented

M: My sis? *(Looking at C5)*

C5: Maybe by stopping eating pork

M: Do people get healed?

C5: They get healed

M: Have you ever seen people who got healed?

*Silence*

C1: If bewitched one doesn’t get healed, if otherwise others get treated

M: Does the disease get treated?

C5: They get treated

M: My sis, does epilepsy get treated? *(Addressing C8)*

C8: They get treated

M: The medicines for curing them, where do they come from?

C3: From clinic

C5: Others get medicines from traditional healers

M: Others?

*Silence*

M: Do you think it is fearful to suffer from this disease? Epilepsy?

C1: Yes, because sufferers when fitting can fall on fire or in a stream

M: Others, what do you think? Is it fearful having epilepsy?

C1: Yes

C8: When drawing water from a well, once fitting one can fall inside

M: What do others think? About epilepsy. My brother? *(Addressing C2)*

C2: If one goes swimming can drown once fitting episode comes

M: Are there diseases that are severe than epilepsy?

*Silence*

C5: There are there

M: What kinds of diseases?

C3: AIDS

M: Others?

C5: Gonorrea

M: Others, is there any severe any disease that is severe?

C7: Cholera

M: What about the less severe disease?

C1: Eye sore

C7: Malaria

M: In your daily life what would be the difficulties you would encounter if you had epilepsy?

*Silence*

C1: Feeling dizzy and falling

M: Others? In your daily life what would be the difficulties you would encounter with having epilepsy?

*Silence*

M: Can you be wandering about?

C4: Can occur anytime, that’s the problem

C1: Can fall on fire

C5: Sometimes one can get confused, not knowing where one would be going

*Silence*

M: If the government brought laws in order to prevent worms and epilepsy; If they say all of those with pigs in your village need to be confining them, not allowing them to go outside but stay within a kraal, what kind of difficult can be there?

C4: Nothing

C5: There could be because they can still kill and eat it

C1: Even in the event that such laws are enacted, people might not build kraals. Instead can be dilly dallying and wandering finding time to go and collect poles from the bush and impaling them in the ground

M: What does other think of any difficult that can arise?

*Silence*

M: Sis, what problems can be there if they said you be confining the pigs? What problems can be there?

C1: It would be a struggle to find their food

M: If they said all of us in the villages should construct latrine and use them, what difficulties can arise?

C5: Some of us cannot manage to build latrines

M: Others? There sis, what could be the problem in building latrines? *(Addressing C8)*

C8: Some of us can manage, but there are others who may fail to do so

M: Can everyone manage to be using the latrines?

C8: Others can be still be going in the bush

M: Although latrines would be there, they would still use the latrines?

A few of them answer in the affirmative

Intervention (Andrew): Why is that so?

*Silence*

C1: Digging the latrines

R: One can construct a latrine, but may still not use it. Why is that so?

C1: Others don’t use latrines for fear of falling inside it

M: Sis? *(Addressing C5)*

C5: Others don’t know how to go and use latrines

M: What about you? *(Looking at C7 and C8)*

C7, C8: *Both just smile shyly*

M: What about if they say pork whether you buy it or slaughter it, you must boil it, what difficulties would you find?

C4: Nothing, as we would be protecting ourselves against diseases

M: Others, what difficulties can you find in boiling meat?

*Silence*

C5: Nothing

M: What about a law that dictates that all meat from slaughtered animals must be inspected, what difficulties would you find?

C1: Others may have problems with transportation because they don’t have it

M: Taking to where examination would take place?

C1: Yes

M: Others, what difficulties can be found concerning meat inspection? Can there be a problem?

C1: Others may cleverly announce that they were going to have meat inspected but turn back before having meat inspected. On their return, they can lie that the meat was inspected when in fact not

M: If meat inspectors condemned the meat, how would the owners feel?

C5: Others may not throw away the meat because they are bought it for sale and would lose lots of money they spent to acquire it

M: What do others think?

C1: One feels stingy to throw it away especially if the pig is a big one. They would say we will still eat even if we may die, so be it

M: If they said all vegetables including *Chiwawa* should be washed, what do you think would be the difficulties?

Chiwawa = pumpkin leaves

C4: No problem

M: What about the issue of *Chiwawa*?

*Silence*

M: Does *Chiwawa* taste great if washed?

C4: Yes it does

M: Does *Chiwawa* taste great if washed, sis? *(Addressing C7)*

C8: No (*shaking head indicating no)*

M: What makes it bad?

C7: They say that it dries up

M: Did you yourselves feel that it got dry?

C7: Hmmmmm

C1: The hairs on *Chiwawa* tend to prick when eating

M: When you wash it?

C1: Yes, it does not get well cooked

M: Others, the women? How do you see it? *(Looking at the girls)*

C8: It does not taste good when it is washed

M: Can it then be a problem, people may not accept this?

C5: Others love to wash it

M: What about hygiene, washing and bathing, if all of us need to bathe and wear clean clothes, what problems can there be?

*Silence*

M: At bathing, how do you see it?

C6: Nothing

M: Can you manage to bathe?

C1: Sometimes when the weather is cold, one may fear bathing that if they bathe they can get sick

M: What problem do others see?

*Silence*

C5: Some of us it may only be laziness to bathe

M: What about all those with worms or suspected to have worms are supposed to be treated, what kind of problems would arise?

C1: Nothing. In fact can feel good that let my life be protected

M: What about others; if we came to your villages and announce that we have come with dewormers. Come boys and girls, what would you think of us?

*Silence*

C5: Nothing, because once you get the drugs and drink them, the disease can be treated

M: What if the said, all pig need to be vaccinated or treated. We all know what vaccination is?

All: yes

M: let us all take the pigs for vaccination, what difficulties would there be?

C1: If there are free range pigs, catching them might be a problem as doing as it can be difficult

M: Others?

C8: Others are just lazy

M: Others?

C2: Transporting them can be a problem

M: Others?

C2: Transportation can be a problem especially if pigs are many

M: What if they said, you should be paying a small amount of money to vaccinate pigs what do you think would be the problem?

C2: If they don’t have transport, or transportation money they would also think of looking for money for vaccination and in the long run may just leave the whole exercise

M: What do others think?

C8: Others may not agree and think that instead of losing money to vaccinate the pigs I would rather leave the pigs as they are

M: Others?

*Silence*

M: I believe we have finished our programme

Most agree

M: From the beginning, we had a discussion concerning pigs, we talked about the goodness and badness of pigs, why we keep pigs, what do we feed our pigs, issue of latrines, issue of washing hands, issues of bathing our bodies, issue of intestinal worms, issue of epilepsy, and at the end we talked about how we can protect against worm infestation and epilepsy, Not so?

A few respond in the affirmative

M: We said we can protect the pigs if they are confined, not them getting to the outside, if we build and use latrines, we boil meat, we wash vegetables, and we bathe properly, we vaccinate our pigs and we ourselves do vaccinations. Have we understood?

A few say yes

M: Are there any questions? Ask, or even adding on, you can do so

C2: If someone goes to the vaccination centre and lacks money but is willing to have pigs vaccinated and requests those doing it to decide what to do

M: Have you understood the question?

C5: Hmmmm

M: What do you think he should do?

*Silence*

M: Have you understood what he has asked?

C8: Yes

M: What should he do?

*Silence*

C8: They can help us

M: What do others think?

*Silence*

M: What do others think?

C5: They can help knowing that the pigs need protection

M: others...
