# Supplementary material for: Why Latrines Are Not Used: Communities’ Perceptions and Practices Regarding Latrines in a Taenia solium Endemic Rural Area in Eastern Zambia
Source: PLoS Negl Trop Dis. 2015 Mar 4;9(3):e0003570. doi: 10.1371/journal.pntd.0003570 (PMC4352092; doi:10.1371/journal.pntd.0003570)
Supplement: S1 Dataset — (ZIP) [file pntd.0003570.s001.zip › FGD transcriptions_Zambia-2010/Mtuna_ Dr Phiri/Men_Mtuna_05-08-10.doc]

**Title: Focus Group MEN**

**Date:** 05/08/10

**Site:** Kakiwa Rural Health Center

**Village:** MTUNA	**Location:** Petauke district, Eastern Province, Zambia

**Duration (total time video):	84 min	- Start:**	14:57	**- End:** 16:18

**Participants:** 8 men

**Informed consent:** signed and available

**Moderator:** Mr Emmanuel Mwanza	**Recorder:** Dr Andrew Phiri

**Transcription/translation:** Dr Andrew Phiri

**Abreviations: Mod: Moderator, R: recorder, I: intervener, C: children, W: women, M: men**

**Disposition (from the left of the Moderator):**

**M**

M1

M2

M3

M4

M5

M6

**R**

M7

M8

Age composition:

M3: the oldest (60 yrs)

M2-M6: the youngest

All the rest are middle age

Typology:

M6: quiet/shy

M3: Dominant/funny

M5: dominant

**Comments about participants:**

M1 is the headman.

**Group dynamics:**

**Text:**

*Introduction:*

Mod: Well, I am very grateful that you have all come here at Kakwiya. What is needed is that we need to speak up as we are doing the recording of the discussion. We must also do it in free way. Before we start we need to know each other

Introductions of the participants, Moderator and recorder is done

Mod: Why we have called you is involving the day-to-day life we experience in our village, the life of pigs, how we keep them.

*Discussion*:

Mod: To begin with, are pigs useful in the village?

All: Very useful

Mod: If pigs are useful, explain what is their goodness?

M5: Various ways of help. If there has not been a disease outbreak, we sale off some pigs to have school fees. When a child is in trouble and has been fined, the money realised from pig sale can be used to pay for such, in times of hunger as well

Mod: Others who can add to the goodness of having pigs? Like what the gentleman explained

M3: Goodness of pigs is this: Breeds many piglets and within 2-3 months can reach market weight. Even what this one has explained pigs help with school fees, for agriculture help. All that is goodness of pigs. Another thing, taste of pork as is very delicious *(speaking in your English)*

*Laughter*

M3: Pork is very good, one can make pork chops

Mod: You have explained very well, is there any badness of keeping pigs, Mr M6?

M6: Is bad if owners have not kept them well. There’s need of having a kraals and latrines must be there. In that case, the pigs will be well taken care of

Mod: What badness do we see in pigs in our village? *(Addressing M8)*

M2: Pigs don’t choose what to feed on, they like washing in dirty water, and generally do not take good care of themselves

Mod: Who else can add to what has been explained?

M1: If you do not have a latrine, they follow a person in the bush. Just after one finished defecation, they quickly eat the feces. Even we say pork is very good to eat, it can also bring us diseases. This is the badness that I personally find

Mod: What can other add on the badness of pigs?

*Silence*

Mod: What do we find as badness of keeping pigs?

M2: Someone told me that you need to test pork, If not well cooked, it can be found with a problem. To me I was told two things, take one piece of pork and any other type of meat. If you put a piece of pork in half-full bottle of coca-cola drink, the drink foams and spills over. I was told that happens because that meat has something inside it. We vehemently disagreed with that person until we went to try it. Therefore, even if you cook such meat or eating it not well prepared, one can have some problems with it

Mod: Uuh Hmm, so you have explained that a pig eats bad things etc. What is the danger to life?

M7: I heard that it brings epilepsy

Mod: Others? What is the danger to a person’s life?

M5: Contracting *vifuwa* from pigs

Vifuwa = coughs

Mod: Others?

M2: They also transmit intestinal worms

M1: Sometimes diarrhoea

M3: If a pig gets very fat...

*Laughter*

M3: If it has too much fat and one consumes it that person can suffer from diarrhoea

Mod: How do we rear the pigs? Are they free-roaming or confined in within fences? How do you truthfully keep these pigs?

All: Free roaming

M1: They do not have fences

M8: They are just kept outside. One only feeds them with maize bran

M5: That’s the way we keep pigs here in the village. We keep them outside. That is why it is easier for them to follow people without latrines in the bush. That’s the rearing in the villages

Mod: The rearing without fences?

M5: Yes, without fences. Sometimes, they can have fences, but that is usually when the owner has just purchased new pigs to make pigs get familiar with the surroundings. Once they get used, they are let out and only fed. The owner confines himself at night, but he doesn’t know what the pigs feed on at night. Thy as well be feeding on undesirable things unknown to owner.

M8: In fact, many aren’t fed at all. They just roam about picking whatever foods they can manage

*Pause*

M8: When severe food shortages arise, pigs can wander about

M5: During this time, they can manage to pick up something, but we are faced with severe hunger, many pigs just pick up and eat grass only.

...Even the taste of pork is not really there. When someone buys, they only purchase for about K50,000 even for pig that would cost K200,000 because it would weigh less. Those are some of the problems we face

K50,000 = about 10 USD; K200,000 = about 40 USD

Mod: Why don’t owners of pigs make kraals and confine the pigs?

M5: That’s unfortunately the tradition we have copied from our parents. This trend has continued here in the village. For those home where parents used to confine pigs, goats etc, it easier for the younger generation to continue the trend wherever they go. It is the same for those whose parents were not confining. It looks like people feel it is much easier to let the pigs roam than think of the trouble of looking for wood to construct kraal. Sometimes, when you make a kraal, women use wood support for kraal for cooking as firewood. Instead of arguing with them, I let it happen and think of not building another in the future.

Mod: So the women destroy the kraal which you would have built?

M4, M5 and M7 agree

Mod: Don’t you correct them and say that what they do is wrong?

M8: Many times such happens if there is a funeral at household. After restraining the pig from the kraal and pressured by passing time, they may decided to remove wood from the kraal. They use such wood to make a fire

M1: Many times as the pigs reproduce a lot, the owner can encounter difficulties in feeding them. They may themselves be having trouble finding their won food as a family and therefore to avoid a similar situation with their pigs, they would rather let them roam about to fend for themselves

Mod: What problem do you observe with pigs that are free roaming?

M4: They find themselves eating indiscriminately, and may thus find themselves sick or dying unknowingly

Mod: Others, what is the badness or dangers of pigs free-roaming without being confined?

M7: We have already said some things. For example, if there is a disease outbreak at my household and one pig has been slaughtered or dies, and this M8 pigs come in contact with the water used to prepare the diseased pig, they too can easily contract disease

Mod: Now, for those pigs that are free-roaming with kraals, you said it started with your parents; how would identify your pig as your own in the village?

M7: It is known

Mod: How is it known?

M1: Some perform ear notching whenever a pig is born

M7, M8 agree

M5: It becomes a problem when a pig is free roaming; Similar looking pigs may mistakenly be slaughtered. If one of my pigs is slaughtered by someone else in that manner far away from my house, I may not realise it then. It can take me even a month or a week to know that I have not seen my pig. I may not even go there to investigate believing that maybe it was stolen. I may lose such a pig because we leave them to sleep outside

Mod: Are there others who can add? The badness of pigs sleeping outside?

M2: When they are many, they reproduce. In that case, I would sell some to buy maize bran. However, when they are many I am not know if a pig has been stolen or has died. Even when I would need to slaughter it, such as at a funeral, it may be difficult to find a pig. Even calling it to be fed, a pig can run away because it is not used to being fed

M8: To add on, a long time during times of predators such as hyenas, any type of livestock such as pigs and goats was not kept outside, they were confined. Nowadays, we see goats and the like sleeping outside... even Chickens,

*Laughter*

Mod: Now that hyenas are no longer there, people are free?

M8: Yes, people are free. But not so long ago, there were stories that hyenas were back. Ooo, people were even confining goats in their own houses

Mod: Sleeping with them in the same hose?

M5, M8: Yes

M8: Confining them in their in kitchens fearing that hyenas can get them. Now when people heard that hyenas had been killed, they let them loose again, not even bothering to build kraals for them

Mod: We have explained the goodness and the dangers of keeping pigs. Now headman, what are the problems or difficulties of keeping pigs? *(Addressing M1)*

M1: In keeping pigs, the problems that are there; I hear those things eat a lot and so need a lot of food to feed them

Mod: What are the other difficulties?

M8: Many die from disease outbreaks. You can be left with one pig sometimes with nothing at all

M3: Number 1) Many of the pigs are very thin, and don’t grow much because they move about a lot. Why is it that our pigs are very thin? We don’t have drugs to give them, that is our difficulty. 2) We don’t know good pig management practises e.g. building good kraals and confining them inside. Also buying feed for them so that they eat together in the kraal. When we do that even buyers would be attracted by how well we would be looking after the pigs. Costs incurred in obtaining pig feed and funds for food are scarce. However, we need to depart from parental management inheritances, we need to build kraals and confine pig together with buying feed for them or even giving them pounded maize or maize bran. The world has changed

Mod: What duties are there for men, women or children are there when taking care of pigs? M2?

M2: If we have a program of keeping pigs at my home, we first make kraal before purchasing pigs. When a pig is purchased, it must be placed in a kraal. Just as we are used as humans eating porridge in the morning, *nshima* in the afternoon and evening, we must also think of the domesticated animal. Women should remember to give feed to it. When a kraal is damaged it is my duty to repair it.

*Silence*

Mod: uhmmm, who else can add on?

M1: Men should apportion duties; Children to lock and open kraal, my wife to feed and myself building and maintaining the kraal. I need to know and investigate where piglets are coming from in order to repair those places

Mod: My brother, how would you know that your pig is sick? Especially that the pigs are free-moving? *(Addressing M4)*

M4: It is known by the way it carries itself; Walking with lowered snout, poor hair coat, even walking it doesn’t do it freely

M3: Shaking or trembling limbs *(demonstrating with his hands)*

Mod: What are the symptoms, M6?

M6: It is visible in that it doesn’t wag its tail, the tail is just lowered. It can be lying under the food storage sheds (granaries)

Mod: What can others say to know that this pig is sick? How do you see it?

M5: When a pig is sick, it is known in many ways, lagging behind other pigs, remaining around home or kraal and separating it from others, lying still/inactive. Even after I have gone and come back from chores, I would still find it in the same spot. After examining it, it only lets out a groan (demonstrates). It doesn’t move about. It’s one way of knowing that this domesticated animal is unwell if it does not move about. If you see it not moving for about two hours, is one way of knowing

M8: You can even see that it has a sunken abdomen, and a change in breathing *(demonstrating both symptoms)*

Mod: It means a pig is unwell?

M8: Yes

Mod: Very well, what do you feed your pigs?

M5, M7: Maize bran

M5: When we have experienced lack of food, then pigs roam, and fend for their own food… just grass...Maybe even following people

M2: Indeed we feed them maize bran. When there are food shortages and we have difficulties in finding maize bran, give them mangoes

M4: Sometimes, pumpkins when it is the time after harvesting

M8: Giving them pumpkins

M5: When we have pumpkins, it means we have survived the hunger, times are good

*Laughter*

M5: But before that time, before we have mangoes, if pigs could speak they would have told us already

Mod: How often do you feed your pigs from morning till evening?

M5: Here in the village when we miss them in the morning, we remember them in the evening. Similarly, when we feed them in the morning, they may go the rest of the day without being fed

M8: Here among the *Nsengas*, we only feed them once in the evening when putting them in the kraal. ...For those that have kraals. They just release them from the kraal in the morning and call them for food to entice tem enter the kraal

Mod: Is that the *Nsenga* way, not so?

M6, M7: Yes

Mod: For you to give it feed in the evening, what kind of food does it eat before then?

M3: They just look for food, that’s why many are thin. Not eating most of the day, they only play in the mud puddles

M5: They burrow in the fields looking for worms so that they can keep themselves well

Mod: Do you have family members who help you with keeping after your pigs?

M5: Like giving maize bran

Mod: Do such a thing happen?

All: Not really

M8: What happens is that when you have many pigs, and you have a relative who can give you maize bran. In agreements we can agree he brings maize bran for about a year and in return I can give him a pig.

M4: Another thing, when you buy a small pig that is pregnant, you agree with someone that you provide maize bran and when the sow gives birth I would give him a piglet

Mod: So that’s help from outside?

M7: Yes

M8: Anyone else can make such an arrangement

M5: Not anyone giving himself to help? No. Not even a relative can do that

M8: That one jus need to benefit

Mod: Anyone else who can add on that? Is there anyone else who helps?

M7: Only those with whom you have made an agreement with. They give you maize bran in exchange for piglet when they are born

M5: In addition you see. Families are different. If I have pigs and when I slaughter one, I can give my relatives some meat. When they deem fit even when you yourself has forgotten you gave them some pork, they in turn can give me some maize bran in future

M8: They can give you maize bran

Mod: Very good, where do you slaughter the pigs in your village?

M3: They just kill anyhow and anywhere. Wherever it is caught and restrained. It is just dragged near bush

Several of them speak at once: Killed anywhere, no specific place

M3: Near bushes, so that the same grass is used to burn off the pig’s hairs

M5: Killed just there

M3: There is no special place for slaughtering pig

Mod: So you just kill it anywhere?

M8: You can’t necessarily kill it anywhere. What is usually done is to Kill pig at the edge of the yard, burn off the hairs and prepare it. When pieces are cut that is when the meat is brought to the house

Mod: Uhmm! So, who makes the decisions concerning slaughter of pigs, so that we may get relish or anything in relation to that? Who has authority to make decisions?

M7, M5: Men at home

*Silence*

Mod: How is such authority with a man when he fails to construct a kraal?

M3: That, making a ruling whether a man or woman… What makes the slaughtering of a pig is hunger in a home, lack of clothing, so what would we do? Or when there is funeral, it is only then that in agreement with spouse, you make a decision. It is because of suffering that a pig is slaughtered

Mod: Who has overall authority?

M7: It is a man

Mod: What about children, you said they are the ones that confine pigs at night. Don’t they have a say, like when they say if they have a craving for meat?

M7: Kids can only suggest, but decisions have to be made between myself and my spouse

M3: Ruling by kids can only be when kids have educational needs, we can say let us kill this pig for the child’s needs they too can suggest. They would have authority in that case

Mod: Truthfully, when a pig is slaughtered, do you bury the intestines?

All murmur “No”

M5: Can give dogs. Even us people we eat the intestines

M2: We get the intestines...

Mod: Say truthfully...do we throw away the intestines?

Several say “No”

M8: Truthfully, we throw them away

M4: We don’t throw them away

M3: It is because of poverty nowadays that we people eat intestines. Intestines were not supposed to be eaten especially of free-roaming pigs. They follow people in the bush. But nowadays because of craving for meat, people have tended to eat intestines. It is not required to eat them

M3: We don’t need to eat intestines of pigs because of its feeding habits. Because of hard times, such behaviour is tolerated. Never to happen in old days

M8: We don’t throw away anything

Mod: Do we throw them away?

M5: Intestines are not thrown away. For those that keep pigs, when they slaughter them they give intestines to kids who clean them and boil them. Afterwards, we can eat the rest of the meat with them. Even at the Boma, these same intestines are the “shockers” *(indicating braaing stand)*

Mod: What about you M2, what do have to say about intestines? You give children the intestine and you elders eat meat because you say it is the first food for them. How is it, are intestine thrown away?

M2: Lifestyles differ. Others give children, others don’t. They ask children to get them grass in exchange for intestines. In the past adults used to grade them, they used to only take small intestines and throw away large ones. Nowadays, there is no grading, they just get and cook them. Nowadays, it is not that it is children, even us adults. Dogs were the ones who used to consume them.

When we slaughter a pig, we first start with the intestines. We eat together with leftover *nshima* of previous day

Mod: So, M1, intestines are not thrown away?

M1: In the past no one used to eat them. When we slaughtered a pig, we could only get liver and the like and throw away the intestines. Dogs were given these to eat. The way others have said nowadays, intestines are “graze”.

Graze = meaning very good food

M5 and M7 laugh

Mod: Are intestines thrown away? *(Addressing M8)*

M8: Hmmm. We don’t throw them away. Even us who don’t keep pigs our kids wait for these intestines. Now, the pig owners just say “these intestines are for my children. You there kids are just wasting your time waiting for them”. We all just come back empty-handed. Children are the ones to be given intestines as they are starters.

Mod: Well, they are not thrown away. How do you see pigs eating human feces? What problems do you see with pigs eating feces?

M5: When you grow up seeing such behaviour and your father once the pig is slaughtered eat it. For you as a child to just refuse may be difficult. So we are used. Even if it eats faces when it is alive we don’t mind but when it is slaughtered, we still eat it. We easily go over it

M8: Even when it eats feces but when it is being sought after for slaughter and it is found in the act it is killed there and then. It is relish

Mod: What do others think about pigs eating feces?

M1: What others have said, even if it is found feasting on feces, when you slaughter it you may not clean it as thoroughly as possible. Some dirt still remains therefore you may still be eating diseases......That’s the badness

M3: In addition, a pig doesn’t get satisfied. They eat like that because they are not fed. In their eating habits they get diseases and die. When pigs die, it means you the owner gets even poorer.

Mod: In addition, hat is the badness of pigs eating feces?

M3: If I have bloody diarrhoea, and that pig eats the feces. The following day we say lets us slaughter this pig, for sure those diseases are transmitted to those children who consume the pork.

Even us who have consumed the pork, it means we have contracted such diseases. It is because of that pig that has brought the disease. That’s the badness of pigs

Mod: Are there others who can add on?

*Silence*

Mod: ...The badness of pigs eating feces?

M2: Feces are things that are not supposed to be eaten. Because that is waste or that has been thrown away. It is because pigs are not well kept that s why they do that.

Mod: What are the dangers that you see to our health, concerning pigs eating feces?

M7: When a pig consumes feces form a patient with bloody diarrhoea, they can contract and transmit such a disease to humans. All those that have eaten it get the disease.

Mod: Aha, the headman?

M1: For those people suffering from the illness that has come nowadays, when they are terminally ill they suffer from diarrhoea. If pig eats such feces and someone eats the pork they too can contract the same illness. It may not be surprising. That’s the badness of pigs

Mod: Can we confirm that if a pig eats faces from an AIDS sufferer who is terminally ill, and humans consume its pork, can they suffer from disease?

M4: Yes we can suffer

M2: It can lead to others diseases, not AIDS. There are some diseases that it can give us depending on what bad things it eats, but not the disease of AIDS.

M8: Not AIDS, some other disease

M5: Because that is contracted through blood

M8: … Some other diarrhoeic diseases...

M3: One can contract a disease from anything. That disease the sufferer had, you also can get it

M4: I agree with that, too.

The group is unease concerning the two opinions

Mod: Is there any times of the year when pork is frequently consumed?

M1: December, Christmas days

Mod: When do others eat a lot of pork?

M2: During these times, 1) because we have money we can buy pork, sometimes if looking it. 2) Also during disease outbreaks in the hot season. They die in large numbers and so owners can give you some

M3: During these times, we have many diseases... funerals. There’s pork at every funeral. Majority eat pork at funerals, because they are slaughtered a lot

*Laughter*

Mod: Others who eat pork can add on?

*Silence*

Mod: Which days do we eat pork? Can others explain?

M6: during these days *Kajinya* or “shockers’” is common

Shockers = method of preparing pork where it is fried, and semi-cooked on a wide home-made pan at a roadside by young men

M8: Just like M3 has said. At funerals pork is eaten a lot. However, it is boiled for a short time

M6: … But the meat does not get well-cooked

M8: … Just eating raw pork. Because it at a funeral, people just eat

M3: Let me say people eat pork throughout the year. Every month pork should be eaten, up to December

Mod: When someone is selling pork, or you have slaughtered it yourself, what features do you pay special attention to when examining the meat?

M3: Whether the pig has died on its own or not

M8: Presence of fat

M3: Appearance of an animal that has died on its own is not mistakable. The meat is darkish

Mod: Other things that you see? *(Addressing M2)*

M2: Some time ago when I was young and used to go to school, my elder brother used to keep pigs. There were those from the clinic who used to inspect meat. One time we slaughtered a pig to take to the Boma, but the pork has cysts *(indicating on his body).* On another occasion, we had a pig whose pork had round things in it but not as plentiful as the previous one. Even when eating, it felt with popping sounds in the mouth *(imitating eating pork with cysts)*

M5: Those are *nsembe*

M2: That meat had cysts all over it. If the meat inspector told us that meat needs to be burnt, but us humans do not easily follow such instructions. We made a plan and managed to sell it to others

*Laughter*

Mod: What other things do you look for before you buy it?

M2: Fat

Mod: Others?

M6: In that manner, fat. An animal that has died on its own looks pale. When it is fat, one is attracted

I (Dr. Mwape): Why is it that you follow presence of fat?

*Laughter*

M6: To have an idea that where that pig comes from was well taken care of

I (Dr. Mwape): … Or that it eats a lot of feces?

M2, M4, M5: Yes, yes

M5: It is fat. The meat is delicious, gladdens the heart

M8: the heart is glad...

M5: …You don’t eat it complaining, but with strength even if you haven’t put on a scale just by looking you can say, “No, not this one”. You let him sale to others

M1: General appearance, when you just look at it, you even quickly remove money from the pocket without doubting. Its appearance, it’s fat

M7: Our village is big. If someone kill a pig from one part of the village and by the time it arrives at my place on the others side without been sold, I conclude that this pork is condemned

M8: Other pigs are those that were pregnant and die soon after giving birth. They know that people around the pig owners’ house know that history. The owners just burn the hairs and sell the pork far away from there.

*Laughter*

Mod: Far away so that you should not know that it died on its own?

All agree

Mod: Just looking at the meat you can see that it is reddened. They say we just killed it by ourselves

M7: It can be known

Mod: Does the meat from slaughtered animals get inspected?

All: No, no

Mod: Why is not the meat inspected?

M5: There is no one to inspect the pigs. If for example it was like what happens when they test for HIV at the human clinic, we come here to have our blood tested. If there was someone who would do that for pigs, for those that keep or those that do “shockers”, he/she would either come to our village or we would come here ourselves. That should be accompanied by a paper from the chief that that person has had pigs examined. Just like at the boma, if you have not had pork examined legally you also sell it secretly

M6: Do not see who inspects, there’s no one to do so

M2: Because of freedom in our villages, we kill pigs as we wish. If government directed or followed up on this, we would follow meat inspection. I remember when I used to roast meat, there was a directive prohibiting sale of pork at roadsides, instead they directed that properly constructed shelters be constructed for such a trade and that meat should be inspected. If such directives were still there, we would not find such meat displayed. Even it can look good; we may not know that this meat is diseased. Even for cattle, you can have a fat one but when inspected by a doctor, it may be found that the meat is not good...that cattle may not be allowed to be sold, it will be returned or they can confiscate that meat and burn it

Mod: If there was a law that you would require you to follow meat inspection before you slaughter your animal?

All: We can love it for the meat to be inspected,

M3: … To avoid diseases

M5: We can get used, to preserve our life or health

Mod: Very well, what are the benefits of having meat inspected?

M7: If a pig is examined and found to be unsuitable or have a disease, even the owner cannot have it slaughtered... just need to buy it drugs

Mod: How do you have pork prepared before it is brought to the table? What kind of preparation methods are used t have pork prepared? Boiling or what?

M7: Women just clean it and put on fire to cook it quickly *(gesticulating with hands)*

*Laughter*

M2: There are different preparation styles, and where we live there are different cooking systems. Others just boil and it is relish. Others briefly boil it followed by frying it. When we were at school and staying alone as boys, we would slice them into small portions and fry the pieces quickly before rushing back for lessons at 14:00.

M8: Truthfully, Moderator we are not even present in the kitchen. Therefore, only know and aware of the cooking hen food is brought to you at the table. You only experience the tenderness or toughness of the meat *(demonstrating if meat is tough)*. By that you will know that this meat has not been fully cooked

*Laughter*

M8: … Now to tell my wife that this meat is not properly cooked can spark arguments

Mod: The wife can ask query you whether she knows how to cook or not?

M8: Yes...its noise then

M3: 1) If it frying, the cooking oil must first be heated, cut meat in pieces, and place them in oil. When there is a frying noise, that when it tastes good

*Laughter*

2) If for boiling, the pork must be cleaned, and boiled with little water followed by frying. That’s when you prepare the soup. That’s not to mean over-cooking. It is not good like that

Mod: Not over boiling?

M3: Not over boiling the meat as if for me without teeth. The meat must be properly done, appropriately boiled and fried

M5: Must be well-cooked because of fear of diseases. Our pigs are not treated for diseases

Mod: So, big man, which meat is preferred by you? Goat meat, beef or pork?

M3: Pork

Mod: Aha..?

M3: There are any things that can come from a pig. First, if pork has a lot of fat, one can make pork sausages from it; Secondly, ... *(tapping his head)* … I have forgotten what I wanted to say

M2 whispers to M3: Is it that you put fat in vegetables?

*Laughter*

M3: That is the goodness of pork, pork sausages, and the other thing is what...

Mod: Who can help him?

M5: Regulations about boiling and frying the pork. What goes on at M3 may not necessarily be what happens at my place. His preferences may be totally different from my household

M3: they want to know how you yourself can have the meat prepared

M5: Here in the village the pork is dangerous and must be properly boiled and not coked for a short time like what M3 said. The disease we are talking about today concerns pork and make us fearful. If it is not properly cooked and a child eats it, that’s not good. That one can even see it oozing blood. You stop the child because you know that the child can face a problem

The pigs we eat are a danger and they do not have drugs to protect them against disease. A pig already has diseases

Mod: Where else do you see that pork is not well prepared?

M7: At funeral. Pork is cooked quickly. Faced with different chores women preparing food at a funeral tend to cook pork quickly and may not be well cooked. Chores such as grinding maize to produce maize flour for *nshima*

M2: Another thing ...That’s what we said about the goodness of kraals. If there is a funeral at a home where pigs are free-roaming, it can take a long time to chase and catch pigs for slaughter. Therefore our women are not given enough time for cooking before people have come from burial. For fear of leaving food uncooked they quickly boil it before mourners return to a home

Mod: Have you ever seen people not eat such pork?

M2, M7, M8: They eat

M7: That is meat

*Laughter*

Mod: Have you ever seen cysts in pork?

All: Yes, we have seen

MOD: How do cysts appear? Where do they come from?

They are shown a picture of pork with cysts

M3: Many times cysts come to pigs when they eat a lot of maize bran. They look the way it is depicted here in the picture. Maize bran is dangerous to pigs.

Mod: Is there a feed that cannot cause cysts in pigs? You mentioned the danger of maize bran, is there any feed appropriate for pigs?

M3: To avoid such a disease, there is need to buy pig feed

Mod: Buying breakfast mealie-meal?

M3: No. Pig meal that they mill nowadays. That is the best meal to give to pigs if you want to keep pigs very well

M4: Moderator, what causes cysts in pigs is *Masi* from *Kachasu* when the pigs eat that

Masi = brewer’s mash

Kachasu = illicit liquor

Mod: So, cysts are in pork. How do they affect you who eat pork?

M5: Meat has no taste, even if you were hungry, you get satisfied...

M8: Tastes like maize

M5: Not eating the pork freely…

M3: Just throw it away

M5: …Maybe children can eat because they not knowledgeable. Buy you the adult needs to tell them to discard it. That pork is not good when it has cysts

Mod: So M6, do people throw way meat with cysts?

All: Don’t throw away the meat

Mod: What happens then?

M6: Just reduce the pork market value when selling. If it is us, we just cut a piece for ourselves

M5: To make the pork taste better, we can just grill the meat. When the water content has gone down remarkably, we eat it

*Laughter*

M8: ...or eat it myself

Mod: Do we use latrines at the village?

*Silence*

M2: Sometimes we do, sometimes we don’t. It is just the differing offices from one to another (referring to different happenings at each household)

Mod: What goodness is there in having a latrine?

M3: Here it a topic about pigs. Avoiding feces to be reached by pigs is the essence of latrines. The goodness is that all the bad things from the intestines are discarded in one pit. It is not only good for pigs… Even flies won’t be around landing on suspended meats, they are avoided as they won’t fly to the bush looking for feces because those would not be there. That’s the goodness about latrines. They prevent flies and protect pigs. In that way, even diseases are lessened

Mod: Are there others who can add on?

M7: There is dignity especially for visitors. When a visitor comes at home and asks to use a latrine, you easily point it out. That person may be happy to go in the bush. There is respect at a home if there is a latrine

Mod: All of us from the village, do we have latrines?

M2, M3: We don’t have

M7, M8: I have

M5: I use my neighbour’s

*Laughter*

M1: I have only had a pit dug, it’s not yet complete

Mod: How do you use the latrines, in respect to different age groups, the adolescents etc? What happens M1?

M1: What happens is …

Mod: You have a child who has come of age or reached puberty and he is initiated....How do you the latrine… even when you have an in-law?

M1: There is need for distinction. Children need to have their own latrine…There are supposed to be two latrines

Mod: What is a bad thing that can arise from people sharing a latrine, even for example with an in-law?

M1: You may meet each other at the latrines when both are coming from different locations as you know how the intestines work, e.g. an in-law from the field and may be unaware that the in-law had gone to the latrine

M8: You may not be aware of it

M1: When you meet each other at the latrines, it becomes embarrassing

M8: It is like you have been undressed

Mod: Does it mean you would have been undressed?

M1: Yes

Mod: So from you explanation, it means the latrines are not used according to regulations?

M4, M5: Following up indeed

Mod: Do children use the latrines properly?

M5: Especially children do not know how to use latrines. They just mess behind the latrine. If they manage to enter the latrine, you just find a child has messed besides the pit opening

M8: So it is you the adult to clean up

Mod: When do you wash your hands?

M3: Washing hands… 1) after using latrine, even if there is no food around, you need to wash hands. Because when wiping yourself, some germs can come in contact with your hands, you may not see them. Secondly, before eating *nshima*, because we do not use spoons but our hands. The washing manner also does not mean using one dish for everyone when washing hands. Water needs to be poured on each other’s hands. In that way each other dirt does not affect someone else

Mod: What goodness is there in washing hands?

M5: The regulation is that what you have handled must be removed by washing. We need to place a 5L container of water and soap outside latrine to avoid placing dirt on cup or utensils if one was allowed to wash hands at the house

Mod: So, do you instruct your children how to use latrines?

M5: We tell them, but for them to follow instructions because of their age they may fail. That’s when you find that they have messed up. They even fail to clean after themselves and can only take an adult to clean up what they would have messed up

Mod: Do we know what intestinal worms are?

There are doubts and murmuring among the participants

M5, M3: I have seen

Mod: Where have you seen them?

M5: I have never seen such worms in humans but I have seen them in goats when it defecated. The worm as long flattened and coiled. That’s my explanation

Pictures of tapeworm proglottids in feces are passed around

M3: I saw such worms in my own child when I was living at the Copperbelt. After feeling pain at defecation, I saw worms coming out of different colours, and different appearance. When we went to the clinic was given dewormers and all the worms came out

Mod: Have you seen the worms *(in the picture)*? There are in feces...looking like caterpillars or a snake skin peel...Have you ever seen such?

They discuss among themselves, about worms and how they look like

Mod: For worms to come to humans, where do you think they come from?

*Silence*

M3: Because “they say” and we have not seen. That information is only relayed to us but in fact we have not seen them. All we hear is that even rape when it is not properly cooked... Not met, but rape itself brings worms. We just used those words

M2: The way he has said it...some vegetables can be responsible. I heard that somewhere they stopped them from eating *tomoria* at some time. After they were stopped from eating, the episode of worms stopped as well. Similarly, the type of cooking vegetables can have an effect, like eating vegetables every day can result in intestinal worms

Tomoria = a kind of commercial vegetable like rape

Mod: Do we have people suffering from intestinal worms in your village?

M5: May be they are there at the village, but we don’t know them

Mod: Do you think intestinal worms are problematic in the life of a person and what are its dangers?

M7, M3: Abdominal pain

M8: A child does not get fat

M5: Poor body condition and an enlarged abdomen

M3: A child has poor appetite

Mod: For you to know that a child has worms, how do you know?

M7: Take the child to the clinic

M2: Sometimes when the child defecates, we can see them in the feces. Other worms look like a small snake, a long one that even wriggles. Sometimes the abdomen enlarges

Mod: Is a disease more severe than intestinal worms?

M5: There are there

Mod: What kind of diseases are they?

M5: The disease they know call AIDS. For worms one can come to the clinic and get treatment and return to good health. Now for AIDS, they can give you drugs that will prolong your life 4 years, 5 years or up to 10 years you are ok, but eventually one succumbs and dies from the disease. Worms are lighter… AIDS had been very big for us

M3: The other disease that is found around this time is swelling of legs, swelling of body. This disease has been very common in these parts since I started living here. I have seen even deaths arising from this disease

Mod: Is this the disease that you are explaining to be more severe than worms?

M3: Yes

Mod: What dangers can affect someone’s life if affected by worms?

M3: It’s only to rush to a clinic to be helped

Mod: Can this disease be prevented?

M8, M2, M3: Yes

M5: As long as a clinic is there, and drugs are available

M3: I believe so because I have a personal testimony. My child who had worms got treated and even grew fatter afterwards

M5: Another thing that kills people in the village is that when a child is sick, people rush to consult a traditional healer. They even sell a pig to finance such a trip for a child with worms. Now when a child dies, they turn around and say someone bewitched the child. When a child is taken to a clinic they say this is worm infestation, and we realise that oooh, we had the other child destroyed for a similar disease

M1: Problem with worms is that what happens in human body is that when you eat food, energy lessens because it is the worms that receive the food. So that person doesn’t have good health because of presence of intestinal worms

Mod: Have you seen Epilepsy in humans? ... And where does it come from?

All: We have seen it before

Mod: Are there patients with such a disease?

M2, M4, M5: They are there

Mod: How do you think epilepsy is transmitted to affect humans?

M5: There are many different ways it comes. When body temperature is elevated in babies, the baby fits. There’s also epilepsy that comes without body temperature rise. Others it is a lifelong one. The one caused by elevated temperature is conquered when a child is taken to a clinic. The bewildering type is that one that has affected someone since childhood and all his life

Mod: Who else can add on?

M1: Epilepsy comes in many forms as what M5 has said. Others it is magic, someone has not been happy with how one has been living, bewitches you and you start fitting and falling; That is the type that does not respond to medication and you know its origin is human

Mod: What bad thing is there for someone to have epilepsy at your household?

*Silence*

M5: There are many things that get destroyed when epilepsy has come to a home

Mod: To add on..?

M1: To add on, there is difficulty in creating a budget to sustain home needs in pursuit of someone getting treated. Women when cooking can fall on fire. When she gets burned, it means that epilepsy won’t be treatable. That’s the major problem

M5: Another thing in addition… What surprises me is that instead of running to clinic, most victims are rushed to traditional healers and use of herbs. Does it mean that epilepsy is not treated here at the clinic? That’s what is backward with us… The use of herbs and emphasis placed on hearsay from people they hear tried the medicines… But a clinic is present here

Mod: What about M2? What problems have epilepsy?

M2: One can fall while riding a bicycle; the cause of disease is not known but I hear that pork and elated wild meats such as wild boar and warthogs are prohibited to be consumed. However, what causes and brings about epilepsy we don’t know

Mod: What about you M4, how did it come about of prohibiting eating of pork?

M3: The subject of epilepsy is not well known by me… I have not experienced it…

Mod: I meant my brother there? *(Pointing to M4)*

M4: After failure at clinic, maybe a child doesn’t get better. In that case victims are taken to traditional healers for herbs. Subsequently, they are stopped from eating pork, chickens and warthog. We follow those instructions because at clinic successful help would be difficult to obtain. It is only at traditional healers where prohibition is advised

Mod: Is there a more severe disease than epilepsy in our daily lives at the village?

*Silence*

Mod: What kind of disease?

M2: There is none

Mod: Well, can prevention be found for such a disease?

M5: this place where we have come is a clinic. Therefore, clinic personnel are free to explain to us if treatment and help is available

Mod: Yes, can prevention against this disease be found? *(Addressing M7)*

M7: At traditional healers place, they give you medicines but prohibit eating pork. This is where we need help

Mod: What about hygiene of fresh vegetables? M6, what goodness do you see about it?

M6: Caring for vegetables..?

Mod: Yes, before they are brought at the table

M6: Just in preparation and putting them in portions by the women *(saying this nervously)*

*Laughter (M7, M8)*

M6: We just receive the vegetables when they are cooked. By the time we see them they would have been placed covered in plates served together with *nshima*

Mod: Can fresh vegetables give us problems if we don’t clean them?

M2: They can give us

Mod: In which ways?

M2: When we make a vegetable called *Mfusha* and we place it for storage we don’t know what can get in touch with them, even crawling over the food. Even rats can bring diseases by crawling and urinating over the vegetable especially if not well cooked and eaten right away. They can give us problems

Mfusha = boiling and drying vegetable

M7: If eating vegetables that have been sprayed by chemicals (to kill pests) by vegetable garden owners. If not properly cleaned, they can give us problems… the same chemicals

M3: proper washing is needed to wash away such chemicals, snails, and pests. Those that cook, our women, need to wash vegetables in warm water… if not followed that can bring us diseases. The same chemicals for killing pests if not well washed can enter our intestines and cause problems

Mod: What is the goodness of body hygiene?

*Silence*

Mod: Is there any problem that can arise if there was law governing good body hygiene?

M1: there would no problem, because if one bathes very well, even malaria cannot be contracted

Mod: Are we as vigilant in taking care of body hygiene for our children as we do for ourselves adults?

M5: Children are somewhat troublesome. What an adult advises them against is what they do the moment you are not there. When you are there, they follow hygiene but you are not present, you find the child has done things that are according to their age because they are but children. A parent need to make regulations that followed such that a child follows suit instead of telling them “shut up” when they have already messed up

M8: It depends on the age of a child. What I know is that a child stays with the mother for a long time. For us who move about trying to make a living may not find time to supervise children

Mod: If there was a law that compels you to build kraals and fences and emphasizes that you confine your pigs throughout, what difficulties can be there?

M7, M3: There cannot be any problem

M3: Even our pigs can change and be looking good if properly fed and confined. Also they would to be vaccinated against diseases using drugs… Let us confine our pigs. As we discussed earlier on, these pigs bring a lot of diseases because they eat feces and many other bad things, and cysts found in pigs. All that can lessen

Mod: If there was a law compelling you to construct a latrine at every household, can there be any difficulty?

M7: Those who don’t understand can be a problem, they can find difficulties

Mod: If they said, we need to boil the meat properly, what difficulties can arise?

M2, M3: None

M3: That’s the problem as well

Mod: Is that a problem as well?

*Laughter*

M3: Not overcooking

*Laughter*

M3: Not overcooking, but reasonable boiling. As I mentioned earlier on, heat the oil and clean the pork very well and fry it. *(Imitating a frying noise).* Boiling it a bit and frying it until is brown. Not over cooking

*Laughter*

Mod: I saw a hand there?

M2: the goodness about boiling properly is that if well done you cannot find any problem in the meat

Mod: ehe? *(Addressing M5)*

M5: Pork is usually thick so it can be brown on the outside but not well done inside. Therefore, if meat has germs they will remain unaffected and trouble you the consumer. Pigs bring a lot of diseases such as swine flu and epilepsy

R: The question was, would you find any difficulties if you were advised to be properly boiling your pork?

M1, M5, M7: There would be no problem

M4: Cooking it well until ready, there would be no problem

Mod: Very good...if they said before a pig is slaughtered it should be inspected can there be any difficulty?

M5, M4: Nothing

M6: That’s when it can be better

Mod: Very good, if there was a law that compelled you to wash vegetables and observe good hygiene, what kind of difficulties can you find?

All: No problem

M8: The way you are asking is good, but if it were us doing the cooking, maybe...Now after these lesson to geo and tell a wife, she would respond that “have you just seen today that my cooking is not good? You are just difficult”

Mod: Can there be any problem if you were asked to have humans with worms were treated?

M1, M2, M5: There may not be any problem

Mod: What about vaccination and treatment of pigs so that problems are eradicated?

M1, M7: There cannot be any problem

M5: There may not anything bad. If anything the goodness that can arise is that we any not experience pig losses as pigs would not be affected by diseases. That is what we are appreciative about. If such interventions are available we are grateful if they can be availed to us

Mod: If they say we will give you that help but first let us be cooperative, you should also contribute something even a small one, what can be the problem there?

M3, M7: There *(at the same time)*

M3: Let me say first *(pointing to M7, but he ignores)*

M7: There, a small contribution should be defined. We can agree to the principle but may not know the actual cost… because we are still suffering

Mod: Yes, big man? *(Pointing at M3)*

M3: If government paid something and we also contributed something, that system would be good. However, that small contribution can be difficult to find for us because of our poverty. In case where one has one pig or many pigs, they may then just not have the intervention done. Even one tenth of total cost can be good

M5 agrees with M3

M3: Yes, it would be a great idea, but would depend on portion of contribution. For us to initiate such intervention can be a problem, only possible if government can initiate it. We don’t know the costs and logistics involved.

Mwanza summarizes at 16:18h and thanked all for attending

Mod: We have talked about many things since we started our discussion concerning keeping of pigs. 10 About the goodness of pigs and hwy we keep them, also discussed the dangers of pigs, difficulties we encounter when keeping pigs, learnt about worms, good hygiene about latrines, goodness and dangers of having or not having latrines, dangers of pigs not having kraals, and protection against worms, cysticercosis and epilepsy, we shared knowledge on treatment and prevention, we talked about goodness of washing vegetables as well as body hygiene and utilizing latrines, especially washing of hands; also looked at prevention needed in pigs.

I thank you all. Is there anyone who has something to add on concerning the things we discussed?... or indeed any question?

M3: I have a question. Some of us do not have pigs. Is there a method that we also can be helped in having pigs so that we keep them this way?

R: You have heard the question, what do you think?

M2: What we have learnt is not about who eat or not pork. All can be affected by the disease. We have been taught on goodness and dangers of pig keeping s that we know. If there can be a thought of providing pigs it can be good, but they have only come to educate us on pig keeping

M1: Can vaccination against pigs reach us here in the village so that our pigs are protected?

R: That help can reach you if vaccines have been mad e available
