# Supplementary material for: Why Latrines Are Not Used: Communities’ Perceptions and Practices Regarding Latrines in a Taenia solium Endemic Rural Area in Eastern Zambia
Source: PLoS Negl Trop Dis. 2015 Mar 4;9(3):e0003570. doi: 10.1371/journal.pntd.0003570 (PMC4352092; doi:10.1371/journal.pntd.0003570)
Supplement: S1 Dataset — (ZIP) [file pntd.0003570.s001.zip › FGD transcriptions_Zambia-2010/Nyazowani_Mr Sakala/Children_Nyazowani_02-08-10.doc]

**Title : Focus Group CHILDREN**

**Date :** 02/08/10

**Site :** Kakiwa Rural Health Center

**Village :** NYAZOWANI	**Location :** Petauke district, Eastern Province, Zambia

**Duration (total time) :**	43 min	**- Start :**	10:42 am	**- End :** 11:25 am

**Participants:** 8 children

**Informed consent:** signed and available

**Moderator :** Mr. Emmanuel Mwanza	**Recorder :** Dr Andrew Phiri

**Transcription/translation :** Mr Boniface Sakala

**Abreviations : M : moderator, R : recorder, I: intervener, C : children, W : women, M : men**

**Disposition (from the left of the Moderator):**

**M**

C1

C2

C3

C4

C5

C6

C7

C8

**R**

Gender distribution:

**C4-C6-C7-C8**: females

C1-C2-C3-C5: males

Age composition:

C1-C2-C3: 18 yrs

C4-C5: 17 yrs

C7: the youngest

Typology:

C1: dominant

C7-C8: quiet

C4: expert

**Comments about participants :**

C4 and C5 went more in the circle (head and chest), acting more actively in the discussion.

C8 doesn’t even laugh when every other child do. She seems to be somewhere else.

**Group dynamics :**

Nice focus group on what has been discussed

**Text:**

Introductions of the mission and informed consent done

*Introduction*:

M: I would like to welcome you all to Kakwiya, and would like to ask you to be very free and contribute very freely to this discussion. The main reason why we have met here is to discuss how we keep pigs in our village, I would be very happy if all of you are free to discuss on how we rear pigs. But before we proceed I would like that we all know each other, I am Emanuel Mwanza I am one of the helpers who work here at the clinic.

All the children introduce themselves.

*Discussion*:

M: Thank you very much, as I said from the start let us all be very free to participate. Why do we keep pigs, what is the good part of rearing pigs?

C4: The goodness of rearing pigs is that when we sale a pig, especially us who are school going we are helped with money for fees.

M: Anyone else?

C7: If there was a funeral at my home they there slaughter a pig so that people can eat.

M: Anyone else?

C6: Sometimes when there is a wedding ceremony, a pig is slaughtered to feed the guests.

M: Who can add anymore to what has been said?

C1: The goodness of keeping pigs is that during the Christmas celebration a pig is slaughtered so that we can celebrate Christmas well.

M: What is bad about a pig?

C4: When pigs enter the field they will damage the crop very badly especially maize, sweet potatoes and other crops.

C3: Then the other bad thing about pigs is that, they sometimes go to the bush eat feces and come and eat in kitchen utensils which are left an attended.

M: Anymore?

C5: They damage the fields.

M: Anymore contributions?

C7: It sometimes goes to the graveyard where they burry premature babies, dig them up and eat.

M: Do pigs eat humans?

All: Yes, especially premature babies.

C1: Yes, especially premature babies are eaten on many occasions.

C2: Yes, because they do not burry them very deep.

M: What is bad about a pig eating feces?

C1: The problems of pigs eating feces is that after eating the feces it would come home and eat from kitchen utensils and because in many homes they send small children to wash kitchen utensils. The children would not clean the plates thoroughly well. We shall then also eat the feces from the bush.

M: Other bad things that have not been covered?

C6: Sometimes it would come and spill water in the home.

C2: A pig sometimes would be slaughtered soon after eating feces. We would just wash the mouth not knowing that some feces are already in the mouth and stomach. Then when we roast the meat we include the intestines which would not have been washed well and there sale our customers contaminated meat which would make the sick, because it has diseases.

M: How do you keep your pigs, do you just let them roam about or they are kept enclosed in a kraal day and night?

C1: They stay in the kraal.

C4: But some do not have kraals.

M: Those without how are they kept?

C1: Those that are without kraals just roam about scavenging on feces and whatever food they may find. Like at our home I call them to feed them three times in a day and after that they go back and continue with the scavenging. The problem with pigs is that they never stop eating, but in the evening they come back to sleep.

C4: Pigs never stop eating especially on eating feces.

*Laughter*

M: Why do those people who do not have kraals not build them?

C2: They do not care for their pigs.

C4: Some are too busy with other chores.

C1: Some keep pigs for sale because they do not eat pork themselves.

C4: Still some do not care as long as they can find their pig and sale.

M: What bad things do you see from these pigs which just roam about?

All: They bring diseases.

C2: They bring diseases to us who eat the pork.

M: How?

C6: Because it just roams about and eats anything it finds that is how it can bring diseases.

M: Would fences be very necessary in the rearing of pigs?

All: Yes.

C1: Fences are very important and necessary in the rearing of pigs, because the fence would prevent them from eating bad food and they would only eat feed that we provide. That way the pigs would not act as a transmitter of disease to us who eat pork.

M: Now if these pigs are just moving a bout, how would you know that this is my pig?

C4: You give it a mark; some times you cut the ear lobe in a certain way.

C1: Some people cut a bit of its tail.

C4: Some are born with a certain colour in a certain part of their body.

C1: These birth colors would be on the legs.

C6: Some would be born with a white forehead.

M: What challenges do you meet in the rearing of pigs?

C3: We meet a lot of problems in the rearing of pigs, like they would go and eat into someone’s field and they would take you to the village council and you would be charged to pay for the damage caused.

C4: Sometimes you would find that some people would axe it if they found it in their field.

M: Are there any family members not from your own home who would help you in the rearing of pigs?

C4: Yes they do but in most cases it is you who takes the lead.

C1: Yes because sometimes you would find that you do not have maize bran to feed the pigs and a relative would pound her maize and if the did not have pigs they would bring the bran to you. Here in the village we eat white maize meal which is good and nice.

*Laughter*

M: So does it mean our relatives help in the rearing of pigs?

All: Yes.

C2: At some point you would give her a pig in appreciation of the bran she gives you or give her a piece of meat when you slaughter the pig.

C1: You give them a pig so that they can also rear a pig to help them if they encountered any problems.

C4: Since problems which affect us are numerous.

M: Now supposing you had ten pigs which are just roaming about how would you know that you pig is sick?

C4: The pig becomes less active and the mane hair would be standing.

C5: Sometimes the pig looses appetite.

C1: Sometimes you would notice a lot of fluid coming out of the nose. Like us who like meat we just notice it and immediately we kill it.

*Laughter*

M: You do not want it die if it fell sick?

C1: Yes because if it died on its own, we may not eat the meat but if I kill it before it dies al the disease in the blood would come out.

M: So you mean if you cut the throat the diseases would come out?

C4: Some of the diseases come out.

C1: Some of the diseases come out some remain and we eat together with the pork.

*Laughter*

M: Who feeds the pigs at home?

C4: In most cases it is us the children who are responsible of feeding the pigs.

C1: The job to feed pigs is for the children of the house.

M: How do you the children share the duties on the feeding of pigs?

C4: Sometimes we draw something like a roster where one feeds them at a particular specified time.

M: How many times do you feed the pigs in a day?

All: Three times.

C3: Some feed them once.

C1: There are those who are lazy in feeding pigs they feed them only once, those are the ones that eat a lot of feces.

C4: Some because of having fed on feces the whole day they do not even eat the maize bran in the evening.

M: Do all the villagers feed the pigs three times a day?

All: Not all villagers some cannot manage.

C6: Some of us manage but some cannot manage.

M: Now those who do not manage what do they think the pigs eat?

C1: they eat feces.

C2: If you feed it once then what can a pig eat, it will eat feces.

C1: And since most people do not have toilets and go to the bush and the pigs would feast on the feces.

C4: Because they are hungry they would follow any one going to the bush and eat feces.

M: When is pork eaten in large quantities?

All: During Christmas, funerals and marriage ceremony.

M: Once a pig is slaughtered, what do you look for to check whether the meat is good?

C4: We check for the fat.

C1: We check for the fat and the general appearance of the meat.

C4: You actually forget that the pig was eating feces.

C1: They way it is baked you even forget that you are still on earth you think you are in heaven with the angels. You forget that it was eating feces.

*Laughter*

M: Before the pig is slaughtered is it examined by the inspectors?

All: It is never examined.

M: Why are they not examined?

C1: The problem is that we are usually not sure whether the meat has got diseases or not.

C4: We are not sure if the meat is fit for human consumption or not.

M: Now if a rule was passed that all pigs should be inspected before slaughter. Would you be happy?

C1: Yes we would be happy because that would reduce the disease burden very much.

M: How do you like your pork prepared, boiled or roasted?

All: Boiled pork is very tasty.

C1, C2, C4: Roasted meat does not cook thoroughly; you find that when you are eating blood would be dripping from it. You will find that as long as meat attains a brown colour outside they will remove it from the pan, when in fact the inside is not even ready.

C1, C3, C6: While boiled meat cooks thoroughly, you find that the way it is outside that is how the inside has cooked. The procedure of cooking boiled meat is very effective because they would boil it in a lot of water and fry it and add spices to the meat which improves the taste of the meat, you even forget that there is death.

M: Why is that most people like roasted pork especially meat around the neck?

C4: Some of the people who like roasted meat they would buy that meat and go and re do the roasting again.

C1: That which they fry some of them just eat without realizing what they are doing.

M: Have you ever heard of nsembe or masese in pork?

All: Yes we have heard about them.

M: Do you know what masese is?

C1: Masese is the solid part of a local beer, which is fed to the pigs.

M: Have you seen this picture?

All: Yes.

M: How does the meat look?

All: It has nsembe.

M: Now if someone comes around carrying a basket with pork do you take care to check if it has nsembe?

All: We are very careful especially to check for nsembe.

C1, C3&C4: We check for nsembe and if the meat has nsembe then we realize that the meat has a disease then we would not buy that meat.

C1: Because it has nsembe then I know the meat has a disease.

M: What if now we wanted to roast the meat even when we know it has nsembe is the meat thrown away?

C1, C2, C3, C4: They would never throw it away but what you would notice is that the meat would be on sale for a long time than if it had no nsembe.

C1: And in most cases you would find that the people who buy that meat are those who are drunk, because they do not know what they are buying.

C6: Indeed they are the ones who buy that meat.

*Laughter*

M: To do what to the mouth?

C4 & C6: To stimulate their taste buds.

*Laughter*

M: They do not care that this meat has nsembe?

C2; No, for them as long as this is meat they just eat.

C4: they just buy and eat the meat without caring what the meat has.

C1: Drunkards have one thing in their mind the see meat, and they have the money to buy the meat so they just buy and eat. That is why most people think drunkards usually loose some part of their brain when they are drunk.

M: Like you would you eat or throw away nsembe meat?

C4: If I am there and see that the meat has nsembe, I would not eat that meat.

C2: But when it is roasted sometimes it is very difficult to see whether it had nsembe or not.

I (Dr. Mwape): Do you eat meat from roasting stands?

C2: Yes.

I (Dr. Mwape): so you eat that meat?

C2: You would buy and eat it there and then.

*Laughter*

C1: Some also buy meat from a roasting stand and take it home to boil it, so we really differ in the way we eat the meat.

M: When you see nsembe what danger do they pose to human life?

C6: They are dangerous to human life because the have a disease.

M: They have a disease?

All: Yes.

M: Anyone else?

C1: I think it is just a disease.

C4: Many times it is the disease that is more dangerous in nsembe infested pork.

M: Is there prevention for *nsembe*?

C1: Yes there would be a prevention, and in my opinion the prevention is to have you pigs in a fence so that you know what they eat, unlike if you let them roam about because you may not know that you neigbour would have thrown Masese and you pig eat them. As result develop the masese condition.

C4: Pigs should be kept in a fence to control what they eat.

M: My sister what do you see as bad when people go to help themselves in the bush (*C7*)?

C4: Sometimes it is just because you are pressed that is why we use the bush, sometimes it at a point where there is no toilet where you can help yourself.

M: What is bad about going in the bush to help yourself?

C1: It brings diseases, you would go into the bush and the flies would come and sit on the feces, once they leave the pile of feces they would go to the neighbor sit on the food they are about to eat and transmit a disease to them.

M: Suppose you had this very well fed castrate, you slaughter. it and found that it had *nsembe* do you throw away such meat?

All: It is never thrown away

C1: You sale it but you are not comfortable selling it.

C6: Sometimes you just take it to the roasting stand so that drunkards can buy it.

*Laughter*

M: So drunkards do not care?

All: Yes they eat any form of meat.

C1: They eat to excite the taste buds.

*Laughter*

M: What if medicine was found that would prevent nsembe, would you be happy?

All: We would be very happy.

M: Why?

C3: We are being protected from diseases.

C1: It would be very nice because you know even when you are selling the meat you must be free that you are selling good meat and you even when you are eating you know that you are eating good meat. Than the current where we are not sure even ourselves.

C2: There was a time when there was a rumour that they would be inspecting the meat, we were very happy but we have seen that in the villages the meat is not inspected.

C1: We were all very happy.

M: Have you ever seen worms, in some child’s feces how do they look?

All: Yes.

C6: They look like earth worms.

C4: Many times some children just vomit them.

C1: Sometimes they appear like intestines but if you observe them, you see them make some movements then you realize that these are worms.

M: How do worms infect a person?

C1: Because of eating anything, like we have said if you eat a pig with nsembe you surely would suffer from worms.

C6: Yes if you eat meat with nsembe you will definitely suffer from worms.

M: Have you ever seen what is on these pictures?

M passes pictures of human feces laden with tapeworm proglottids

C4: I have never seen these since I was born.

M: You have never seen these?

All: No.

M: They look like a wood borer or the shaded skin of a snake. You have never seen anything like this?

All: No.

R: You are not participating, have you ever seen these before?

*Silence*

M: Is there medicine to treat worms?

All: Yes.

M: What is bad about worms being in a person stomach?

C1: They share the food that a person eats so the person would not get a full benefit of the food that he/she has eaten, because part of eat would go to feeding the worms.

C4: Because the person is sharing the food with them the person does not gain weight.

C1: Then the people think that you are suffering from lets go (AIDS).

*Laughter*

C4: When in fact it is worms.

M: How would you know that this person has worms?

C6: The person eats very frequently, it seems they feel hungry soon after eating food.

C1: The stomach of such a person rumbles very much.

M: What makes the stomach rumble?

C4: The worms.

C1: We think it is the worms.

M: What other signs would show us that this person has worms?

C4: The person is frequently sick.

M: What would happen with this person?

C1, C4: The person would die.

M: would that be the most dangerous thing about worms?

C1: Yes.

M: Have you ever heard of people who suffer from fits?

All: Yes.

C1: Here we call it the bicycle.

*Laughter*

M: In your village are there any people who suffer from fits or the bicycle as you call it?

All: They are there.

M: How do fits infect the person?

C1: Some people are bewitched some it is just a normal disease.

C3: Some people it is because of negligence, you would find that a child has been sick for a long time then they do not take it to the hospital and because of other complications the child would start fitting.

C4: Some other people even when they have been given the medicine and the child is feeling better then when he starts fitting they would say it is because it is not yet full moon.

M: Now when there is a full moon what happens?

C4, C6: The child stops fitting.

C1: They stop fitting for a period only, it does not completely finish.

C4: Some it is because blood has finished in the body of the child then it would start fitting.

C1: Then they come to ask Mr. Mvula to give the child treatment.

*Laughter*

M: Is treatment available at the clinic?

C1, C4: There is treatment, they give the child injections, and then the child would stop fitting.

M: Is there a disease which you think is bigger than fits in the villages?

C3: HIV and AIDS.

C4: There are two diseases which are big AIDS and fits.

M: Are fits a very bad disease?

C3: Yes, but AIDS is bigger.

*Laughter*

M: Which disease do you think is smaller than the two?

C1: Chest pains, sneezing.

C4: Stomach pains can stop after some time.

C2: Malaria because you can be treated if you go to the clinic.

C3: Malaria if you go to the hospital but if you just stay at home you would be in grave danger.

*Laughter*

M: I know that at home we have toilets.

All: Yes.

M: Do we all have toilets?

All: Some have got and some do not have.

R: Those with toilets can they lift up their hands?

All: Lift up their hands.

R: Do you have a toilet C1?

C1: Yes we have a toilet, I said some have and some do not have.

M: What is the use of a toilet to a person?

C3, C4, C6: It helps to prevent a lot of diseases, because after eating the food we need to get rid of the waste material so if we have a toilet we do al that in the toilet.

C6: We go and help ourselves in the toilet.

C4: We all go to the toilet that is referred to as the big house.

*Laughter*

M: So those without toilets what do they use?

C1: They use the bush; they are the ones who bring diseases in the villages.

M: Can your neighbours use your toilet?

C4: They do.

C6: Now some do not want they still go to the bush.

C2: Some are used to going in the bush so even if there is a toilet in the neighborhood they go to the bush.

*Laughter*

I (Dr. Mwape): If they go to the bush then the pigs would not have food. Is that not so?

C4: The pig would be following him immediately he shits the pig would eat the feces.

C2: The pig would be waiting for him to shit.

C1: If it is a small child shitting the pig would just lift the child off with it’s snout to start eating the feces.

*Laughter*

M: Do we use the toilet in the manner they are supposed to be used?

All: Yes.

M: How do we use them?

C4: The use of a toilet is after we have eaten and when we feel like going to the toilet then we go to the toilet and leave our waste in there.

C1: If I use the toilet and once I have finished I must clean the toilet and cover the hole with a lid.

C2: the toilet must be kept clean all the time.

M: How does the lid look like?

C1: It may be made of wood or cement.

C2: It must have a handle.

M: Why do you keep the toilet closed?

C4: To prevent flies.

C6: To prevent flies from coming in and then coming out to contaminate our food.

M: We have now come to the hygiene of vegetables, would they have a disease if we did not wash them before cooking?

All: Yes they would have diseases.

M: How could that be possible, say like they are in the field?

C4: There are some fields which are okay but some fields have snails so the snails would touch the vegetables and if we did not wash the vegetables we would surely be eating some disease.

C6: Usually at the field there are no toilets, so the flies would go and sit on the feces and then sit on the vegetables, if we did not wash the vegetables then we would be eating diseases.

M: All those fields which toilets do they use when they are at the field?

All: They go to the bush.

M: So vegetables from the fields would they be alright.

C2: No.

M: What is good about washing vegetables?

C1: To remove disease that was caused as a result of flies sitting on the vegetables.

M: They say that pumpkin leaves harden when they are washed, what do you do in that situation?

C6: You first remove the fibers from the leaves and then wash them, if you follow that procedure then it does not harden.

M: What do you think should be done in vegetable hygiene?

All: The vegetables must be washed at all times.

M: When do we wash our hands?

C4: When eating nshima, when coming from the toilet.

C1, C3: when you touch anything dirty you must wash your hands.

M: how do you touch those dirty things which would make you wash your hands?

C1: Supposing my mother left me with a small child if it shits I would need to clean it, after cleaning the child I must wash my hands.

C4: If I come from the toilet I must wash my hands.

C1: In the toilet while cleaning my self the tissue would puncture and some of my fingers touch feces, I must wash my hands straight from the toilet.

*Laughter*

M: Do you wash your hands when you come from the toilet here in the village *(C8)*.

C8: Yes.

C3: Some do not do it.

C1: It is worse among drunkards because they would just come and start eating even without washing their hands and then diseases strike.

C6: They do not even wait for you to give them water to wash their hands.

M: My sister *(C7)* what is good about washing you hands?

C7: We wash our hands when we come from the toilet because we want to prevent diseases.

M: Do the children wash their hands when they come from the toilet?

All: No, because most of them do not appreciate why they are washing their hands.

I (Dr. Mwape): Do you wash your hands?

C2: Yes, because we know the consequences of not washing our hands when we come from the toilet.

C1: If I come from the toilet I must wash my hands with a piece of soap and wipe my hands with a face towel, if I should want to eat some nshima I must wash my hands with soap and start eating.

I (Dr. Mwape): What if you come from the bush?

All: You must wash your hands.

M: Now for example if we are eating fresh maize at the field do we wash our hands?

C4: If you go in the field to collect the maize and start roasting it on a fire, we can wash our hands but sometimes we forget.

C1: Most times we do not wash our hands when we are at the field.

C4: It becomes too urgent sometimes.

C1: Especially if you are hungry you just start eating.

*Laughter*

M: what benefits accrue to us in personal hygiene (*C8*)?

All: You prevent diseases.

C6: You would find that you have some rash which comes out if you do not wash.

C1: The poles on you skin are covered with dirt if you do not wash, then you would fall sick.

C4: You must wash you close.

C1: Yes you must wash you clothes so that they are clean.

M: Would we be happy if our meat in the villages was inspected?

All: Yes, we would be very happy.

C1: Because we would know that our pig has no diseases, even if I ate the meat there is no risk of a disease.

C6: That is when you enjoy the meat because you know it is okay.

M: Would we be happy that the pigs are kept in a fence?

All: Yes, we would be very happy.

M: What is good about a fence?

C1: The pigs do not eat feces they eat the food that we give them

C4: Also to prevent swine fever, if pigs are in a fence most times they do die from swine fever.

C1: That animal should have a place where it feeds from, not where I use the same utensil as a pig.

M: What problems would you face if you do not have a fence?

C4: The pig would bring you a lot of cases.

C1: And also it brings diseases any time.

M: What problems would we have if we did not have toilet at our homes?

C4, C1: Diseases would be abounding.

C1: You would fall sick and die.

M: Would we all manage to have toilets?

C1: There are some who are lazy who do not want to have a toilet, some are use to going in the bush. Those who understand and dig a toilet are those who appreciate what others are telling them.

M: Is washing hands a good life?

All: That is very good life style.

M: Why?

C6: It prevents diseases.

M: If they brought medicine to protect pigs from worms and *nsembe*, would we be happy if this came to our area?

All: We would be very happy.

C1: Very happy.

C4: If we the people get vaccinations to prevent diseases we would also be happy that the animals are given vaccinations to prevent diseases.

M: What is important for a pig to be vaccinated?

C1: Nsembe and worms can be prevented from infecting the pig so that it is healthy.

M: Do people throw away the intestines after slaughtering the pig, knowing it was eating feces?

C1: No, we eat the intestines.

*Laughter*

M: If you slaughtered a pig and it was examined and found that it has a disease can it be thrown away?

C1: Yes, it can be thrown away.

C2, C3, C4: Agree.

M: If the government bought a rule that all pigs should be kept in a fence, what problems would be faced?

All: There would be no problem.

C6: that would also help protect our lives.

C1: A lot of things would be prevented: cases, diseases and eating from utensils all these things would stop.

M: We have come to the end of our discussion, we discussed the good and bad part of pigs, how we rear them in the villages, we have also discussed about inspecting the meat which is currently not being inspected, we discussed about worms, nsembe, washing of hands when you come from the toilet, we learnt that if we wash our hands we are preventing disease, we also learnt that pigs should be vaccinated, in the end we learnt that it would be good if the government brought a rule that all pigs should be kept in a fence. If you have any questions you can ask me.

I (Dr. Mwape): What is *Mpanda*?

M: Fence.

I (Dr. Mwape): Why then do you not keep the pigs in a fence if it is good?

C3: Not appreciating the idea.

C4: Lack of value and understanding.

C1: Some of the just turn into politics and lack of understanding. Some are just too difficult even on very obvious issues. So in our area what people have resorted to is just make snares and if it is caught in the snare you just burry it because if you tell them to make a fence and enclose their pigs they would tell you that their pigs do not go any where they just sleep at home.

I (Dr. Mwape): If your father told you to bring an axe what do you think he is going to do?

*Silence*

M: The life of going into the bush to help oneself is that alright.

C6: That is not alright.

C1: We think that he is going to help himself, but you would find that the pig would come back and eat from the kitchen utensils or since when we eat even us humans we would want to drink water the pig also would come direct from there and drink water from the same items we use for storing drinking water that is not good.

**The end**
