# Supplementary material for: Why Latrines Are Not Used: Communities’ Perceptions and Practices Regarding Latrines in a Taenia solium Endemic Rural Area in Eastern Zambia
Source: PLoS Negl Trop Dis. 2015 Mar 4;9(3):e0003570. doi: 10.1371/journal.pntd.0003570 (PMC4352092; doi:10.1371/journal.pntd.0003570)
Supplement: S1 Dataset — (ZIP) [file pntd.0003570.s001.zip › FGD transcriptions_Zambia-2010/Nyazowani_Mr Sakala/NYAZOWANI VILLAGE-MEN-Evans edited.doc]

This one was a little boring maybe since it was Benjamin moderating. The moderator seemed to have talked more that the discussants.

**NYAZOWANI VILLAGE – MEN**

M: Thank you very much for coming to this discussion, there are no teachers between you and me. We are here to discuss how we rear pigs in our villages and communities, we must all be free to contribute to the discussion and be free to comment on any question that will be asked and say what we do or what happens at our homes, we must be as honest as possible but first let’s get to know one another.

*All participants introduce themselves.*

M: We’ll begin by looking at the benefits of pigs, what are the benefits rearing pigs?

C4: The benefit is that we eat the meat.

M: Anyone else?

C8: The other benefits of rearing pigs are that sometimes you would have a funeral and you would slaughter a pig to feed the mourners, then you would slaughter a pig at a wedding ceremony and use the meat to feed the guests and sometimes you would slaughter a pig sale the meat and pay for your children school fees.

C6: You would also slaughter a pig and distribute the meat to people so that they can help you weed your field.

M: Anyone else?

C3: Sometimes you would receive a funeral message from Lusaka, you would sale pig and use the money as transport to go to Lusaka.

M: Anyone else?

C6: If you have children in secondary school you would sale or slaughter a pig to help you send them to school.

C2: If the pig multiply to a very good number, you slaughter one and eat with your family at home.

M: C1?

C1: We rear pigs so that we can eat at home.

M: What are the disadvantages of rearing pigs?

C8: The disadvantages of rearing pigs are that a pig eats anything it finds, therefore there are those who do not have toilets at their homes who go into the bush to help themselves and the pigs would follow them and eat their faeces. The same pig would come home we catch it and slaughter it, and then we would feed on the meat.

C2: Sometimes you would go into the bush shit and the pig would come and feed from the utensils at home.

M: Anyone else, C1?

C1: The pig would go in the bush and feed on rubbish and come to feed from your home utensils.

M: Anyone else?

C4: You would find that a woman would have a miscarriage, and when the women go to bury the still born baby, because in our tradition the still born babies are not buried dip a pig would go and dig up the baby and eat.

M: Anyone else?

C7: There are times when the person who goes to the bush to help himself is sick say from any diarrhoea disease, the pig eats his faeces and you in turn eat pork from such a pig or it feed from the utensils then it would have transmitted the disease to you.

M: How do we rear our pigs, do we let them to roam around or build kraals for them?

C6: We let them roam about.

M: You let them roam about?

C6: Yes.

M: Anyone else?

C8: Many times pigs would just roam about even when there is a kraal they come and go.

M: What is the benefit of pigs roaming about?

C8: The advantage is that sometimes because of the difficulties we face to find feed for them, when the roam about the can scavenge on any food they find, even though some of the food would not be good.

*Laughter*

M: Anyone else?

*Silence*

M: What are the benefits of letting pigs roam about?

C5: Like my brother said earlier we let them out so that they can scavenge some food then we supplement them in the evening with a bit of feed.

C8: When we let the pigs roam about they feed on many types of rubbish, and our main purpose of rearing pigs is to use them as relish when we slaughter them. The problem therefore of letting them roam about is that they would eat some of the rubbish that are diseased and they would pass on these diseases to us when we slaughter and eat the meat.

M: Anyone else?

C3: Sometimes it would eat from someone’s pot and the person would hit it and break its leg.

C4: There are some people whose fields are near the village, the pigs would go and destroy their crops and then we would have cases.

M: Anyone else?

C6: Sometimes a pig would go into the bush eat faeces and find children eating it would come and feed from the same plates where the children are eating from and they would continue eating.

M: Can we not do the same also?

*Laughter*

C6: No.

M: The disadvantages of letting pigs roam about?

C2: Sometimes the owner of garden would put a fence around their garden to protect the crops, but the pig is a difficult animal which would make away even through thorns. Some owners have resorted to putting snares around their gardens, a pig would be caught in those snares and die.

M: Anyone else?

C5: The pigs can bring a lot of problems if they are left roaming about, they can also bring diseases.

M: How do we keep pigs and what problems do you face?

C3: The other problems we find in the rearing of pigs are that, when the pigs have multiplied and have reached a market age and you even sell some and the swine fever strikes they would all die. In some cases you find that you would have only one or two pigs remaining. This in my opinion is the greatest challenge for most people who rear pigs.

M: Anymore in addition to what C3 has said?

C4: If you rear the pigs and they multiply, you would have problems in finding feed for the pigs and that may be one of the reasons why people let them out.

M: Are there any people who help us in the problem we face in the rearing of pigs?

C6: No body.

M: For example during the swine fever attack who helps you?

All: No one.

M: How do you know that your pig is sick?

C3: We sometimes know from the young one when they become inactive and do not feed well, after a few days the piglet would die and then another one would die then we realize that our heard has been violated by a disease. Then in a few days the mother would become inactive and not feed well, then it would stand still with its snout into the ground.

M: Anyone else?

C6: The tail also is not very active.

C4: The ears would also not be standing straight instead they would hang down.

M: Others, how would you know that the pig is sick?

C1: It becomes inactive and does not feed well and the snout is down on the floor.

M: What job do we men perform in the rearing of pigs?

C3: The pigs help us solve our financial problems in a home.

M: What role do we play in the rearing of pigs?

*Silence*

Dr. Phiri: What role do we play in the rearing of pigs are we involved in feeding them or are we the ones who give them water?

C8: The major role we play is that of building a kraal, the feeding and other roles are usually performed by women and other members of the family.

M: At the beginning you said that you let the pigs roam about and you do not keep them in the kraals, now what kraals are you talking about?

C6: Yes, during the rainy season we build kraals for them so that they can be kept inside, so that they do not stray into other people’s fields and that is when we feed them.

M: What other roles do we play apart from building kraals?

C3: The major role men play is to build the kraal, all other roles of feeding the pigs, putting them in pen for the night and watering them are played by the children.

M: All the time?

C3: Yes

M: Is that the case in all homes?

C3: Yes there are only a few instances when men can take part in feeding the pigs, for example when the children are busy then the father can feed them.

M: then it is not always that the children only can feed the pigs you also feed them is that not so?

All: Yes.

M: What other roles do we take part in?

*Silence*

M: How do we feed our pigs and what do we feed them on?

C4: Maize bran.

M: How many times in a day?

C3: Three times a day.

M: Do you just give them bran only?

C4: Maize bran and like this season we give them pumpkins.

C3: Also some leftover nshima.

M: How many times do you give them maize bran in a day?

C6: Three times a day.

M: Do they fend for themselves at times?

C4: When they are outside, yes they look for some food.

C8: sometimes they fend for themselves.

M: Are there any other family members who help us in the rearing of pigs?

*Silence*

M: Are there any other people who help us in the rearing of pigs those we do not stay with?

All: None.

M: Who slaughters a pig?

C6: The men.

M: The men?

All: Yes.

M: Is that all?

All: Yes, a woman cannot slaughter a pig.

C7: The women only bring water to clean the meat.

M: But who keeps the pigs?

C3: It is the women.

All: The women.

M: Because they stay at home?

C3: We also stay at home sometimes.

C8: Keeping them we help each other.

M: Who makes a decision to slaughter the pig?

All: It is the men.

M: At all times?

C3 & C6: Not all the times.

M: What do others say?

*Silence*

M: C4, who makes a decision to slaughter a pig in your household?

C4: There are times when you have your own pigs and your wife has her own pigs in the same kraal, she would ask the husband to slaughter one of her pigs.

M: So the women can also make a decision?

All: Yes.

M: Can the children also make a decision?

C4: Yes, a child can if he/she has her own pig.

*Phone rings*

M: You said you all feed the pigs. What is good about feeding pigs?

*Phone rings*

C8: The benefit of finding pigs is that they grow up healthy.

M: Others?

C4: They gain weight.

M: Others?

C3: If the pigs are fed well, they always remain around the home they do not wonder about eating faeces and other bad food.

M: What is the benefit of pigs eating faeces?

C4: There is nothing good about pigs eating faeces.

C3: There is absolutely no benefit.

M: Why?

C6: Because in some households there are no toilets and sometimes there is only one toilet for so many people, when the pigs go to the bush to eat faeces they would bring diseases.

M: Anyone else?

C8: It would eat the faeces to keep alive.

M: Anything else, what is bad about a pig eating faeces?

C4: For example if a pig ate faeces of a person who is suffering from diarrhea and comes home then feeds in any utensils which we use at home, they can transmit that disease to the households.

M: Anything else C2?

C2: There are some pigs which are very term and if they go to the bush and eat faeces, then it comes home and starts to play with the children and the children in the process may touch its mouth and get a disease or the faeces into their mouth.

C3: There are times when a pig has eaten faeces and comes home with faeces all over the mouth and it would be stinking badly that even you the owner does not feel comfortable being near it.

M: How do we feel when we see a pig eating faeces?

All: We do not feel well.

*Laughter*

M: Do we eat pork?

All: Yes we eat.

M: Where does the pork come from?

C8: The meat comes from the villages.

M: Do we buy or we are just given this meat.

C8: Sometimes we buy and sometimes we slaughter our own pigs.

M: When do you usually eat pork?

C8: We mostly eat pork during weddings, church gatherings and at funerals.

M: How about when you are playing football?

C8: That is also another time when we eat a lot of pork.

M: When you want to buy pork what do you check for?

C3 & C8: Fat.

M: Others?

C4: To check if it has no nsembe.

M: Others?

C1: I check for the appearance of the flesh.

M: Others?

C4: We check for the appearance of the meat if the pig died on its own the meat would appear very red.

M: Is the meat examined after slaughtering?

All: No.

M: Why?

C4: Here there is no inspector.

M: There is no one; do you know that Mr. Mvula is a meat inspector?

All: We do not know.

M: Would you be happy if your pork was examined?

C6: we would be very happy considering that it can carry diseases.

M: Others?

C4: Yes we would like it but others would not throw away their meat even when they are told that the pig has a disease.

*Laughter*

C8: To be honest we all know that there is a meat inspector but the problem is that if the inspector recommended that we throw away the meat if it was found not fit for human consumption, we would loose, we therefore ignore the inspector and do it our way.

*Laughter*

M: So you are aware of the presence of the inspector?

All: Yes

M: How do you like you meat prepared?

C4: We like roasted meat.

M: Why?

C4: It has a nice aroma.

M: Others?

C6: I like it boiled>

M: Why?

C6: Because it is prepared with some soup.

C5: In my opinion all the preparations are good because we are interested in the pork, whether it is cooked, dried or roasted.

M: How is the meat prepared before eating?

C4: We start by killing it, removing the hair and cutting the meat in small parts.

C3: He is talking of how it is cooked.

M: Well we are talking from the time you slaughter up to the time it is cooked in the pot.

C3: When we decide to slaughter a pig we cut it on the neck to bleed it, then some use hot water to remove the hair and some use fire by lighting dry grass then we bring some water to wash the skin after that the pig is slit open in the middle.

M: So we have heard that pigs are prepared in different ways, why do we differ in the method used?

C3: Some people do not want their meat to have those hairs on it, so when you use hot water the hot water removes both the thin skin on the pig and the hair completely.

M: Any other reasons?

C8: The pig is usually found in mud that is why some people prefer to use boiling water so that all the dirt can be washed during this process.

M: Anyone else?

C6: Others use the fire to remove the hair because when it is finally done the meat appears brown.

M: Anyone else?

C4: Others also after they finish removing the top skin they put more fire to brown the skin.

M: Do you know masese?

All: Yes.

M: Have we ever seen Masese in pork?

All: Yes.

M: What do we think when we see masese?

C4: We think that the pork has a disease.

M: Anyone else?

C6: We think of throwing away the meat.

M: Do you throw it away?

C3 & C6: We do not throw it away.

M: Do you eat the meat even when we know that it has masese?

All: Yes.

M: Why?

C3: We just eat because we are used and we eat because it is meat.

M: Anyone else?

C8: What we do not want is to lose out.

M: Loss to whom?

C8: Loss to the person who was rearing the pig.

M: Does it mean when you eat you make a profit?

C8: Yes when I eat the meat I benefit.

M: So we are saying that even when we see that the meat has masese we just eat, in short we would say we eat that meat with masese purposely?

C4: Yes.

M: Have we ever seen someone suffering from worms? (Passes pictures with proglottids in a humans faeces.)

All: yes.

M: Have we ever seen worms in a person’s faeces?

All: Yes.

M: When we see worms in someone’s faeces what do we think or do we know them?

*Silence*

M: Would we know that these are worms?

C3: It is difficult but when the worms start coming out then we would know.

M: My question was whether you have seen them and if you have where?

C5: We see them when we ourselves pass them out, when we are infected with them.

M: Anyone else?

C6: Sometimes we see the worms in our children’s faeces.

M: Others?

C4: Sometimes they are found in pigs, I have seen them after slaughtering a pig.

M: Where?

C4: In the flesh itself, I have found worms.

M: You, you are have a pork roasting stand so you see these things.

M: How would you know that this person or child has worms?

C6: A person who has worms usually has a swollen looking stomach.

M: Anyone else?

C7: The stomach of a child with worms always appears full.

M: How about an old person?

C7: It appears the same way.

M: Anyone else?

C3: Sometimes even yourself you would find the stomach rumbles all the time.

M: Anyone else?

C5: You find that when you are eating food you do not get satisfied, then we would suspect that we have worms, because we are sharing the food with the worms also.

M: Anyone else?

*Silence*

M: Where do these worms come from?

C6: They come from the stomach.

M: They come from the stomach and come back to the stomach?

C6: They come from the pigs and go to man.

C3: They come from the different foods which the pigs eat.

M: Like which foods?

C5: like from the pig.

C3: Like if you consistently eat rape you would have worms.

M: Rape?

C3: Yes, rape.

M: If you ate it too much?

C3: If you eat it too much it brings worms.

M: Anyone else?

C4: If the relish is not cooked completely they would transmit worms.

M: Anyone else?

*Silence*

M: Can these worms be prevented?

All: Yes, the can be prevented.

M: In which way?

*Silence*

M: C1?

C1: They can be prevented.

M: In which way?

C1: We fear them.

Dr. Phiri: How can they be prevented?

C1: To fear them.

M: give us an example how they can be prevented?

C3: To prevent worms we first must make our food clean.

M: Anyone else?

*Silence*

M: C7 how can we prevent worm infection?

C7: By taking care of our food.

M: Anyone else?

C8: If you knew that we are infected by worms you go to the hospital and they will give you treatment.

M: Others how can we treat worms?

C4: The food must be thoroughly cooked before serving it to people.

M: Anyone else?

*Silence*

M: Are these the only ways?

C6: there are many ways.

M: Then let us discuss the ways.

C3: By taking care of ourselves, we are giving you the same answer because that is the only way we know how.

Dr. Phiri: Have you ever seen worms like those in the picture which has just been circulated to you?

C1: To see them?

Dr. Phiri: Yes in the faeces of someone.

C4: Inside the toilet?

C3: No in the faeces.

C4: Yes they are found especially in the intestines of pigs after slaughtering.

Dr. Phiri: How about in humans?

C6: They are found.

Dr. Phiri: Have you ever seen them?

C8: Well if they found in animals most likely they would be found in humans as well, although we have never seen them in humans.

M: Is there medicine for worms?

All: yes the medicine is there.

M: Where is it found?

All: At the hospital.

M: Are you scared of contracting worms?

*Silence*

M: Are you scared of worm infection?

All: Yes.

C3: Yes because they can kill you.

M: What disease are you very scared of?

*Silence*

C4: The one that is more dangerous than worms?

C4: Yes.

M: The disease that is more dangerous than worms?

C4: Yes, Diarrhea

M: The disease that is more dangerous than worms?

C3: HIV and AIDS.

M: Anyone else?

C8: Cholera.

M: Are there many people who suffer from worms in the village?

All: Yes.

M: Do you think worm infection is a very prevalent in Zambia?

C3: Well this was a very prevalent disease in yester years but not anymore.

M: Do you know that worm infection is a very difficult disease in Zambia?

C8: No we do not.

M: What would happen to a person infected with worms?

C5: If the person who has worms does not get treatment quickly he would die.

M: Anyone else?

C4: It would harden his stomach, would appear like he has eaten when not.

M: He would eat a lot of food?

M: Anyone else?

C3: the person does not gain weight and appears very slim.

M: How about small children?

C4: The child will be very thin and with a bulging stomach.

C3: The child would eat continuously and does not get satisfied.

M: Anyone else?

C4: Does not get fat.

M: How about his life?

C3: The life of such a child is always sickly.

M: Do they grow up?

C3: Yes, but they are usually stunted.

M: Nsembe or masese have we ever seen them?

C4: Yes we have seen them before.

M: How does it look and can we give an example?

C4: The look white, every where you cut you find white stuff in the flesh.

M: Where does masese come from?

C3: those come from pigs feeding on waste material that are thrown away by those who brew Kachasu (Local illicit brew). When pigs feed on these then they develop masese.

M: Anymore thoughts?

C4: Sometimes it is just a disease even if the pigs do not eat those wastes from the brewing of local beer.

M: Anyone else?

*Silence*

M: Can this disease of Masese be prevented?

C7: It can be prevented if they are not given masese.

C3: We cannot prevent the disease because we do not have the medicine to prevent this.

M: Anyone else?

C4: It can be prevented if we had the medicine so that they can grow well.

M: Can this disease be cured?

C8: The disease can be cured if the medicine is found.

M: Are we frightened of masese infection?

C3: Yes we are frightened of masese disease because they damage a person’s body.

M: Are we scared of the masese disease?

All: Yes.

M: Is there any other disease that we know that surpasses masese?

*Silence*

M: There is no example of a disease that we know that surpasses masese?

C6: Yes there is swine fever.

M: Any other disease?

C4: There is disease which starts with a pig losing its hair and then the skin begins to shrink

M: Are there many people who suffer from masese disease in our villages?

C8: We do not know.

M: Is masese a very prevalent disease in Zambia?

C8: We do not know again.

M: You cannot answer for all.

C8: Well I should say I do not know.

M: Anyone else?

C4: It is not very prevalent, because you may find that one out of seven pigs would have masese.

M: I had said from the start that all of us should participate; we are all just educating each other.

All: Yes.
M: We have heard about fits?

All: yes.

M: How do you contract fits?

C6: Fits are contracted in numerous ways.

M: Give an example of these ways?

C6: Some of it comes through bewitching and the other one comes from the eating of pork meat, the masese once eaten causes other diseases in the stomach.

M: Anyone else what causes fits?

C3: Sometimes it is caused by high body temperature, and then you see a person starts to fit.

M: Anyone else?

C4: Some of them it is just a disease that affects them because of the food we eat but some of them are bewitched.

M: Has it got any cure?

C4: Those who suffer from the natural fits can get cured, the medicines are there but for those who are bewitched there is no cure unless the person who bewitched him reverses the curse.

M: If we contract this disease where do we go?

C3: Sometimes blood would finish in the body and therefore a person would start fitting.

M: Anyone else?

*Silence*

Dr. Phiri: If the blood finishes in the body and you start fitting, and if you were given blood would you be cured?

C3: Yes you get well.

Dr. Kabemba: Do you know people who suffer from fits in your village?

C3: Yes they are there.

Dr. Kabemba: How do you know that they suffer from fits?

C3: If a person has fits, he usually is confused.

Dr. Kabemba: Do you know where it comes from?

All: We do not know.

M: But C6 had said it?

M: Say what you said before.

C6: I said sometimes some of it comes from being bewitched and some of it comes from eating pork with nsembe which when it goes into the stomach changes to another disease like worms and then you start fitting.

M: I am happy you used the word sometimes.

M: We had earlier asked if there was medicine for fits or where do we go when we are attacked by fits?

C6: When we suffer from fits sometimes we go to a traditional healer and if it a child with high temperature we rush it to the clinic.

C3: like it has already been said that there are several causes of fits one from being bewitched, in that case we try our traditional medicine and if it from natural cause then we go to the hospital.

Dr. Kabemba: How do you know that this is as a result of being bewitched or it is from natural causes?

C4: We know because sometimes we take the person to the hospital and when the hospital fails to treat it then we bring the person home and try our traditional medicine and if it fails then we would conclude that may be the person was bewitched.

C8 Sometimes it happens that there are two people from the locality suffering from the same disease, they all go to the same hospital and one gets cure and the other continues to suffer from the same disease and eventually dies, we would therefore conclude that the other one was suffering from natural fits while the other had been bewitched.

M: Do you think we can prevent diseases that affect pigs if we do not keep our animals in the kraal?

C6: Yes we can prevent diseases.

M: How?

C6: Yes they can be prevented because pigs now would not follow a person who goes into the bush to shit, they would not be able to eat rubbish that would prevent them from transmitting the diseases.

Dr. Phiri: Would there be a problem if government said all our pigs should be kept in the kraals?

All: There would be no problem.

Dr. Kabemba: If there is no problem why do you not keep them in the kraal now?

C3: Like we said earlier on the problem is how to feed them, this time of the year there is plenty of food and one can buy some feed cheaply, but come October and the months that follow there is a serious food crisis that is why it is difficult to keep them inside.

Dr. Kabemba: So that problem would it would not be there when the government decree?

C4: Yes that problem would continue because in the rain season even us humans do not have enough to eat, in fact we eat the same maize bran to make porriedge to eat ourselves what more pigs.

C5: Now let me say this it is very difficult for us but if government said we should keep them in the kraal some would not obey but there are those who would want to look after their animals in a manner that is befitting they would. You know people say so many things and some are just difficult.

C8: like we have been saying pigs cannot wonder very far if they are given enough feed but because they are not fed enough they would damage the kraal to get out and look for feed, but if pigs were fed well and enough food they would not wonder about.

C3: There was one year when there was a very bad feminine and people used to go to Mozambique to buy food and when this maize was brought here we used to buy one five litre container of maize at eleven thousand kwacha. We would then buy some maize bran from the grinding mill mix it with the five litre of maize and grind to make maize meal; it is therefore very difficult if you take the scenario I have given to start giving feed to the pig.

*Laughter*

C8: I still remember the year it was 1995.

M: What if government says we should use toilets can we manage to do that?

C3: We can manage because toilets are usually sited very near the house.

M: Why?

C4: To prevent pigs from eating faeces when they go roaming about.

M: Do we all have toilets at our homes?

C3: Some of us have and some do not have.

M: Why?

C4: Sometimes there would be a toilet at home but then at times you see your father enter the toilet even when he leaves you cannot enter the toilet immediately after him, so you pretend like you are going somewhere, when in fact you are going to the toilet.

C3: Sometimes you want to go to the toilet then your sister enters the toilet you cannot all enter the toilet so you are forced to go to the bush otherwise you would shit in your pants.

*Laughter*

M: What do others think?

C8: Even if the government passed that rule the problem of going to the bush would not be completely done away with, because let us take for example that I am a newly wed man and have come to stay with my in laws, there would be a toilet of course at that home but it may just be one toilet and for me to pass and go to the toilet it would be very difficult, as a result I may go to the bush and come back with a piece of log and my in laws would think I went to fetch firewood.

*Laughter*

M: So there are people who are scared to use the toilet at home?

*Silence*

M: What makes you not use the same toilet as your in laws?

C3: Respect, because what if I went into the toilet and urinated on the side and my mother in law came in she would find wet urine. She would know that it is my son in law who has urinated on the side.

*Laughter*

M: If your mother in law saw your urine what would be the problem?

C3: It becomes a problem to you in your mind and you feel shy.

C8: It is shameful.

C4: Even if your mother in law has just left the toilet you cannot go in.

C8: Because whatever you would see that would be wrong you would think it is your mother in law who had done it.

*Laughter*

M: So is it wrong to go to the toilet?

C8: No, we are not really saying that going in the toilet is a bad idea, but supposing you go to the toilet and your mother in law found that you had done the toilet on the side, your mother in law would say how can this old man do this. In relation with our culture and life style in the village it does not show respect.

Dr. Phiri: So when you go in the toilet, you must be very careful that they do not find you with any blemish?

C8: Yes that is correct.

M: So regarding the toilet would they find you wanting?

*Silence*

Dr. Phiri: So even when the government says you should have toilets the culture would prevent us using the toilet.

C3: Like we said before it would be very difficult if there were only two toilet and you are at you in laws homestead, because it is rather not our custom to use the same toilet with your in laws.

M: It is very careless for an old person to miss the hole and shit on the side, are you sick or what?

C3: There are times when you are pressed in the process you do it on the side, but because you had not carried anything to clean the toilet with you just walk out.

C4: Supposing you had diarrhea you would mess the sides of the toilets and when your mother in law walks in she would find the toilet in a mess.

*Laughter*

C2: sometimes you would want to go to the toilet and you did not see your mother in law get in so when you want to get in you find your mother in law squatting helping herself. That would not be taken kindly by your in laws here in the village.

C3: Because you would have seen the nakedness of your mother in law you would feel ashamed.

M: You are just two and that is an accident.

All: That is taboo and they can even charge you for seeing you mother in law naked.

Dr. Phiri: Can you be charged money.

C8: You have seen her nakedness you can be charged and need to pay money.

Dr. Kabemba: May be use the toilets with proper doors?

M: If you use a toilet with a proper door would you still be accuse of having seen the nakedness of your in laws?

All: That would solve the problem.

C6: With a door there would be some form of privacy.

C8: But even with a door, even if it is a very small opening eyes are very quick to see, so according to our life style here in the village it is very difficult for you and your in laws to use one toilet.

Dr. Phiri: Would you not meet your mother in law in the bush?

All: We meet, but here is a difference.

Dr. Phiri: What is the difference meeting your mother in law in the bush and the toilet?

C3: You do not go nearby you go to the bush further from the village.

C2: you go like you are going to the field.

Dr. Phiri: You would find your mother in law there.

C8: in the bush it is very easy to ignore a person, but in the toilet immediately you open the door and go back she would know that you have seen her.

*Laughter*

M: If the government said you should boil your pork, would we manage?

All: yes we can manage.

M: Why?

C3: If the meat is boiled thoroughly everything is cooked inside the meat and therefore it is much safer to eat that meat.

M: What if the government says that all pork should be examined before it is eaten; even at a funeral can we manage?

C8: It would work with some people and not with others, because some of them only considers what would happen should the meat be declared not fit for human consumption. I do believe there is a law by government to have all the meat is examined before being eaten but it is not followed.

C3: There are these people who roast meat say if they buy a pig at two hundred thousand and he has already paid for the pig, and then it is examined and recommended for throwing away, it would mean the owner has lost, that is why sometimes they do not have their meat examined.

C4: Sometimes because you have already paid your money you just give may twenty thousand kwacha to the person so that he can declare the meat fit for human consumption.

M: What if the government said all vegetables should be washed before they are cooked, can you manage?

C8: I think that would be managed without any difficult, because there is no loss we know that is hygiene.

M: If government said we should practice good personal hygiene, can we manage?

All: We can manage.

M: Say if they say we should wash our hands when we come from the toilet?

Dr. Kabemba: And when coming from the bush?

*Laughter*

All: It cannot be managed.

C3: You know when we were outside waiting for our friends to come out we all went to the toilet, and then when we came in you gave us some food none of us washed our hands we just ate the food. Usually it is not possible to wash your hands every time.

*Laughter*

M: Is it difficult to wash your hands when you come from the toilet?

C3: It is not difficult but we forget.

C8: Sometimes if you picked a paper and when you want to clean yourself you find that the fingers have got some faeces, at that point you immediately wash your hands when you arrive home. But if you do not find yourself in that problem you forget and do not wash your hands.

Dr. Phiri: What would be the problem?

C4: like we are sited here when you scratch your arm pits you must wash your hands, but that is not practical because from here you would just get a banana and eat.

Dr. Phiri: Do you wash your hands when eating roasted meat?

C4: We wash at the beginning but after that we just continue doing what we do, because first of all we may not find so much water to wash our hands each time.

M: So when you are roasting meat when you go to the toilet you do not wash your hands?

C4: We wash our hands from the toilet, but when we scratch and touch other things we do not wash our hands.

Dr. Kabemba: What is Lunsa?

C3: It is Nsenga.

Lunsa means dirty in nsenga.

*Laughter*

M: How many times do you wash the body?

All: Twice.

M: Is that true?

All: Yes.

M: How many times and what times?

C8: In the morning and in the evening before sleeping.

Dr. Kabemba: How many times do you wash your body?

C3: Like I know myself like here in the village we cannot take a bath twice in a day. Most people bath once per day, like when I was leaving home I may not have taken a bath but when I go back I would take a bath also when I go to the field in the morning I may not take a bath, I come to take bath when I return from the field.

C8: Yes there are times when we have so much work and you cannot bath twice but in a normal day we manage to take a bath twice.

M: Let us say government says they would need to vaccinate all against worms can we manage?

All: We can manage.

C3: Because it is the government which brings medicine so that we can be protected from worms, we can manage.

M: What if the government says all pigs should be vaccinated can we manage?

C4: We can manage if those giving medicine can come to our homes, because it may not be possible for a person who has man pigs to manage to bring all the pigs.

C8: Yes like my friend has just said we can manage but if we were to bring the pigs here, that would be difficult.

C3: In addition there was a time when they came and said all dogs, pigs, goats and chickens should be vaccinated but what happened was that all the animals died, but it would be a very good thing if our animals are vaccinated and protected from diseases.

Dr. Kabemba: What if they say you should also contribute to the vaccination of your pigs would you pay?

C3: They must give me a chance to contribute in the next trip, to contribute towards the vaccination.

C8: Yes it would be manageable but that contribution should not be more than five thousand kwacha, because there are some people with more than twenty pigs that would be an astronomical amount, so some would just say well that is very expensive.

Dr. Kabemba: They would just sale two to cover the cost.

C8: What if the one you have sold is sick?

*Laughter*

M: What if government says we vaccinate all pigs but you must pay something towards the cost can we manage?

C6: Yes we can follow but it depends on how much they are asking for.

Dr. Kabemba: Now there is vaccination for cattle, do the people manage to pay?

All: Not all manage.

C3: Most people do not manage because they say one animal you have to pay fifteen thousand kwacha now if you have ten animals it means you spend one hundred and fifty thousand kwacha so it is better for me to buy my own medicine and vaccinate them myself. In the past what used to happen is that they would announce that al the animals should be brought at a central place to be vaccinated and people brought their animals for vaccination, but now because of the price people shun the activity.

M: Anyone else?

*Silence*

M: Thank you very much we have discussed a lot of things on pigs, the goodness of rearing pigs, the badness, the one who makes a decision to slaughter a pig, the diseases found in a pig, the way we can prevent the diseases affecting pigs, how we know that our pig is sick, we learnt about worms and how we can prevent this disease.
