# Supplementary material for: Why Latrines Are Not Used: Communities’ Perceptions and Practices Regarding Latrines in a Taenia solium Endemic Rural Area in Eastern Zambia
Source: PLoS Negl Trop Dis. 2015 Mar 4;9(3):e0003570. doi: 10.1371/journal.pntd.0003570 (PMC4352092; doi:10.1371/journal.pntd.0003570)
Supplement: S1 Dataset — (ZIP) [file pntd.0003570.s001.zip › FGD transcriptions_Zambia-2010/Nyazowani_Mr Sakala/Women_Nyazowani_02-08-10.doc]

**Title : Focus Group WOMEN**

**Date :** 02/08/10

**Site :** Kakiwa Rural Health Center

**Village :** NYAZOWANI	**Location :** Petauke district, Eastern Province, Zambia

**Duration (total time) :**	85 min	**- Start :**	12:00 pm	**- End :** 13:23 pm

**Participants:** 8 women

**Informed consent:** signed and available

**Moderator :** Ms Ruth Chiwa	**Recorder :** Dr Andrew Phiri

**Transcription/translation :** Mr Boniface Sakala

**Abreviations : M : moderator, R : recorder, I: intervener, C : children, W : women, M : men**

**Disposition (from the left of the Moderator):**

**M**

W1

**R**

W2

W3

W4

W5

W6

W7

W8

Age composition:

W1: the oldest

W8: old

W6: the youngest

All the rest: middle age

Typology:

W6-W7: shy

W8: quiet

W4: dominant

**Comments about participants :**

**Group dynamics :**

A lot of external interventions and especially from Mr Mvula who are the head of the health center… He reacted much more as a health worker as a FG facilitator.

**Text:**

The informed consent process was done with participants being videotaped.

*Introduction*:

M: Thank you very much for coming we receive you with both hands, before we start our discussion we need to know each other. I think you all know me how about you?

W1: I am Mrs. Mwale.

W2: I am Salome Tembo

W3: I am Tembo

W4: I am Eliza Phiri

W5: I am Amelina Zulu

W6: I am Betty Phiri

W7: I am Matilda Chirwa

W8: I am Grace Banda

M: Thank you very much all these have already introduced themselves, in this discussion we need to discuss what we know and what we do there will be no wrong answer because we are all different and think differently so you must be very free, discuss the things you practice at your homes and those which you do not do. Is there any question? Our discussion is based on the rearing of pigs, I will ask a question and you give the best answer.

*Discussion*:

M: Is it necessary to have pigs in our village?

All: Yes it is necessary.

M: Why?

W2: Because pigs are helpful.

M: They help us in which way?

W3: We rear pigs because when we sale them we can send our children to school.

W1: And also you slaughter a pig to use as relish in the home.

M: Anymore?

W1: If there is a funeral you slaughter a pig to assist you feed the mourners.

W4: If you have a daughter undergoing initiation you would sale a pig and use the money to assist and also you can slaughter the pig so that those who are helping can eat.

M: Anyone else, is a pig necessary?

W6: Yes it is necessary because there are many things which happen.

M: What are those things?

W6: There are many.

M: What is the benefit of rearing pigs?

W7: Some of us do not eat pork but we keep them to sale and assist us send children to school, help us buy clothes and many other home use utensils.

M: What do others say?

W4: A pig has two sides it is good and bad at the same time, yes we eat the meat but we do not eat the meat very freely, because while we keep pigs we do not keep them in a manner which is good. We were supposed to keep pigs in a kraal surrounded by a fence and give the feed so they are maintained in a clean environment but we let them out to scavenge on many things, it would follow a person in the bush eat the feces of that person who may be sick and we in turn would slaughter the meat and that disease which that person is suffering from would affect us if we eat the meat of the pig. Now that is the problem we want you to teach us and tell us the good rearing practice of pigs, so that we can prevent this from happening.

M: Anyone else the benefit of rearing pigs?

W8: Like it has already been said the benefits of rearing pigs is that we can sale and get some money to use in the home, also to assist us in the weeding our fields.

W3: I rear pigs because I want to sale and help me solve the numerous problems I encounter in my daily life.

W2: I rear pigs to eat as well because pork is very nice and taste meat. Yes we know all the bad things it eats but the meat is very good. The other thing is that pigs unlike goats has a lot of meat so when you slaughter a pig you would have a lot of meat, also if you know how to prepare pork it is very nice meat.

M: What is badness about a pig?

W2: The badness of a pig is that it eats bad things like our feces which we are throwing away.

M: Anyone else?

W1: It is called you eat me on purpose when you know the bad things I do, it would follow us and eat our feces but we do like the meat.

M: I want you to discuss the disadvantages of rearing a pig.

W2: She is asking for the disadvantages of a pig.

W1: The disadvantage of a pig is that it eats anything; it is our rearing practice which we need to be told. If you had a pig and had it penned and you cannot feed it properly it would thin and die in the kraal.

M: Anyone else on the disadvantages of pigs?

W8: The badness o pig is that when you are going to the bush it would be following you behind.

M: Anyone else?

W2: The problem with pigs is that they can transmit diseases.

M: How about at the graveyard?

W5: Yes they would go to the grave yard and dig up still born children which are not buried very deep.

W4: Yes a pig is bad in many ways like it has been said a pig would go to the graveyard dig up still born children and eat, there are some people if they eat pork they develop very severe rash. So all in all a pig though the meat is nice it is a very bad animal.

M: Does a pig perform any job?

*Silence*

M: Is a pig useful?

W3: The only job it performs is the financial help we benefit from.

M: Anyone else?

W5: The only job it does is when it helps us to solve our financial problems.

M: What dangers would be associated with a pig?

*Silence*

M: What frightening things would be associated with a pig?

W5: A pig can be associated with transmission of diseases.

M: Anyone else, C4?

W4: Even if I have a toilet and others do not have a toilet it is very difficult for one to provide prevention for the whole village.

M: Anyone else?

W4: Even if a pig helps us in many ways, at wedding ceremonies, initiation ceremony, funerals and other events it is not a good animal to eat because of what it eats.

M: Now I want you to concentrate on discussing the dangers that are associated with a pig.

W4: A pig is associated with eating still born children, it gives rashes to some other people these are the dangers of eating pork.

W3: Even at Kalindawalo a certain woman had a new born baby she left the baby laying on a reed mat got into the house to pick something, she found the baby had been stolen by a pig which ate it.

Kalindawalo = a nearby Chiefdom

R: Did the baby die?

W3: Yes, by the time the mother came she found it had already started eating it.

M: Do you keep any other animals apart from pigs at you home for you women?

W3: There are some other animals.

M: Like which ones?

All: Goats, chickens, cattle.

M: Just for you, like you C3 the cattle are yours as C2?

W3: Yes but I keep it together with my husband and I have a pig which is completely mine.

W1: I have my own cow as me.

M: Why do you have it as your own?

W4: Like there would be animals which my parents give me, we have not bought it with my husband but my parents gave me the animal, it therefore means I am the absolute owner. You cannot refuse a gift from your parents because you are married; you get it and become the absolute owner.

*Laughter*

M: Anyone else?

W8: Like me the chickens are mine.

W2: There are times when you have a small field of your own and when you harvest and sale the crops, you can buy your own animals.

W4: Yes, sometimes on your small field you would have such a good harvest that you would decide to sale part of the crop and buy a goat or any other animal that you want.

M: Do wives have their own field and your husband has a different field?

All: Yes, because of what happens in the homes, if you have one field and you harvest, the husband would not be fair in distributing the income that comes from that.

W3: It is because of these problems that women usually decide to have a small field of their own on the side so that you can use the money realized from the sale of your crops to solve your problems.

W4: Sometimes after you harvest the crops you would find that you cannot sit down with your husband to plan how the money should be spent, most times they take charge and then they do not spend the money according to the problems which you have at your home, that is then how us women would propose that we have separate fields. The men usually accuse the women that we are lazy and that is why the take charge of the money, but when we go our separate ways we find that the women harvest even a larger crop, and then we can now decide what to buy ourselves.

M: Do you take part in the rearing of pigs?

W3: We are the ones who feed them and pen them in for the night.

M: Anyone else?

W4: Like we have already said we are the ones who mind the pigs at home, but then if you husband is lazy and does not make a fence and a kraal that is why we leave the pigs roam outside.

M: What role do we play in the rearing of pigs, C6?

W6: I feed them maize bran and pen them for the night.

M: Are there any other people who help in the rearing of pigs in a family?

W4: Our husbands help in building a kraal.

W3: The children help in the feeding and penning them for the night.

M: Is it only one person who feed the pigs or it is done by one person?

W2: Only one person should be responsible for feeding the pigs it cannot be all, because some people have bad hands and the pigs may develop nsembe. So in all homes you choose two children who would be responsible for feeding them and penning them.

W4: What we have heard about *nsembe* is that it is caused by feeding pigs with *masese* which turn into *nsembe* in a pig.

W2: Does not agree with that.

W3: Some say that you can prevent nsembe by adding ash in the feed when feeding them.

W2: I have reared pigs on several occasions and each time it is me who feeds the pigs they would develop *nsembe* but if at our home I assign the role of feeding them to another person the castrates do not develop nsembe. I have therefore concluded that it is my hands which are bad and that it is when you have bad hands that the pigs develop *nsembe*.

M: Which other people assist in the rearing of pigs that are not from your immediate family?

W2: Some we think can help.

M: Do not think but those who assist in the rearing of pigs?

W3, W4: There is no body.

M: Who decides that this pig we should slaughter it, this one should be sold and that this one is for the child?

W8: The owner of the pigs.

W5: The one who takes care of the pigs is the one who decides that let us slaughter this pig and eat at home. Sometimes it can be the husband sometimes it can be the wife who suggest and a collective decision is reached

All: The children do not get involved in the decision making but they can be told as things go on.

W3: The children are only informed that we have this problem so we have decided to sale this pig, but they are not party to decision making.

W5: Informing them we do.

W2: If you have a child at home and he is lacking certain things, he can ask the parents to sale one pig to solve her problem, but that is just like asking for help not making a decision. Yes there if the case merits the proposed suggestion then their wish is granted.

M: Who feeds the pigs in a family?

W5: It is the woman.

M: Anyone else?

W8, W2: It is the woman who is responsible for feeding the pigs in a home.

M: How about the children?

All: the children participate in the feeding of pigs.

M: How about the men?

All: The men do not feed the pigs may be when you are not there.

M: Why?

All: Feeding pigs is usually a woman’s job.

M: Feeding pigs is a job for the women.

W4: The work of men in the rearing of pigs is to build kraals for the pigs a job which they fail miserably.

R: Have you ever asked your husband to make a fence?

W4: Many times.

All: They fail every time you ask them.

W4: Right now I have pigs at my home and he has failed to make a fence.

R: Can I ask him when he comes?

*Laughter*

W4: Yes Please.

*More laughter*

W4: A person who is so difficult cannot come here he has remained at home.

M: How many times do you feed the pigs?

W4: He cannot come because he knows that whatever he would come and say here is a lie.

M: Madame C4, how many times do you feed the pigs?

W4: Three times, I feed them in the morning, mid day and in the evening.

M: What type of food do you feed them?

W4: I feed them maize bran.

M: Anyone else?

All: Sometimes we feed them bran, left over and pumpkins

M: What good do you see from a pig eating human feces?

All: There is nothing good.

W4: There is nothing good in any way about that.

I (Dr. Mwape): What is bad?

All: It is bad for a pig to eat human feces.

All: If you do not have feed you can buy maize bran to feed them than them eating human waste.

M: Where do you buy the maize bran from?

W3: You buy it from the grinding mills.

M: What other bad things do you see from a pig eating human feces?

W4: We are in the village so what happens is that a pig would come from eating feces in the bush and then it would come and eat in kitchen utensils. Sometimes you would be preparing to eat nshima and put it outside, when you just enter the house for a second it would come and eat in the plates; as a result you throw away the food. That in my opinion is not good, so even if rear these animals they are not good, we do not know which diseases it transmit we just fall sick.

M: Now from the perspective of the pig which eats feces, what is its life?

W4: In my own opinion even if it is very profitable to rear pigs but because of its life style of eating feces I think it would be better to think of rearing other animals which may be profitable than rearing a pig which have very good financial gains but bring diseases to the households.

M: What dangers would you think of from a pig which eats feces?

W4: The problem I want to discuss is that eating meat from pigs that feeds on feces can cause a lot of problems, because these same pigs come and eat from our kitchen utensils and contaminate them with all kinds of diseases. These diseases are transmitted to us without knowing, this circle continues without a solution.

M: How regularly do you eat pork?

All: Not that regularly there is a time when we eat a lot of it.

M: When is that time?

W4: Most times people buy live pigs.

W8: When you have money you buy pork and eat.

M: Madam C4 is pork eaten regularly?

W7: Those who eat may be able to say because I do not eat pork.

M: Madam?

W6: Like this time when there are so many roasting stands.

M: What other time?

W3: During the Christmas festive season, that is when people eat a lot of pork.

M: Any other time?

W6: During Christmas and New Year.

W2: At a funeral.

W4: When you slaughter a pig and give the meat to people so that they can help you with the weeding.

M: Do they eat a lot of pork during a funeral?

All: Yes, pork is consumed in large quantities during a funeral.

M: Do you all eat pork?

All: No, but some of our family members eat pork.

M: Where does this pork come from?

All: The meat is usually bought from meat vendors who slaughter pigs.

M: So for all of you here you just let the pigs grow without slaughtering some?

All: We are the ones who supply those with roasting stands we sale them live pigs and they slaughter them themselves.

M: If there is a basket of meat, the vendor has brought what do we look for when we want to buy the meat or decide not to?

W1: If we have the money we buy the meat, the vendors bring.

M: You buy any meat?

All: Meat which is not good is seen, even if they say we just slaughtered it the appearance of the meat is reddish, an indication that the animal was sick and died on its own, even if it is beef.

W4: the meat which was sick we can see it because the meat does not look well.

M: What other signs do you look for?

W8: The meat does not look nice.

M: C7?

W7: Well the only people who eat the meat are the ones who are suppose to buy and know the appearance of the meat.

M: If a basket of meat comes I would peep and make a comment whether the meat is good or bad even if I do not eat the meat.

All: That is correct.

W7: It appears red.

W3: Some of the meat’s vendors sale meat which is packed in plastics and some just leave it in the dish after cutting into small pieces and cover it with leaves then the flies have a field day.

R: We want to know what you are looking for in the meat before you buy the meat.

All: the meat appears red and sometimes even the blood appears red rose.

R: You are not being to the point.

W4: We are not to the point?

M: Yes, we want you to tell us how do you judge that this meat is alright and that this meat is good and what do you look for?

W1: you look for fat in the meat and how it was processed if the skin was well done.

W4: Sometimes the meat has a reddish appearance or sometimes you were seeing the pig sick and the slaughter it.

W1: When you see the fat there you can buy.

I (Dr. Mwape): How does meat from a sick pig looks like?

W2: Sometimes it looks red, sometimes it would have no fat and sometimes it would have red blood coming out.

W2: Sometimes it has nsembe.

W1: The skin would have more folds that necessary.

W3: the skin and the flesh would have separated to an extent that when you hold it in you hand and shake the flesh would be moving on its own.

W4: The skin would have separated from the flesh because of being thin, the meat which is okay the flesh and skin would be attached.

M: Is this pork examined?

All: It is never examined.

M: Why?

W4: There is no inspector.

M: There are no machines or there is no inspector?

W4: There is no inspector, if they would come and condemn the meat why should we eat that kind of meat.

M: Do these people who inspect meat come on their own or we have to call them?

W4: we have to call them.

W3, W2: We have to call them that I am slaughtering a pig can you come and examine it for me.

M: Do you call them for free?

W3: We are not sure.

W4: If you seriously wanted you r meat examined you would go to the veterinary offices report that each time you want to slaughter a pig you would want the meat examined, then they would tell you the what you should do if you have to pay them money for them to come they would tell you.

M: Would you be happy if the meat was examined?

All: Yes we would like that.

M: Why?

W4: Because it brings a lot of diseases, we would then be protected from those diseases which are found in a pig which we cannot see.

W8: So that we can eat pork with confidence, knowing that we are eating good meat.

W5: Because to the naked eye the meat may look good when in fact it has a disease.

M: Mr. Mvula knows how to examine meat.

Mr. Mvula = the Catchment Environmental Health Technician

W4: That would be very good.

M: You would not hate him if he recommended you meat to be thrown away?

All: No

*Laughter*

W4: If you Mr. Mvula you condemn the meat you must wait and see that the met is being burnt, because there are so many diseases here and we do not know where they come from, some of the things that bring us these diseases we keep them ourselves. We would definitely be very delighted if the inspections are done.

W1: That would be very good because Mr. Mvula is nearby.

M: How do we cook pork?

W2: Pork must be thoroughly cooked for a long time.

M: How about others, even if you do not eat how do you see those who cook pork prepare it?

W8: When you buy pork you cut it in small pieces put the pieces in a pot, cook it for a while and when the water is dry you fry it and then and more water to boil for some time and finish.

M: Anyone else?

W6: You cut the meat into small pieces put them in a pot add water and salt leave to boil for some time and when the water dries you fry it for a short period until when the meat is brown, add some tomatoes and onion and leave them to cook for some time.

W5: You add a bit of cooking oil to bring out the flavor.

W4: Considering that this meat has a potential to carry diseases you boil it much longer adding a bit of water slowly each time until when the meat is thoroughly cooked. But even if we cook the meat there is still a problem because most people like roasted meat which does not cook thorough, especially those who drink just eat partially roasted meat which is very dangerous because the bacteria in the meat is usually not killed by this method of cooking.

M: Why do you think people like roasted meat?

W2: The people who sale roasted meat say they make more money this way.

W4: Because of the size of the pieces the traders cut they make more money.

M: How about those who buy the roasted meat?

W4: The people who buy the roasted meat are usually those who drink and the young men, but as for us the women we prefer to buy raw pork and cook it ourselves at home.

W8: I personally do not think roasted meat cooks thoroughly.

M: The women do not participate in the roasting meat business?

All: No.

M: You do not roast the meat?

All: No.

M: How about that meat which you roast for your husband’s?

W3: But that meat we usually cut it in small pieces and put on the pan and fry it.

W4: Well roasted meat has got a problem whichever way we prepare it, because the point is it does not cook thoroughly compared to boiled meat.

M: So you do not please your husband’s?

*Laughter*

All: We do in another way.

R: So you do to please your husband and if so in which way?

All: We do, sometimes we leave a few pieces for the husband.

W1: Yes we do, when a husband comes back home we would give him a few pieces to taste if the salt is okay.

All: Sometimes you leave some pieces for him to eat in the morning.

W4: But some of the young women do not give their husband’s a few pieces to taste salt.

All: Some still do that even now.

*Laughter*

I (Dr. Mwape): What if your husband comes back drunk with a piece of meat and asks roast it but you notice that it has masese what would you do?

W4: If he was not drunk I could stop him but if he is drunk there is nothing I can do but to obey his order.

W3: That is difficult because it is very difficult to argue with a drunk.

W4: May be the meat has even been soiled because as he is coming he may have been falling down.

M: Have you ever seen *nsembe* or *masese* in pork?

All: Yes we have seen them.

M: You have seen them?

All: Yes.

M: What do you think about *nsembe*?

W4: We think this a disease which affects even us human beings, it is bad.

M: What do you think of nsembe?

W5: It is bad.

W1: You can see on the picture they are so big and they would be popping open as you cook.

M: What do you think of *nsembe* in the meat?

All: The only way we do it is to throw away that meat because it is not good; sometimes it is so disgusting even to look at the meat.

W3: It looks like some gems.

M: Have you ever eaten meat with *nsembe*?

W4:No. When you just see it you cannot buy it.

R: How about eating it?

W4: If you ever see meat with *nsembe* you cannot buy it, it is bad.

W1: Just like you are seeing in the picture this is bad meat you cannot even buy it.

M: But some say if you cook it, it is very nice.

All: That is not correct.

M: And some say it is very nice when you roast it.

All: May be because we are different but it is not nice.

W2: Meat that has nsembe does not stop dripping water even when you roast it, because of the same *nsembe*.

W5: Some say they would want to dry it and eat the meat.

W2: But the water does not stop dripping still.

I (Dr. Mwape): How about the taste?

W4: It cannot have a good taste, because if a veterinarian examined the meat they would tell you that the meat has a disease, how can it taste well?

I (Dr. Mwape): How does it feel in the mouth?

W2: These feel like you are eating grit.

I (Dr. Mwape): So you have eaten the meat before?

W2: Yes, I have, it does not taste well.

M: Do we have toilets at our households?

All: Yes.

M: All of us?

All: Yes

M: If we have, do we use the toilets?

All: Yes we do.

M: The whole family?

W3: In our homes according to our family yes we do.

W2: if there is a son in law he would have his own toilet and you and your children another one just like that.

M: What about us who do not have what do we use?

W4: I have had mine just dug recently so at the moment I am using my neighbour’s toilet.

M: With your entire family you use the neighbour’s toilet?

W4: There are many toilets in the village which they can use, like them and their sisters cannot use the same toilet.

M: So in your village, do you all use toilets?

W1: Yes the toilets are there.

M: In the village do all people have toilets and nobody goes anywhere?

W1: I have a toilet and C3 has a toilet, I believe all the people have a toilet.

M: Mr. Mvula you have heard you should go to their village and check if all have toilets?

W1: Let him come to check.

M: All of you have toilets in your village?

W1: Yes.

W4: I think we should say it is the only village where I have seen where there are toilets; we should thank them for a job well done.

M: In Nyazowani village you have few toilets?

W4: Yes.

M: Where do those who do not have toilets go to?

W4: In other people’s toilets.

M: So all the people go in other people’s toilet?

W4: Yes, there are fewer toilets like I have just had mine dug and the only remaining work is to build a hut over it.

M: Why can you not use the same toilet with children and your son in law?

W2: We fear meeting in the toilet with your son in law.

M: Why?

W2: it is just not respectful

All: It is different from town and here in the village, here it is viewed as being disrespectful to use the same toilet with your son in law.

W1: Let us say you have diarrhea and you mess the toilet and your son in law enters the same toilet? Here in the village that is not acceptable.

M: Why do you use toilets?

W4: The reasons for using the toilets are many, say right now those leaves you see will soon be burnt, therefore there will be very little place to hide because if you went into the bush every passersby will see you squatting down.

W2: The other reason relating to our discussion is that say you go behind the house and you help yourself, then a pig comes and eats your feces and supposing you left your kitchen utensils outside. The pig would come and feed from the utensils there by contaminating with feces or a disease, so we use toilets to prevent diseases which would be as a result of pigs eating feces. In the evening the toilet is cleansed with would ash.

M: We have said we cannot use the same toilet as children?

All: Yes.

M: Does it mean the neighbor has no children?

W4: My neighbor whose toilet I use has two small girls.

R: Which toilet do these children use?

W4: They go to the same toilet.

R: Are you not afraid of meeting them?

W4: There is still a very big difference with your own big sons.

I (Dr. Mwape): Why can you not use the same toilets with you older children?

W2: In the Nsengaland it is considered very disrespectful to use the same toilet with your grown up children, it is always good to have two separate toilets one for the older children and one for you and your husband and the younger children.

W4: In town we can use the same toilet with your in law but here in the village we still follow our Nsenga cultural norms which do not take kindly to using the same toilet with your son in law.

R: You are not afraid to meet your son in law?

All: You just bend your head down in shame, when that happens.

*Laughter*

W4: You just feel the shame of what has transpired.

I (Dr. Mwape): The men do not take an axe, madam give me my axe?

*Laughter*

W4: To do what with the axe?

W2: Yes they do while you think that he is going to fetch fire wood, when he is going to help himself.

W4: They do not even ask you for the axe, they just go pretending that they are going to fetch for fire wood and they come back with a small piece of firewood on the shoulder when in fact the whole mission was to go and help oneself in the bush.

*Laughter*

W4: We all do realize that this is not a good life of going into the bush.

M: Whose job is it to make sure that the children are bathed in a home?

All: That is a job for the woman.

M: Do you think it is important to bath the children?

All: Yes.

M: Why?

W2, W3, W4: It is very important because the dirty on the skin can be a cause of many problems, sometimes children can develop a fever because of not having taken a bath.

W3: Some children you really have to chase them if you want to bath them, no the older ones do it themselves the problem is with the young ones.

W2: We bath our children because we want the poles on the skin to be always open so that the body can inspire from all the points in the body.

M: So after bathing the child you let them the same dirt clothes that he was wearing?

W4: What should happen is that after you bath the child you should take a clean pair of clothes to change.

M: At what age do we stop bathing a child?

W3: We usually stop bathing them after seven years.

W4: A child who is twelve years is the only one who can manage to bath it’s self anyone below twelve should be bathed.

M: How about washing their clothes?

All: It is at the same age.

M: What would you say about the same children washing their hands when they come from the toilet?

W4: This is a very difficult job when they are very young, you would tell them to wash their hands when they come from the toilet, but usually with the young ones below twelve years they would not do it that well but we do tell to wash their hands if they come from the toilet. The one who take this very seriously are the one who are twelve years and older.

M: What do others say?

W8: We tell them to wash their hands when they come from the toilet.

I (Mr. Mvula): Grandmother what do you tell your grand children about washing their hands when they come from the toilet?

W1: I do tell them to wash their hands when they come from the toilet, those who listen do it but others would just brush you down.

M: Why do the children have to wash their hands when they come from the toilet?

All: Well because they are coming from the toilet where they could have come in contact with dirt.

I (Dr. Mwape): The old man when he comes from the bush does he wash his hands?

W4: He knows the rules so he must wash his hands.

W3: He must wash his hands.

W2: That fire wood which he has been carrying he should wash his hands to remove the dirt.

M: Is it necessary to wash your hands before eating food?

W2: Yes.

M: Do we manage to wash our hands before eating?

All: Yes.

M: When eating nshima?

All: Yes.

M: But not when eating groundnuts?

W4: In case of groundnuts we just unshelled and eat, because they are dry.

W8: But the fresh ones.

W4: The fresh one when you dig them up you just remove the shell and eat but when you cook them, you sit down take water and wash your hands to eat.

M: How about fresh maize?

W4: With fresh maize it is absolutely necessary that you wash your hands after removing the covers.

R: Do you wash your hands?

W4: I was saying so because it would seem like I am just answering her for this workshop, when in practice I do not do it.

M: Now you said when things are dry you do not wash your hands you only do it when they are wet.

W4: Yes when I get groundnuts from the grain store I just shell and eat I do not wash my hands.

W3: But in normal practice we must wash our hands before eating anything.

W4: Well I am being very honest I have never washed my hands before eating groundnuts from the grain store.

M: Any other additions from what she has said?

W6: No that is the truth.

W1: I think she has said the truth, most times when we are getting groundnuts from the grain store we just get one, shell and eat without washing our hands.

M: Do we know worms?

All: Yes.

M: Have we ever seen them?

All: Yes.

M: Where have we seen them?

W3: Sometimes when a child or an adult goes to the toilet they would come out to gather with the feces.

M: Anyone else?

W4: I saw it in the feces of my child.

W2: I saw it after vomiting it myself and I brought it here at the clinic.

M: How do these worms look?

W5: Some are reddish and some are white.

W4: Some are yellowish, one of my children coughed it out and it started to wriggle.

M: Where do we think these worms come from?

W4: they must come from the dirt we eat.

M: Where do the worms come from?

W1: I cannot lie. I do not know where they come from.

M: In our mind where do we think they come from?

W4: They must come from the food we eat.

M: Have you seen the pictures?

All: yes

M: Have you seen those white things?

All: Yes.

M: Have you ever seen them in someone’s stool?

All: No.

M: What you are seeing in this picture is the start of a worm, which looks like a wood borer. Do we know where worms come from?

All: We do not know.

M: We just find someone has worms?

All: Yes.

M: Do you think the person was born with them?

W2: we just think it is one of the diseases.

W4: We do not think children are born with them, because if an old person can be crying from their infestation how about a small baby, these come from the food we eat.

M: What food do you eat that can cause worm infestation?

W4: There are different kinds of foods that we eat that would be responsible for worm infestation; it is very difficult for us to blame one particular food.

W1: If we cannot point to a particular food it is better you tell us that food which causes worms if we talk so much they will not tell us.

W4: that is what we are saying.

M: C7, what do you think causes worms?

W7: I do not know.

M: My sister *(C6)*?

W6: I have heard that if you eat soil you may suffer from worms.

M: What do the people who have worms suffer from?

W4: Loss of appetite.

W1: some become very thin even when they eat well. We had a case of a small child who was infested with worms he just kept growing thin and we did not know, but one day when he cried for some tea after drinking the tea a worm came from his nose and then when it was taken to the hospital a lot of worms came out, we do not know what causes this terrible disease.

M: What do people who suffer from worms complain of?

W2: Stomach pain, rumbling of the stomach and movement in the stomach before you eat.

W4: Some lose weight, appetite and complain of stomach pain.

M: Does the movement in the stomach look like child’s movement.

W2: No a child moves differently, it like the intestines are moving.

M: Do you think worms can be prevented?

All: Yes.

M: How?

W5: By going to the hospital.

M: Is there medicine for the worms to come out available?

W4: Yes, the medicine is there when we go to the clinic we are given the medicine to remove all the worms.

M: Is this a frightening disease

W4: It is dangerous because if you vomit it and bite it.

W2: They say if you bite it you would die.

W1: Yes, that is what they say.

M: Have we ever witnessed someone who died after biting a worm?

W4: We have never seen anyone.

W2: We have just heard that one can die if they bite the worm while vomiting it but we have never witnessed someone dying from that.

M: Is there a disease which is greater than worms?

*Silence*

M: Any diseases that we know which are greater than worms?

W4: There are chest pains, back ache, coughing, malaria and many others.

W3: There is Malaria and fits.

W4, W2: There is a headache; coughing and sneezing these are what would be tamed as minor ailments.

M: Do you think that there are many people who suffer from worms in our village?

W3, W4: It would be difficult to say, because most times you are not well aware if the problem affect another home but when it affects your home.

M: Do you think it is very frightening if someone is suffering from worms?

All: It is very frightening.

M: Why?

W4: It is because these worms bite the patient and they would keep the patient sickly, there is nothing good about having worms.

W8: The person does not feed well and if it is a child does not grow up well.

M: In everyday life what problems would a person suffering from worms face?

W4: If you are not feeling fine especially in the stomach, you would go to the hospital and sick for assistance.

M: We are talking of problems the person would face in their life if they had worms?

W1: The person would die.

W4: The person would die, if it is a young person they do not grow up well, usually their body does not look bright.

M: Do you know masese or nsembe?

All: Yes.

M: Have you ever seen them?

All: Yes.

M: Do you think this disease affect only animals or humans too?

W4: This disease affects even humans, since we eat those animals.

M: C6 what do you think of this disease, does it affect humans as well?

W6: I think that it also affects humans.

M: Do you know fits?

All: Yes.

M: Where does it come from?

W6: From pigs.

M: How does it affect humans from a pig?

W5: My own analysis is that it is transmitted through pigs by eating pork with nsembe.

M: What do others say?

W4: Yes many diseases can be spread by a pig, if a child suffers from fits the medicine man would usually ask that the child should not eat pork, so it is possible that fits could come from pigs.

M: Why do you think they stop a child from eating pork when he suffers from fits?

W4: When the child is fine the medicine man does not stop them from eating pork, but as soon as the child starts fitting the medicine man would stop the child eating pork which means they know that pork is a cause.

R: Who stops the child from eating pork?

W2, W3: The people who treat the disease.

R: Where do they get this medicine?

W3: They dig from the bush.

W2: they stop the child from eating pork and anything that dies on its own

R: Are they found in the villages?

W3: Yes they are there who treat fits.

I (Dr. Mwape): What type of doctors are these?

W3: These are traditional healers

I (Dr. Mwape): local medicine?

W2, W3, W4: They are the ones who say do not give them pork or meat that dies on its own.

I (Mr. Mvula): Why do they stop the person from eating pork?

W3, W4: they know that pork is the one that is a problem.

I (Mr. Mvula): They know?

All: Yes they know.

M: Where else can we get fits?

W4: Sometimes you can be bewitched, sometimes you would suffer from malaria and the body temperature is too hot the person would start to fit.

M: Do you know anybody with this disease?

All: Yes

M: Do you know them, where do you think they got this disease?

W4: The disease could come from any of the foods we eat.

M: Please mention these foods.

W4: Pork, hare meat and bubble fish.

W2: Some say do not eat bush meat.

W4: So when they stop you from eating these foods it means they know that they are the ones which could be the cause of this problem.

M: Can this disease be prevented?

All: They are preventable.

M: How?

W3: There are medicines in given by the traditional healers if these fail the hospitals has medicine to mitigate the disease.

W4: It is like she has explained we have a similar case where we tried traditional healers and when it failed we took the child to the hospital where it is receiving attention.

M: Does this disease get cured?

W5: Sometimes they get cured and sometimes not, but us when we went to the hospital at the boma they told us that the child has very little blood, they gave it blood and a drip of water after that the child has never had fits from last year up to now, so we can confidently say yes they get cured.

M: Anyone else, can fits be cured?

W2: Yes, sometimes, those who do not get cured are those who are bewitched we have a person who started fitting when they were very young but now is a fully grown adult and has continued suffering.

M: Anymore additions?

*Silence*

M: Is it frightening to have this disease?

All: Yes it is very frightening.

M: How?

W2: You cannot sit near a fire; you cannot draw water from a river if you fall in any of these you would die, sometimes when you are walking on the road you fall down, that is why it is very frightening.

M: Do these people get married?

W4: They do not get married, if it is a man it is worse.

W3: Unless the disease starts when I am already married but if it started when I was young it is difficult to get married.

I (Dr. Mwape): Why?

W4: Because it is very difficult for a person suffering from fits to perform any house chores.

W3: Unless the disease starts when you are already married.

R: Would the husband marry two wives?

W3: Some would marry two wives but some would just get on.

M: Is this disease more dangerous than any other disease or is there a more dangerous disease than this?

W4: The diseases are dangerous but fits are more dangerous because a person suffering from fits has no peace, they can fall down any time if it is a woman or man you cannot get married you remain with your parents, now if you do not have parents you would die fast because if you fall on a fire or water that would be the end.

W3: There is no one to take care of you so you have a very short life because if you have no parents there would be no one to take care of you.

M: Which is a lesser disease compared to fits?

W8: Malaria, because if you go to the hospital they give you medicine you would be cured.

M: Do you think there are a lot of people suffering from this disease?

All: No that many.

M: Do you think this is a very serious disease?

W2: This is very serious disease.

W4: For some people who see this disease from very far they may down play it but if your family member is affected by this disease then you appreciate the severity of this disease and how bad it is, it is an extremely very bad disease.

M: In everyday life what challenges does a person with fits meet?

All: A person suffering from fits has no peace because they may fall any time.

W1: person suffering from fits is usually not one hundred percent normal, and sometimes a person would start fitting when you are about to make love, that is why such a person cannot stay in a marriage.

W4: Even when a disease like fits finds you already married sometimes a marriage of many years can breakdown because of fits, your partner may not be ready to continue with the marriage.

M: If to prevent worms and fits the government came up with a ruling that all those who rear pigs should make a good kraal with a fence around it what problems would we encounter in our village?

All: That would be very welcome idea.

W4: Even those men who are lazy in putting up a kraal and a fence round the kraal would be compelled to do so because government has brought a ruling.

M: If the government brought a rule that all households should have a toilet and they should use it what problems would be encountered in our village?

All: That would be very good.

W4: There is no problem; in fact every home should have a toilet.

W5: It means you have respected yourself.

M: There would be no arguments?

All: Yes.

M: What if we say from now on all the people who buy meat must boil it thoroughly well, what problems would be encountered?

All: there would be no problems.

W2: there would be no problems because you are trying to protect us from the ailments.

W4: It would be beneficial to us and not you who are teaching us so there would be no conflict.

W2: This would help those who were no cooking the meat thoroughly, but for us who have been doing it would only serve to remind us to do even better.

W4: There are those who do not understand and need to be helped in the way they cook the meat.

M: What if they say all the animals which are slaughtered should be examined?

All: We would love that.

W4: We would love it because then we would know why we were having a lot of problems in the past, because it is the animals which we rear which are giving us problems.

M: If we were told that all vegetables should be washed, what problems would we face?

All: there would be no problems.

W4: Normally all vegetables need to be washed before cooking, I may be cleaning at my home but some people may find the practice not acceptable. Some people would just look at the vegetables and without cleaning them put them in a pot and start cooking that they are not dirt but hey may not know if a snail crawled on the leaves.

I (Dr. Mwape): Do you wash pumpkin leaves?

W4: Yes, pumpkin leaves we clean them before cutting, then put them out on drying place to remove the water.

All: None.

M: Do you not think that people may complain that we are being troubled?

All: No.

W4: This personal hygiene is good for all the people, but some people would think that those telling them are just bothering them, some would look clean when they move aroud but if you went to their home you get surprised at the firth you find at their home, you cannot even drink water from their home.

W1: Some you would find them very clean but look at their child, the child would be un washed and very dirty.

M: How many times do you take a bath?

W3: Here in the village we bath only once.

W4: We bath only once in the evening because during the day we are very busy with our different chores.

W3: To speak the truth we bath only once.

W4: If you take a bath during mid day it either means you are going somewhere or there was very little manual work that day, but most times we bath once in the evening per day.

M: How about if we are clean the whole day like today it means in the evening we shall not take a bath.

W3, W4: When coming here we just washed our feet, so when we go back we must wash our bodies.

*Laughter*

W4: We just washed our feet, face and hands so we shall wash our bodies when we go back.

*Laughter*

M: What if all the people were told to receive a vaccination against worms what problems would be there?

All: None.

M: Would people agree to receive the medicine?

W4: Yes they would agree, why they should refuse.

M: There would not be talking?

W4: You just bring the medicine those without worms and those with worms should all drink the medicine including the mothers.

M: What if they say that all the pigs should be vaccinated what problems would be encountered?

W4: there would be no problems, me I had my pigs vaccinated sometime back.

W3: Even me I went to the veterinary offices to buy medicine.

W1: they should do it because it is the law.

I (Dr. Mwape): What if the government brought medicine and asked you to pay for the medicine, would you pay?

W4: Yes we would pay. We would do everything in our power to make sure we pay so that the pigs are health.

I (Mr. Mvula): Why do you not pay for cattle?

W3: For cattle we just go there to buy medicine and inject them ourselves.

I (Mr. Mvula): Where?

W3: At the boma.

I (Mr. Mvula): Fish?

W3: Yes.

W4: We had a problem with my animal after treating it with *berenil* and it was not looking fine we took it to the TT offices where they had it examined and treated it, right now it is fine.

TT = a term used for local people who were trained to offer primary veterinary care in the absence of trained veterinary personnel.

I (Mr. Mvula): To the TT, that was very good.

M: What role does TT play in the role treatment of cattle?

W2: TT people worked for a long time with people who were stationed at Nyamphande, so they gained a lot of experience in the treatment of cattle and in this area they act like the veterinarians, who assist with all problems concerning cattle.

M: We have now come to the end of our discussion, from the time we started we discussed why we rear pig, the goodness of rearing pigs, what do pigs eat and who feeds them, we talked about masese, how does masese come, we talked about toilets and the goodness of a toilet, we talked about personal hygiene, why we wash our hands when we come from the toilet, why wash our hands before eating, we discussed about worms, we discussed about fits and we all also discussed the measures we would take to prevent fits and worms like restraining our pigs in the kraal so that they do not wonder about, boil the meat and wash the vegetables and lastly we discussed about vaccination of pigs. If you have any questions you can now do so.

W1: We can only say that this has been very well worthy while programme to enlighten us about how we should look after our animals. All was very clear and we would have no further questions.

M: Thank you very much.
