# Supplementary material for: Why Latrines Are Not Used: Communities’ Perceptions and Practices Regarding Latrines in a Taenia solium Endemic Rural Area in Eastern Zambia
Source: PLoS Negl Trop Dis. 2015 Mar 4;9(3):e0003570. doi: 10.1371/journal.pntd.0003570 (PMC4352092; doi:10.1371/journal.pntd.0003570)
Supplement: S1 Dataset — (ZIP) [file pntd.0003570.s001.zip › FGD transcriptions_Zambia-2010/Sikalinda_David Mwanza/CHILDREN Sikalinda-edited-kem.doc]

**Title : Focus Group CHILDREN**

**Date :** 01/08/10

**Site :** Kakiwa Rural Health Center

**Village :** SIKALINDA	**Location :** Petauke district, Eastern Province, Zambia

**Duration (total time) :**	55 min	**- Start :**	11:08 am	**- End :** 12:03 am

**Participants:** 8 children

**Informed consent:** signed and available

**Moderator :** Ms Ruth Chiwa	**Recorder :** Dr Andrew Phiri

**Transcription/translation :** David Mwanza

**Abreviations : M : moderator, R : recorder, I: intervener, C : children, W : women, M : men**

**Disposition (from the left of the Moderator):**

**M**

C1

C2

C3

C4

C5

C6

C7

C8

**R**

Gender distribution:

**C1-C6-C7-C8**: females

C2-C3-C4-C5: males

Age composition:

All are between 14-17 yrs

C3: the oldest

Typology:

C8: rambler

C3: expert, funny

C1: dominant

C5: shy, quiet

**Comments about participants :**

The oldest (18yrs), average age 14yrs

**Group dynamics :**

Very lively lot

Level of participation was very interactive apart from C5 who is not at school; at 12 hasn’t been to school.

Reluctance and difficulties in describing type of worms, difficult differentiating tape worms from other types.

- Laughter about toilets, their use and pigs confinement.

- Laughter concerning decision making of pigs welfare and slaughter.

- They showed disgust about pig indiscriminate eating habits.

Used local language, two even used English expressions.

Discussion was spontaneous kids were relaxed.

**Text:**

Introductions of the mission and informed consent done

*Introduction*:

M: Morning girls, morning boys. You are welcome. I am so thankful that you have come so that we can talk about pigs. Firstly, before we get into details, we need to know each other, not so?

All: Yes

M: Since you know me, is that not so?

All: yes

M: You can introduce yourselves.

*Children introduce themselves*

M: We have visitors here; do you want to know him?

All: Yes

*R walks away.*

C3: Yes, We want to know

*R comes back to sit*

M: You do not know them?

C3: Yes, we want to know.

M: Dr Mwape, they want to know you.

I (Dr Mwape): Oh, I am	Mwape, are you getting me?

All: Yes.

I (Dr Mwape): I am Mwape and I come from Luapula province but I live in Lusaka.

C3: From Luapula?

I (Dr Mwape): Do you know where Luapula is?

C3: No.

I (Dr Mwape): Yes, you wouldn’t know Luapula, because it’s very far, .but I stay in Lusaka. This one is Severine, we work with her but she comes from abroad. But you know this one *(Pointing to Mr Mwanza)*.

All: No, we don’t.

I (Dr Mwape): Oh, you don’t know this one? This is Mr. Mwanza. He lives here, you don’t really know him?

C7 and 8: We know him.

I (Dr Mwape): You know him?(*laughs*)

All: Yes (*amid smiles*)

M: So now you know them?

All: Yes

M: We want to talk about pigs. I’m asking you to be very open and free children. You need to say what you know. There is nothing scarily. Is that not so?

All: Yes

M: Now I’ll ask you a question, is it important to have pigs in our village?

C1: No, it is not important because they have diseases.

M: Others who can add on?

C7: It is important.

M: Why is it important?

C7: Because we eat, its food.

M: Yes (allowing C6 to speak)

C1: Because it is God’s creation.

M: Ok, because we eat. Any other? Yes! (*Points to C3*)

C3: It is important for business. For me, I did not have school fees and this troubled me very much *(all smile)*, so I had a K50 000 ($10) and I went to buy a pig and I sold the pork in very small pieces at “shokas”. It got finished and out of that money, I realized a K100, 000 ($20). It really helped me. I paid at school. We get help with school fees.

M: Brother (point s at C2), you said that it is not important, why?

C2: Because it brings diseases.

M: Who else can add?

C1: It is important because we get money to have the field cultivated.

M: What are the benefits of rearing pigs?

C7: It helps.

M: Helps in which way?

C7: Like when we have problems, we slaughter and it helps sort out some of these problems.

M: Like what problems?

C7: Funerals.

M: Who can add on?

C2: It is better to kill goats during funerals, not pigs.

M: Others? Yes C4?

C4: They are good, they need to be reared.

M: What is the badness of rearing pigs?

C6: If there are feces, pigs will come and eat

C5: Because it eats feces of different kinds.

M: Any other?

C3: Pigs are both good and bad. It is bad because it eats feces. But what is needed are fences so that pigs do not move anyhow. So we need fences so that they should not be going outside.

M: Who wants to add on?

C7: There is need to dig pit latrines so that they do not eat feces.

M: Anyone who has something to say?

*Silence*

M: What are the threats that pigs pose to people? What are the dangers of pigs?

C1: It brings diseases.

M: It brings diseases, like which ones?

C1: Epilepsy

M: What else? What are the threats of pigs?

C4: It has *nsembe*.

M: Who else?

C7: It brings cholera

C1: It also brings a mysterious cough.

C4: Also brings flu…

M: Yes, what else?

C5: Stomach problems.

C3: And even diarrhea.

M: What are the uses of pigs?

*Silence*

M: Are pigs useless?

All: No, they are useful.

M: What are the uses?

C7: It helps.

M: In which way?

Children laugh with panic

C8: You sell a pig and buy the household goods needed in the home.

M: Others, yes C4…

C4: Even when you have a case against someone, you sell a pig and pay the charge.

M: Others, lets add!

*Silence*

M: Do all your parents keep pigs?

All: Yes, they do.

C6, 7: Us we don’t.

C6: They finished due to problems.

M: Whose parents keep pigs here?

C4, 8: Our parents do.

M: But parents to all of you have once kept pigs, not so?

All: Yes

M: Why do you think parents keep pigs?

C4: To get helped in time of need.

M: Getting helped In which ways, you need to explain.

C7: During times of problems, you just get the pig sell it and been helped.

M: Like which problems?

C6: If there is an initiation ceremony, on the last day, they kill a pig/s for the feast.

C4: If you do not have maize for mealie-meal in the rain season, you sell a pig in order to buy mealie-meal.

C8: During funerals, pigs help.

M: How do we keep pigs?

C7: We build an enclosure and start giving the feed.

M: Who can add on?

C1: Some are kept in the fence.

C2: Give them food so that they do not get outside.

M: What do you think about pigs that just roam around? There are pigs that just roam around and they eat feces. What do you think about those kinds of pigs?

C6: They are given food. They should be fed from inside the enclosure.

M: Is it good or bad to keep pigs outside?

All: It is bad.

M: How is it bad?

C7: They eat feces…

C4: …and they come to feed from our pots and plates.

M: What are the benefits of roaming pigs?

*Silence*

C2: Nothing.

C3: There is nothing beneficial because they eat rubbish and when they come back home, they feed from out pots, so they bring diseases.

*Some children laugh*

M: So, there is no any benefit?

C7, 8: Nothing, there is nothing.

M: How do you keep pigs in your family?

C1: We keep them in an enclosure.

M: What time do you do that?

C1: In the morning.

C6: We take them into an enclosure in the afternoon.

M: What time do they come out?

C2: They do not come out; we feed them from the enclosure.

M: What about you sister (C6), when you take the pigs in an enclosure in the afternoon, what time do they come out?

C6: In the morning.

M: Who can add? C4?

C4: We enclose them in the afternoon and we will let them out the next afternoon.

M: Others? Yes C4?

C4: We feed them in the morning and the afternoon.

M: How do you identify your pigs? How do you know that these pigs are your pigs?

C4: We know since they are ours.

C1: We prick them into the ear as an identity.

C9: We even cut the tail

*Other children laugh*

C1: Others would put a plus sign on the ear, also they are castrated.

M: Even female ones?

C7: No, only male ones. Others know through colors.

C8: Others just count them.

C4: They count and know the number of pigs they have.

C2: (amid murmuring from other children and opening of the door) Others just close them up and feed from there.

M: What are the benefits of rearing pigs?

C1: Because they eat feces.

C5: They are difficult to feed.

M: What are the dangers of rearing pigs? What problems would be encountered in rearing pigs? There are problems, now we want to know, which problems are those?

C7: Because they eat feces.

M: Others?

C5: They are troublesome

M: How are they troublesome?

C7: Sometimes, the feed for the pigs would finish (C2 agrees).

M: What problems are found in the rearing of pigs?

*Silence*

M: How do you know that your pigs are sick or that one of our pigs is sick? How do you know that the pigs are sick?

C7: They do not eat or drink water.

C1: They get thin and they do not eat

*C3 murmurs in agreement*

M: Others?

C2: We know because they get thin.

C4: Some will face down

*Silence*

M: What do you feed the pigs? What food do you give the pigs?

C1: We put medicine in the feed.

M: Which medicine?

C5: Sisal

M: Which sisal, any sisal or what? Which one?

C5: Any

M: What type of food do you give pigs?

C1: Peelings of grains (maize bran) and *nshima*

Nshima = Zambia’s staple food made from maize meal

C7: Pumpkins

C4: Water

C7: Even some rotten foods thrown away (*laughs*).

C2: Maize

M: Others? Who feeds them?

C2: Elderly people

C3: (*Reactively*) anyone can do it, even parents.

C4: Adults (in addition to the previous speaker)

M: Are there people who have been given the responsibility of feeding pigs?

*Silence*

M: Are there no people who have been given the responsibility of feeding pigs?

C3: Others do that.

M: What about in your home?

C4: They didn’t choose any one specifically.

C3: School going children feed when they are home and those non-school goers feed them when the school goers are not around.

*Silence*

M: Do pigs find food on their own?

C2, 3: Yes they do.

M: Like which food?

C7: Feces (*she laughs and others join into laughter*).

C2: Pumpkins

M: Where do they find feces?

C3, C4: In the bush

C3, C7: Sometimes, feces are just in the bushes close to their village especially where there are no toilets.

M: Do you take part in caring pigs?

*Silence, they all smile.*

M: Do you take part in rearing pigs?

C3: Yes

M: How?

C3, 4: Feeding them

C6: Taking/leading them to the enclosure (*all laugh*).

M: Apart from family members, who else takes part in caring for pigs?

C3: Nobody

C7: Pigs survive on their own. Sometimes, when a child has defecated, adults will call the pig to come and eat the feces (*she laughs and others join in laughter*).

C6: (adding)...the child will run away and the pig comes to eat

*Laughter*

M: There is no one else who helps?

C2: There are just people who will allow your pigs to feed with theirs.

C4: Some pigs will just feed from a household even without being invited

C1: Sometimes, you put some food and they will come to eat.

M: Who decides when to kill a pig or not to in your households?

C8: My father.

C7: It’s my father.

C6: My father

C4: It is my father

C3: The owner of the pig even if it’s a child is the one who should say that lets kill this pig.

C2: The owner of the pigs is supposed to authorize.

M: Those of you who say ‘my father’ why your father?

C7: Because he is the oldest in the home. Since he is the head of the family (*she laughs and others join her in laughter*).

M: Does it happen that every person owns pig individually in a home, that this pig is for my father, these are for my mother and that these are mine?

C3: Yes, it happens.

C3: Parents give pigs to children

*C7 laughs*

M: My sister can give you a pig but they will not consult you when they want to sell or kill it.

C4: Even if they give you, they do not consult you when they want to sell of kill it.

C2: You just find out that your pig is nowhere to be seen.

C4: When you ask them, they say that they did not know (*C8 laughs as a sign of agreement*)

C3: There are times when I can buy a pig for myself and parents will not have a say on it. I will buy a small pig and say that this pig is mine and no one will have a say.

M: Who feeds the pigs?

C3: All of us

C4: All of us

M: How many times do we feed them?

C1: Three times.

C4: Three times a day

M: What times are these?

C1: (with gestures) in the morning, afternoon and evening.

M: Which foods do you give them?

C1, C2, C3, C4: Peels off grains.

C3: *Nshima*

C7: Yes *nshima*

C6: Just get peels of grains and water and mix it.

M: Do you think that there any problem with pigs eating feces?

All: Yes

C3: A very big problem

M: Which problem is this?

C2: When they eat feces, they come back to feed from our pots.

C4: …and you kill the same pig and	eat which means, that we eat feces

*Laughter*

C3: To add on that, someone may go to the toilet and from there, they do not wash their hands, the same thing make people get cholera.

M: What are the benefits of pigs eating feces?

All: There is no benefit.

C4: They eat feces and there is nothing beneficial about that.

*Laughter*

M: Do we eat pork regularly?

All: Yes

C3: It is very important

They all laugh and all speak and making so much noise that no one’s contribution is clear.

M: Are there no specific days when pork is to be eaten very much?

All: No, no

M: What about during initiation festivals?

C3: Yes but even during funerals.

M: C6, when do we often eat pork?

C6: In the dry season.

M: Where does this pork come from, which people eat during public events?

C7: It comes from pigs.

C3: It comes from other villages, even from the *boma* (central district)

C4: Like from *Misoro*. In fact, they come from	every village.

Misoro = example of a village where pork comes from

C3: (*interrupts*) From *Nyamphande* (village) and everywhere else, even in our village.

M: Do they test the pig or pork before killing or after killing it?

All: No.

C3: They don’t.

M: Why do you think they don’t?

C3: It’s just based on trust; they believe that it is good.

C7: As long as it has fat

C3: On health, pork is evident. You can tell from the appearance of the meat.

M: Do they throw away the carcass of thin pigs or sick pigs?

C3: Some people eat but some don’t, they throw it away in the bush.

C4: Some people when they find that a pig has died on its own, they take it round the village selling while others throw away.

M: If pork was to be tested, what would be your comments?

C2: It would be good, since it brings diseases.

M: Others, on the inspection of pigs and pork?

C1: It is good to prevent diseases.

C7: We do not need to eat if it is found with an infection.

M: Would it be a good thing to test pork?

All: Yes.

C3: Very much, very much

Laughter

M: How do we eat our meat or how it is cooked?

C2: We remove the fur, cut it, wash it…

M: Who can continue?

C7: We wash it with warm water.

M: How do we cook? Some of you are adults and I think you already started cooking, how do you do it?

*All children laugh.*

C4: We add a little water…

C3: We fry it, add cooking oil

Others laugh, because C3 is a boy and it is unexpected for a boy to know more about the kitchen

C4: Some add cooking oil and tomato.

C7: No, not that way.

M: How do they cook it?

C7: We start by frying it, that’s when we add tomato, oil and a bit of water for gravy.

M: Others?

C6: We put in the pot to boil a bit, then we fry it to make it brown, then we add tomato and that’s all.

C4: We even add curry powder.

*Some children laugh*

M: What about roasting it because there is a difference between roasting and stewing?

C1: They just wash it and roast.

C7: There is a lot of dirty in the way pork is roasted.

Children speak at the same time causing noise.

M: Please be loud and clear, we need to get you. Can one person speak?

C1: Pork is roasted in two ways, one way is very bad but the other one is ok.

M: How do your parents cook pork?

C7: They wash it first and put it in the pot, then fry it.

M: C4, what do you say?

C4: You fry it, and then add tomatoes.

C1: Some add tomato, onion and salt. That’s all.

M: Do we know why your parents cook in that way?

C3: So that it tastes delicious and tasty

*C7 and C8 smile*

M: Do they roast it?

C4: Some do it (*while others say no*).

M: Do we know *nsembe*?

All: Yes

M: How do they look?

C4: They are small and white in color.

M passes pictures of pork laden with numerous cysts.

M: What comes to your mind when you see *nsembe*, C7?

C7: It suggests that the pig is infected.

M: Have you ever eaten pork with *nsembe*?

C4: No, we don’t, we do not buy it.

M: Why not eating?

C4: Because it has diseases.

M: Others, have you eaten such before?

All: No

M: Why?

C4: Because it looked like it had an infection.

M: When they slaughtered such a pig at your home, what did they do?

C4: They sold it.

M: Did people buy the infected pork?

All: Yes they did.

M: Do you all have toilets at home?

All: Yes (most of them)

M: All of us have toilets?

C3: We do not have at our home

While others say that they have

M: Do we all enter into the toilets that we have and use it?

All: Yes (*while some children laugh*)

M: All of us including our parents?

All: Yes

M: Why

C1: For fear that pigs will eat the feces

C2: To avoid dirty, and some parents even have two toilets

*Friends laugh*

M: For those of us who say that we do not have toilets, where do we go?

C3: We go to the bush

*Laughter*

M: Why do you go in the bush?

C3: It is not always that one will feel like going to the toilet only when at home, sometimes even as you go to the bush to fetch firewood. When that happens, you defecate in the bush

M: What do those people who do not have toilets do when they are pressed?

All: They go to the bush and relive themselves.

C3, 4: Some go to the bush; others go in their neighbors’ toilets.

M: Now let’s talk about bathing, Do we all bath? Do you manage to bath?

All: Yes, we do.

M: What time of the day do you bath?

C7: In the evening

C2: Some in the morning, while some it is in the afternoon

C3: With me, I bath twice. In the morning and in the afternoon (they all laugh)

C7: With me, I bath once.

M: Where do you draw the water from, the water you use to bath?

All: From the well

C3: From the borehole.

C2: (*Laughs*) A bole hole and a well are the same

*C4 laughs*

M: What time or when do you wash your hands?

C4: Every time after using the toilet

C7: You need to wash with soap

*C7 and C8 laugh*

C4: When one is about to eat *nshima*, they wash their hands.

M: Why do you wash your hands?

C3: To wash the germs from the hands (they all laugh and murmur) you need to need to wash the hands before cooking or touching the food.

M: Have you ever seen worms?

All: Yes

M: How do they look like?

C3: With me, I have seen them before.

Moderator passes pictures of the tapeworm in human stool for the respondents to see.

M: Have we seen those before? The whitish things?

All: Yes

M: What do you think those things are?

All: They are worms.

R: Have we seen those in vomit etc?

All: Yes

M: Where do you think they come from?

C4: They just start

C3: They can come from the soil.

M: Are babies born with those?

All: No

M: Where do they come from?

C7: If someone eats soil, then they get worms.

C3: Soil. The most important is that it comes from the soil.

M: Yes, my brother (*C5*)

C5: They come from the soil.

M: Why do children sometimes complain of abdominal pain? Why do they feel some pain in the stomach?

C4: It’s because of worms

C3: It is worms in most cases

M: How do you know that someone has worms?

C3: Over eating. The person would be eating too much. He can even finish a bucket of *nshima* alone

*All laughs*

M: What else?

C3: The most important thing is over eating. You find that the person eats unnecessarily.

C4: One would eat at home but also at the neighbor

M: Can these diseases be treated?

All: Yes

M: C6?

C6: Yes we can prevent them

C4: You can even start diarrhea

M: Do people get cured

All: Yes

M: How do they get cured?

C3: Yes they do get cured. You take medication. I have once recovered from such. It weakens you as if you will die. But you find out that the following day, you recover.

C1: (*Interrupts*) – the stomach is clean.

M: Are these diseases scary.

All: Yes

C3: Yes, because you can die

M: Are there worse diseases than these?

All: Yes

M: Like which ones?

C3: AIDS

C4: Diarrhea

C2: Diarrhea, malnutrition

M: What about minor diseases than these?

C2: A common cold

C5: Headache

C2: TB

C1: TB

M: Do you think that there people in your village with this disease?

A: Yes.

C3: We may not just know, but they are there. Even me, I may have it. If am tested, you might find them in me.

M: If one has worms, is it very dangerous?

All: Yes

M: How?

C3: Over eating. A person will develop a pot belly (the others laugh)

M: What is the major problem with a person having worms?

C3: The major problem is over eating. The person may eat for an hour but still wants to eat because they still fee hungry

*C8 laughs*

C1: When he/she eats, they would complain that they feel something moving in the stomach.

M: Others?

*Silence*

M: We have seen cysts, not so?

All: Yes

M: We know *nsembe*, we have seen *nsembe* before, what is the relationship between *nsembe* and people with epilepsy, is there any relationship between *nsembe* and people with epilepsy?

C1: Epilepsy is also a disease

M: Where do you think epilepsy comes from?

C3: It comes from pigs.

C8: Some are born with epilepsy.

C7: Some, it starts while they are young and they grow up with it.

C6: Yes (*In agreement with C7*)

C2: Others are bewitched.

M: What are the symptoms of epilepsy?

C2: Madness.

C7: Abnormality behaviour from time to time

C3: Fitting.

C4: Fitting

C1: Fitting

C7: Unfortunately, one may get seizures and fall on the water (pond or dam).

M: Do you know people in your village that suffer from this disease?

All: Yes

C3: There is one at our home.

C5: Even at our home.

M: How does this illness come about or how did it come to the people you say have the disease at home?

C1: By eating pork.

C3: Sometimes, people just bewitch them.

C8: Some people get cured after treatment, others don’t.

C7: Others do not get cured, they die with the disease.

M: Is this disease treatable?

C3: Some are but others are not.

C7: Some people have the disease forever.

M: Why is not treatable?

C3: It means that the person was bewitched.

C7: If you have been bewitched it is not treatable.

M: For those that we treat, how do we treat them?

C6: By looking for medicine.

C7: Herbs.

M: Others

C4: Herbs from the traditional healers.

M: Do we know people who know those herbs?

All: Yes

M: Do people get cured?

All: Yes

M: Is it dangerous to have this illness?

All: Yes

C4: You can fall into the pond, drown and die.

C7: (continues)… if you are unfortunate.

M: Others

C4: You can fall on the fire

M: Could there be worse diseases then epilepsy? Which ones?

C3: It is only AIDS

M: Do you think that there are smaller diseases compared to this disease? What are they?

C7: Asthma

C3: TB, Malaria, TB

C1: Stomach upsets

M: Do we have people with epilepsy in our village?

All: Yes

M: Is this illness a big problem

All: Very much.

C3: You can die.

C1: You can fit in the night and wake up with bruises

*C7 laughs*

M: What is your perception about someone with epilepsy?

C7: We think that such a person may die anytime

M: Others?

C1: One may get seizures even when one is in a tree and hence would fall and injure themselves or even die.

M: What if government asked us to keep our pigs in enclosures, would that be a problem or would we manage and heed to such a rule?

C4: Others can’t manage

C7: Others wouldn’t do that, they wouldn’t manage. They are not used to keeping their pigs in enclosures…

M: Why?

C3: If it comes as government regulation, yes it can work out: everyone who does not follow the regulation will be arrested (*gestures hand cuffing*)

C2: It would be difficult because people do not have adequate feed for the pigs. At certain times of the year people don’t even have food to feed themselves.

M: Others, what do we think about this?

C3: Other people depend on feces as food for the pigs (*other agree with him*) and hence would not house them.

M: What if there was a government regulation about compulsory toilet use for the people?

C7: There wouldn’t be order in the toilet

C3: Toilets would be dug but if someone is already in the bush, they would opt to defecate in the bush.

C7: People are used to using the bush

*Laughter*

C2: Even at school where there are toilets pupils still go to the bush.

C3: That would be possible but the problem is when someone is already in the bush. They would defecate in the bush in open space.

M: What about if the regulation was about boiling pork very much, would such a regulation be welcome?

C3: Others would not follow because they are used to way they prepare it.

C7: Others may take it as time wasting

S*he laughs and others laugh too*

M: What about everyone washing vegetables before cooking?

C7: Others would not be washing (*she smiles*)

M: Why?

C7: The need to be fast

C1: Others are just used not to wash

C4: Others say that the garden is far from the village, so the vegetables are clean

M: What about a regulation about inspecting a pig before slaughter?

C8: Others would not be doing that

M: Why?

C2: It might be wasting money

C7: Others fear losses in case it has infections

C2: If the pig is infected, they may be need for refunds

M: Others, what do we think about this?

C4: It must be inspected before slaughter, which is very important.

M: What about bathing, if we want to reduce these diseases of worms and epilepsy and where everyone was to be asked to bath every day. Can people like it or not?

C7: Others cannot manage.

M: Why?

C3: They can’t like it because they are not used to bathing every day.

C2: They even have stretches of dirt on their bodies and clothes.

M: What if government brought medicines for worms?

All talk and it is not clear

M: Can people refuse to be treated for worms?

C4: Some can resist the medicine because people here are suspicious of anything.

C3: If they are brought to the clinic here, people can take them.

M: What if we said that all pigs should be taken for vaccinations?

C2: Some can agree but others can’t.

C7: Others cannot do that.

M: For us who cannot do it, why wouldn’t we do that?

C2: There is a suspicion that vaccinations are not safe and that pigs can die and people wouldn’t want that.

M: My brother, C5, say something.

C5: Some do not want.

M: Others on the vaccination of pigs?

C3: Others can and others can’t.

M: Why, for those of us who can’t take them?

C2: Others fear that their pigs would be found with illnesses and maybe asked to kill them.

C3: It is easy to know that is sick just from the outlook, just even the way it is walking, so there is no need to vaccinate.

M: I believe, we have reached the end of our discussion. We have discussed the benefits and disadvantages rearing pigs, about toilets, how we care for our pigs, about cleanliness, the need to wash hands after visiting the toilet, and washing hands, before eating, we have also discussed epilepsy and its prevention; we have discussed worms, about vaccinations and the need to have enclosures for our pigs. Also about medication for those people that might have worms. Are there questions?

C2: I have a question. Is it possible that those pigs (non traditional) also have infections?

M: Is it possible that pigs from the diary farm can also have *nsembe*? Can we answer this question?

C3: We can’t answer this question because we do not have those kinds of pigs here.

M: I am grateful that you came and thank you for your answers and an interesting discussion. Thank you.

**THE END**
