# Supplementary material for: Why Latrines Are Not Used: Communities’ Perceptions and Practices Regarding Latrines in a Taenia solium Endemic Rural Area in Eastern Zambia
Source: PLoS Negl Trop Dis. 2015 Mar 4;9(3):e0003570. doi: 10.1371/journal.pntd.0003570 (PMC4352092; doi:10.1371/journal.pntd.0003570)
Supplement: S1 Dataset — (ZIP) [file pntd.0003570.s001.zip › FGD transcriptions_Zambia-2010/Sikalinda_David Mwanza/Sikalinda-men-evans edited.doc]

**Title : Focus Group MEN**

**Date :** 01/08/10

**Site :** Kakiwa Rural Health Center

**Village :** SIKALINDA	**Location :** Petauke district, Eastern Province, Zambia

**Duration (total time) :	62 min**	**- Start :**	14:32 am	**- End :** 15:35 pm

**Participants:** 8 men

**Informed consent:** signed and available

**Moderator :** Mr Emmanuel Mwanza	**Recorder :** Dr Andrew Phiri

**Transcription/translation :** David Mwanza

**Abreviations : M : moderator, R : recorder, I: intervener, C : children, W : women, M : men**

**Disposition (from the left of the Moderator):**

**M**

M1

M2

M3

M4

M5

M6

**R**

M7

M8

Age composition:

M2: the oldest

M8: the youngest

M7: young

All are young husband

Typology:

M7: shy

M4: rambler, funny

(M5) M6: dominant

M1: quiet

M3: expert

**Comments about participants :**

**Group dynamics :**

**Text:**

*Introduction*:

M: As my friend has said, you are welcome. First of all, let us know each other. But to begin with, me, My name is Emmanuel Mwanza. I am a volunteer here at Kakwiya. What about you?

Members introduce themselves

*Discussion*:

M: Fine, you are welcome. Be free as you take part in the discussion. We called you that we can talk about how you take care of your pigs. Firstly, I will ask you a question. What are the benefits of rearing pigs? The importance of pigs, what is it?

M7: They are helpful during funerals and weddings.

M2: To raise money if you do not have.

M3: They help in raising money for school fees for our children.

M: Others, pigs, how do they help us?

M5: When you sell pork, the prices for pigs are high; you make more money when you sell a pig than when you sell a goat.

M4: Yes, the money you can raise after selling pork can be more than that of selling a small cow.

M: What else?

M6: The importance of pigs, pigs are very helpful. If I have five circumcised male pigs, it means that I will be helped. For harvesting, we hire people to help us and we pay them a pig. Also during wedding, I can hire people to come and help me with weeding. This is what I find as the goodness of rearing pigs. So rearing pigs is very good, it is very helpful in many ways according to how we have explained.

M: Now, let us come to the second part. Is there anything bad on the part of pigs? Are there any disadvantages of rearing pigs?

M8: The badness of pigs, if we do not have toilets, pigs will follow you as you go to the bush to defaecate. They also have nsembe. When you kill it, you will find the diseases. That is another badness.

M: What else, who can add on what he has said on the badness of pigs?

M4: They eat a lot regardless of time. They can eat overnight, even if it is raining.

M: What else my brother, who can add (*M5*)?

M5: They follow as you go to defecate whether they see you or not. Even if you leave it, you will see it in no time following you. Sometimes, it will appear as soon as you start defecating. They are very bad.

M6: When a pig is very hungry, they are very impatient; they make you stand up before you finish defecating. They will push you and put you aside. They will feed on feces and later come to eat from your plates. They bring diseases.

M: What else?

M4: It you are not careful, pigs can eat a human being. It doesn’t care about anything. It doesn’t choose.

M: What else?

*Silence*

M: How helpful are pigs?

M7: They help us in many ways. Sometimes, I do not have money; I will sell and find money.

M: What else?

M3: They are helpful during funerals. When people come for a funeral, they need to eat and we slaughter pigs for meat.

M: Okay, so you mean that pigs are able to mourn the dead?

M3: No, pigs will be killed and be eaten as food by the people who come for the funeral.

M2: And the kind of meat is very delicious, such that when you eat it, even the mind will settle down.

*All laugh*.

M5: Nsembe are very nice. When one is eating, the person feels very nice. They mix with fat, in fact, pork is just irresistible, even if you wanted to refuse, you end up saying that I will only eat for today, tomorrow I will not eat. Today I will eat since I have found it here.

*All laugh*.

M: Okay, now the threats, what are the threats of pigs? How would you explain them? The threats that are found in pigs, would there be any?

M4: Threats are there but on our own without the doctor, we cannot know. We say that the meat is nice.

M: What else? What are the threats of pigs on the lives of the people?

M6: When I eat, they will bring diseases. They bring diseases such as worms, diarrhea. Mainly, in nsenga, we just say “minyolo”.

minyolo = meaning worms

M5: Just to add on, nsembe lead to diseases. Although we said that they are nice, when you eat them, they bring diseases.

M2: The other thing is that if it has too much fat, it can cause diarrhea.

M8: The other thing is that it causes disease, as for me, I don’t have AIDS, now if the pig has eaten the stool of an AIDS patient and later, I feed from the cup where the pig will feed from and leave AIDS. Wouldn’t I be found with HIV that I did not have at first?

M: How? So, you are saying that you can contract the HIV virus through pigs?

M8: If a pig eats faeces of an AIDS patient and they come to feed from your plates, and then the other person without AIDS come o feed from that plate without washing it, he will contract the disease.

M: Have you heard the question? He is saying that, we have confirmed that a person in this village has HIV, and that person has diarrhoea, now the pig goes to eat those feces and come to eat from the plates that the other person without HIV will feed from, wouldn’t one contract the disease? Is that possible?

The majority says yes.

M3: As for me, I refuse, I cannot contract AIDS in that way.

M: The pig eats the stool and finishes everything, you can’t contract the disease?

M3: I can’t contract it. HIV is found in blood and not in human stool. AIDS comes through blood contact. The only threat is that pigs can only bring diarrhea, worms and others. But it is not possible that a person can contract the disease through a pig eating the stool of an HIV patient. No, it is not possible but just other diseases.

M: You mean you can’t contract it even if the person who defecated has diarrhoea and there is some blood in the stool, you can’t still contract the disease you mentioned?

M5: You have to contract it, because that blood will have come from the body of a person.

*M4, M6 and M8 laugh.*

M: What else? Who can make additions on this matter?

M6: You can contract HIV, because it will not only get to plates and pots, pigs can even transfer the virus to mealie meal. When you put mealie meal in the sun, the pig will just come directly. Maybe, my wife will not even sieve the mealie meal which means that maybe, I can contract the disease.

M: How do we keep our pigs? You said that we have pigs where we come from. Now, how do we keep our pigs where you come from? Do we leave them to roam around?

M6: To be honest, in the village, pigs just roam. It is rare that we keep them in enclosures. But at the farm, during this season, they are allowed to roam but not in the rain season, only during the rainy season we keep them tied or in enclosures.

M: What else?

M3: We close them up sometimes, and sometimes, we do not.

M7: What we need to do is to keep our pigs in enclosures and feed them from there. Pigs are very important.

M2: Yes, that is good. We need to keep pigs in enclosures. That way, we can prevent some diseases.

M: What about ourselves, how do we keep our own pigs? Do we have enclosures?

M6: For me, my pigs are at the farm and I keep them into enclosures.

M3: We sometimes keep in enclosures but there are moments when we let them free to look for food on their own.

*All laugh.*

M: What type of food?

M3: It is feces.

M6: In addition, in the rainy season, it is difficult to find food even for ourselves, so we let the pigs free so that they can look for their food. It is very difficult to maintain cleanliness.

M4: Pigs defecate along the pathways and they cause a lot of dirty. The children will walk along those paths bear footed. When the pigs come to the village, they continue defecating and when it rains, the village looks very dirty, such that when the children are playing, they are likely to contract several diseases.

M5: I wonder, pigs eat our faeces yet we are their masters. I don’t know what they think we are.

M7: They can eat a live person.

M: They can eat a live person?

M7: Yes, because the faeces come from you as a person, which means that if a pig eats a faeces, they can eat the person who defecated. They eat your faeces; this means that they eat the person who defecated.

*All laugh*

M: What is wrong with pigs eating faeces?

M6: The problem is like we said, we can suffer from diseases. That’s why, where we can, we need to take care of the pigs, but where we can’t afford, we can allow them to roam around to look for food but they will bring diseases.

M: How do you identify your pigs? Because they are many, how do you know that this pig is mine?

M7: Pigs can roam far and wide, they can even go the end of Kakwiya village but they do not forget where they would have come from. They will go back at night after the moon has shined and they will come back at dawn, but the owner will think that the pig slept at home.

M4: Sometimes, they lose direction. It can end up being part of someone else’s pigs. They rebel and start looking like a wild animal. It will change the appearance to an extent where even the owner will have forgotten it. The teeth will protrude and look different. It may start going into other people’s fields, they will kill it, sometimes, if you go with dogs to hunt it, it may kill all the dogs that you have.

M5: The question is how we identify our pigs.

M3: What we do is to prick the ear and that is how I know that that this pig is mine.

M5: Yes we prick the ear.

M6: Sometimes, we cut the tail a bit.

*M8 laughs*

M: What problems do we face in rearing pigs?

M5: Feeding is the major problem. We do not have enough food and it is expensive. When you buy, it finishes in no time.

M: What else? Who can add on the point that the brother mentioned.

M3: In the rainy season, when we have a shortage of food, we forget about our pigs, so what will happen is that we eat all that we find. So they go to eat from other peoples fields and they rebel and look for their own food.

M: What else? Who can add on that?

M6: Pigs bring diseases. They roam and they will spread disease. In the rain season there will even be mosquitoes and they spread malaria. That is what I have seen to be the problem. The biggest problem is food, for us to manage more especially in the rain season.

M: Are there other people or relatives that help you in rearing your pigs? You have pigs, are there people who help out I rearing pigs?

M4: They are there but they are few. The people who think like that, they would bring the feed but at the end of the year, they will ask for one pig as payment. Because pigs would want food, goats would want food, chickens would want food, so food becomes a problem.

M: Others? Are there people who help in rearing pigs?

M6: To be honest, they are not there. We do not help each other and this problem cannot end. When we reach at that point, we depend on ourselves, because we have a lot of livestock.

M: How do you know that your pig is sick? Since you said that you may have a lot of livestock, how would you know that my pig is sick, what would make you know that the pig is sick?

M6: It is easy. A lot of people know my pigs, so if it is sick, they will see it and they will come to tell me. The other thing is that it fails to walk. My neighbor will tell me that the pig is there and it looks sick. Sometimes, it may walk and come home, it dies and I will eat it, and again I will get diseases.

M: Others? Who can add on?

M5: Normally, they face down. Those who are experienced will know that the pig is sick. Sometimes, it will refuse to eat food.

M3: Also, if it refuses to eat and they would not follow someone to the bush.

M4: In fact, there are months when pigs normally die and these people who sell pork know the time when there are such things. Sometimes, the outbreak will affect the pigs but sometimes, it will bypass the pigs.

M6: These people who sell pork also bring diseases. An outbreak can even occur and pigs can die in numbers.

M: How many times do you feed your pigs?

M1: Twice, in the morning and in the afternoon.

M: Is that possible without problems?

All: Yes

M5: It is not possible. Pigs eat a lot and two times a day is not enough

M: Yes my brother (M6)

M6: Pigs should be fed thrice: In the morning, afternoon and in the evening. Although this depends on the how busy you are as the owner.

M3: Three times is good. If you have money, you need to add salt to the feed. When they eat such food, they will not go very far even when you go the field; you will find them at home.

M: Do most people manage in the village to feed the pigs three times a day?

M2: No, they don’t.

Others agree.

M7: They feed on the remains from our pots. They will get the remains from the pot where they cooked nshima and give it to the pigs and they say the pigs are satisfied.

M: Now if pigs are not fed, what happens to them?

All: They will be moving about a lot and they will be going into other people’s fields to look for food.

M: Who feeds the pigs? In the family, who normally feeds the pigs? Who has the role of feeding the pigs?

M7: Anyone who remembers can get the food and feed the pigs. You do not need to employ someone to feed the pigs.

M1: Most of the times, the women are the ones who feed the pigs because they are always at home.

M5: Yes, that is the answer, women feed the pigs since they are always at home and they know when the pigs come from the bush.

M: What about the children? Don’t they have the responsibility of feeding the pigs?

M5: Children do not have the interest but they do so with encouragement from the parents. The parents will tell the children to prepare the feed for the pigs.

M4: Sometimes, the children are told to take care of particular animals. So, responsibilities to take care of certain animals are shared among the children. This means that if goats are outside, the child responsible for goats will know that he will be punished. The animals will even know the person that cares for them and will be able to identify him when he calls them.

M6: Just to add on, if I feed my pigs they will even know me. So, when I call them for food, they will come. So when it comes to feeding the pigs, I personally do it. The children can just help.

M7: For me, I do it personally at the farm but children can also do it.

M: Who is the decision maker over what should happen to pigs?

M4: It is the men, but you will find situations when women will also make such decisions, but mainly, the key is the man.

M: It is the men?

*All nod their heads in agreement.*

M: When do people mostly eat pork? The time when we often eat pork, when do we often eat pork?

M3: The pork meat normally, it is in the dry season after harvesting, also during Christmas and New Year’s Day. Mostly, it is during this season.

M: Others, when do you eat pork mostly?

M7: Yes, it is mostly after harvesting. It is this same season not in the rain season because when it is in the rain season, everyone is busy with farming.

M1: We eat at any time. We do not wait until I kill my own animal, sometimes, even when other people have killed it, I will also buy or take a share and I will eat pork.

M: When an animal has been killed, let’s say a pig, what do you look out for when you want to buy pork? When pork is being sold maybe in a bucket, what really are you interested in, the things you look out for in pork.

M5: *Nsembe*

M2: Fat

M: Yes my brother (*M5*) why nsembe?

*All laugh*.

M5: It is important because nsembe suggest that the pig is sick. Nsembe show if the pig is sick or not. So, the person who wants to kill a pig will have to use a wire to open the mouth and if there are whitish things under the tongue, you know that it is sick.

M4: Those people who buy pigs for resell usually check out for nsembe before they buy. Nsembe usually start from the mouth and even in the eyes. So, it is easy to know that this pig is sick.

M: Why do we mind about fat?

M4: It tastes nice with a lot of fat.

*All laugh*.

M2: Actually, you can add salt more and more and it is nice and it is really good.

*All laugh.*

M: Others?

M3: If it has a lot of fat, the fat makes you get fade up with the meat so easily. You even get satisfied early because the fat will make you lose your desire for meat.

M4: And again, fat will make you forget about all the bad things that pigs do. As you are eating, you enjoy the meat and forget that pigs eat stool.

M6: Yes, that is the truth; fat actually makes you forget about all the bad things that pigs do. Even if you saw a pig eating faeces and now it is killed, you forget all about what you saw when the meat is in the plate.

*All laugh*.

M7: Yes, when you taste it, then you start salivating.

*All laugh*.

M4: When you are roasting it, it can even put out the fire. Sometimes, you just say, give children, give children. It is nice. It is cooking, you even decide to take it out of the fire for fear that it will melt.

*All laugh.*

M: Fine, fine, is a pig tested before slaughter?

All: No

M4: It is not tested.

M: Why is the meat not tested?

M7: We do not need to wait for testing. It does not even come to mind that we need to test it.

M5: The problem is that veterinary doctors do not come here to make sure that it is tested. In fact, they want us to pay for the services which we cannot afford because, as we have already said, we are poor.

M: How do you prepare the meat? When a pig is killed, in all honesty, how is pork prepared in our villages?

M6: When people have killed a pig and I want to buy, I do not need to roast it but take it home so that it is boiled very well and be fried. Roasting meat is not good. It has to be boiled. It has to be fried to an extent where I should just tell my wife, that my wife, stop frying it, it will melt and it will finish. When you taste it, you say that, yes, this is relish.

*All laugh and smile.*

M4: We need to boil and burn all the fat.

*All laugh.*

M3: There is a difference when pork is roasted and when it is boiled. Pork must be boiled so that it is cooked well, more especially if they add onions, oh; I need to even eat even the residue.

M7. When women boil it and add onions, it really tastes delicious.

*All laugh.*

M: What is wrong with roasted meat? Is there any problem with roasting pork?

All: yes

M: Which ones?

M1: It doesn’t get ready. We just eat.

M2: It doesn’t get ready and there is no cleanliness. Someone will come, take the steak on the palm, add salt, eat from there but they will touch it without washing their hands. They will even salivate

M7: It gives us diseases. It does not cook, because the raw meat has diseases and when it is boiled well, and then the diseases will bun. The fire will still be raw inside.

M3: And the people just put the meat anyhow and you see a lot of fries coming landing on the meat. We do not even know where the fries come from.

M2: They come from the same place (toilet).

M6: In most cases, as we have already said, if I have HIV, HIV can also be transmitted if the meat was touched by an HIV patient who had a sore and the meat is eaten by someone else. The problem comes from us the people.

M: Have you ever seen nsembe?

All: Yes

M: How do they look?

M7: They look whitish

M: What is wrong with nsembe? What problem do nsembe have?

M4: They do not get cooked. They burst in the mouth as we chew. They burst in the mouth, on fire, they will also burst.

M7: Yes, they do not get cooked even when you boil. They burst in the mouth as you chew.

M: Is it possible to find the treatment for nsembe?

M4: No, the problem is that nsembe do not get burnt or cooked. They burst in the mouth. If they would be cooked, we would say that it gets ready.

M: Would you be happy if pigs were tested before slaughter?

All: yes

M: Would there be problems?

M6: Yes, it depends on the reason why you want to slaughter the pig. If it is for business, you can take it for testing. Now, if it is for the funeral, you my not have the time to start taking it for testing. I mean, what you would take people for at a funeral taking a pig for testing and making people wait.

A picture passes so that the discussants can see the cysts.

M: Do you eat the meat when you find that it has nsembe?

Some say they do and others say they don’t at the same time.

M6: For me, they eat, if it is at funeral, you eat, what can you do?

M: You people, a very fat pig is killed but you find that there are nsembe, what do you do? Do you throw it away?

M1: Me I can’t.

Others say: they can eat the meat.

M8: What is needed is that people should be coming to tell us what we can do if the pig has nsembe, or else, the problem may not end.

M: Do we know worms?

All: yes

M: How do they look?

M7: They look like strings.

M passes pictures for all to see.

M4: Yes, they are found in the intestines for goats.

M2: Even for pigs.

M: Those are worms (pointing at the pictures).

All: Yes

M: What are the threats of worms?

M2: They cause malnutrition

M: How do you know that someone has worms?

M4: If it is a child, they develop a potbelly and they eat a lot.

M2: They eat a lot as if they are pigs.

M5: Again, there will be sounds from the stomach.

M: Have we ever seen epileptic people?

All: Yes

M: What is the source of epilepsy? According to your thinking, where does epilepsy come from?

M4. We don’t know. We think that it comes as a result of being bewitched. We just hear that he was bewitched while others say they just started fitting. Now for us to know exactly where the diseases come from, we do not know.

M6: The disease of epilepsy, it comes in many ways, firstly, it comes as a result of cold, sometimes, and it is because of high body temperature due to malaria.

M4: Someone will get the disease, and when they start taking medication from the clinic, they will recover, but there are people who will have epilepsy and no matter how much you try medication, they will still continue fitting.

M7: Also due to anaemia someone can start fitting.

M: Is there cure for epilepsy?

M4: Yes, doctors can cure it if it comes naturally but if it is because of witchcraft, they cannot cure it. If someone is anaemic, take them to the clinic and the Doctors will treat.

M: What are the advantages of washing vegetables?

M6: Vegetables should be washed. When it rains, there is a lot of dirt that remains on the vegetable leaves. A lot of insects including snails leave a lot of dirty there. The snail may leave some mucus and that should be washed out.

M8: There are germs on vegetables, so they should be washed so that these can come out since they are diseases.

M: What about personal hygiene? How is this important on the daily wellbeing of an individual or indeed the household?

M2: We need to bath and that way, because when we bath, we can prevent diseases.

M8: If one doesn’t bath, the poles on the skin close and diseases can easily come.

M: If government introduced a law that all pigs should be kept in enclosures, would there be any problem?

All: No, there wouldn’t be any problem.

M: What about mandatory testing of pigs before slaughter?

M4: That would be good as long as the veterinary doctors would be coming here to test.

M: Is it good for us to be using toilets?

All: Yes

M: How about washing of hands, do people manage to wash their hands after using the toilet before they touch anything else?

All: Yes

M6: Some manage but some do not. There are people who would go to the toilet and without washing their hands, they handle food and yet they have come from the bush or the toilet. Like that there is no hygiene.

M7: There are also situations where you are on a trip and you go aside to answer the call of nature. After doing that, you can not wash your hands because there is no water there and when you buy bananas, you just eat.

*All laugh.*

M: Would we be happy if the cure for epilepsy, worms and vaccines for pigs was found?

All: Yes

M5: That would be very good according to how our lives would be with regard to how these diseases would come. It can help us prevent some of the diseases that afflict us as in our villages.

M: Fine, now, as we conclude, I wish to thank you very much. We have discussed the advantages and the disadvantages of rearing pigs, how they eat faeces and bring us diseases, about cleanliness and the consequences of not being hygienic, we have also talked about how we can prepare and clean our relish (pork and vegetables), and what would come up if we do not take heed. We have also discussed possible testing and vaccination of our pigs. At this point, I wish t allow for questions if there are any.

M4: The question that I have as for me is that, what type of diseases do the things like the ones we have seen on the picture bring to people?

R: For today, we wanted to hear from you, later we will have a meeting at your village where we will educate you. We didn’t want to tell you for fear of you going back to the village and start saying that, the problem is this and that, this and that.

M: In other words, because this is like we have put a load on your back, what I can say is that this is the first meeting and we will call for another one in future so that we can all come to chart the way forward. Is that okay?

All: Yes

M6: Now, since we need to pay the veterinary officers when they come to vaccinate our livestock even as poor as we are, how would we say government is helping us?

**THE END**
