# Supplementary material for: Why Latrines Are Not Used: Communities’ Perceptions and Practices Regarding Latrines in a Taenia solium Endemic Rural Area in Eastern Zambia
Source: PLoS Negl Trop Dis. 2015 Mar 4;9(3):e0003570. doi: 10.1371/journal.pntd.0003570 (PMC4352092; doi:10.1371/journal.pntd.0003570)
Supplement: S1 Dataset — (ZIP) [file pntd.0003570.s001.zip › FGD transcriptions_Zambia-2010/Sikalinda_David Mwanza/WOMEN - SIKALINDA-edited-kem real.doc]

**WOMEN-SIKALINDA VILLAGE**

M: How are you mothers? You are all welcome. We called you so that we talk to each other about pigs. Firstly, we need to know each other. My name is Mrs Ruth Chiwa.

*Participants introduce themselves.*

M: Do we know these?

*Moderator points at the camerawoman and also the observer.*

All: No

R: This one is Severine. She comes from Belgium. She is our visitor. She came to Lusaka and we work with her at the University. Her job is to work with this machine (camera). So we tell her that these women have said this. We told her that nsenga women are clever and talk a lot so she has come to prove it by herself. The children were talking a lot, we could just say, “but these children talk a lot”. That’s why we have brought her here.

Dr Mwape: What is the meaning of Sikalinda?

A few voices: It is a village.

Dr Mwape: The word Sikalinda, what does it mean?

W6: It means that we have settled.

Dr Mwape: Okey.

M: Be free and open. There is nothing like a correct answer or a wrong answer. All the answers that you will give will be correct since those are your opinions. People differ in the way we think. This is my first question, are pigs important in our villages?

W4: Yes, it helps with paying of school fees, but also during misfortunes.

M: What misfortunes?

W4 and W1: Funerals, funerals need food.

W1: We also get helped during initiation ceremonies; we slaughter a pig so that people can be helped at such ceremonies.

W7: Also for weddings, when weeds are too much in the field, you slaughter one and ask people to weed and you give them the pork.

W2: If you have a bad harvest, we exchange pigs with maize so that your children can have food.

W6: On marriage ceremonies, we slaughter pigs for food so that people can eat.

M: What is the goodness if pigs?

W5: Pigs are very good.

M: How are they good? What is the goodness of pigs?

W7: Pork tastes nice, it’s delicious. That’s why it is nice. Since you said we should be free, we are answering you according to what we think.

M: Yes, you should be free and answer freely without holding back anything.

W6: Its good because it’s usually enough for a number of people...

W5: …very unlike goat meat.

W5, 6: Pork is good, even a small amount of the meat may be enough for the whole family.

M: Others? What are is the goodness of pigs?

W6: It has fat, even if you do not have cooking oil, it still cooks well.

M: Others?

W7: What we have already said is what we think is the goodness of pigs.

M: What are the disadvantages of eating pork?

*Most women mumble saying “It may have diseases”.*

W1: The badness, firstly, it may have nsembe but we still eat it. Instead of throwing it away, we eat it.

W5: The other problem is that since some people don’t have toilets, it eats faeces. Even when children haven’t carefully disposed off faeces, it will go and eat. Sometimes, you find that a pig would give birth and eat its own offspring.

W6: Even at the graveyard, if you didn’t bury well especially human pre mature babies, a pig can go and eat the body. Pigs do not choose. They eat young ones chickens, goats, just anything.

W3: They do not choose.

W6: Those are the problems we find in pigs.

M: What are the uses of pigs?

W4: They are useful since they help us.

W5: It helps. They are important. They help when we have problems. Yes, when we sell it according to the problem that we have.

M: What are the threats of pigs? The dangers of pigs.

W7: You need to teach us about the dangers, that’s why we are here so that where we do not know, you can teach us, that’s when we shall know.

W4: The dangers are that even when you keep them, you know that have dangers since they eat children’s faeces such that even when eating you remember what it was eating, and that’s why some of us don’t even eat.

W1: Another danger is that a lot of people don’t eat pork because pigs are dirty and they have diseases, they eat faeces and came to drink from our buckets and kitchen utensils since we just keep them on the ground. They eat faeces and come direct to feed from our cups. And when a child picks up the cup, they’ll just drink from it without knowing what the pig would have left in the cup. That way the pig brings diseases and that’s a danger.

M: Others?

W6: Another danger is that when we eat the meat, it may cause epilepsy among our children. And there is no medicine where you can say I will cure my pigs.

W4: Another thing is that when there is a disease outbreak like African swine fever, they die all at once during an outbreak. Yet, when they die, instead of throwing them away we eat them.

W3: It becomes relish.

M: Do you keep other livestock apart from pigs? For you as women?

All: Yes we do.

M: Like which ones?

W2, 3, 4, 5; Speak together saying goats, chickens etc.

W6: Yes, goats, chickens, sheep, etc.

W1: We keep, like for me I have ducks, sheep, goats and if you ask why these, it’s because they do not die easily and do not have a lot of infections or disease outbreaks. They also do not transmit diseases to humans. Chickens are a little difficult because they also have diseases but are easy to rear. And furthermore, chickens are not as expensive to buy as pigs. Just like a goat, it’s not as expensive as a pig. Even when the goat is very big, the one selling will only ask for very little money. But for a pig even a very small one would be very expensive since its meat is quite a lot, that’s why we keep other livestock animals.

M: Who can add on?

W7: Just like they have said, chickens just like pigs also die a lot during disease out breaks. Goats are better. Goats do not die any how. But they give us problems in rearing them. Also the prices for goats are very low. So we end up keeping pigs but they also die during disease out breaks.

W4: You keep, they die, and you buy others and keep again.

W6: They are helpful. We cannot stop keeping them.

M: Do we all here keep pigs?

W7: No, some of us do not keep, they finished a long time ago.

W4: Some of us did but they died recently.

M: So, all of us do not have pigs?

W4: Some have.

M: How many have pigs?

W7: There are two that have.

*W1, 2, 3, 4, 7, raise their hands acknowledging that they have pigs.*

W7: I thought there are only two who have.

*Laughter.*

W4: They haven’t yet finished from outbreaks.

M: What is our role in rearing pigs?

W4: Our role?

M: Yes.

W4: They help us in a lot of ways. We need to feed them so that they eat.

M: No, no, your role in the keeping of pigs?

W4: Our role is giving them food so that they eat; you give in the morning, again in the afternoon. If you are not busy you give them maize bran three times in day..

W1: Another role is making enclosures where they can sleep.

M: Others?

W3: It is the same.

M: Who else assists us in the keeping of pigs?

W1: There is no one.

W4: We are assisted by the building of pens and feeding them, enclosing them. In the evening you call them and close the pen and open the enclosure in the morning so they go out because if we keep them in-doors for a long time, they develop polio in their legs..

M: Who helps making enclosures?

All: Husbands make the enclosures.

M: And if you do not have a husband?

W3: We hire someone (others agree with her).

W1: Even the neighbors can help you, for instance they can give you the feed and you later give them a pig in return.

M: How about relatives, do relatives help?

All: Yes

M: How do they help?

W4, 1: They help by feeding and leading them into the enclosure and also building the enclosures..

M: Who has the authority to decide when to sell or slaughter a pig?

W3: It’s me, the owner, the person who keeps and feeds them..

W5: It is the husband

W3: She has said that what if you do not have a husband, it is yourself.

W5: It is the husband who decides that you sell or not, it is the husband.

M: Don’t you as women have the power to decide that lest sell apig?

W4: Yes, you can but the final voice should come from the husband

(W5 agrees with her). If you make decisions without consulting your husband, you can not sleep the whole night.

M: What about children, don’t they also make decisions about which pig should be killed?

W7: No, how can a child have authority? A child is being kept at his mother’s house and needs direction, how can he or she have authority to make such decisions?

W4: A child can suggest but it’s not usually taken seriously.

W7: Yes, it cannot be taken seriously.

M: Who feeds the pig?

W4: It’s the children; we ask them that you feed the pigs, when I, the mother is busy.

W7: Am cooking, you feed the pigs.

M: What kind of feed do you give them?

Selected voices Maize bran, nsembe, pumpkins.

M: That’s all?

All: Yes

M: How many times do you feed them in a day?

W8: Three times.

W2: Others just twice.

W8: If you have a lot of feed you give them three times, if not then just twice.

*There is a knock at the door.*

M: What are these times?

W4: Morning, afternoon and in the evening when you call them so that they get into the enclosure for the night.

M: Is there any goodness in pigs eating faeces? What do you think?

*Silence*.

M: Pigs eating human faeces, is there any goodness?

W7: Human faces?

M: What do we think about the goodness of that?

All: No, there is no goodness in that, only badness.

W7: There is just badness in that. We eat it because of problems. That’s why some people vomit immediately they put the meat in the ir mouths, the moment they remember that the pigs were eating feces.

M: What is the other disadvantage of pigs eating faeces?

W5, 6: When they eat feces, they come back with faces on their mouth and drink from our buckets.

W7: …they come and drink water from our utensils.

W3: Even when you have put mealie meal in the sun to dry, they just come and eat from there.

M: I thought that since there is very little feed for the pigs, so the human feaces is like feed for the pigs, not so?.

W1: That is not goodness. If I They inconvenience the process of defecating because they are impatient, I may not finish defecating, I need to move to another place where I can continue defecating.

M: Now, pigs eat faeces and people eat pigs. What do you think about this?

W7: Like I said it at the beginning. This is where you should teach us because we came to learn about pigs, this is where you should teach us.

W1: We need to keep pigs indoors and provide them with enough feed, and then they won’t come out. And all of us in the villages we should dig pit latrines so that all the older children should be using the toilets. For the younger children, their parents should make sure that they clean the area when their children have defecated.

M: Do you think pigs’ health is at risk as they eat faeces?

W4, 5, 6,7: Yes, they also get infections as they eat those things.

W4: It will eat faeces with diseases and hence also get those diseases.

M: How about on our health, pigs eating faeces?

W4, 6: It’s not good at all.

W6: We need to keep the pigs in enclosures so they just roam about. The problem is that our pigs easily die when we keep them indoors. It’s up to you to build an enclosure for the pigs, or the pigs would have died a long time ago.

W7: I think when there are enclosures there will be no pigs.

W8: They need to be allowed to roam so the enclosure needs therefore to be big enough so they can still roam.

M: Pig meat, do we eat it very frequently?

W6: Very much.

W1: That our relish.

W3: At shokas, a lot.

W1: We eat every time. It is the commonest relish. We are able to eat even three times a week. Goat meat is scarce. What is common is pork(other women agree with her).

W7: But if you don’t have money can you even eat it three times a week?

W8: Like right now we have outbreaks so there is pork meat everywhere.

M: Are there times/seasons when we eat pork more often?

All: Yes

M: Like which times?

W1: Like this same season, from August to October (other women agree wit her)

W4: Because right now all you find is shokas everwhere.

M: And other times?

W4: In the rainy season, because we go to the fields, we eat a lot of shokas.

W8: In the rainy season we slaughter and use the meat for preparation of the fields, weeding, buying seeds, and fertilizers.

*W7 gets up and walks out.*

M: And during celebrations?

All: We slaughter a lot.

W6: We slaughter pigs during celebrations like weddings and also at funerals …

M: These same pigs that we slaughter at these times, where do they come from?

W5: Some pigs are reserved to be slaughtered on Christmas day and also new year’s day.

M: When someone has come selling pork, all of us women go to have a look at the meat, not so?

All: We call one another.

M: What do we normally look for to decide whether we should not buy or buy the pork?

*W7 comes back*

W5: We check for the size of the pieces and the appearance of the meat.

W6: We see if has fat.

W8: If it’s dull looking, then no. But if it has a lot of fat then we buy.

W6: They will even tell you are late this one has no fat…

W8: …then I will just buy vegetables, what can I do.

M: Sister (W2) say something, what do you consider when you want to buy pork?

W2: I need to look at it, whether it has fat or not, if it doesn’t have fat, I just say that this is bad meat, I will buy a different one.

M: Are pigs tested before slaughter?

All: No, they are not tested for anything.

W5: We just judge by looking at the meat.

M: Why?

W5: There is no one who can come to test the pigs.

W1: In our villages there is no one to examine or test the livestock that we keep. If there was someone to test, we would be doing it. We wish there was someone who could come to test the pigs. At leat they would be telling us whether to slaughter or not.

M: Would we be happy if pigs were tested before slaughter?

All: Yes, (all speak at the same time and results into noise).

W3: Like today, they slaughtered early in the morning in the village, without testing.

M: You don’t know who can examine?

All: No we don’t know.

W6: We just know those that examine people.

M: Talking about how we prepare the meat in readiness to eat, how do we cook our pork?

W8: By frying it.

M: You just get it and fry it?

W7: You have to wash it first.

W8: We wash it, add salt and put it on fire.

M: Others

W4: After frying, we add tomato and start boiling...

W7: (interrupts) …let me also explain how I cook it. When I put the pieces on fire, I add a lot of water so that it really boils. After frying, I also add some more water to boil. That’s better; pork should be boiled for along time, that’s when it will taste nice. Not just frying it for a short time, it does not test nice that way.

M: Others? How do we cook the pork? Since the way we cook as individuals differs

W1: If we want to eat it urgently, we just roast it; you just roast and eat it. (Amid laughter from others). That’s what we do you just roast it and eat.

W7: Yes we need to be truthful.

W4: We just roast it. It is an express way.

M: Why do we roast the meat? Why do we prepare the meat like that?

W6: No reason, we just do that so we can eat the meat.

W1: We do it when we are late for something, if you are going somewhere you’ll need to hurry or else the vehicle will leave you. We think it’s faster that way.

W7: Even when I am not behind time. Roasting is good, it is faster. I would be cooking nshima and I am roasting the meat, that way, the meal will be ready in no time.

W6: The roasted one is very tasty. Even at the neighbours, if they roast you’ll even know that our neighbours are eating pork.

M: Sometimes, it is to impress our husbands, not so?

Women agree amid laugh).

W3: If you give him the boiled one he can even chance you saying it’s too over done.

M: How about nsembe? Do we know nsembe?

All: Yes, we know it.

M: Do see them?

All: Yes.

M: When you see them, what does it mean to you?

W1, 4: We know that those nsembe are diseases but we do not have a choice.

W8: You can even see in the meat, that this one has the nsembe.

W4: They are bad.

*The women speak all at once on the badness of cysts resulting in mumbling noises which is not clear.*

M: (Passes picture of pork laden with cysts for women to see the cysts). So when you see them what do you think?

W1: We know that this is a disease but what to do with such meat is what we do not know.

W6: Others say you cook the meat and the nsembe will be no more.

W1: That snot true they don’t go away by cooking, they don’t get cooked

W2: You’ll have to add a lot of water and boil for a long time and that way the meat won’t be nice.

M: Have we ever eaten meat like that?

W7: Meat with nsembe? As for me I have never eaten it, it’s not very nice in the mouth and you can vomit.

M: And others have we already eaten meat like that befeore?

W4: As for me I do not even pork.

W3: I eat but I have never eaten one with nsembe.

W1: How can you eat when you can even see the cysts, it’s very nauseas.

M: Do we have toilets at our households in our village?

W7: Most of us have toilets. Only those who recently come to the village do not have toilets. But most of us have the toilets.

M: Do we consistently use them?

All: Yes

M: All the time?

All: Yes all the time.

W7: Even if it is at night. I use the toilet. I will go with a torch and I will go to post my letters (meaning defecating). (Other women laugh).

M: How about those you are saying do not have toilets?

All: They go to the bush.

*All women speak and there is noise.*

W5: Those without toilets go to the bush.

W7: Some go to the neighbours’ toilet if the neighbour has. Surely, the bush, is there anything nice in going to the bush?

W5: And it’s the hot season and there is no bush since the grass has dried or has been burnt.

W8: And if the neighbour has no toilet where do they go?

W6: Obviously to the bush.

M: What are the disadvantages of not having toilets?

W1, 6, 7: There is no respect.

W8: It doesn’t give respect if you have visitors, then you cant show them the toilet since it’s not there..

W4: When visitors come, it is embarrassing to tell them that you do not have a toilet. But if you have you will feel free and show them.

W1: Another disadvantage of not having a toilet is when you have diarrhoea, it is embarrassing and difficult if you do not have a toilet and you can go to the neighbours worse still it may be too late to get to the bush. When you are in the bush you find that a pig is even waiting for you to finish, I can’t manage that.

W2: Worse if you leave in the middle of the village, you’ll mess yourself up before you even get to the bush.

W6: Toilets give respect.

W5: You can even take your time when you are in the toilet other than when you are in the bush. You do not need to check here and there when you are in the toilet like you do in the bush to check if there is someone seeing you.

M: Concerning cleanliness, who cleans children?

W5: It is us the mothers.

W6: A woman washes the clothes, reminds the children to wash their hands after visiting the toilet. Even when a child defecates you clean up the child and take the faecse to the toilet.

M: Is it important to bath?

W4: Yes, it is, ac child can have all sorts of illnesses and it prevents malaria.

W1: Every person should wash for example people should wash their faces every day morning. If one doesn’t do that’s you see that the eyes are not ok. So even us adults should wash our faces or else you’ll have some eye infections. And if you do not bath, the sweat pores will be blocked and by the time you wash such a child you’ll make the condition worse.W5: Even the body feels heavy if you do not bath.

W7: A child looks better after bathing.

M: Whose responsibility is it to ensure that children are clean?

All: It’s us the mothers.

M: There is no one who helps?

W4: Men also help by buying soap, lotion and other things. But the major person is the woman who should bath the child. That way the husbands help.

W1: But if you are busy, the husband can also bath a child.

M: What about children bathing fellow children?

W6: They do. Peers bath each other and older children will bath younger ones if you give instructions to do so and also teach them how to bath their young siblings.

M: What do we teach children about washing hands?

W6: We teach them to wash hands after using the toilet before they go back to play.

W7: Let me answer, when we give them food, the children should water each other. They should not wash hands in the same bucket using the same water, so that the other children eventually eat their friend’s dirt. As for me, I stopped a long time ago allowing children washing their hands using the same water. We should not use the same water to wash the hands of all the children.

M: What do the others say?

W6: You use a cup so that each child washes their hands separately and the water does not get very dirty.

M: Others, what do we do, we are talking about what we do at our households.

W7: What I have said is what I do in my household.

W4: That’s what we do, but we allow all the children to wash from one dish and if the water gets dirty it’s thrown away and fresh water put for all to wash.

W2: Some of us have older children who are responsible for themselves. We stopped bathing them.

W8: We need to pour water on each other’s hands when washing hands.

M: Why do we do that?

W4: We were taught that others may have sores in their hands and hence can transmit diseases to others. That is what we learnt to avoid illnesses being transmitted to others.

*Other women agree with her and there is some noise.*

W1: Some do not cut their nails and nails hold a lot of dirt and if they wash they leave the dirt there. So they shouldn’t wash using the same water.

M: Have we ever seen worms?

All: Yes we have.

M: Where have we seen them?

W4: With me, I saw them in Lusaka at my sisters’ home in one of her children. We wre told that the child had expelled a worm. They look like small snakes.

M: Others?

W5: I had worms, so I saw them.

W7: Yes we have to tell the truth. But we came to learn, how come we are not learning?

M: (Passes picture of tapeworms for the women to see). Have you seen those before?

All: No

M: In someone’s faces, have you seen them?

All: No, we haven’t.

M: They look like snake’ skin, have you seen them

W7: Are all these discharged from the stomach as well with faeces?

M: Yes they are.

*Most women laugh softly.*

*W7 repeats her question.*

M: Yes.

W1: They are on someone’s faeces right there.

W7: This is how we learn.

R: Segments, they look like the skin of snakes, have you seen them before? (Describing the tapeworm)

M: Have you seen them before?

All: No.

M: we just see other roundworms?

All: yes.

W7: Now, this, what is this? Is it a worm? When it is small, it is like this? And when it grows it will be like this? (speaks while looking at the picture)

M: Yes, but that is in the initial stages.

W7: So when its small, it looks like this and when it grows does it become a big snake like worm?

W1: They grow later. It is just that we normally do not check the stool

after our children have defecated. We would be seeing them. How can we see them because after defacating we just get up, we do not check for anything, so how can we see them?

W7: Now, for an adult, how can you see them? If you use a toilet, thye faces go into the toilet?

W4: For children, it is easier. If there is a worm you can’t miss it.

W7: It comes out:

W4: Yes, I even saw a child running around with a live one coming out.

W5: Actually children defaecate live ones.

W1: Its even cold to touch.

W5: Like it’s from the fridge.

W6: They are scary.

W4: When the person I saw was given medicine, he discharged everything at the same time entangled together like a ball.

M: How do you know that someone has worms?

W4: The stomach bulges whether hungry or satisfied. The person will develop a potbelly. Even before they eat, they look like they have already eaten.

W8: They do not even grow well. They do not look nice.

W6: Sometimes, it is persistent stomach pains...

W7:…(interrupts) that’s when you take the child to the clinic and you see worms coming out. But there are signs when a child has worms.

W8: Because the body does not grow well.

W1: The body temperature also rises and there is also anaemia.

M: Where do we think worms come from?

W1: They say when you eat soil.

W5: And eating rape (a popular vegetable) and other foods.

W1: Yes, rape and other leafy foods, that’s what they tell us.

M: Do we think that this disease can be treated or prevented?

All: Yes

M: How can we treat this disease?

W7: Yes it can as long as you go to the clinic since medicines are plenty.

W6: This can come from you people the medical people.

W7: Since when we are given medicines, they all come out. How can treatment be unavailable? Think clearly.

M: How is prevention like for abdominal worms?

W4: We need to wash rape with warm water. We do not need to wash rape with cold water.

M: Others?

W5,6: We should not be eating soil, those that eat including children should stop. And we shouldn’t be eating rape everyday.

W1: The rape should be we’ll cooked not like they cook it in big cities hotels.

W5: Its even not nice if its not well cooked.

M: Others?

*Silence.*

M: Can this disease be cured?

All: Yes, it can be cured.

M: Have you witnessed it being cured?

*Silence*

M: Is this disease very dangerous?

All: Yes

M: How is it scary?

W2: People die.

W1: When they are a lot in your abdomen, the worms get the food that you eat and sack your blood. I have experienced that.

W5: I have also seen that, they were big and red in colour.

M: Are there diseases that are more deadly than this one?

All: Yes

W7: There are many diseases that are deadly.

M: Which ones would you consider more deadly than intestinal worms?

W4: More deadly disease?

M: Yes

W4: Apart from worms?

M: Yes.

W4: It is this same disease that doesn’t have a cure. It is HIV. It has no cure.

M: Others.

W7: Its AIDS.

M: What about minor diseases to this one?

W5: Headache, flu, coughs, eye problems.

W8: Ear problems.

M: Are there many people in our village that have intestinal worms?

W1: Yes, we see them by their signs; you will find that there are children who have malgrowth. You will find that a child that will be born after such children will grow up and surpass the one with worms. Malgrowth is a sign of a person having worms.

W5: Some have very still pot bellies.

W1: Regardless of how much medicine you give him/her, nothing will change.

W5: The hair will change.

W8: The cheeks will also change and become swollen.

M: Is it scary to have worms in the stomach?

All: Yes

W4: They kill

W5: They are fatal

M: In our daily lives as people, what do you know about us human beings having worms in our stomach?

W7: We think of going to the clinic. We go to the clinic, get some medicine and drink. That’s how you can get cured.

M: What problems are faced by people with worms in their stomachs in their daily lives?

W6: They lead to persistent stomach problems. Even when you are in marriage, they persist. Sometimes, you may even be pregnant, but you still have worms. You defecate and you find that you still have worms.

W4: They eat too much without getting satisfied.

M: Do we know epilepsy?

All: Yes

M: Where do we think this disease comes from?

W7: We just observe that someone fits.

W1: Some are born with epilepsy. Sometimes, they are bewitched.

W7: Here in the village, we always think that epilepsy comes from witches, now you should teach us about this disease. Every time someone has epilepsy, us villagers think that the child has been bewitched, now we want you to teach us.

M: What do others say?

W4: When a person has epilepsy, they would just freeze and release some foam from their mouth. After sometime, they regain their conscious. Epilepsy comes in many forms, that’s why people would say the person was bewitched because some people do not even release foam from their mouth. It would happen and people would say ‘he/she was fitting just here’.

M: What do you say my sister (W2).

W2: These things are very surprising. Some children would just be born and they start fitting. Some are inborn.

W6: We are told that when we are pregnant, we are advised to take immunization but we resist. Therefore, when a child is born, they will start fitting. We are the ones who contribute to this problem.

M: Where does epilepsy come from?

W7: It is genetic

M: Others, what do you think?

W4:This disease is very surprising that even newly born babies can fit. We think that it is inborn. How can a child fit when the child has just been born? So we think that the mother is the one that has transmitted it to the child.

M: Is it only children who suffer from epilepsy.

All: No

M: What do we think causes epilepsy in adults?

W4: We think in adults, it is because they are bewitched. They even die from it.

M: Don’t we think some meat causes epilepsy?

W1: It is pork. We suspect pork very much.

M: How do we know that someone has epilepsy?

W7: They behave abnormally.

W4: They are not normal.

W5: Insanity, you know that the wires in the brain are cut (they all laugh)

W4: Madness is evident, some wires would be missing in the their brain (they all laugh)

M: Do we see the wires?

ALL: No (they all laugh).

W4: They do not concentrate on anything.

W2: Paralysis to some parts of the body (some agree with her).

M: Do you know people with epilepsy?

All: Yes

R: In the village?

All: Yes

R: Where do you think they got this disease?

W1: Some say they were born with it. Others say it developed after they were already grown up.

W7: What we said earlier is what happens. We also think it is anaemia. That someone started taking the treatment for malaria late.

M: Do we think that this disease can be cured?

W1:On our own, we normally prefer herbs. When you start treatment early, some recover. But others die of epilepsy. It is like a family disease.

M: Is epilepsy curable?

W5, 7: Some do, others don’t.

W7: Others have it when they are young but stop when they grow up. So people would say, that that person used to fit but he/she doesn’t any more.

M: Where do these herbs come from?

W7: I don’t know. Other just look for herbs and the child will stop fiting.

W4: When they use herbs, they will ask the child not to eat pork and warthog meat.

W3: Others will say that you should not eat eggs.

W5: Others will say that do not eat from the pot used for cooking pork. Do not eat the eggs of a chicken.

W7: Others will simply ask the child not to eat the whole of the pig.

M: He/she should have his own pots?

W5: Yes, if they cook pork at the neighbour’s house, he should not eat. He should have his own plates and pots and should take care of those.

M: What if he is hungry?

R: Then, there is no eating?

W4: There won’t be eating. The child will have to know that I do not need to eat this and I do not need to eat that.

R: Who tells the child what pot to eat from?

W5: The parents, the people that stay with the child are the ones that will tell him that this food, you should not be eating, this food, you should not be eating.

M: Do you fear that you may contract this disease?

W4: Yes, they say that if the child produces a fart and the gas comes to you, you will contract the disease (all women laugh)

W5: Yes, you contract it and it will come to you.

W4: When the child is fiting, the fear is that when the child farts, you will get the disease.

W7: Now, if the child is yours…

W4: …you need to sit close to his head and not his buttocks at least to avoid the gas after he has produced the gas.

W5: Sometimes, it is difficult because you are the mother.

W7: But the truth is that, if you put your child on the laps, even if the child farts, the mother will not contract epilepsy if she was not meant to get it.

W3: I think witches just bewitche, I don’t think it is true that you can contract it from fart.

W7: If you were not meant to get epilepsy, you can not get it unless you already had the disease. Would you run away from your own child saying that the child may fart at you?

W4: That is what we hear from elderly people.

W7: They used to say like that. There have never been liers like those elderly people of our time. There were lies. When I grew up, we were told not to eat egss, no one would come and say here you are, eat this egg. They said that eggs are not to be eaten by the young ones.

W4: (While W7 is still speaking) they said if he farts at you, you will contract the disease.

W3: They also forbade those small fat rats.

W7: They said we should not eat them.

W2: What about those big fat rats (mfuko)?

W7: More especially those, we were not allowed to eat those ones.

W5: They used to be selfish; all they wanted was for the children not to eat certain nice things.

W7: Gluttony is what was the case. They were so selfish that they did not allow children to eat certain things. (all laugh)

W5: You could just eat gravy or soup!

*W7 faces down and laughs*.

W7: You see, we have forgotten that we are being video taped.

M: Now about this disease epilepsy, are there worse ones than this?

All: Yes

W5: Others are aged 15, 16…

M: I mean diseases, are there diseases that are worse than epilepsy?

W1: The greatest disease is HIV. That’s the disease that has no cure.

W5: It has no cure. That is the disease that has come.

W6: When someone is sick, we just say this is AIDS, we start doubting how long the person may live.

W5: We just say this is HIV, we do not know whether they will live any longer.

M: What about smaller diseases compared to these ones?

W5: Headache.

W3: Side pains

W5: Back ache.

M: Are there a lot of people in the village who suffer from epilepsy?

All: Yes.

M: What do we do with them?

W8: We just leave them, because it is not their wish.

W4: We leave them like that, because they will have tried all sorts of treatment even from herbalist and things can not work out, you just leave it like that. They will grow up with it and even have children, without recovering from epilepsy.

R: They can even marry?

W4: They cannot even get married; there is a man in the village...

W1: …some time ago, he used to fit once for a while but now, it is an everyday issue.

W4: He fits every day. Even eating is now a problem, he can’t eat nshima, he would vomit once he does that. So he only eats porridge.

M: Do we think that this disease is very dangerous?

*All women speak.*

W5: Yes, they are dangerous, they do not heal.

W3: Maybe the person may fall on the fire, on the water or on the pond.

M: What about in everyday life, if a person has epilepsy, what do we think of such a person? What do you think can happen?

W6: When a person has epilepsy, we just know that this person is no longer a normal human being, because any time he may fall on water and will die, anytime, he ay fall on fire, and he will die. So we just say God alone knows when the person will die.

M: others?

W4: Others will behave in such a way that when you are talking to them, they will suddenly shift their attention to something different and start talking about different things. So, you know as I have said that some wires will have gotten disconnected in the brain.

M: Now, if in the attempt to control these diseases of epilepsy and worms, there was a regulation maybe from Nyamphande (The area Chief) or the boma that we all have toilets both young and old, what problem would be there?

W4: No, there wouldn’t be a problem.

W5: Diseases would be prevented if you have toilets.

M: There wouldn’t be any problem?

W4: Problems cannot end. Cleanliness can’t be 100%

W7: Problems are many madam. Problems can not completely end. Complete cleanliness is only found in death. When they throw you, that’s when the problem may end, not when you are alive like this, it is a lie.

R: Any problem that would be there if we were to construct toilets?

W1: The problem is that, it not everyone that can adhere to cleanliness rules. Not all the people may know the importance of having toilets. That’s why if they can be taught or us who have come here go to teach them about this, they can build toilets without any problem. Without that, it is not possible.

M: What about a regulation that all pigs should be kept in an

enclosure? And that no pig should be roaming, we should be keeping them indoors.

W5: It would be very good. We would be happy that we have been protected from disease.

M: There wouldn’t be any problem?

W4: That is a sure way of preventing diseases. Because pigs eat from there and they come to feed from my kitchen utensils and they bring diseases. They would have been kept away by putting them in enclosures.

W7: Infact, that’s the best. Diseases would be prevented. Now what problem would be there? When they are in the fence, they will be eating from there, which will have meant that you have reduced the chances of contracting diseases.

M: What if the regulation was that when ever any one was supposed to cook pork, it should be boiled for a long time before eating, what problem would be there?

W6: There wouldn’t be a problem

W7: It depends on the individual. Others are used to boil for a few minutes. Even if you advise them, they will not listen. Because following such regulations is reducing the problem. Sometimes, the person may say that they will follow your advice, but when you are not there, they will do their own things. Some people don’t fear diseases (some women laugh).

*All women start talking at the same time creating noise.*

W1: We should have workshops on how to cook. That’s how everyone will learn. We need a place where women can meet like we have met here so that some women can demonstrate to others how they cook, they would explain the steps which they follow when cooking. In that way, some people will learn not just like this. We should be told to bring pots and mealie meal and explain the process, hours are passing, and this would be good.

M: What if every pig should be tested before slaughter?

W2: That would be very good. Sometimes, we eat pork even when we

know that the pig was sick. When ever we see pork on the plate, we do not take time, we just eat. So if they are tested, it would really be helpful.

W5: Diseases would be controlled. At least, our lives would be prolonged.

W7: We would appreciate. As I said earlier, it depends on the individual. Like we have learnt here, we would not complain and we can’t refuse. If the meat is tested and we are told to throw away, we would throw away, but if we are told to eat, we just eat.

M: There wouldn’t be any problem? Like people saying that this is our relish, why are they giving us regulations over our meat?

W5: It depends

W7: It depends on the person. Some people can’t change. But even if I hide, I will just hide from myself. It is my health that is at risk.

M: What if the regulation was about washing vegetables before cooking?

W5: Yes, that would be good.

W7: How can I cook vegetables as if I know where the person who brought it had touched? There is no way I can cook vegetables without washing. Those people who sell vegetables are sometimes dirty. I always need to wash the vegetables.

W2: If I get them from my own field… (did not finish the sentence).

W7: (interrupts W2) …if snails walked on vegetables, it is not difficult to see. How can one notwash the vegetables? (There is laughter).

W1: Depending on the circumstances, we do not wash vegetable. If

you are behind time, you cannot have time of washing vegetables. We normally knock of late from our fields.

W7: That is how we eat snails. It is not difficulty to see where snails had passed. The vegetables would be dirty. But if I collected myself, I may be sure that the vegetables are clean.

W6: We eat snails (she laughs) in that way without noticing.

W7: With me, I am particular with washing vegetables. That’s why, I

refuse to eat food if I see that a person cooking did not wash vegetables.

M: What about compulsory bathing, if bathing is made compulsory?

All: No, there wouldn’t be a problem.

W1: Any person is supposed to be clean. How can you claim to be

clean if you bath but eat dirty food? A person is supposed to be clean and make sure that the family too is also clean.

M: What is it that you call cleanliness?

W7: Clean food, the body, water, plates, sometimes, dirt can even be

seen on the hands. (Some women laugh). Everything, cleanliness is not only bathing.

M: Do you only bath when you see that they are dirty?

W7: No, whether I went to the field or not, I need to bath. It doesn’t matter what I was doing, I need to bath. A person does not get dirty by going to the field alone.

W4: You can be dirty even when you haven’t done any work. Dirt is dirt, we need to bath always.

W7: Even those who drive still get dirty. You will find that someone is from a car but he is still dirty. Being dirty does not only mean you went to the field. You will see dirt on the hands, by the neck. Someone should bath.

M: Would there be any problem all people with worms were be treated?

All: No, there would not be any problem

M: We can be very happy

W6: That would be very good because the majority of the people have worms. We have worms.

M: You don’t see any problem W8?

W8: No, there would be any problem.

M: Can we all accept?

W5: Yes, even now.

W6: We can

W3: No one can refuse.

W2: Those who can refuse can suffer on their own.

W7: That’s why I said that people differ. Offices always differ from one another.

W6: We can advise each other.

M: What about mandatory testing of pigs?

W6: It would be very good. I mean, pigs have a lot of diseases. There is need for sensitizing.

W3: As long as you teach us.

M: We have reached the end of our discussion (before she completes the idea)

R: Excuse me!

M: Ok

M: Now, if you are asked to pay for vaccination for your pigs, would you pay?

All: Yes, we can

R: How much can we pay?

W3: It’s you who can tell us.

W6: It depends on what you tell us

R: But can you pay?

All: Yes

W1: The only problem is that they ask us to take our pigs to the boma

(Central district) and that is difficult because it is very far.

W3: Infact, if you have many pigs, maybe 13 pigs, how can you take all of them to the boma. It would be better if Veterinary doctors come here to the village.

W7: You have to book a car, and booking a car is very expensive, it is as if you are going to buy life.

M: I believe, we have reached the end of our discussion. We have

discussed pigs, the advantages and disadvantages of rearing pigs, who feeds them, the advantages and disadvantages of toilets, the benefits of cleanliness, washing hands after using the toilet, also about worms, about nsembe, masese, epilepsy and finally, we discussed how we can control epilepsy and worms. About taking pigs for vaccination, everyone with worms should receive treatment. In case of any question, you can ask now.

R: Before that, I have a question. I may ask you mum (pointing towards W7), If someone has epilepsy, do you segregate them during eating, sleeping and others?

W7: You (W5) explain to him, about someone being segregated.

W5: That segregation is on the foods the person has been asked not to

eat. You do not segregate him just for the sake of segregating him. You do that after the person has been told not to eat certain foods.

W4: If this person finds the foods that he/she was asked not to eat, it means that he/she should be given different foods on separate plates. This is for good intentions. It is not done to hate the person or anyhow.

R: What if you use a pot to cook pork, can you use it the following day to cook beef and can this person eat?

W4: We can’t use the same pot to cook the beef meant to be eaten by the epileptic

person. The fat of pork may remain in the pot and cause problems to the person who is suffering from epilepsy.

Dr Mwape: There is someone who said that pigs have a lot of fat, do you follow fat?

*All laugh, W4 points at W7*

W7: Yes, it is fat, it is delicious and also that, it is much more than what it seems ( in terms of quantity).

Dr Mwape:	(laughs) Do nsembe look like fat?

W7: Is it all the pigs that have masese? There are few ones that have masese, some do not have.

Dr Mwape: Okey, now, if you kill a pig, a big pig but you find that it has masese, what do you do with it?

W6: It will be bought and will finish.

Dr Mwape: It would be bought?

All: yes

R: If you are asked to throw away pork with nsembe after it has been tested, would you accept?

All: Yes, we can

W5: We can, because it would be evident that the disease has been found.

R: Even if you want to sell so that you can raise school fees for the child after you have had a problem with fees, would you still accept to throw away?

W6: Yes, we can throw away. Because if I sell, the person that I will sell to will still test it and will find that the pork has an infection. So it is better to ask veterinary doctors to come and test to see if it is okay.

W1: Before, slaughtering a pig, a person gets a stick and uses it to open the mouth of the pig and see if there is nsembe, if there is, it has to be thrown away. When checked if it has nsembe, most people do not buy it, if they find that the pig has nsembe because it does not taste nice.

Dr Mwape: So, you have eaten such pork before, for you to know how it tastes.

W1: Yes

(Dr Mwape walks in front of the panel)

The End.
