# Supplementary material for: Why Latrines Are Not Used: Communities’ Perceptions and Practices Regarding Latrines in a Taenia solium Endemic Rural Area in Eastern Zambia
Source: PLoS Negl Trop Dis. 2015 Mar 4;9(3):e0003570. doi: 10.1371/journal.pntd.0003570 (PMC4352092; doi:10.1371/journal.pntd.0003570)
Supplement: S1 Dataset — (ZIP) [file pntd.0003570.s001.zip › FGD transcriptions_Zambia-2010/Wonzi_Dr Phiri/Children_Wonzi_30-07-10.doc]

**Title : Focus Group CHILDREN**

**Date : 3**0/07/10

**Site :** Kakiwa Rural Health Center

**Village :** WONZI	**Location :** Petauke district, Eastern Province, Zambia

**Duration (total time) :**	64 min	**- Start :**	10:18 am	**- End :** 11:22 am

**Participants:** 8 children

**Informed consent:** signed and available

**Moderator :** Ms Ruth Chiwa	**Recorder :** Dr Andrew Phiri

**Transcription/translation :** Dr Andrew Phiri

**Abreviations :**

**M : moderator, R : recorder, I: intervener, C : children, W : women, M : men**

**Disposition (from the left of the Moderator):**

**M**

C1

C2

C3

C4

C5

C6

C7

C8

**R**

Gender distribution:

**C3-C4-C6-C8**: females

C1-C2-C5-C7: males

Age composition:

C1: Oldest (about 17 years old)

C2: Youngest (about 10 years old)

Typology:

C7: Dominant

C1: Dominant/funny

C2, C3, C6: Quiet

C5: Expert; Dominant

**Comments about participants :**

**Group dynamics :**

**Text:**

Introductions of the mission and informed consent done

*Introduction*:

M: Good morning? How have you woken up?

All: We have woken up well

M: We welcome/receive you all here at Kakwiya. Why we have come here, I belief, has been explained a bit, not so?

All: Yes

M: Before we have gone inside our discussion we need to know each other, not so?

All: Yes

M: I will start by introducing myself

She proceeds to introduce herself and every child follows suit. The research team is introduced later.

M: I will request you to be free to speak. Is there anyone who scares us?

All: No

M: Is there anyone who scares us?

All: No one

M: You need to be free. This subject is not a difficult one. Everyone needs to speak and contribute. There should not be anyone remaining quiet. We need to educate each other, not so?

A few: Yes

M: When I have asked a question, you need to respond according to what you know, not so?

A few: yes

M: They also said they will take a video. You heard them say that, not so?

C7: Yes

M: There is no problem with such an arrangement because you also want to be seen in a video?

A few: Yes

*Discussion*:

M: Now, the main reason we have all come is to talk about pigs. Do we know what pigs are?

All: yes

M: We all know what pigs are?

All: Yes

M: And we also have them?

All: Yes

M: If indeed we have pigs, do you think it is necessary to have pigs in the village?

*Pause/silence*

C7: Yes

M: If yes indeed it is necessary to have pigs, what is the main reason of having pigs?

C7: We eat and obtain relish from pigs

M: We get relish from pigs?

All: Yes

M: Others?

*Pause*

M: Others who can add on? Do we just partake of relish or there are other things that we need pigs for?

C8: We sell pigs in order to get money

M: To find money!

A few: Yes

M: Others who can add on?

C1: During funerals or general difficulties pigs help to solve such general problems

M: Difficulties are many, not so?

A few: Yes

M: In what or with what kind of difficulties do we get helped?

C6: During funerals, pork is used as food for mourners to eat

M: So that people eat?

C6: Yes

C1: So that mourners do not leave the bereaved home hungry

M: So that people are not left hungry?

C1: Yes

M: Any other way?

*No response*

M: What do you think is the goodness of pigs?

*Pause/silence*

M: The goodness of pigs... the benefits of pigs?

C7: The goodness of pigs is that we eat it as relish

*C4 and C5 giggle*

M: Any other? We need not be shy; we should be children who are free....Any other things?

C7: Another thing is that we use sales of pigs to obtain funds for school fees

M: Getting money for school; any other thing?

*Pause/silence*

M: We need to be free...we need to be relaxed

C5: Yes

M: Is it scary? Is what we have been talking about very strange?

A few: No

M: They are not strange?

All: They are not

M: Ehe! So we all need to talk. Another thing....What is the goodness of pigs?

*Pause/silence*

M: What about the badness or dangers of pigs?

*Silence*

Moderator repeats question

C7: The badness of pigs is that they have diseases

C1: Indiscriminate eating habits, not caring what it eats

M: Not caring what it eats. What kind of things does it eat for us to conclude that it does not care what it eats?

C7: Eating faeces

*A few laugh softly*

C5, C1: Playing in mud

C1: Playing like that, uh...they do not look good

M: Which other bad thing?

C3: When a funeral of a young person has happened, pigs can exhume buried still-born babies from their shallow graves *(saying softly)*

M: Please, can you speak up? We did not hear what you said

C1: Speak up *(addressing C3)*

M: Please repeat what you just said?

C3: When a funeral of a young person has happened, pigs can exhume buried still-born babies from their shallow graves

M: Have you ever seen babies taken away from the graveyard?

C3 and a few others: No *(others just shake their heads)*

M: But you just hear about these things?

A few: Yes

C8: Another thing. When a baby has been born and is accidentally left outside alone, a pig is capable of taking the child and eating it

M: Others who can add on?

*Silence*

M: There are no untrue answers here, alright?

A few: Hmmmm

M: All the answers you are saying are true, not so?

All: Yes

M: What do we see as the benefits of pigs?

C7: The benefit of pigs is that when you sell pigs, we have some income. In that way, we are helped

C1: It helps us with relish at home. When there is no relish, we get that thing and slaughter it and obtain relish

M: What about the dangers of pigs, what are the dangers that are there with pigs?

C8: The dangers are that it has harmful germs

M: Harmful germs like which ones?

*Silence*

C7: Another thing; we also suffer from “flu” that comes from pigs

M: Pig tends to suffer from flu?

*C5 laughs*

C7: Yes

M: ooh... Anything else?

*Silence*

M: Let us not be shy, gentleman. Are you feeling shy of me?

*Pause*

M: Don’t feel shy with me. These ones over there *(indicating other researchers)* do not even understand what we are talking about, not so? They do not even know the *Nsenga* language. They only understand English. Therefore, we need to be free people. The only ones who understand each other are we here *(indicating the focus group),* not so?

A few: Hmmm

M: Uhummm

*Pause*

M: How do we keep pigs in the villages?

C3: Giving them maize bran

C7: Others put them in a kraal?

C8 interjects inaudibly?

M: What do others do?

C7: Others keep them confined in kraals, while some let them stay outside, free-roaming

M: Others keep them without kraals, others confine them in kraals?

A few: Hmmm

M: Others? Does it mean those that keep them in kraals, do the pigs remain confined or what?

C1: Others are confined, but for most pigs when they are released they eat indiscriminately. For example, when someone has left kitchen utensils outside they eat from them. That is why some put pigs in kraals

M: So those that let the pigs roam about, what do you think is the goodness of pigs wandering about?

*Silence and shuffling of feet*

M: The goodness of pigs to be wandering anywhere, going wherever they want; what do you think is the goodness?

*Silence*

M: What about the dangers or the badness of pigs wandering? ... C2? What about the dangers of pigs loitering; the dangers of pigs to be roaming about?

*Silence*

M: Do we keep pigs?

A few: Yes

M: When you keep pigs, you said others confine them in kraals?

All: hmmm

M: Others said the pigs just remain outside, isn’t that so?

A few: Hmm

M: So if they stay outside, what danger is there?

C5: They eat faeces

M: They eat faces, who else can add on?

C7: They eat anything, anyhow; as long it is pleasing to them they eat. Sometimes, they contract diseases

M: Anyone else who can add on?

*Silence*

M: What kind of faeces do they eat?

C8: That of humans

*Loud laughter*

C8: They eat human faeces

M: That of humans?

C8: Yes

M: Do they go in the toilets?

All: No, no

C8: But when you go in the bush to relieve yourself

M: We relieve ourselves in the bush?

All: Hmm

M: Ehhh?

A few: Hmm

C7: We relieve ourselves when we are travelling

M: Only when we are on a journey?

A few: Yes

M: When we are not on a journey we do not relieve ourselves in a bush?

A few: Yes

M: Do you keep pigs in your families?

A few: Hmm

M: Are they there?

C7: Others keep pigs, others do not

M: How do you identify that these pigs are your own?

C1: Others tag their ears; others know them by their colours, that yes this is mine

C7: Other pigs tend to be at home; when they go out of home, they tend to get back later in the day

M: What about you C2, how do you identify your pigs?

C2: Give it a name

M: Giving it a name?

C2: Yes

M: Such that when you call it, it comes?

C2: Yes

M: Who else may add on? How do you identify the pigs?

*Pause*

M: My sis *(referring to C3),* how do you identify your pigs?

C3: By piercing/notching their ears

M: Piercing their ears.....Because in *Wonzi* there are many pigs indeed

*Pause*

M: My sis *(referring to C4)*, how do you identify your pigs?

C4: By tying a string or cloth around the neck

M: Tying a cloth! Do you use any kind of cloth to tie?

C4: Hmm

M: Now suppose it dies, it can die as a result?

*Laughter*

C4: No, you just tie it loosely

M: Tying it loosely?

C4: Yes

M: My brother *(referring to C5),* how do you identify your pigs?

C5: Cutting their tails

*Laughter*

M: Cutting their tails!

M: What about you my sister *(addressing C6),* how do you identify your pigs?

C6: Tying them with a cloth, ear notching and placing a string through the ear

M: Who pierces or notches them?

C6: It is my father

M: It is dad. What about you my brother *(referring to C7),* how do you identify them?

C7: Pigs learn to know or get used to their owner’s home

M: My sister *(addressing C8)*, how do you identify your pigs?

C8: By the general appearance of the pig. Our pigs are white on their feet

M: You pierce their feet?

Words for white and piercing are similar in pronunciations

C8: No, they are white on their feet

M: They are white?

C8: Yes

M: So at rearing of pigs, what kinds of difficulties or problems are there?

*Pause*

M: What kind of difficulties are there in rearing pigs?

C1: There are no problems. When you get helped, difficulties end

M: We have taken pigs and reared them. We say these pigs are ours, not so?

C1 and a few others: Yes

M: When rearing pigs, there are difficulties...

C1: Yes

M: So what kind of difficulties do we meet in rearing pigs?

*Silence, while others fidget or fiddle*

M: Is the question hard or you have not understood it properly?

C1: We have not understood it

C8: We did not understood it well

M: I will give an example. For example, children at home; for child to grow there has to be some difficulties, not so?

C1: Yes

M: I believe you know that. There is getting unwell, that is a difficulty as well?

A few: Hmm

M: If a child is troublesome, naughty and creates fights. Aren’t those difficulties?

A few: Hmm

M: It is the same thing with pigs. In keeping pigs there are difficulties. So, I am asking, what kind of difficulties do we encounter?

C1: We get helped when money is lacking. We sell that thing to realise money. Like that difficulties end

There is a misconception and misunderstanding here because the “*mabvuto”* in *Nsenga* can mean difficulties, problems or a funeral

R: So what are those difficulties that would make you to sell pigs?

C1: If I do not have footwear, I can sell a pig to purchase some

M: Have we understood the question?

All: Yes

M: Can someone respond to it?

*Silence*

M: You are all feeling shy, aren’t you?

C7: Difficulties are that they like to eat indiscriminately; when they come from eating such things in the bush, they also eat from our kitchen utensils. Now when we use those utensils to eat food from, we also can contract diseases. Those are the difficulties or problems with pigs

M: Those are problems with pigs?

C7: Yes

C8: Another problem is that pigs have malaria

M: A pig has malaria?

C8: Yes

*Many of the children are restless*

M: Any others who can add on the difficulties?

*Silence*

M: So that malaria you are mentioning, can a person contract it from pigs?

*Pause*

C7: Yes

C8: Only those people that eat pork can get malaria

M: Only those that eat pork can contract malaria that comes from pigs?

C8: Yes

M: Have you seen people suffering from malaria contracted from pigs?

*Silence*

M: Do you see them?

C7: We have not seen them but we only hear that at such a place there is someone who is sick of it

M: Because of pigs?

C8, C7: Yes

M: How do we know that one among our pigs is unwell?

C1, C5: It is sheepish

C7: Where it has lain, it just lays around there doing nothing

C1: Then you know that this pig is sick

M: How do others know that pigs are sick?

C6: They shiver or tremble

M: They shiver?

C6: Yes

M: Others?

C7: The hairs remain raised

M: How do others know that this pig is sick?

*Pause*

M: What about feeding, what do we feed the pigs?

C8: Maize bran and pumpkins

M: Others? What do we feed the pigs?

C5: The “shoulders”!

M: The?

C5: “Shoulders”

M: “Shoulders”?

C5: Yes

M: What are “shoulders?”

C5: Those that come from sunflower when it has been squeezed or crushed

M: Those are the “shoulders”?....Is it English, perhaps?

C5 and a few colleagues just smile shyly

M: I have just heard it today, “shoulders”. What else do we give our pigs?

*Silence*

M: Who feeds the pigs? In your family, who feeds the pigs?

*Silence*

M: My sister *(addressing C3)* who in your family feeds the pigs?

She shyly does not respond

M: Hmm?

C3: At home or.....

M: At your home, in your family?

C8: Ourselves

M: You yourselves feed them? What about others, who else feeds them in your family? My brother *(addressing C7)*

C7: Myself, and in my absence my mother feeds them

M: My sister? (Addressing C6)

C6: Myself, and in my absence my younger siblings

M: What about you my brother? (Addressing C5)?

C5: We feed them properly

M: You feed them properly, but who feeds them?

*Silence*

M: Is there someone who feed them?

C5: Yes

M: Or they just eat on their own, wherever they wish to go?

C5: No. We just put food for them, and they eat on their own

M: What about you my sister? *(Addressing C4)*

C4: I feed them and in my absence, my younger sibling does it

M: What about you? *(Addressing C3)*

C3: When I am not around, my younger sibling feeds them

M: What about you my brother? *(Addressing C2)*

C2: When all of us have left home and my father has remained, he also feeds them

M: What about you C1?

C1: Just myself and my father. When we are both not at home, the pigs remain hungry like that. When we come back that’s when we feed them

*C4 laughs*

M: Why do only the two of you feed them?

C1: Others at home do not know how to feed the pigs

M: They do not know how to feed? Are you the only ones who know how to feed?

C1: No, What I mean concerning how to feed is that maybe they may give all of the maize bran and finish everything we have. But for us, we apportion the feed appropriately giving it in small portions in a day and the following day like that

M: Now in the case that you both have left and you have gone far from home and you stay there for many days, so the pigs also remain just like that?

C1: No, there is someone who we leave with instructions on what to feed

M: Don’t these pigs find their own food?

C7: They find their own food, but the kind of food they kind is a bad kind

M: Bad kind like which ones?

C5: Faeces

*Laughter*

C1: They eat different things such as earth worms, mud...

C7: Bugs and insects

M: Do you take part or play a role in pig keeping in your families?

*Silence and reluctance*

M: Pig keeping, in a family we keep pigs, eh?

A few: Yes

M: So, do you also take part?

C7: No

M: You never take any part?

C7: Yes

M: Why not?

*Silence*

R: Is feeding not part of keeping pigs?

A few: Yes

C5: It is keeping

R: So, you also take part in keeping?

C7: Yes

*Silence*

M: Apart from family members, those who keep pigs, are there some other people who assist you in keeping pigs?

A few: No

M: Why are there no others?

*Silence*

C7: Because the pigs do not stay at their own homes, they stay at our household. So when pigs get back to our homes, it us who collect feed to give them

M: So, keeping pigs is also getting feed and giving them?

A few: Yes

M: Is that keeping?

A few: Yes

C7: ...and also putting them in a kraal

M: What about who makes decisions such as today we should slaughter this pig? Who is it in a family?

C1, C7: It is parents

C1: It is our parents who decide and say “let us slaughter this one”

M: It is parents.....What about in your family, C2? Who makes a decision and says “let us slaughter this pig today?”

C2: Parents

M: What about you sister? *(Addressing C3)*

C3: It is parents

M: What about you? *(Addresses C4)*

C4: Parents

M: So, you children do not take any part?

A few: Hmmm

M: Why is that you do not take any part?

C1: We fear that if we as children say that we should slaughter a pig, may be our parents may be unhappy with it

C7: In fact, they may not agree that we slaughter it

C7, C1: But only them to decide.........

C1: That we should slaughter

C7: That is when we can take part after they have decided

C1: When such a decision is made even us feel good that today we may eat well... *(Smiling)*

M: We have already discussed who feeds pigs, not so?

All: Hmmmm

M: How many times are these pigs fed per day?

C4, C1: Three times

M: Which times?

C3: Morning, afternoon and evening

A few other kids (C2, C5, and C7) say the same thing at the same time

M: Do you manage?

A few: Yes

C1: Others do not manage to feed like that because the pigs can be gone even for three days.

M: Pigs?

C1: Without being seen. When it comes that is when it is given food

M: So when they have gone there is no following them?

C1: Others follow them.......

C7: But some cannot find them even if they followed them

M: Do the pigs move about a lot?

A few: Yes

C1: They roam about a lot

C7: If they are let loose, without confining them in kraal, they move about a lot

C1: They just play on their own

M: We talked about pigs eating faeces?

A few: Yes

M: What is the goodness of pigs eating faeces?

*Silence, a few look bewildered*

M: The goodness of eating faeces, what do you think yourselves?

*Silence*

M: Is the question not well understood?

C7: It is not well understood

C8: The goodness or badness?

C1: We heard that the goodness! That is why we do not know how to answer

*Laughter*

M: We said that pigs eat faeces, didn’t we? Now, we want to find out the goodness of pigs eating faeces, not so?

C7: Yes

M: Or why is it that pigs eat faeces?

C1: Not giving them feed. If they are given feed and confined in a kraal, they would be eating those foods that they are given. But when you let it loose, it follows the smell or goes in smelly places......

*Laughter*

M: Do you think pigs eating faeces is a good thing?

All: No, it is a very bad thing

M: It is not a good thing, why?

C1, C7: Because........ *(Saying it at the same time)*

C7: Faeces....

C1: ..... There are diseases

*Laughter*

C8: There are bad diseases

M: What kind of bad diseases are they?

C3: Cholera

C7: For example cholera

C1: Cholera

C7: When it goes there to feed.....

C1: We contract some....

C7: When some things remain at its mouth.....

C2: It can touch the pot.......

C1: When we kill such a pig, we do not know also that this pig has eaten such and such things

M: Is that the badness or danger of a pig that eat faeces?

All: Yes

M: What about to our health..... A pig has eaten faeces.....

A few: Hmm

M: Is it good to our health?

All: No, it is bad

M: What about to the health of a pig?

C7: To a pig’s health, it is good

C1, C5: It is good

M: How is it good?

*Laughter*

C4: It feels good

C7, C2: It feels good

C1: But it is us who do not feel good

M: We do not feel good, ourselves?

C1: Because we do not give them the feed that it wants, so it eats those things that we give

M: Is pork eaten frequently?

C7: No

M: Is pork eaten frequently?

C7: No

C1: Days pass....

M: Days pass before pork is seen?

A few: Yes

M: Why?

C7: These things are difficult because they have regulations

M: Is there a time when pork is consumed frequently?

C7: It is there

M: What time or occasion?

C1, C7: During times of disease outbreaks

M: During times of disease outbreaks, is it the time that we eat frequently?

C7: Yes, because they just die like that

C1: When it is sick, we kill it before it dies. When it dies on its own we throw it away

M: Is there any other time we eat pork frequently, C3? What time is it?

C3: Times when there is a disease epidemic, or when they are slaughtered

M: Which times do we often slaughter pigs?

C5: At Christmas

M: Is it at Christmas when we often slaughter pigs?

A few: Hmmm

M: Any other times?

C7: At feasts

M: Which kind of feasts?

C1: At Good Friday,

M: At Good Friday

C1: When an initiation ceremony has taken place

M: At initiation; any other occasion when we often eat pork?

C7: Church times; when visitors or friends come at church

M: At church when friends have visited?

C7: Yes

M: Is there any other time?

C7: No, there is not

M: When a pig is being slaughtered, is the meat inspected?

A few: No

C1: It is not inspected or examined

M: I believe you know that there are doctors who inspect meat, who examine animals before they are slaughtered

All: Yes

M: You know them?

A few: Hmmm

M: So, have you ever seen them in the village or have your parents summoned them to come and inspect pork?

A few: No

C7, C1: But for cattle only

C1: When slaughtering cattle

C7: Not in the village

C1: They are called only when an animal has a disease

M: Why do think is the reason why they don’t come to inspect pork?

C1: They are not told by our parents

C7: Our parents are supposed to let them know to come and inspect them

M: What do you think is the goodness of meat (pork) inspection?

C7: So that we know if the meat has diseases or not

C1 repeats what C7 said

M: When a pig has been slaughtered, eh?

C7: Yes

M: A pig is slaughtered and you have bought some pork, in what manner do you like to eat it? Concerning cooking preparations

C7: In terms of preparation, others have it roasted, others have it cooked

M: How do you think your mothers prepare pork? Or us girls, I think we have reached a stage when we also cook, not so?

Girls respond: Hmmmm

M: How do you cook it?

C5: Cutting and chopping it and placing the meat in a dish and washing it

C1: Washing the meat with warm water and putting it in cooking oil, cook it

C7: ....and frying it

M: After it has been fried, then it is removed from the fire?

*Laughter*

C7: No

C1: Putting in some water

C8: Putting in water and allowing it to boil

M: How many hours do you think it takes for the meat to be boiling?

C7: For others, it doesn’t even reach one hour

M: One hour is not even reached?

C7: Yes

M: What about other cooking styles, how else is it prepared for us to eat?

*Pause*

M: C2?

*Silence*

M: Have you ever seen “shockers”?

All: Yes

M: How is “shockers” prepared?

C7: They chop it and place the pieces on a pan and put it on a fire

M: Is that “shockers”?

All: Yes

C7: When water has evaporated, they then add some cooking oil and start frying

M: Do they fry the “shockers” that are found at the roadside?

C7: Yes

C1: Others fry while others do not. They just boil it until it ready

M: Until it is ready and blood is not there anymore? What about the roasted one?

*Silence*

M: Do we see people roast meat?

A few: Yes

M: Or maybe some of us also roast sometimes?

C7: We also roast

C7: We also roast when there is no relish

M: You also roast? Now, what is the manner of roasting?

C7: When we cut it, we add some salt to it, and place it on a fire cooking, until when blood had drained that is when we remove it

M: Have we ever heard that there is *Masese* in pigs?

Masese = cysts

A few: Yes

M: Have you seen them before?

A few: Yes

M: Are these *masese* good or bad?

All: They are bad

M: Why do say they are bad?

C1: Because they are a disease

C5: Those are diseases

C7: That is a disease, because those *nsembe* are associated with disease

Nsembe = also meaning cysts

C1: It means diseases increase

M: Hmm? *(Looking at C1)*

C1: There can be a disease in a body

M: In a pig’s body?

C1: No, in a human body

M: When they eat?

C1: When they (i.e. humans) eat, it means diseases increase in the body from eating such a diseased pig

M: Do you eat meat with cysts?

C7: Others eat, others do not

C1: Others do not eat, they throw it away

R: What about you?

C7: I don’t eat

M: Why don’t you eat?

C5: Because that’s a disease

M: What about those that eats?

C7: Others eat, just eat because it is meat irrespective of its status, they do not think that there are diseases....They do not think about it

M: Toilet. Do we know what a toilet is?

All: Yes

M: Do we have them in our households/families?

A few: Yes

C7: Others do not have

C1: Others have, while yet others do not have

M: Do we utilise them?

Some: Yes

M: All of us use them or they are some who use something else?

C7: Others use the bush

C1: Those that do not have use their friends’ ones

M: What about you, do you have a toilet? *(Looking at C2)*

C2: No, we do not have toilets

Both C2 and C3 nod their heads in disagreement; they are brother and sister)

M: What do you use?

C2: We use our friends’

*Laughter by all*

M: What about at your friends’ place, if you find people already occupying them? Not so?

*Even more laughter*

C1: You wait

C7: The easier thing.........

C8: ......is to head to the bush

C7: ....... is to head into the bush

M: Is it a good thing to head into the bush?

C5: No, it is not a good thing

M: Why is it a bad thing?

C7: That is where pigs also go

*Laughter*

C1: You find that when you are busy, you can find it behind you waiting

M: What about other family members, what do they use? Do they also use the toilet or go to the bush?

C7: Others use toilets

C1: Others, it is just the bush

M: What do you think many people use?

C1: Toilets

M: Those within the village?

C1: Toilets

M: So, if we came to *Wonzi* village and counted the number of toilets, would they tally with the number of people there?

C8: No

C1: Speaking truthfully, they do not match up

M: They do not match up, meaning that many people use the bush?

All: The bush

M: Is it a good thing?

All: No

M: Now, when you ask them that “why are there few toilets in the village?” what do they answer you?

C8: They scold us

M: Scold you?

C8: Yes

M: Why? Why do they scold you?

C7: Others do not have those people who can dig properly. When they look for someone to dig, they dilly dally. That is why others do not have toilets

M: Bathing, do we know what hygiene is?

All: Yes

M: Reaching your age, do they have to tell you that get water and bathe?

All: No

C1: We see ourselves that “now I have not looked good on my body” and so get water and go and bathe

M: Do we just bathe when we have stopped looking good?

C1: No

C5: Even when we are alright

C1: Even when we are not dirty. But for the body to be free and should not have dirt. It is that dirt that makes different diseases to come into the body

M: Others? Do we only bathe when we are dirty or when we are not dirty we don’t bathe?

A few: No

C8: We bathe again

C7: We bathe. You just follow that, when it is a new day, it is to bathe

M: So have you already bathed early in the morning?

C8, C7, C5: No

C5: I have only bathed on my legs

C3: I have bathed

M: Where do you get the water that you use?

Most: From a borehole

M: Is a borehole present there?

All: Yes

M: Is it functional?

All: Yes

M: Is the water inspected?

C5: I don’t know for sure

C7: We don’t know well on that one

M: What about the issue of washing hands; which times do you wash your hands?

Many respond: When we have come from the bush

C7: When eating *nshima*

C1: Even after I have been with my friends and we have handshake our hands, I need to come and wash my hands... Before eating that thing......Even when you are on a journey and you have reached someone’s home, you need to wash hands

M: When you have come back from the bush or from cutting firewood, when you return home do you need to wash your hands?

A few: Yes

C7: Aaah! When we have come back from the bush...

M: Maybe what kind of bush did you mean?

C4, C7: Toilet

C1: From playing, you have to wash hands to remove dirt

M: What about when eating *nshima*?

Most: We wash

M: Have you heard that there are intestinal worms?

All: Yes

M: Have you seen them before?

C7: Yeees

M: Have we seen them before?

All: Yes, we have

M: What do they look like?

C7: They look like snakes or earth worms

The term used here can mean both or either snakes and/or earth worms

C1: They look alike on both ends

M: Others have seen them; how did they see them?

*Silence*

C1 laughs

C7: Just like that.....looking like small snakes

C1: C7, you are pointing and indicating your finger, do they grow that big?

C7: Yes

M: What about others? My sister, you had agreed that you have seen them before. What do they look like? *(Addressing C6)*

C6: They are small

M: They are small. What do they compare with?

C4: Others are like that cable/string *(indicating the microphone wire)*

M: Hmm?

C4: Others are like that cable/string

M: In length or thickness?

C7: Thickness only

M: There are just like a string?

C7, C4: Yes

Pictures of proglottids are shown around

M: Have we ever seen such things before?

*Silence*

M: Have we seen such things before? C4 give the pictures for others to see. Have you seen these in stool before?

No response

M: In human faeces! Have you ever seen that before?

The children did not respond and seemed preoccupied with pictures

M: Those do not look like faeces, do they?

A few: No *(almost inaudibly)*

*Pause*

M: They look like meat, not so?

A few: Yes

M: But have we seen those things before? We have not seen?

C7: Personally, I have not seen. May be these have *(pointing at C2 and friends)*

This is followed by C2, C3, C4 and C6 saying they have not seen them as well

M: What about such as these? *(As she handing over pictures of pork with cysts).* That is pork? Have you seen that? That is pork

C2: *Nsembe*, *nsembe*

M: What do you see yourselves?

C1: Cysts, cysts

M: Cysts, cysts?

When C7 looks at the last picture he starts to laugh

*Others join in and laugh*

*Showed disgust*

M: For those that said they saw intestinal worms or that they suffered from it, is there anyone here who has ever suffered from intestinal worms?

C7, C1: They are there

M: Are they there?

C7: Yes

M: What did they say they were feeling?

C1: They used to have abdominal pains and when they would go to the bush, those *tudoyo* would fall out

Tudoyo = Literally meaning bugs, insects etc

C7: They would fall down looking like string made from a tree bark

*Laughter*

C1: They would just see them in plentiful numbers; even when wiping.....

*Laughter*

M: What about others? Those who removed these things, what did they tell you they were feeling?

C7: They would easily get hungry

M: Others?

*Pause*

M: Where they revealing why they were feeling hungry at short intervals or quicker than normal?

C7: They were not saying

M: You were just surprised that a person was just eating....?

C1: Yes

M: ....but hunger was not been satisfied?

C1: It would get satisfied

C7: It would be satisfied, but they would get hungry faster because those things in side used to consume some food

M: Do you think these intestinal worms can be prevented?

C1: Yes

M: How can they be prevented?

C1: They can be prevented at the clinic.

C7: They would give drugs

C1: Yes, give you some drugs so that when you go to the bush, you completely pass or defecate them

*Laughter*

C7: So that everything comes out

C1: Everything comes out

C7: You become clean

M: In your own thinking, do you think this disease is more severe than any other disease? These intestinal worms?

C1: All diseases are bad

C7: Yes, it is a disease as bad as others, but I don’t think it surpasses all other diseases. A disease is a disease because even they trouble a lot.

M: What about others? What do you think about the disease of intestinal worms? Is a disease that is fearful so much?

C4: Yes, especially when you vomit the worm and chew or bite it; one surely dies

M: When you vomit it, you die?

C4: When you bite it

C7: When you bite into it

M: The one who dies, is it the worm or you the person?

C1, C4: You the person

M: Others?

*Pause*

M: Do you think there are other minor diseases, less severe than intestinal worms?

C1: Minor ones? When a leg is painful, then it a small illness

M: Do you there are many people in *Wonzi* village that have this disease?

C1: Others have the disease, but they hide and not disclose it

M: What do others think about this?

*Silence*

M: In *Wonzi* village are there many people that have this disease?

C7: No, they may not have. Others may have it, but they hide it. You just see some getting thin and you may not know what they are suffering from

M: Do you think having intestinal worms is a very fearful thing?

C7: Yes

M: How is it fearful?

*Silence*

M: Having intestinal worms, do you think it is fearful thing to have?

C1: It is a fearful thing because they are also a disease

C7: It is fearful

M: Why is it so?

C6: Disease is bad, because you worry, are uncomfortable and fret about it that may be I will die

M: Does this illness kill its victims?

C7: Yes

C1: They kill because when you go to the bush and you see it you speculate about your own life

*Laughter*

C1: When I see it (meaning worm) wriggling like that, I also imagine and worry that such a worm can be wriggling in my intestines.

*Even more laughter*

M: What is the danger or badness of having this disease?

*Silence*

M: What are the dangers of having intestinal worms?

C1: Not caring for things we touch. Touching this or handling, not knowing that if I handle soil, I may contract the disease

C4: They say eating soil

M: Yes?

C4: Eating soil

M: Is eating soil the danger of this disease?

C7: When you suffer from this disease, the fear is eating at short frequencies

C1: Playing a lot in water

M: Let us speak properly and plainly, I don’t understand what you are saying; playing a lot in the water. When you have this disease, you play a lot in the water?

C1: No. When you stay in the water, you contract those diseases

M: What about others; what are the dangers of this disease? Let us imagine we have this disease. What then do we think are the dangers of this disease?

C7: The dangers of having this disease is that when you have it, it is troublesome

M: We want the trouble of this disease; what sort of trouble?

C1: The trouble is pain; When it starts to pain you suffer, you complain

M: What about the issue of *masese*? We have all seen *masese*?

A few: Yes

M: And we have seen pigs like that?

A few: Yes

M: We bought and ate it?

*Nervous laughter*

M: So, have we ever seen cysts in a human being

*Pause*

M: or we have heard about it?

*Silence*

M: Have we heard about it?

*Pause*

M: Have we heard about it

C7: No

M: People do not have cysts, not so?

A few: Yes

M: Why don’t we eat meat with cysts, if we do not eat it?

C1: We fear

C7: There are diseases in that......

C1: We fear eating meat with cysts because of fear of contracting diseases. We just see it by its appearance that it has diseases; can we get it and eat it?

M: What about people that fit; have we ever heard of other people that fit (have epilepsy)?

A few: Yes

M: What do you think causes it?

C1: Indiscriminate eating of different things. Others it is from childhood, congenital

M: Indiscriminate eating of which kinds of foods?

C1: Especially different or strange meats. When one is told not to eat it and you proceed to eat it. You can get sick

M: Have we ever seen people who fit?

A few: yes

M: Are they there many people in *Wonzi*?

C1: They are there

C7: They are there, but they are few

M: There are there, but they are few?

C7: Yes

M: Do you think the disease of *masese* can be eradicated?

C1: The kind of eradication.....

M: Hmm?

C1: ...is to avoid or stopping eating that pig

C7: Hmm!

C1: When you eat that pig....

C7: That’s when you will contract the disease

C1: Even you will have the disease

M: Not eating any kind of pig or what kind of pig?

C1: That pig that has them. There are pigs that have and those that do not have

M: Do you fear having such an illness?

Most say: Yes

M: Why do you fear?

C1, C5: We fear that we may die...

M: Are there diseases that are more severe than this disease of *masese*?

C7: There are there

M: Like which ones?

C1: Like AIDs

*Laughter*

M: Others? Which diseases are more severe than this disease of *masese*?

C7: TB

C1: ....and cholera

C7: Cholera

C1: Cholera comes in a bad way

M: What about the less severe?

*Pause*

M: Less severe ones, the ones that are not fearful? What kind?

C1: Headache and a boil

M: Do you think many people in the village have such kind of a disease?

C1: Others have, while for others...... we cannot really know what is happening inside their bodies

M: Do you think that when a person has a disease of *masese* or has started fitting, is it a difficult or troubling thing?

C1: Yes

M: Is a very fearful thing?

A few: Yes

M: How is it fearful?

C1: That person can get ill even when cooking. She may fall in to the fireplace without knowing and get burned by fire

M: What about in everyday life or health, if a person has a disease coming from *masese........*

*Pause*

M: What do you think about it? Is it a troubling thing or what?

C1: It is a troubling thing when you are just “sitted”

C7: Not rushing to the hospital, it is troubling

C1: But if you go to the hospital, then your life or health is helped. But if you don’t remember that when you get sick and remain “sitted”, it means your life or health sooner....

M: Sister, can you add on? *(Addressing C4).* Having this disease or maybe a person is fitting because he/she ate pork with cysts.....so that person has the disease. What trouble is there?

C4: Epilepsy is troublesome

M: How is epilepsy troublesome?

C4: You may not know that you have epilepsy as people during attacks would not know that they have fitted

M: Who else can add on?

*Silence*

M: So, prevention against these diseases; we have talked about intestinal worms, not so?

A few: Yes

M: The disease of *masese*, not so?

A few: Yes

M: Others even reach up to fitting, not so?

A few: Yes

M: Now, in order that we prevent against these diseases, what can we do?

C7: Rushing to the hospital

C1: Going to the hospital

C7: To be given medicines

C1: That’s when diseases can be prevented, instead of just “sitting”. When you are “sitted”, it means your life or health can expire

M: Like one method of prevention of these diseases is that we should be confining our pigs in kraals, what can we say about that?

*Silence*

M: Has the question been well understood?

C1: Yes

M: I am saying that there are diseases of intestinal worms, and *masese*, not so?

C1: Yes

M: For these diseases, one method of prevention is to confine pigs in kraals, not so?

A few: Yes

M: Now, you..... What can you say about confining pigs in kraals without them venturing outside?

R: Can there be any difficulties or hindrances of confining pigs in kraals?

M: Has the question been understood or not?

C1: It has been understood. Now we are just looking at how to respond

M: Let us look at how we can respond

R: Can there be any difficulties or problems you may find if it is required that everyone must confine pigs in kraals?

*Pause*

R: Have we understood? ...Can you be pleased with it?

M: Can it be a difficult thing to confine pigs in kraals?

C7: Hmm

M: All of us there at *Wonzi*, each one to get their pigs and confining them; each one who has pigs to confine them in kraals, can it be a difficult thing?

Most: No, it cannot be a difficult thing

M: It may not be a difficult thing? What about pig feed? Where will be coming from?

C7: It will be us to take feed to the pigs in the kraals

M: Can we manage?

C7: Yes

M: What about if we said in *Wonzi*, in order for us to prevent these diseases, all of us should build toilets; everyone should be using a toilet as long as he/she is a human being; what do you think about it? Can it be a good or bad thing?

Most: It can be a good thing

C1: Because.......

M: How can you say it is a good thing; when people go in the bush pigs also feed on faeces, not so?

Most: Yes

M: How can be a good thing?

C1: It is a good thing.....

C7: Because when we use the toilets, it means pigs won’t be eating those faeces

C1: They will be eating good things only; when we go to the bush that pig can be following us even without us being aware of it such that when you are done you find the pig comes to eat them

*Mild laughter*

M: What about the preparation of meat? If we said when pork is bought in this village it needs to be boiled...Do we know what to boil means?

All: Yes

M: Do you think it can be a good or bad thing?

Most: It would be a good thing

M: How is it a good thing?

C1: It removes those........

C7: Those tudoyo-tudoyo that are in there

Tudoyo-tudoyo = germs

C1: Get killed by that water

C7: Those things are removed, they get burned

M: What about if we say those personnel that look at animal health should be coming to inspect your pig before and after slaughtering it; what do you think it would be?

C7: It would be a good thing

C1: It can be a good thing because when we have slaughtered it, sometimes.......

C7: We don’t know.......

C1: .......if it has disease

C7: If it has diseases or not. We just eat it unknowingly, or maybe that meat has been well cooked, and after we eat it we contract some diseases

M: What about washing of vegetables? Vegetables such as *Ciwawa*, rape, cabbage, Chinese...You know them, not so?

Ciwawa = pumpkin leaves; cabbage, Chinese = another type of cabbage called Chinese cabbage

All: Yes

M: If we say every person when cooking this relish, he/she is required to wash them first. Sometimes, we do not wash *Ciwawa*, not so?

All: Yes

M: Now, if we say every vegetable needs to be washed; can it be a good or bad thing?

All: It can be a good thing

C1: Because there are usually many things that are present on these vegetables such as dirt, different insects and bugs in them.....So that if there is water, when washed the dirt can be removed

M: What about the issue of hygiene? Washing of the whole body, what do we think? We said we only bathe when we see that we are dirty, that when we see that we are not dirty we don’t bathe. Isn’t that what we said?

C5: Yes

M: We only bathe when we are dirty, nor so?

*Laughter*

M: On the issue of bathing, what do we have to say?

*Pause*

M: What do we say concerning the issue of bathing? C2, can you speak also? Is bathing a bad thing?

C2: No

M: Eh?

C2: It is a good thing

M: Why is it a good thing?

C2: Because it removes some diseases on the body

M: Any other thing? What do we say concerning the issue of bathing? Parents have to beat us up for us to bathe, not so?

Most: No

C1: We have already outgrown our parents. When you have bathed, you will sleep well. If you have not bathed you be surprised that your friends start chiding and teasing you. You will see and judge yourself; you may have cracked heels that even blood comes out of the cracked feet

*Laughter*

M: What being given drugs? If we say if you have such diseases, you need to go to the clinic/hospital to be given drugs if for example you have intestinal worms, what do you think about it?

*Pause*

M: Is it a good or bad thing?

C1: It is a good thing to be given drugs...

M: How is it a good thing?

C1: So that good health and life continues or improves

C7: When you have been on a course of medication, it means even those worms that are inside would have been fatigued

C1: They would be fatigued and not share the food. They would be ... *(sayings something that is inaudible)*

M: Do we all know what vaccination is?

All: Yes

M: Have we ever seen vaccination of pigs?

C2, C5, C6: Bored, restless by now

C1: We only hear about it

C7: We just hear that there is vaccination for pigs

M: Have you ever gone to *Nyamphande* to take pigs for vaccination?

*Nyamphande* = where the veterinary assistant is centred

C7: Others have gone while others have not

M: In your thinking, do you think it is good or bad thing?

C7: It is a good thing to take pigs for vaccination

C1: Because when you take your pig you will know that my pig is not well, it has a disease. By you taking it there it may be given treatment so that that disease ends

M: Who else can add on concerning taking pigs for vaccination?

M repeats the question

*Silence*

Ruth summarises at 11:17h

M: I believe we have come to the end of our programme, not so?

Most: Yes

M: So today, from the beginning we discussed about issues of pigs, the goodness and the dangers, how we keep them, how we feed them, until we reached diseases, those diseases that can be contracted coming from pigs, like intestinal worms or *masese, not so?*

Most: Yes

M: Now we have reached at a stage of how we can prevent those diseases, we talked about confining pigs; they should be going outdoors, having toilets, until we reached vaccinations of pigs. So, if you have any question concerning the issues we have discussed, you can ask? You should be free to ask, ok?

A few: Yes

M: Is there any question? Let us ask

*Silence*

M: You do not have any questions, right?

C7: We do not have

M: We do not have?

C7: Yes, They are difficult to come up with *(laughs)*

M: We are very thankful to you, do you understand?

All: Yes

M: We are grateful of you!

About 35 seconds later

M: You are not taking your drinks?

C5: We are drinking

M: Why aren’t you drinking them then?

R: Then give me those drinks I take them *(addressing the children)*

I (Dr Kabemba): Who? Naomi
