# Supplementary material for: Why Latrines Are Not Used: Communities’ Perceptions and Practices Regarding Latrines in a Taenia solium Endemic Rural Area in Eastern Zambia
Source: PLoS Negl Trop Dis. 2015 Mar 4;9(3):e0003570. doi: 10.1371/journal.pntd.0003570 (PMC4352092; doi:10.1371/journal.pntd.0003570)
Supplement: S1 Dataset — (ZIP) [file pntd.0003570.s001.zip › FGD transcriptions_Zambia-2010/Wonzi_Dr Phiri/Men_Wonzy_30-07-10.doc]

**Title : Focus Group MEN**

**Date : 3**0/07/10

**Site :** Kakiwa Rural Health Center

**Village :** WONZI	**Location :** Petauke district, Eastern Province, Zambia

**Duration (total time) :**	65 min	**- Start :**	13:20 pm	**- End :** 14:25 pm

**Participants:** 8 men

**Informed consent:** signed and available

**Moderator :** Mr. Emmanuel Mwanza	**Recorder :** Dr Andrew Phiri

**Transcription/translation :** Dr Andrew Phiri

**Abreviations :**

**M : moderator, R : recorder, I: intervener, C : children, W : women, M : men**

**Disposition (from the left of the Moderator):**

**M**

M1

M2

M3

M4

M5

M6

**R**

M7

M8

Age composition:

M1: the oldest (>65yrs)

M8: the youngest

M4-M7: young

Typology:

M5: dominant

M4-M7-M8: quiet

**Comments about participants :**

M2 is the headman.

M6 was reluctant to sign consent.

Oldest was commenting frankly.

M5, M7, M8 act in a local drama group. They were very acting their answers

**Group dynamics :**

Level of participation was very active and interactive

Interest was very high especially on confinement of pigs and toilet habits

Sequence was appreciated

**- Emotional aspects:**

Laugher on meat inspection and destroying/throwing away of condemned meat; laugher and animation of pork preparation and consumption. Reluctance on worm differentiation, needed helped description. Loud laugher on washing of hands, use of toilet.

**- General comments:**

Local language was used punctuated with symbolism and examples. Spontaneous relevant discussions after meeting concerning the drama group incorporating issues of cysticercosis/taeniosis in a play.

**Text:**

*Introduction*:

M: Thank you very much. Firstly, I am grateful that we have managed to meet at this appropriate time. The first thing I wanted to do is that we introduce each other. I will start and then each one will follow suit. As we do that, we need to be speaking out loudly so that the voice is heard clearly

He proceeds to introduce himself and every man follows suit

The research team is introduced later

M: Thank you very much. Just like what the doctor said, the main reason we are gathered is to share knowledge about the welfare and health of pigs, the way we live with them in the villages, the entire life of the pig. Everyone is free to take part in sharing this knowledge. You are free to answer the questions I will pose in order that we share information

To begin with, those pigs we keep how necessary are they? How needful are the pigs?

M3: The reason why we keep pigs in the village is that we get relish from them

M: Good, anyone else?

M1: A pig is helpful when we encounter problems

M: What kind of problems?

M1: Like at funerals; they would say “go and get one pig so that our friends who will come would eat some of it”

M: Others? Pigs?

M7: Pigs help us in various ways; when I am troubled by weeds, I slaughter a pig and hire agricultural help to weed them out. If I rear pigs and wish to make a kraal, the same pigs can help me do it

M: Hmmm...Any others?

M5: Another thing: When pigs have been kept and a child has passed at school or even a child has not passed, I need to get one pig and sale it so that that child continues with school, the fees that I have been charged with at school

M: Yes, yes. I belief we are moving together here. What are the dangers or badness of pigs? You just explained the good things of having pigs? What are the dangers that you find in the life of pigs?

M5: The danger that we find is the way they eat, the way a pig eats; it does not care what it eats. That’s the danger that we find in that it eats indiscriminately. Even when I have gone aside, it is capable of shoving me off and starting to eat. That is the danger that has been encountered

“It is capable of shoving me off and starting to eat” = symbolizing that when he goes to the toilet the pig can access the faeces and eat them

M: Others?

M3: The other danger of pigs is that even when a person dies and we have not properly cared where to bury that person, a pig is capable of exhuming that person and eating him/her; even when a dog dies, a pig can eat it. That’s the danger of pigs

Bury that person = i.e. buried in shallow graves

M: Others? The dangers of pigs?

M8: The danger of pigs is that even when you have thrown water on the ground, a pig will come from anywhere and wash at that place

M: In the dirty water?

M8: Yes

M: Very well. Now in the village, the keeping of pigs.....How do we keep the pigs?

M2: Some make kraals where they are confined while others let the pigs roam freely

M1: Let them roam about

M3: Other pigs just sleep outside; they do not have a kraal. The owners just feed them by pouring maize bran on the ground. The pigs just wander and sleep wherever they feel tired

M: Why is it that many people do not build kraals for pigs?

M3: It is just laziness

M4: It is just laziness of the pig owners

M1: Long time ago, we used to build kraals for pigs or goats because there were greedy predator animals like hyenas or leopards. Now these animals are not there anymore to instil fear of catching our livestock. Now our livestock just sleep outside...even goats, pigs since hyenas are not there

M5: Even for a human to catch a pig!! It is different from a goat; they can quickly and easily capture and silence a goat. Now for a pig even if they whip it any kind, it will continually scream and people would be alarmed when they hear it and say “Hey, it is not alright this side” *(Miming a scream of a shrieking pig, and a pursuing owner or another person)*

When you reach there you just find the intruders crouching and say “yes here are the thieves”. That thing personally demonstrates and points a problem by itself

M: Good; now, you have talked about not building kraals and that they just roam freely and wherever it is weary, it lies; you also give it maize bran on the ground. How do you know that this pig is mine?

M4: Ear notching

M1: When they roam around they still come back at home until you give them feed

M2: You give them a sign like notching their ears

M: If alone for example, you have notched an ear and others also do the same, how would you differentiate them and know that this pig is mine?

M2: The way that ear has been notched

M1: Others pierce them

M4: What is yours is yours; you cannot fail to identify it

M3: Another thing for us to know is that if I have reared pigs and this one *(pointing at M2)* is my neighbour. If a person is found to be holding this pig, they would be told that this pig is for my friend over there

M1: It is not yours

M3: You will fight. So those people leave it. Thereafter, they will come and tell me that one of your pigs doesn’t move about well, that will give me a plan and when customers come, that will be the pig to sell

“Pigs doesn’t move about well” = meaning moves about a lot

Customers = i.e. traders

M: Ok, fine. What benefit is there in building a kraal? What benefits can you find in keeping pigs in kraals?

M7: The benefits and goodness of building a kraal is protection of what foods to give them. You know, when I have built a kraal for pigs to sleep in, it means they will be eating the feed that I will be giving them myself. They will be no need for them to be wandering about and going in the bush and eating unwanted things

M6: Another thing, especially during the rainy season, during the rains pigs suffer a lot. When rains come down, pigs are always running about, going in there and there. In that way, they do have a good life

M: Can others add on the benefits of kraals?

M4: The benefits of a kraal, I have seen, are that they get protected. Where they go they may meet other pigs with a disease outbreak (referring to African swine fever). Because of that contaminated air, the pig can contract it. When they are in a kraal the disease outbreak skirts those in a kraal

M: Ok, fine. There are those pigs that roam on their own; what difficulties do you encounter?

M3: There are many difficulties

M: Fine

M3: The problems, 1) It can come across diseases and get sick; sometimes while out there unknown people can break its legs. It returns with a leg that is damaged; sometimes while there they can kill it and leave it like that. Those are the difficulties we meet

M: Fine, the keeping of pigs at home, who at home feeds the pigs, when it comes to household duties?

M1: It is a woman

M: Woman!

M1: I may leave the house, so it is woman who keeps

M: Very well

M3: and a child

M: Anyone else who may explain the keeping of pigs in the villages we live; how do we keep pigs?

M5: Three-quarters I believe...A young man is one who goes here and there. The women and children are the ones who remain at home. Sometimes, you may leave it to a wife or sometimes to children with instructions that “keep, take care and feed these pigs”. Most times, these are the ones who feed

M: Is there anyone else who may add? Yes *(addressing M8)*

M8: I can add on here; Women are the ones that stay at home. For example, visitors can come and not find me but would find my wife as men are not usually at home. I can also leave instructions with a child to be giving feed to livestock at home

M: Very well, very well. The one with absolute authority or maybe as you have explained that sometimes you have difficulties, you need to have relish. Who has authority among the father, mother or children?

M5: As now if there is a problem, so the one to have authority is me the man who is at home. I need to tell them that “look at this problem that has come. Let us catch this thing, may be selling it and let us be helped in this way”. It may be a funeral. In that case, I would need to ask my clan cousins to catch that thing and kill it so that we use it for food with those that have come for the funeral.

M1: It is a man who points that: “Get this thing to help us”

M: So you have said that children may be tasked or have a chance of putting them in kraal. So at no time does a child have a privilege to comment or say on things?

M1: They say something

M2, M3: The privilege for children is there

M3: Even for the madam, a privilege is there

M1: Even when they wish to eat something good, they may say “Father, let us eat, slaughter this so that we eat something good today”. I would say “Very well, you want to eat pork. Go ahead and slaughter one of them and we eat”

M: Very well

M1: We listen to children

M: Hmmm

M1: When they complain

M: So if we say the feeding of pigs; how many times per day do we feed them, from sunrise to sunset?

M6: We feed once, especially in the late afternoon before nightfall and before going to bed. This is what makes it that they eat once per day because sometimes the availability of feed is poor. We feed them maize bran. Now when mealy-meal has finished in the house or you had recently milled maize and mealy-meal is there but maize bran has been depleted. When we see that maize bran stores are little we start to feed them with small rations. Sometimes when maize bran has run out, they remain hungry

M: Others who may wish to add on? Others say we feed them once

M3: Once a day, when they eat it would be in the morning.....

M1: You give once in the evening as they go to sleep or if they have a kraal you call them to the kraal shouting “to eat, to eat” and you feed them when they would have entered in the kraal. Once they have eaten, they will only eat the following morning

M3: When they eat in the morning, it means they will last the whole day without eating

M: So, from morning till evening, what do the pigs eat?

M3: It will be picking and picking

M5: It is now self-reliance

M1: Picking here and there, eating wherever it goes. It finds food on its own

M: The way you have said pigs move freely, and it is only in the evening that you feed them. What benefits.....what difficulties do you find with those pigs that freely roam?

M7: The difficulties that we find are. .....they eat in the evening and in the morning when they come out they just move freely on their own, going in the bush and eating unwanted things. When it is evening that is when we call them, we find others are muddy, have unwanted things all over their bodies. And then we would give them maize bran and they go and enter the kraal. The following day, we do the same thing

M: Hmmmmmm: So in the life of a pig, when are the times that we use pork? What times in a year is pork frequently eaten?

M3: The times of *chisalo*. Starting from the times when we have harvested, from June, July, and August until October. That’s when pork is consumed a lot.

Times of *chisalo* = July-Aug

*Pause*

M3: When it comes to times when we do agricultural activities, it is at Christmas..!

M5: ......and New Year

M4: New year!

M3: That is when they are eaten a lot

M5: That is when they get killed

M: Very well. Are there any who can add on?

A drink belonging to M2 falls and distracts people momentarily

M: For example, you have pigs at home. So among its friends how would you know that this pig is sick?

M5: A sick pig when it is among a group of them is observable; it doesn’t lift its tail like its friends. Its tail is droopy. Then when you see its hairs, you see it looking scruffy *(demonstrating)*

M1: Ruffled

M5: And it fails to move about and you notice it’s snout on the ground; it fails to lift it high. It only reaches around this height *(demonstrating a short distance).* It only wants to lower its snout closer to the ground.....so you know that our buddy is....

M1: Is Sick

M5: It needs treatment, only found at the general

At the general = meaning the General Hospital in town

M: uhhh, uhmmm

M5: Sure, sure

M6: Another thing to know that a pig is sick is that when you call them and you give them feed, but you find that a particular one is not eating. You then know that then this..........

M1: The pig is sick

M: Very well. How would you know that this pork is good? What would you use to know that it is good meat?

M3: The smell/aroma is number one. Two, it should not have cysts

M: Aha

M3: It looks like a meat that has good proportion of a fatty layer; that is what we use to know

M1: And when it has been slaughtered for meat, you see that the blood is good; the meat has fats, it does not have cysts......aahh!!! You say this meat is good

M: Oho!

M5: And another thing is that, if pork is good, when a good animal has been slaughtered, it is clearly observed by sight. It looks clear or whitish. You can then conclude that this meat is good and that this meat has not been touched with diseases. Now the one with diseases is not mistakable, even when the animal is slaughtered, its meat is clearly seen that it is reddened in appearance and even inside it is observable that it is changed.

You would say “this meat is pork but it doesn’t look to be ok”

M3: Even when you want to remove the hairs, they remain intact on the skin. It is known that this pig was sick and even when they move about with it in the village, that meat has good fatty layer and good blood, and it is singed properly, it is known on the aspect of the blood

M1: Just looking at it

M3: Just looking at the meat, you tell him that this meat is from a pig with African swine fever, and it smells differently or has a distinct smell

M1: And it’s reddened; you know that this pork is not good

M5: The blood shows on its own, when you put the meat on a palm like this *(demonstrating)* and when it has been hit by heat of the sun, the blot clots; But when it is from a diseased pig, you find that the blood is thin......you know that this was not healthy

M: Very good. For example, when slaughtering animals, does the meat we eat get inspected?

All: It is not inspected

M1: It is not inspected but at the Boma it is inspected

Boma = i.e. at Petauke town

M: What?

M1: When pigs are slaughtered, they are inspected. We do not have the means of inspection in the villages

M: I have seen a hand there!

M6: It is not inspected here in the villages. As long as we have slaughtered the pig, we just sell. So those at the Boma or nearby follow the local council regulations. But here we are free because there are no veterinarians whom we can tell to inspect for us

M: Very well. What do you think would be the goodness or benefits of inspection? The goodness of pigs, to say the benefits I have found are so and so

M1: The goodness of inspection is that when they come, they clearly notify you if the meat is diseased or not. That is good because you will know now things will go like this, like that. They tell you plainly

M2: They tell you explicitly that this pig has diseases

M1: It is not looking good

M: I have a hand there, a contribution?

M6: It is good to have inspection like we have already said that when it is sick we just eat it without knowing that our life or health will be destroyed. So when they have inspected, they will tell you that this relish is not supposed to be eaten but to be thrown away. So, that is the beauty of having meat inspected

M: Very well. For example, the inspectors have inspected and they have found that “Mr Daka,, your pig is not alright, Mr Mwanza your pig is not ok, the whole carcass!” Can you agree to, generally speaking in the whole village, can you agree that the whole pig should be buried?

M1: Yes, we agree. When a pig has died on its own, we have seen that there’s nowhere where someone has stabbed it, we say “go and throw it away”. So it is the dogs that eat

M: Dogs?

M3: Pork!! Truthfully..........!

*Loud laughter by all*

M3: If pork has a good fatty layer

M5: It should be cooked

M3:...And it has been found with a disease but my eyes have not been satisfied

M5: Just cooking it

M3:...it is cooked

*Loud laughter*

M3: That kind of pork is edible. Just when the doctor leaves, I would get it

M1: It has a fatty layer

M3: We would say, “We are going to grill it”

M: Good, I have seen a hand

M6: It is not possible and it would be difficult to throw the meat away. If the doctor has inspected it, but says...

M5: It has diseases

M6: It has diseases, you see what we do in the village; that it has died on its own, and it may even been rotting, but a person says “I will take this thing and grill it, grill it very well and use it for relish”. Even when we would have people from the veterinary department nearby to inspect animals for us, there may be times when the vet people tell us to throw away, we ourselves may go behind their backs getting that meat and going to have it cooked

M5: Even times when a pig has clearly been seen as having diseases, even if a vet person told me that “throw away this meat”, I would tell my children “go and singe this pig”; the following morning even the offals would be in a pot

M: Hmm, hmmm, hmmmm

M2: For example, the young men that buy and slaughter cattle to take to Lusaka, they have them inspected, but even when they have been found with a disease, they don’t burn them. Instead, they are still taken to Lusaka

M: Hmm, hmmm, hmmmm

M2: Even when they dissect for example a liver, because I am in many instances also involved in skinning of animals.....

M: Very well

M2: When they have dissected a liver and the doctor has left it somewhere

M: Hmm

M2: It is scrambling for yourself and saying now my meat will be plentiful and you hide that condemned liver

*Laughter*

M5: Now they see that this is where the disease or lesion is, and the doctor has dissected it out and thrown it away, that is the one they pick up and place it into a plastic and request the wife to prepare a soup with.

Now they see that this is where the disease or lesion is, and the doctor has dissected it out and thrown it away, you know doctors also crave for meat. They will have cut it off and thrown it away; and now even myself I pick it and say “woman here”

*Laughter*

M1: This meat is very good, but it hasn’t looked good

M: So, when a doctor has discarded the condemned meat, don’t they know that you have collected the discarded things?

M5: They don’t know that I have taken that thing; In fact, I would have increased the meat/relish at home. Even my wife will set out some more of the meat for me, even the one special one on the side, I will be ok

*Mild laughter*

M2: You can even invite your friend, that “there is some meat here”

M: So, when you think, can’t you contract diseases in such a manner; when a doctor has condemned the meat as being unsuitable and you secretly take it away and ask your wives to prepare it; don’t you think there is a problem there?

M8: The problem is there because it is like transferring disease from there to our homes. That is the biggest problem that can be encountered

M: Hmm. Uhmm

M8: It means all of us at the household can get sick

M1: Others after taking that meat with a disease, they say that all the diseases or their germs will be destroyed when I cook it in a pot

M: That all the diseases will be burned?

M1: That all the diseases will be burned, the meat would be fine....hmmmm

*Laughter*

M: So, in preparation of pork, what type is very tasty? *Shokas* or boiled one? The way you eat it in the village, the one that is very tasty, how is it prepared?

M4: Boiled one

M2: Many....

M: Boiled one?

M4: Yes

M2: Many like it roasted...Yes, the roasted one

M3: Even when a pig is slaughtered.....Let me say explicitly that the neck muscles *(indicating his neck as he contributes)*

M: Very well

M2: We need to roast them. That is the first meat to eat

M: This one here *(indicating neck as well)*

M2: Yes, this one here

M5: The muscles at the neck

M3: We eat and finish that one; that is when they cook the rest of the meat. We ignore or don’t bother that those germs can die or not because we do not have such a thought

M1: That kind of eating is done in a hurry

M: Very well

*Laughter*

M1: However, the cooked one is the best and not the *shokas* one

M: So the issues of intestines, speaking truthfully the way we live in the village, how do you take care of them, do you throw them away or the intestines are also used as relish?

M2: They are relish

M7: Intestines or offal are made relish. But in the past...

M: Hmm?

M7: In the past, we never used to eat them; we used to throw them away. Nowadays, it is relish, if someone has chanced the intestines

M5: Those are offal

M7: It means at the household one has been healed or satisfied

M1: The plenty fullness of them makes one consider that he has fulfilled something

M6: They are also cheap

M6: We eat pork with cysts. Never heard of people throwing it away

M2: Others eat, but grill it first to overcome the cysts. We do not, however, consume

M: So there is no thinking of the bad things that the pigs consume?

M7: No, there we wash and clean them properly

M: Hmm?

M7: Removing all those unwanted things, washing properly until one knows that here I have taken good care. These intestines are now relish

M: So there is no danger whatsoever now that you have washed and cleaned them?

M1: There is nothing; if there is any disease they will also be removed by heat, they will get burned

*Laughter*

M: So, I believe all of us know what cysts are?

All: Yes

M: Have you ever seen cysts before?

All: Yes

Pictures depicting pork with cysts are passed around as the discussion continues

M: How do cysts in pigs come about?

M5: Cysts come about in pigs from the manner in which we feed our pigs, not caring for our livestock. We can get brewers’ mash...

M: Very well

M5: .....and give the pigs the mash; it means we have caused our pigs to have cysts. We can get mealy-meal flour from the grinding mills after a milling process and the leftovers are lying around the mill and diesel has accidentally been mixed with it

M3: Oil

M5: Oil, and we gather those and we give them to pigs. In that case, cysts need to start growing in the pigs; Even when you have caught a pig when it is still alive and you get a stick and place it in its mouth and you check its mouth *(indicating the side of the mouth);* because it shows around the sides of its mouth. Even when a pig is alive, it shows that this pig has cysts or not. They are seen inside the mouth

M1: When you turn the lips inside out or side of the mouth

M5: When you turn the cheeks inside out *(demonstrating).* They are seen here, here and there; looking white in colour

M: Very well

M5: That this pig has cysts

M: Now, the animal has been slaughtered, we have reared a pig and now we have slaughtered it, and we have found that this pig has cysts, is it possible that we throw away the meat?

*Silence*

M: Pork with cysts?

M6: I saw a pig with cysts before, now there is choosing for everyone, the way everyone has said, if a pig has been found dead and the owner has said he cannot eat it, but there are others who dare that “myself, even if this meat has cysts, I will get it and grill it and cook it”; but I have never seen that it has been thrown away on the account that it has cysts, it is eaten

M: Very well, what about if it is business, can you take it for business?

Most: No, no

M5: And also, if the one who has bought it and he has proceeded to slaughter it, he would return it to you; he returns it to you saying “I have found that your pig is not good;

M1: Look at these cysts

M5: ... Look at these cysts, so get it back

M1: Return my money and get back your meat

M5: ...And give me another one instead

M2: We people are also different, when we see that it has cysts, some of us refuse to eat it and say “I can’t eat this one with cysts”, but others eat

M: Hmmm, hmmm!....aha?

M5: These others who consume such, three-quarters have it grilled. They grill it until it is dry, because when they cook, those things *(meaning cysts)* do not get cooked

M1: They are still white

M5: You find that it is still fresh at that site because the grain is inside and the water is outside the grain; so after you cook it you find that it is not cooked at all; even when you roast it at the fireplace, that thing doesn’t get cooked; but you should grill it and all the water is drained such that when you get to cook it, aaah, it is *(demonstrating eating it with nshima)* good morning

*Laughter*

M: I guess we know what intestinal worms are?

The term used is synonymous with round worms. A picture of faeces with tapeworm proglottids is passed around for all to see

M4, M5: Yes *(in a small voice)*

M: Have you seen intestinal worms before?

A few: Yes

M: Where have you seen intestinal worms?

M5: I have personally seen them in a pig

M: Hmm!

M5: In the intestines of pigs

M: Aha?

M5: I have seen them just after slaughtering. After slaughtering and singeing the hairs because it must have been getting hot inside the abdomen as the carcass was swelling up, the worms started coming out from the back as they did not have room inside. We even pulled out some of the worms. After opening the intestines we found plenty of intestinal worms *(demonstrating worms twisted together in a ball)*

M: Very well, in our villages, have we ever seen intestinal worms in a human being, whether in a child or anyone else who has intestinal worms?

A few: yes

M: Very well; how were they looking?

M4: They were looking, whitish-whitish

M: uhmmm

M4: Like a snake

M: How do you know that someone has intestinal worms, like in a family?

M5: The way one looks, the way they eat;

M: Hmm!

M5: If that person has intestinal worms, he/she eats a lot; eating in a powerful way. Shortly after eating, he/she feels hungry again. Even you look at the person, the abdomen doesn’t look normal; it still looks as if it is full or swollen up

M4: Like someone who is swollen

M5: The abdomen is filled up so, indeed but shortly feeling hungry even after eating recently. Frequently, that person will be looking for food from pots and even looking for maize grain from granaries to roast

M1: Others have poor appetite; they fail to eat a lot. When they eat a little, they stop. You ask them “are you satisfied?” they would respond “yes”.

M: Hmmmmmmmmm

M1: Even when a different food is given to them they just eat a little as well and say they are satisfied. You then conclude that this person has a disease

M: Is having these intestinal worms, as a disease, a fearful thing? If it is fearful, how fearful is it in having them?

M8: Having this disease of intestinal looks like a fearful thing because any mistake with that disease you are supposed to die

M1: It needs to go to the clinic

M8: Because food within the intestines reduces for the worms; that food that you are supposed to use, they get it; you never have any food at all; you do not even have enough strength

M: Very well

M4: It is not a life also that when you go to the toilet you remove worms; that is a bad life; you cannot gain weight with the same intestinal worms

M: Who else can add on the badness of intestinal worms? How fearful are they?

M3: The fearful nature is that disease sometimes is that when the worms are numerous, they even come out of the mouth; so others who educate say that when you bite it when it is coming out, it means that you have made poison that can even kill you

M1: Until you die

M3: That is the badness of those things

M: What about others? What is the danger of this disease? Yes my brother *(addressing M5)*

M5: The danger of this disease is that when these things are many in the intestines, you yourself will eat, now when they are many it means you will lack strength from the eaten food, so what follows is that you start to swell up *(touching the knees)*, you begin to swell because food in the abdomen will lack any sort of strength. So, in our bodies, for example if I have intestinal worms, even if I eat that food it will have no use whatsoever, I will just put or deposit food in vain there, because they will receive it. It means my body will have no kind of energy. Finally, I would start to swell up

R: Apart from round worms, are there any other types of intestinal worms you have seen?

M: Hmm

R: Those kinds of worms that look like a tape

M5: Those that are flattened

R: Yes, the flattened ones; have you ever seen them?

M1, M2, M5: Yes

R: In faeces?

M5: Yes

R: In faeces of children or adults?

M5: Uhmmm

M3: There are found

R: Are they found? Those flattened ones?

M5: Yes, those really flattened one, like a peeled skin of a snake

M1: Elongated ones

R: Enhee, pieces and pieces of them. Have you seen them before?

M5: Yes, the shortened ones *(demonstrating on his fingers)*

Up to this stage the group did not indicate the difference between round and tapeworms worms as a *Nsenga* term combines all types of worms. After clarification, most admitted seeing proglottids in human faeces

M: So this a picture which had been passed around

A few: Yes

M: Are there any kinds of diseases that are more severe, those that you see in the village, than those caused by intestinal worms?

M1: More severe diseases?

M: Yes the ones that you know

M1: The ones that we know are those they announce, the one they call AIDS *(as he runs his left hands nervously in his hair)*

M: Very well

M5: And TB

M: Can intestinal worms kill a person?

M5: Yes, they kill

M: These intestinal worms, you know they are different types of them, can there be any protection against these diseases?

M5: Protection, the way we have already heard, the ones that can offer protection are these big people *(pointing)* that have come so that here in the village areas we are saved; three-quarters, there are many children that remove those things here

M3: You know we don’t know these things

M5: To know for sure that we can do such a thing to eradicate these, we are left confused. So now that these people have come we are happy because they can bring effective and proper drugs so that we save the children in the village

M: Very well....very well; Any other who may add on?

M3: We do not know salvation, because when you feel abdominal pain, you go and dig up some roots thinking that it’s just a stomach ache, but there is nothing that happens. So the ones who have come are the ones to educate us on what to do

M: Hmmm, Hmmmm. I believe that the issues are well understood. Let me say another question is: How do you see the goodness of a toilet? How do you look at it?

M1: The goodness of having a toilet at home

M: Aha

M1: When there’s pain in the abdomen and you have the urge to use the toilet you can easily enter and use it even when it is raining. Secondly, when a visitor has come to your home and asks you where the toilet is, you just indicate that “there it is!” You cannot point him to the bush. That is the goodness of a toilet

M: Aha! I have seen a hand over there

M5: The goodness of a toilet is; the greatest of them is our own protection because of diseases because those diseases...When I have gone to the toilet I need to be relieved instead of going to the bush. When you are in the middle of defecating you can easily be met by someone who is also making quick glances around and in rushing away, you leave with the job half done in order to complete it elsewhere near another tree. That way doesn’t show well, but in the toilet because all the diseases that I have.......

M1: Will remain there

M5: ....even if I am suffering from dysentery and others, all those will go in there. It means I would have saved others

M1: Saved others

M5: ...As well from the disease that I have

M: Yes, yes; what is the goodness of a toilet my brother? *(Addressing M6)*

M6: The goodness of a toilet is: 1) protecting ourselves who have a toilet. Just as we have already said, in the village we rear pigs; we don’t confine them and so they roam anywhere. So if we have a toilet, it would be very difficult for pigs to be eating those things that come out; Chickens won’t have access to those things. Even flies, if you have defecated outside, many flies will go there. It may be behind a house. When flies come from there, from there, they land on the *nshima* you would have served for instance. In that way even our health and life becomes......

M1: Shortened very much

M6: ...not a good one

M: Is it that all people use the toilets? Or are the toilets sufficient where we live in the village that all people are using the toilets properly?

M4 and a few others: No

M: Anha? *(Pointing at M6)*

M1: The major thing is laziness. However, a law is there if a person has a house he/she needs to have a toilet.

M: Very well

M1: Nonetheless, in the village there is a few number of toilets, may be three. You notice that all people go in there

M: Others?

M7: They go to the bush

M: Any other?

M1: Others go to the bush *(laughing)*

M: Why do they go to the bush?

M4: Lacking a toilet at home

M1: Laziness of building toilets

M: Laziness. So, in being lazy in not having a toilet, what is the benefit that can arise from not having a toilet; what problems can there be in such a case?

M3: They are many; 1) You can suffer from dysentery 2) You can suffer from what they call diarrhoea; you can suffer from it. Now, when you get sick of it, it can be spread and affect others, it cannot fail to do so; but if you have a toilet that is the only way you will prevent it...That is the goodness of having a toilet

M1: Problems of not having a toilet, when rains have come and you do not have a toilet and you have the urge to use the toilet, now it means you will go in the rains in order to defecate

*Others laugh softly*

M1: Now, if you have a toilet as I have already spoken ... actually a roof is there

M2: There are those that rely on others; When other people nearby have a toilet, they would say that they cannot build theirs because so and so has one. In that way, the available toilet fills up very quickly and thereby giving problems to the owners of toilets to build another one! Have you seen?

M: Unha?

M5: For three-quarters, it looks like it is a habit, such that even if a person says “let us build toilets”, you find someone because of habit would retort “Hey you give me an axe!”; now that is the father,

M: Very well

M5: “Give me an axe you, Chipisani”. He would get and put an axe on the shoulder meanwhile he has felt the urge to use the toilet. Like that he would be on his way, without knowing that, that child should not be aware that the father is going to the....

Chipisani = a fictitious name of a boy

M2: The toilet

M5: He would clutch on the axe and put on his shoulder *(demonstrating)* and off he goes. While in the bush he would do everything and leave that “funeral” there and upon returning he would carry a piece of firewood on his shoulder and staggering with it, he would pitch it in the yard with some pomp. Meanwhile a child would think that “my father has come from cutting firewood” meanwhile he had gone to discard some other things. So that one is a habit only, such that when you tell someone that “let us build a toilet”, he may not understand

M: Is that preserving self respect at home such that children do not know or how is it?

M5: It the self respect of the father alone so that the children do not know that their father has gone to the toilet. In that way, it is hiding from the children

M: Yes, yes, I saw a hand here, my brother? *(Pointing to M6)*

M6: The issue of toilets in the village, the Health professionals have tried to announce. They have taken care to announce that at every household, there must be a toilet. So the way we have already discussed, that is usually laziness. The end result of that laziness is that the family would suffer from diseases frequently. The victims would say “I think there is someone who is bewitching my children; they get sick often”, but we have forgotten that we heap our problems behind the yard there. So, the professional people have tried to announce that all of us should have toilets. In fact, sometimes we just invite these diseases ourselves.

M2: Even yesterday, we were at a meeting concerning the same toilets. Now, they have entrusted this responsibility to us headmen. They said that “you headmen, if anyone in your village don’t build a toilet; we are going to give punishment to you” *(repeating sentiment)*

M5: In fact they discovered; In fact they found that a toilet is very useful indeed......

M1: Anyone who has a house needs to have a toilet

M5: Even a minister, he can be going from there, and he has felt that he needs to defecate, the minister would stop his vehicle dramatically and he would approach the house. We would mistakenly think that he wants some water to drink, but he would proceed firstly to ask “what about......what about....”

He uses symbolism crudely interpreted as “he has felt that the big man needs to come out

M3, M1: A house of laws

M5: We would say “ooh you can go there, gentleman”. So after coming from there, then he would ask for some water to be poured on his hands to wash them. He would say “Please, I beg of you, that’s the reason I came”. Later, there he would go. Having a toilet gives one high sense of respect and dignity

M1: You would have assisted your friends because you have a toilet

M: What problems do you encounter in bush open air defecation? *(Repeating question immediately)*

M2: There is a big problem; there is no freedom in defecating in the bush, fearing that perhaps my friends will find me; it is better if you have met your fellow man. Now if it is a woman who has found you there, there is a fuss

*Laughter*

M2: She would say “I found the father of so and so”

*More laughter*

R: Headman

M2: “I found the father of so and so”. I found him squatting and the way he ran away *(demonstrating the running away)* and cutting firewood on his way

*Laughter*

M5: Even running into buffalo beans. Meanwhile the big man is requesting to be dropped *(symbolising the urge to still defecate)*

Buffalo beans = the pods of this wild beans are very itchy when they come into contact with skin

M: Others? The badness of toilets.....Yes? *(Addressing M7)*

M7: The other bad thing about using the bush as toilet is that when I have gone there and have started to relieve myself, you find that flies are everywhere and even sitting on your body. Sometimes forgetting to or not knowing that this where a fly sat, you rub your hand on the site. After moving about you buy a banana even without touching the thing inside you start eating it. That is a mistake

M: Very well, very well! Is there anyone who can add on concerning the badness of the using the bush?

M4: In fact at the bush you cannot be relieved well or fully because you relieve yourself with fear; maybe there can be someone who can find me; even sitting in the bush you are glancing around. It is not needed to be glancing around; you need to be fully relieved, with enough freedom. In so doing, it means you would have managed.

M: Good, yes? *(Addressing M6)*

M6: The way it has been spoken off already, 1) the badness of the using the bush is that it brings about diseases, secondly, no dignity or respect of using the bush; There is completely no respect; sometimes, you may think to yourself that where I am going is where it is bushy and overgrown and I can go and use that place. You find that at that same place, there is already a person. And also....

M1: Maybe it is relative

M4 laughs

M6: Another problem that is there is that sometimes you are in the middle of the village, and you have suffered from diarrhoea, now for you to travel and find a place where it is secluded and overgrown, it means by then you would have soiled yourself. So with all that, it means respect is lost

If they know you, they would say “he has soiled himself”

M5: They would say he has put on stockings; meanwhile their friend has ....hahahahahahah

M1: It is the abdomen of a toad

M: I saw a hand up there

M8: Yes; the badness of using the bush is that, for instance the way the bush is burned nowadays, there is nowhere to hide because many bushes have been burned down

M1: Leaves have dropped, too

M8: Leaves have dropped. Trees have no leaves, now there is nowhere you can hide

M: Hmmm, hmmmm

M8: Even when a person is passing on a road, he/she is supposed to see you; that is what I can say about the badness of using the bush

M1: That there is someone over there

M5: You would need to look for a big tree, holding on to it *(demonstrating a big tree trunk)* and you are ready then, like this *(demonstrating hiding behind the tree trunk).* It does not show respect

*A few smile and laugh softly*

M: So that has no respect?

All: No

M5: It doesn’t have respect

M: Very well, what about the washing of hands? How many times do we wash our hands? When do we wash our hands?

M3: Many times washing of hands for us here in the village, we don’t use it; we just come from there and upon return you easily forget. It is because there is no toilet. For where you have come, from a distance away and is it is far, and by the time you reach home...in fact on the way you can stand and chat with your friend such that by the time you reach home you even forget and ask yourself that “where have I come from”? With that you end up staying like that, three-quarters of us use that system

M: You have explained that from coming from the toilet, some do not wash their hands. Now, what about children?

M1: When children have used the toilet?

M: Yes

M3: Even children do not wash their hands

M: Hmmm. Hmmmm!!!

M3: It is me who is destroying those children now. They know that there is no forcing that “you.....

M1: They see what you do when you come from the toilet

M3: ...wash your hands, wash your hands”; it is not there because I myself am not doing it

M: Uhm?

M3: Even the child notices such, that when it comes from there, it also proceeds to go and play within the yard. Even for you to tell the child that “wash your hands” is not there. So that is what happens in our villages for sure

M: Are there others who may add on washing of hands?

M1: Washing of hands in the village...when going in the toilet, you have something to use when wiping yourself. Now if you have wiped yourself, there is no need to remember and say “Wait, let me wash your hands”. In fact, where you wiping yourself with hands? *(Making a gesture of the same)*

*Loud and long laughter*

M6: On the part of washing hands; it is the same, the health personnel talk about it. They say that as long as you have entered that toilet,

M1: You need to wash your hands

M6: When you come out of there, it means you have come out with germs on your hands, you cannot see them, as they themselves say; we cannot see them with our eyes. So when you have gone in a toilet and have come out, it means that you have contracted those germs from there. That is why they tell us that, every time when you have come from and before you touch anything, wash your hands so that those germs should be removed, especially that in so doing we use soap. So, reluctance or laziness of washing hands is the same as destroying our lives

M: Hmmm, hmmmm. So you have explained that washing of hands does not take place?

A few: Yes

M: So the outcome of such, the outcome for children who have been raised in such a community, do you think at a household or the community we live in, can it be a good life?

All: No

M4: It is a bad life

M1: It becomes a difficult thing. It is like harbouring diseases. Illnesses do not get eradicated

M: Very well, before eating *nshima* what is the habit? There is sugarcane, there are sweet potatoes. From coming from the field, there he goes to eat sugarcanes. How do you compare it, sweet potatoes and *nshima* in terms of washing of hands? When do you properly wash your hands.......?

M4, M6: At *nshima*

M6: But when eating sugar cane, sometimes bananas and others.....

M1: You just get and eat them

M3: Just eating them

M6: Only at eating *nshima* that is when you know that you need to wash your hands

M: Only at eating *nshima*?

Most: Only at *nshima*

M6: Yes; even with porridge, if one has a spoon you cannot tell that person to go and wash his/her hands. They will just get a spoon and eat

M1: In fact, a spoon is there for scooping the food

M: Why is that so?

M3: It is because we do not know; we are not knowledgeable even when we are told by what he is talking about *(meaning M6)* because it is not all of us who go to meetings. Some of us just stay at home. So what has happened to our colleagues there, even if they brought the issues to us, just what the headman has also explained, you look at it as if they are just troubling you. When they explain you easily forget about it. The main reason is that we do not know

M: Hmmm, hmmmmm.

M3: Yes

M: What do you think would be the benefit of washing hands?

M5: A kind of life that can exist when not washing hands, I think, it is that there wouldn’t be a good life; because what comes out of there is malaria, malaria come from there, TB comes from there, because where I have gone and upon my return I just pick up food to eat without knowing that what has attached to my hands is a TB germ or a malaria germ that is attached there, I do not know. Like that, it would mean I have contracted malaria and taken to children at home; if it is TB, I have also contracted it there and when eating with a children without washing my hands, or even I have given a piece of a sugarcane to a child when I am eating it, meanwhile I have come from using the toilet, and if I am giving a piece of sugarcane to a child, it means I would have given malaria or TB to that child just because I did not wash my hands

M: Are there others who can add on?

A door squeaks as someone is leaving or entering. This slightly distracts the group

M4: It is good to wash hands because you protect diseases yourself. After coming from the toilet, and you have washed your hands, you can eat anything that you would like to eat. There is nothing that you can fear

M1: Washing of hands is a good thing indeed. For example, while sitted a fly may land on you and you may swat and kill it as you squash it. Now if you have not washed your hands, you would have harboured diseases. By washing hands.... from where you have come, you are required to wash your hands so that you look good

M: Very well, anyone else who can add on concerning the goodness of washing hands?

I have seen a hand

M6: Another good thing, the way we have spoken before, illnesses lessen at the household

M: So what are the benefits or goodness of washing of fresh vegetables? The hygiene and caring of vegetables!

M1: The benefits...It is the women who prepare and cook the relish. For us who observe, when they pluck vegetables from the fields, they immerse and wash them with water in a dish. They shake the leaves before they meticulously remove the inedible bits followed by cutting them. That shows that they have well taken care here

M: They would have taken care there?

M1: Yes

M: Does it happen to every vegetable in the village that it has diseases?

M3: Yes, disease can be found. Number 1) especially if the vegetable is rape; in most instances because of lack of fertiliser we use faeces from chickens, goats and place them in the vegetables. Already with those things, flies cannot miss. So, the benefits of vegetables are that when they have been brought from the garden, they need to be washed first before being put in the pot. This is because diseases can be contracted because of those things at the garden; the vegetables take up matter from the chicken droppings, or perhaps from goats. So, diseases can enter into those vegetables. That is the benefit of washing vegetables.

M: Yes, another one?

M5: I believe that a live thing needs to have diseases. So, as a rape needs to be cultivated, it needs to have diseases. Three-quarters when women have plucked vegetables from the field, they may not know that I may have gone there to defecate, so when they have plucked those vegetables if they won’t wash them properly, so that the vegetables are happy with it, they will not know that maybe a child left stool on it in the field. If they have just carelessly washed them, it means those diseases which a person left there have still remained on the vegetables that we want to eat. However, they must clean the vegetables properly so that they are clean before they are cooked.

M: Yes? *(Addressing M6)*

M6: Another thing, in order that those vegetables grow, the one who grow in gardens also spray poisons on them to protect them against those germs. So, if you have just bought, cut and put them in a pot, you may not know the time lapse from spraying to cooking. Sometimes that person may have no money, and decides to sell the vegetables. Poison may still remain on the leaves and so when you wash them it becomes better.

M: Which other problem do you think may be encountered when utensils are not well looked after?

*Silence*

M: We have moved from vegetables, and now to utensils used for eating or eating from. What are the difficulties if they are not well looked after?

M3: The main difficult if utensils are not well taken care of means that those things also bring diseases, considering what we have been learning here. They also bring diseases, one: we may say and claim that we manage to do it but if you do not take care of the utensils then problems still come as those things are not well looked after. That is the difficult we may encounter.

M: Very well, M2? *(Addressed was headman)*

M2: Many times, it is the women. The women are the ones who care for things, now if the women have not good care of those things, even malaria is rife, occurring frequently because the women may not take good care of the utensils, the manner of washing; Some wash the utensils when they have already set a pot on the fire...... Even the way used/dirty water is disposed off, they may just pour it on the ground within the yard

M1: I however see some women who things very well. Before they have set the pot on fire, they start off with washing those utensils and put them to dry. After doing that, then they set the pot on fire. After the food is ready that is when she puts food in the dried utensils. So after you have finished eating, she goes to wash them again so that they are put away. I then say, this one is good because she takes good care of the utensils. Not a kind who after eating just leaves them, putting them aside like that, that is not caring at all

M: I have seen a hand over there

M6: Yes; the difficult that is there if they are not taken good care of plates or maybe pots. One, it is flies. That thing wants to look for food as well, so when they enter the house they see those plates and pots. You will see that they be full in there; sometimes they will some other food that has been put there. If that is open, the flies would be coming here and going there, one place to another, meaning that that person who is going to eat that food will start becoming ill

M1: Will start to be unwell

M: Very well. Do we all know what hygiene is?

All: Yes

M: So, what does body hygiene translate to? What does body hygiene mean? What does it mean in relation to our lives in the village?

What are the benefits of good body hygiene?

M1: The benefits of caring for your body mean taking good care of your body. Not that every day I am unkempt. One needs to care for life so that frequently reminding oneself that “let me go and bathe now”

M: Others?

M5: I believe that body hygiene is actually or synonymous to bathing of the body. When you bathe and you are done, you also need to get clean clothes to wear. Not after bathing, you get back in the dirty clothes, it means you have brought upon your body that ....you demonstrate that you have not bathed, if it means getting sick, you have to get sick. After bathing and getting back dirty clothes means that you have punished your own body. What you need to do is get that one that is clean and allows air to flow through properly so that the body is happy

M: Yes, yes, yes; do you know the disease epilepsy?

A few: Yes

M: Are there people suffering from this disease there in the village? Have you seen this disease in some people?

M1: The disease of epilepsy is there in the village

M: What kind of difficulty comes with the disease of epilepsy in a person?

M3: The difficulty of epilepsy is that a person begins to appear like a mentally disturbed person, or coming to the point of being mad; one becomes a person who doesn’t do a straightforward thing, a confused thing. Now you on your own may think he or she is doing it intentionally and maliciously. An epileptic person does not have straight forward things.....he/she becomes a mad person or a fool

M: Where does the disease of epilepsy actually come from?

M1: The disease of epilepsy! Even we get surprised by it. You just see that someone is well one moment and the next he or she falls to the ground and starts to fit like this (demonstrates), and foams at the mouth. This is followed by being still and shortly after, he or she awakens. Now what is perplexing is that what kind of disease is this? How is it? That is what has made me to come here, it confuses me

M: Yes, my brother? *(Addressing M6)*

M6: The disease of epilepsy especially in the villages when a person has started to have fits, 1) what comes is the thought that they have bewitched my child, maybe it is my mother or father that has bewitched. That is the first part that is there. Secondly, we hear that the health people say that if it is a child or an adult that has suffered from malaria and now that malaria has become bigger or worse in his/her body, it means when that person’s body has become very hot, that person can fit. I personally know of those two scenarios.

M: Yes? *(Addressing M7)*

M7: The part I wanted to say has been explained for me by M6

M: So, concerning this disease of epilepsy, what prevention can be put so that it is treated? What kind can there be?

M3: For this disease of epilepsy to be treated you have to teach us

M: Very well!

M3: Yes; Left on our won, it is lost upon us, we may dig...in our village we use root herbs. But to see that an epileptic patient has become well is not there.

So, here where we have come, they may teach us

M: Can this disease be cured or the medicines you said are dug, where people getting cured or were they not? Can treatment be found?

M1: Some other medicines are found;

M3: There two different types of epilepsy; there is a real epilepsy. This one is not cured by plant roots

M: Hmmm

M3: There is one they themselves call *Kanjive*. We call it *Kanjive* in the village. That is the one that three-quarters of the medicines are looked for in the villages and they are found. Isn’t it so gentleman *(looking at M5)*

M5: It is true

M3: Not epilepsy. They need to explain, inform us here, we may not know it

M5: Then when talking about epilepsy, we may say in the middle part, it is a thing that, even in the village we may attempt to look for this and that medicine because when a child starts to fit starting from morning until the whole day (sunset)...

M1: Without eating

M5: Without eating, until night fall. Even for you who may look for medicines gets confused. You may contemplate that what kind of medicine can I give to this child who does not stop fitting.

M: Hmmm

M5: It means things there have now become very difficult. You the one who can look for medicines and the ones giving care to the patient can get your thoughts confused. What remains now is to ferry the patient and bring to the lady *(looking at Nurse Ruth)* and say look at this person. Even when she comes to see the patient herself, she finds that we have heavily tattooed the patient’s body. She would retort that “What you have done is assault. Why have you done this to this child?” We would respond that “We were looking for a way to treat our child”. Now, you know our friends here *(at the clinic)* who know these things very well; that is when they inject the person until she/he sleeps (sedated). And you say “now my child has now become better”.

So even after that person has woken up, he/she still remains mentally confused. Even we get confused. So it is only you who have come who can teach us concrete things, you should tell us concrete things which we may place in our heads

M1: So that we know very well

M: Very well, very well. If vaccination for pigs has been found, can you agree that pigs get vaccinated freely without any hindrance?

All: Yes

M5: that would be a joyous thing

M1: It means protecting, when that thing is given, it we who eat that pig. When they have given it medicine, we also may become well off.

M: Would there be any difficulty?

A few: No

M3: There would be no difficulty because 1) pigs do not have a good life; of all things that we have kept, pigs do not have a good life. If I buy a pig from this one *(pointing at M2)*, for those things are very expensive,

M: Hmmmm

M3: I will spend on him *(M2)* about K150, 000 or K2000, 000 but even after a week it may need to get sick right there and then

M: Hmmmm

M3: Now, you just remain complaining that my money? Even if it is a sick one, I would say “let me just slaughter this pig and sell pork to people in the village to recoup my money” because they is no protection of any kind.

So, if protection of pigs has come, we would be very happy

M: Do you know that boiling may kill some germs in the meat?

A few: Yes

M: Yes, I had seen a hand over there?

M5: I wanted to say something concerning vaccines, the issue of vaccination of pigs; it may be a very good thing so that at least even pigs should have medicines in their bodies so that many diseases in their bodies should be removed

M: Very well

M5: Because three-quarters, we may find even cysts may not be found anymore

M1: They are not there!

M: Hmmm; hmmm

M5: We can be happy if vaccination of pigs had to come, thank you

Mwanza summarized at 14:30h

M: Now I have reached here, I believe we shared wisdom on the issues we have discussed. We talked about the benefits of keeping pigs, the goodness of pigs, as well as the dangers found in pigs, where we keep our pigs, pigs that do not have kraals, and the challenges or badness of pigs confined in the kraals-the feeding, and in conclusion we talked about prevention that can be found like vaccination of pigs, having good toilets, good hygiene, not so?

A few: Yes

M: The things that can prevent the diseases of intestinal worms or disease of tapeworm on issues of hygiene, and the keeping of pigs.

Now that I have reached here, I am thanking you very much especially on any part that you have contributed. I am very grateful, thank you very much

All: Thank you, thank you *(as they clap their hands)*

**The End**
