# Supplementary material for: Why Latrines Are Not Used: Communities’ Perceptions and Practices Regarding Latrines in a Taenia solium Endemic Rural Area in Eastern Zambia
Source: PLoS Negl Trop Dis. 2015 Mar 4;9(3):e0003570. doi: 10.1371/journal.pntd.0003570 (PMC4352092; doi:10.1371/journal.pntd.0003570)
Supplement: S1 Dataset — (ZIP) [file pntd.0003570.s001.zip › FGD transcriptions_Zambia-2010/Wonzi_Dr Phiri/Women_Wonzi_30-07-10.doc]

**Title : Focus Group WOMEN**

**Date : 3**0/07/10

**Site :** Kakiwa Rural Health Center

**Village :** WONZI	**Location :** Petauke district, Eastern Province, Zambia

**Duration (total time) :**	55 min (in 2 parts)	**- Start :**	11:51 am	**- End :** 12:46 pm

**Participants:** 8 women

**Informed consent:** signed and available

**Moderator :** Ms Ruth Chiwa	**Recorder :** Dr Andrew Phiri

**Transcription/translation :** Dr Andrew Phiri

**Abreviations :**

**M : moderator, R : recorder, I: intervener, C : children, W : women, M : men**

**Disposition (from the left of the Moderator):**

**M**

W1

**R**

W2

W3

W4

W5

W6

W7

W8

Age composition:

W8: the oldest

W1-W5: young

Typology:

W8: expert (helping at the center), dominant

W7: rambler

W3: very shy

W4 –W6: very quiet

W2: Funny/ Slight/dominant

**Comments about participants :**

**Group dynamics :**

**Text:**

The informed consent process was done with participants being videotaped.

This was followed by introductions of the research team

*Introduction*:

M: How have you woken up ladies?

All women: We have woken up well

M: To begin with, we need to know each other and to greet each other. I will first start.

She introduces herself and every respondent does the same

M: We now need to be free, expressing ourselves freely. We need to educate each other, exchanging ideas in the way you yourselves know especially concerning the pigs that we keep in our village.

They have already indicated that they are also taking a video, not so?

A few: Yes

M: This video is not going to be taken far. It will be taken to Lusaka where they will view what we would have discussed here. After that it will be destroyed. Our pictures won’t go to Europe, not so?

A few: Yes

M: So, we are going to start with the issue of pigs in our village. Do we think that pigs are necessarily useful in our village?

Most women: Yes

M: Why do we think like that?

W5: A pig is helpful at many things

M: In which ways? We need to explain properly

W7: A pig helps during funerals

W3: Pigs help in weddings

W4: Pigs are helpful when children have no clothes, and you sell some to buy them clothes

W2: Pigs are helpful in times like when a pupil has passed exams. You take that livestock and sell it and use the proceeds to take the child to school

W6: Pigs help...If I have a sick child and I have no money, I take a pig, sell it and use the money to take the child to the hospital

W8: Pigs help with issues of agriculture. If I have been troubled with such issues and need to hire agricultural help, I can slaughter and use a pig to pay for weeding

M: What about....A pig has its goodness or benefits?

All women: Yes

M: What is the goodness of pigs?

W1: There are many

W8: What we have just explained are the good things about them. Like using money proceeds to support school-going children by buying school uniforms for example; at agricultural fields as well, eating at funerals, after we have slaughtered it people can consume it, during celebrations or feasts, people eat such meat

W2: We get helped like that

M: What about the badness of pigs?

W2: The badness of pigs is that when that thing has become sick and we get it, slaughter it, that clearly already has diseases and we eat the diseased meat. That is a mistake because it is diseased

W1: Another thing is that pigs eat a lot of things; it goes to the grave yard and exhume things, and while we eat we don t know what is happening

W8: The badness is what we already have spoken of is that yes we eat it but what it eats itself are many things. It eats indiscriminately. Even human faeces that have been deposited in the bush it goes and eats them, even a stillborn baby or newly born baby that has died, it can go and exhume it from its grave and eat it. We don’t know the kind of meat that we eat and how it is affected because of what the pig eats; it doesn’t choose what it eats

M: Is there anyone who can add on?

W5: We don’t have ways of protecting those pigs. We don’t have them! We do not even have people to examine them here. What we are asking for are doctors to look and exam these animals; if they have diseases we can discard them, if they are well we can get and eat

M: What are the benefits of pigs?

W8: Pigs! Pigs have no use; they don’t help in ploughing for instance

W6: It just roams about

W8: It just roams about and has no use at all. We keep it just to be eaten. It does not plough at all

W4: The only time it is useful is when you have encountered trouble, like weeds in a field. That is when it is useful

W2: That is when it assists us

W8: But on its own it is of no use. It doesn’t plough at all

Other women agree

M: What about the dangers?

W8: Those are some of the dangers. Those unknown diseases it has resulting from what it eats. We do not know what happens in our bodies and what results when we eat such pork since it does not get inspected or examined.

M: Do you also rear other types of livestock in your families?

A few: We keep

W7: But as for pigs they multiply faster as compared to goats. We get helped with pigs

M: What kind of animals do we keep on your own as women?

W5: Cattle

W8: Chickens

W1: Goats

W2: Ducks

M: Your very own?

Women: Yes

M: Why?

W1: To be helped

W8: Similarly to the reason we mentioned for pigs, like in times of funerals, during celebrations, at weddings we can slaughter some to be used for food

W2: Even at initiation ceremonies because others may not eat pork, so may prefer goat meat. That is how we get assisted

M: Do you take part yourselves in keeping pigs?

Some Women: Yes, we take part

M: What type of duties do you perform?

W6: Feeding them, that is one duty

W7: Securing them at night

W3: Caring for them

W8: Taking care of them; like feeding them maize bran in the evenings. That is supplementing to what they eat during the day

M: What kind of foods do they eat during the day?

W5: Grass, human faeces

*Laughter*

W8: Faeces, especially when some people who do not have toilets and go and use the bush. You find it has followed the person and after that person has defecated, it eats

Other women: It eats

W2: So at home without knowing that it has eaten something bad, you get and slaughter it

W8: Even after eating such matters, it can come back home and eat from household utensils like plates; when plates have not been well secured you find it would have eaten from them; it eats anything, it can eat sugarcane peels, pumpkins, it doesn’t choose what it eats, it eats anything

M: Who else assists you in keeping pigs?

W5: There is no one

M: You women do all things by yourselves?

A few women: Yes

W2: Husbands are also there

W4: When men have bought them, they are the ones who care for them a lot

W8: Not men, they do not particularly pay much attention. There are a few of them

W2: Men only take care when pigs sleep outside and have to build a kraal for them

M: What about those people outside the family?

W7: They do not take part

W8: As a matter of fact, they (i.e. pigs) are not theirs. They also rear theirs

M: What about who makes decisions, like “let us slaughter this pig” or “let us sell this one”. Who is it?

W5: When a woman has suffered or has encountered some difficulties, she needs to discuss it with her husband. She would say “I have these problems here at home, why don’t we get that pig and slaughter it? Women can lobby with their husbands

W2: That’s when a husband can agree that “let us slaughter it”. So, together with a husband a decision is reached because that thing belongs to both of us. However, sometimes children can propose, too; they can say “mother, let us slaughter a pig, relish has been very difficult”. Like that a child can rule depending on the ideas and thinking of that child and you as a parent can allow having the thing slaughtered

M: What about the general welfare of the pig, to see that pigs are fine, who oversees such?

W5, W7: It is a woman

W1: A woman feeds them maize bran

W4: Yes

M: Who feeds pigs?

All women: It is a woman

M: Who fees pigs? It may be different in various families

W7: As for me, even children feed them

W2: You can assign such a duty to a child to feed them and you as an adult (mother) supervises. You ask if the pigs have been fed, if not you request the child to do it.

W4: If a mother doesn’t supervise, children may be relaxed and not do it

M: What about for others? Who feed the pigs?

W6, W8: Ourselves

W4: If there is no child at home, it is yourself that feeds

M: Why do you have to feed yourselves? Is there a particular reason why that child or yourselves should the ones who feed the pigs?

W4: Maybe a child does not pay attention to the feeding when she/he is sent to do it. If you don’t pay attention you as an adult, it may mean the pig can go hungry

W2: Another thing, if your “mphumi” (crudely translated to knack-a specific talent for something, especially one difficult to explain or teach- for raising particular good qualities or numbers of things) only allows to breed only males, you change “mphumi” so that pigs can breed females as well. Similarly, if pigs only breed females and you want them to change to breed some males also. In that case, you let the child do the duty to see his/her kind of talent *(mphumi)*

W7: As for me, they do not give birth or breed until I give a child to rear

M: How many times do we feed these pigs?

A few women: Three times a day

M: What times do you feed them?

A few women: Mornings, afternoons and evenings

M: What about those pigs that eat faeces? What goodness do you think is there about pigs eating faeces?

W8: There is no goodness about eating faeces

A few other women: There is no goodness

W8: It is just that we suffer and have nowhere to keep them. Faeces are not god for eating. What is needed, however, is that a kraal is built for them and the pigs are confined and fed within the enclosure. We don’t manage and therefore fail to keep them; our husbands fail to build proper kraals such that some pigs break out of kraals and go to eat faeces. Faeces are not good to be eaten.

W2: Sometimes, when you confine pigs in a kraal, you build a kraal and place them there and you feed them but there is a difference between that pig that is in kraal and the one that is ...

A few women: ...... that is outside

W2: ...... the one that is outside. The one that is outside may be found to be.....

A few women: ... to be getting fatter

W2: ....is getting fatter while the confined one is getting thinner, but you feed them in the mornings, afternoons and evenings. Sometimes previously-served feed that was not eaten is found as you place the next fresh feed for it. It is just found to be getting thin

M: What is the badness or danger of pigs eating human faeces?

*Silence*

W1: Faeces are not good to eat as we also feed on the pigs

M: What about on the health and life of the pig itself in respect of it eating faeces?

W5: That thing is a created thing. It was created to eat faeces, to eat things that are acceptable to it. That is its nature. There is nothing that we can do because it eats them

*Laughter*

M: What about the health and life of us humans who eat the pig? A pig eats faeces....

W8: And we eat it

M: What do we think about it?

W7: What makes us eat pigs; I personally see it as just a craving for meat (“*mandu*”)

*Laughter accompanied by animated exchanges*

W4: They get assisted who eat it

W8: Poverty, I guess

W2: That is a kind of meat that they think is easier and more manageable to slaughter and consume. When you compare a pig with cattle, it is easier and reasonable to slaughter a pig because of its size

W8: The other good thing about a pig is that it can conceive and from the resulting pregnancy it can give birth to 8 piglets.

W5: 12!

W8: 12 piglets. That is why we think pigs are better because they propagate and increase in number quickly. They are better than cattle or goats. Goats only give birth to one or two kids. A pig gives birth to more young

M: Do we frequently consume pork?

*Momentary silence followed by women speaking at once*

W2: Yes, it is different when compared to goat meat or beef. For beef we last ate it when there was a disease outbreak. There is currently no one who can suggest that we slaughter cattle for beef. There is no one

W5: We eat pork often. You can eat today, tomorrow and the subsequent day

M: During what times or events do you frequently eat pork?

W2: From this month (i.e. July) up to Christmas time. After Christmas and New Year, consumption is limited.

Other women agree

M: If there are people selling pork, they come to sell it, what do we see that makes us buy that meat?

W2, W3: It is the fat

W8: You see the good look of the fat, and generally how the appearance of the pork is like; it looks whitish

W2: How the meat appears

W7: It actually does not have a taint/smell. It differs with that of goats in that goat meat has a bad smell. It is true, pork does not a smell!

W8: Lack of fat

W2: Can’t buy pork without fat

M: What do we see that prevents us from buying the meat?

W2: There are differences that make us not buy the meat. Sometimes, the pork is from a pig that died of a disease and therefore it has a taint

W3: It could have died of a disease

W5: It may have cysts

W2: It may be having cysts

W8: When the meat is reddened

W4: It may not have fat, looks dull

W2: Yes, it may just be dull. Like that you cannot purchase it

M: Does a pig get examined or inspected when being slaughtered?

All women: It is not inspected

W8: We just eat it

M: Why not?

W5: There is no doctor to inspect

M: Would you like for the meat to be inspected?

All: Yes, we can like that

M: Why is that?

W2, W5, W6: To know whether it is diseased or not

W8: Because that thing eats bad or detestable things

W2: Different things

W8: It eats indiscriminately; maybe the meat may have diseases and those diseases in the meat can be transmitted to us humans

W2: From time to time we may be complaining of various diseases. We may at short intervals complain of malaria, you know when that thing is unwell it does not lift its head. It has its head lowered down

*Pause*

W2: It does not even wag its tail and you know that this thing is unwell. When we see that, it is the same pig that we slaughter and eat. Shortly after, you complain of malaria. Maybe malaria came from eating that meat that was diseased.

M: What about preparation of meat, how do we do it?

W7: Getting it, slicing it into small pieces and washing it first before cooking in a pot. When water has finished, we then fry it. When you don’t wash it, there is a danger that maybe flies would settle on it and these same flies could have come from settling on stool especially of a child. It is also a problem if you have not washed the meat. Therefore, there is need to wash first before puting the meat on the fire.

W2: After boiling it must be fried. When it is ready, you would have added tomatoes or onions. When the meat is finally ready, it must be covered with a lid, and put away in a safe place after. That is when you start to prepare *nshima* and you eat afterwards

M: Is there any other way in which we prepare pork before consumption?

W8: Roasting, just roasting it

W1: If the meat is bad, it can be boiled briefly, grilled “*ubambika*” and kept to be eaten later

M: Any other forms of preparation?

*Silence*

M: Is there another way of preparing the meat from the animal we would have slaughtered?

W7: It is what we have already mentioned

Others agree

W8: Cooking, boiling and added necessary things like tomatoes. Another one is briefly boiled it/steaming it and drying it

W6: Grilling it

W8: Grilling it

M: Why do you grill it?

W8: When the meat is a lot and we fear that it may spoil or rot.

W7: Sometimes, those pigs that are unwell and when one have died they get it. It does not taste good if cooked like that when it is fresh but when you have washed it, boil it briefly and grilling it before eating.

M: Is there a time when we eat pork in a different way?

*Silence*

W2: Not at all

M: Like at a funeral?

Simultaneous discussion by participants

W8: At a funeral, they slaughter it, boiling it with only salt added to it. They boil and fry it, sometimes even without adding tomatoes

W7: They do not even fry it but only add curry

M: What about cysts? Do we know what cysts are?

Women: Yes

M: Have you seen them before?

Women: Yes

M: What are your thoughts on them?

W1: For meat with cysts to be good, what is needed is to boil it first and grilling. That way the cysts are minimised or removed

W5: They pop out

W1: The meat should first be dried before cooking. The cysts are minimal and after grilling it, it can be cooked. That it is comparably good and tastes better

M: What do others think about cysts that are found in pork?

W3: Others get that meat and throw it away

M: Why do they throw it away?

W3: Because it has cysts

W2: They say that it is because it has diseases

W7: It has diseases

W8: Even when pork has those cysts, those things that look white and are small, when you get that pork it has a distinct smell. It is not pleasing to eat such. That is why we like to boil it first and grilling it later until it is dry before cooking it. But for the young men who sell *kajinya* or shokas, those who sell pork by the roadside, they just boil it briefly and eat it like that

W2: They do not wash the meat. They just slice it and place it on fire and fry it. Now when you buy it you just go on eating it but the meat was not washed or cleaned

M: What about you, have you eaten meat with cysts before?

W1, W2, W3, W4: We have eaten pork with cysts before

W2: And we found out that it is not good meat. Now we have thoughts or ideas that when a pig has been slaughtered at home or you have found meat with cysts you get it and boil it and grill it

W1: Grilling it

W2: Because it does not smell good, it has a taint

W8: It has a bad smell or taint. It looks reddened

W4: In fact, the cysts in the meat get shattered or rupture in the mouth when you are eating. You can hear the sound as they pops up when eating *(miming a sound of popping)*

W8: Even in a pot when cooking, you can hear the cysts popping *(demonstrating)*

M: Toilets! Do we utilise toilets in our village?

All women: Yes

M: Are they there?

A few women: They are there

Simultaneous response: Others do not have

W8: Some of us do not have toilets. When going to the bush, it (meaning a pig) follows you behind

*Laughter*

W8: It properly follows you from behind. It even hides from you and when it notices you are about to stand from a squatting position it appears to come and eat the faeces

W5: Others even knock you off when you are in the process of defecating. When you are looking in another direction, it could be coming from the opposite direction

*Laughter*

M: So, how do we look at the issue of toilets?

*Pause*

M: Is it a good thing to have a toilet or not?

All: It is good to have toilets

W8: It is a good thing to have a toilet

M: Why is good to have a toilet?

W8: Because there would be benefit or a good reason for keeping those livestock well. It would help us to protect them by having toilets so that they would avoid eating those faeces

W7: The problem you yourself can offer prevention by building a toilet at your household, but others may not do so

W6: The neighbours would not have them

W7: So the pigs will move from your place to go and eat from there

M: What about other family members?

*Pause*

M: Where do they go in terms of toilet issue?

All: They go in the bush

M: In the same household, some use the toilet and others go to the bush?

W2: Some go in the same toilet you have built

W4: Sometimes, children fear going in to the toilets

W6: They go in the bush

W5: For some when they enter the toilet, the urge to defecate goes but the urge is not troubled when they feel and use the bush

W7: At a household there may be small and big children. The smaller ones fear falling in the pit latrine (toilet)

W8: Others just refuse using the toilet; they say that “I don’t want to use the toilet because the urge goes when I go there; I prefer to go in the bush because I am used to doing so”. For some they do not even build toilets, they would rather remain like that

W2/W6: If someone is using the toilet when you wish to use it, you instead decide to go in the bush

M: What about the men?

W7: Men go to the bush with an axe, if they do not have a toilet. Afterwards, they just come back with firewood

W2: Meanwhile he has come from toileting in the bush

*Laughter*

M: What about the health and hygiene? Who within the family bathes children?

All: It is the woman

M: A woman?

All: Yes

M: Does it mean all of us are the ones who bathe the children?

All: Yes

M: Is there no one else who assists us?

W5: Older children can bathe their younger siblings

W2: In fact it must be those that are bright and can do it

Other women agree

W2: Women, can also assign young girls to bathe younger siblings. The danger sometimes is that when you request them to do it they retort and say “but even yesterday, it is myself who bathed them!” and therefore refuse to do so. Therefore, you yourself have to get the child and bathe her/him

M: Do you think it is necessary to bathe children or not?

All: It is necessary

M: Why is it necessary?

W3: There is need for people to be clean

W2: They need to be clean. You know humans have pores on their skins and if they are blocked you find a child complains that he/she is not feeling well. When you bathe the child you find that the child relaxes and feels free and well

M: Do you tell your children to wash their hands when they have come from the toilet?

*Silence*

W1: There are few. Most children when they come from the toilet quickly run to go back to play

W7: Others do no wash at all

W6: For some when they have come from the toilet they even rush to get food and rush off quickly

W4: ... and eat it

M: What about at eating, do you ask them to wash their hands?

All: Yes

M: Do they wash their hands?

All: Yes, they wash

M: Why do they wash their hands?

W5, W6: To remove dirt and to become clean

M: I have heard here that children when they have come from the bush just proceed to get food and eat

W3: No

W1: But for some it is true indeed, they come in straight from the bush and pick a cob of fresh maize from a pot and run away

W6: Even for sweet potatoes

W1: They do that except for times of eating *nshima*

W2: Before eating *nshima*, they make sure that they wash their hands; even for small children, they would ask to be allowed to wash their hands even though for sweet potatoes he/she may have gotten them without washing hands

M: Have we ever heard of intestinal worm disease?

All: Yes

M: Have you seen them before?

Most women: Yes

M: Did we see them in faeces of children or adults?

W5, W4: In a child’s stool

W7: I saw them in my child, after I bought dewormers and dewormed , the child defecated them, big ones!

M: Others who have seen them before?

W5: I have seen them before in a child I looked after. What I used to notice was that when that child ate food, it used to vomit and have dark diarrhoeic stool. After the child was given the drugs, it defecated big worms.

Interjection by Andrew: Did the child defecate outside?

W5: Yes, I wanted to check and verify as previously the child used to defecate dark stool

W3: I had some worms myself

*Suppressed laughter*

W3: I had them

M: Tell us about it then

W3: I defecated them after I bought and drank dewormers. I went in the bush to remove them

M: When someone has intestinal worms, what do you think are the symptoms? How would we know that this person has intestinal worms?

W7: For some children, they eat too much. Even after coming from recently eating something, they would eat again if offered food. Even the abdomen is enlarged and you would think that “yes, this child has worms”

M: What about others?

W5: The cheeks are usually shiny. The child eats a lot and even after condemning her/him, he/she goes on as if he/she does not understand you.

W4: Even the abdomen does not get smaller or deflated

W5: It does not get deflated

W4: Even after the child has been to the toilet, the abdomen remains swollen

W5: They even cry out because of abdominal pain

W8: For others, they have poor looking hair. The hair does not grow properly. They may even have ringworm on their heads, and have poor appetite

W2: Others say they feel an itching sensation at the throat and proceed to vomit afterwards. They may try to clear the throat to no avail. After touching the back of the throat, they would vomit a worm. You would see it like that and it would be wriggling

M: Do we think we can prevent this disease and if we can prevent it in which ways can we do it?

*Silence*

M: Intestinal worm disease! Do we think we can prevent it?

W1, W2: We may not know how to prevent them

W7: We can prevent with medicines. Like I have said before, we can prevent them. Even with the type of food we eat, we need to be careful

W1: We do not know how this disease comes about

W7: Others say it is from the same soil. Some children indeed eat soil. My child used to eat a lot of soil

W4: Even for others who do not eat soil can also have disease

W1: Yes, even for those who don’t eat soil!

M: We do not know how the worms come about?

Most women: We do not know

M: Are there some of you with ideas of how worms come about?

W1, W4: Others have said from the soil

M: Do you think that having such a disease is a fearful thing or not?

All: It is a fearful thing

M: How is it fearful?

W2: When you have vomited it and you bite it, you surely die

W2: They are different from the ones that come from the anus. The ones that come from the mouth are dangerous. When you bite even just at the tip end, and when it falls to the ground, you need to die yourself

M: Do you think there are other diseases more severe than intestinal worms?

W1: There are there

M: What kind of diseases?

W1: Some malarias do not come well. Like cerebral malaria is fear provoking

M: What about the least severe disease than the intestinal worms?

W2: There is no disease that is not fearsome. All diseases are fearful

W8: Diseases are fearful, like that one that touches the back of the head

W7: Cerebral malaria

W8: Cerebral malaria, epilepsy. The disease of epilepsy is bad because someone can fall on fire. All diseases are bad

W2: All diseases are bad, indeed

M: Do we think that in our village, many people suffer from this same disease?

Participants looked confused as they did not understand the question

W8: You mean malaria?

M: No, disease of intestinal worms

Most agree that there are there

W8: There are there but I would not know them well

W2: You would not know

M: Do we think having this disease is a fearful thing? If for instance we ourselves have them, we have intestinal worms, do you think it is a fearful thing?

Women are shown the pictures of proglottids

M: If we have worms, do you think it is a fearful thing?

A few: It is fearful

M: Have we ever seen those things *(referring to proglottids)* in human faeces?

W4: No, I have not seen them before

W7, W8: I have not seen

M: Have you seen them before, those whitish things?

Animated discussion ensured

W3, W4: They are white and in segments

W2: They are seen in dysentery cases

W4: Yes, the disease of dysentery is where such things come from. You can see they are sometimes mixed with blood, they are white

M: So these things, these worms are not fully grown? *(pointing on the picture)*

W7: They are the small ones!

W: The worms

W7: Yes, those small ones

W5: When you go to the bush, you can feel them dropping *(miming dropping sounds)*

M: What do you think is the danger or problem that can be there with the disease of intestinal worms?

*Silence*

M: On daily life

W4: Sometimes, a child does not grow well if he/she has this disease. The child is usually sickly...... that is a problem

M: What do others think is the danger or problem that we face if a person has intestinal worms?

W5: To look for medicine for the victim so that he/she gets better

W7: For others, the abdomen is usually swollen

W6: And the child does not grow at all

W2: A child looks...

W3: Sickly

W2: ..... Sickly and is not happy at all

M: Do we know what cysts are?

A few women: Yes

M: Do we know what cysts are?

*Silence*

M: Have we seen them before?

W8: Cysts from pork?

M: Yes, cysts from pork

Most women: Yes, we have seen them

Pictures of pork with cysts are shown the women

All: We have seen

M: Do we think this disease is only present in pigs or it is found in humans as well?

W8: Looks like this disease can also be found in humans because of the meat that we eat. When you eat meat with cysts and one goes to the stomach it can.........

W2, W3, W7: Even you can have it

W8: Yes, even you can have it

All agree

M: Do we know of anyone who has them?

Most women: No

W8: We may not know that person

W7: Only know after one has being examined and diagnosed with it

W2: That so and so was examined and has them

W6: Yes, that so and so was found with them for sure

M: What is the prevention for this disease of intestinal worms?

W8: Prevention ... ? *(and then she hesitates)*

M: Yes

W8: uuuhh

M: Shall we say how do cysts get to be present in pork?

W5: It is because there is no medication for this disease. If there was medication, this disease would not be present

W8: How I feel personally, is that the way cysts come about to be in pigs is that this animal does not choose what it eats, it eats indiscriminately. However, what food brings about that disease to be in its flesh is unknown to us

W2: Another thing; what our parents used to say long time ago, they used to say because of the brew they used to make and after sieving off the mash (*Mase*=brewers mash) from beer making and giving it to pigs was a reason the pigs developed cysts.

Mash (from the beer) = *Mase*=brewers mash

M: Do we know what epilepsy/fits are?

Most women: Yes, we know it

M: Have we heard of anyone who fits?

All: Yes

W8: Yes, we have heard of it

M: What do you think makes one to fit?

W3: That it is a disease that just comes naturally

W2 repeats the same sentiment

W1: That malaria has worsened

W7 and W8 repeat the same sentiment

W8: Others say it is as a result of being bewitched

M: Others?

W7: Those are the only reasons we know

M: Don’t we ever think that pigs can also bring such things?

W5: Pigs can bring fits because when a person has started to have fits, they are forbidden from consuming pork

Others agree

W2: They are forbidden to eat pork. They are told “the medication that I have given, you need to stop eating pork”

W7: Not eating pork

M: When you investigate why do you think it is so?

W1: We never know really as we have not investigated the matter or asked why they are forbidden from eating pork.

W4: Others are told that “after you have finished my medication that is when you can resume eating pork”

W7: No!

W8: They forbid taking pork

W7: They strictly forbid consumption of pork. They are told not to eat pork as continued consumption will result in fits not resolving

M: Where do you get the medications you have mentioned?

W8: Medication for epilepsy?

M: Yes

W2: For others, we bring them here at the clinic

W7: Others it is traditional medicine

W2: However, those that come to the clinic after being given medication they are not told to stop eating pork. After being given such medical help some get healed. It means that what they had was malaria, but for others ...

W1: They would have been bewitched

W2: You find that they go to the clinic perpetually without any relief, treatment fails but after attempting traditional medicine you find that they get better. That is when they are told “do not eat pork!”

M: Do you think this disease can be prevented?

*Silence*

W1: We may not prevent it for we don’t know how

W3: Only prevention is to stop eating pork

W8: You see, even a patient suffering from epilepsy and has been taken for traditional medication, he/she is told to stop eating pork. Therefore, for protection, one has to stop eating pork

W2: May be they researched that when someone eats it, the condition worsens. You see it when it gets sick. It has a droopy stance, lowering its head like this *(demonstrating).* They found out that for this person to get well, it is fitting that he/she stops eating pork. And you find for sure that when they are stopped from eating pork, they stop fitting and falling

M: So, does this disease get treated?

W2: Some do not get healed

W6, W7: They get well

W7: Epilepsy get treated

W2: Some get treated, while others do not

W7: They do not get better if the source is from “people”, but one has just naturally suffered from it, they get healed

“People” = i.e. being bewitched

M: Do you think having this disease is a fearful thing?

W2: Yes, it a very fearful thing

Others in unison: It is a very fearful thing!!

M: Are there other diseases more severe than this one?

W1: All diseases are fearsome

W2: But this one is very fearful

W7, W8: It is a very bad disease

M: Why is that so?

W2: When they start to fit and fall......

W5: Others mess themselves; can bite their tongue, fall, for such it can be almost dying

W5: You may find the victim has fallen while other people are not present; they can fall on fire and get burned. Like the way ... (name withheld)...got burned, from the hip and down the limbs

W5: Even that one that fell in a well, she died

W2: Yes, she fell in a well, and died

W8: There is also that wife of (name withheld) that fell and got burned. All the feet were burned, the skin was peeled off. The toes became stiff like this *(demonstrating)*

W2: Yes, her feet became stiff *(also demonstrating)*

W8: Now when she was falling and fitting, she was not aware that she was being burnt. She was alone in the kitchen. It means epilepsy is...

W7, W2: Bad

W8: It is similar to dying. When the head is like this (pointing to her head as she was making a circle which means someone is mentally unstable), one is like dead and comes back to life. For someone not to be aware that the toes are being burnt/blackened, eeh? For someone is aware even when being burnt a bit by a little fire. For her, she was being burnt until the toes became stiff like this *(demonstrating)*

W2: Like drowning, too. One who is aware of what is happening cannot fall on a cliff edge for he/she knows that she/he will die as a result of the fall. One can get killed if he/she falls in water. If one is at the well, one can fall in as they are fitting and drown, with water filling up the stomach until one dies. It is bad!

W7, W8: Epilepsy is very bad

M: Do we have many people suffering from this kind of disease in our village?

All: They are there in our village

W8: There are there, like the one I have spoken about. There are there. She got burned and her feet were even rotting and got crooked like this

W2: There is also.....

W8: She firstly got burnt on her hand. But now both her feet have been affected as a result of a recent incident

W2: There is also one of our in-laws who had a dislocated shoulder after falling at the roadside. The youngsters with her were scared to touch her as she was fitting and therefore did not care and look after her. So the shoulder got dislocated. She stayed here at this clinic for about four days

M: W5, you can also add on

W5: It is the same with my mother; She is the one of whom is being spoken of now. The child to my uncle who is of average height also fits

M: Do we think this disease is very serious?

All women: Yes!

W8: It is a bad disease...because it affects him/her anytime, she may be at the fireplace cooking and it takes her into the fire; she may be at the well, and the disease can take her into the well and she would fall in

W5: That one (name withheld) you know (name withheld); It is me who saved her that time when I used to stay at McKenzie’s. The whole torso had almost gone in, flipping over; I pulled her up and prevented her from falling in a well. If I was not there, she would have fallen in

W8: That is where this disease is bad, because you can see this person and think he/she is normal but she/he can fall down at any time, and what happens next is unusual and makes one have pity

M: What kind of problems, challenges that a victim of this disease experiences in a daily life?

W8: When the disease has become chronic, a person looks like.....

Other women: Mentally unstable

W5: Like a wayward person, just a mentally retarded person

W8: The victim’s memory becomes negatively affected

W2: You may be speaking with them, but get surprised that they retort sharply at you, they become moody and their mood swings can affect others; you just ignore them. Sometimes even when talking with you, you may end up in argument and if you join in and answer back, you may look as if you are also confused

W7: Like that one (name withheld); she just beats you up and ignores about the beating

W1: That one (name withheld) really beats people

W8: At last like the other one (name withheld). The disease makes someone look like an insane person, mentally disturbed

W2: Like the other girl, she is like mad now. She just laughs about, stops and laughs again after some time. She even follows women going to draw water from the stream even when she does not have a bucket. When you ask her where she is going, she just laughs. The victim gets confused because the disease becomes chronic

**NOTE: All the withheld names are of different persons**

M: In order to prevent these diseases in pigs, those diseases coming from pigs, like intestinal worms and epilepsy, how do you think we can prevent them?

W7: In order to prevent them, it is just to stop eating pork

M: Prevention like the first method is that we need to build kraals such that pigs do not go out, or are confined. How do we think about it?

W7: One can easily build a kraal, but pigs in our village are difficult

W1: They break the kraal to go outside because they are used to staying outside and eating anything

W7: Even the mother of (name withheld) confined a pig for about two weeks. The pig looked sick afterwards with its head lowered like this. After releasing it from the kraal, it is now alright

W4: They want the outside

W2: Our *Nsenga* pigs seem like they are difficult ones. They cannot be compared to the white man’s pigs (i.e. exotic breeds); when the exotic pigs are confined they grow in body weight. I do not know about our kind of pigs, how they were created

M: What if we said everyone in the village should build a toilet and that no one should be going to the bush, what do you think about it?

W5: It can happen

W8: Building toilets can be possible, it can happen, but not everyone would manage doing it

W4: Laziness

W8: There are others who say that when they go in the toilet the urge to defaecate goes away. Those same ones are the ones that go to the bush. They insist that “when they use the toilet, the urge goes away but I feel good when I go to the bush”

W2: For some .....

W4: For others I think it is laziness. They cannot build their own toilet.

W2: It is laziness to build their toilet

W4: They rush to use the toilet you yourself have built. You find quickly, they rush to use your toilet

W2: In fact, people are just difficult, especially our menfolk who drink *Chibuku* over there. When they are asked to build a toilet they respond that “hey you! Do not make noise for me”. So, how can you as a woman build a toilet?

Chibuku = an opaque beer made from malt/maize etc

W1: He would say this is the rainy season

Other women: Dry season (postharvest)

W1: It is postharvest, let me enjoy some beer. After several reminders, you let him be

M: What about if we said we need to cook pork adequately by boiling it for a long time, what can we say about it?

W8: We can be cooking that meat, but the problem is with those that practise “Shokas”.

Shokas = the young men who trade in a pork or goat meat preparation on big pans at the roadside

Others women agree: Yes, those who practice *shokas*

W8: Those who do *shokas*

W3: They like the roasted meat

W8: Those same ones, especially the drunkards. When they are drunk they go where *shokas* is prepared and want to eat pieces of that meat

W2: Just upon arrival there

W8: They like the fats

W4: They love to eat even raw meat, the one not fully prepared

W2: They get a piece from the pan and after adding some salt to it they swallow it. You find that meanwhile the wife at home has properly prepared meat

W8: Yes, she would have cooked properly

W2: After eating meat at *shokas* you find them going straight to the bush to defecate

M: What about if we said every animal before and even after it has been slaughtered, it has to be examined by veterinarians who look after health of animals, how can it be?

All: It can be a good thing!

M: How can it be a good thing, what is the reason?

W3: They need to be examined; they need to be given medication

W2: So that we would know that what we are cooking is a good thing; even for me to go up and buy *Shokas* meat, I would have known that that meat is good. If they have to get and after adding salt and swallowing it *(making reference to drunkards like in her earlier submission),* we would know that it is good meat

W4: May be diseases can be reduced, the one of fitting (epilepsy)

M: What can you say about washing fresh vegetables? I think for *Chiwawa*, most of us do not wash it many times

Chiwawa = pumpkin leaves

W7: We fear that *chiwawa* can dry up, and so we just cook it like that

W1: If you have plucked it and it has no dirt, you may wash it

W7: The one without dirt, is not washed

W2: You get it...

W8: Speaking truthfully, we do not wash *Chiwawa*; especially that one that grows in the rainy season and creeps on support like trees or stalks, we just pluck it, and not wash it all.

W7: We do not wash it

W8: We just slice it and put in a cooking pot

W3: When you wash it, it becomes watery and spoiled

W2: It becomes watery and spoiled (shrivelled) even if you have added cooking oil! The leaves will be dry, toooo! *(Exclaiming to emphasize the dryness of the leaves)*

M: What about rape?

Rape = also a vegetable, but a commercial one

All women: We wash rape

M: Why do you do it?

W5: Because it has dirt. When it is being watered, dirty water from the ground splashes on the leaves

W8: And because of the chemicals they apply on them. That is why we wash it to remove them before we slice the leaves into pieces. We wash it thoroughly in water, cut it up and then cook it

W1: There are also insects like aphids on the leaves

M: What about if they say one way of prevention of epilepsy and intestinal worms is to wash up properly, hygiene, not so?

All: Yes

M: What do you think about that?

W8: That can be good. Now for some, they do not manage to bathe children every day. You find 2, 3 or 4 days pass before bathing a child

W1: For others, they only bathe kids when new clothes have been bought

W6: So that a child wears new clothes

W1: Like in these parts, they would say “I will bathe the child so that this coming Sunday, the child wears new clothes”

W2: Now looking at the child’s feet...

W6: The feet are scaly and cracked

W7: For others, when they want to go somewhere, they assign a child, but one who is slightly older, with a height like this *(measuring height)* to bathe fellow children *(siblings)*. Now like that a child is not properly bathed at all

M: What if they said, all who have diseases or intestinal worms need to be given medications?

All women: It can be a good thing!

M: How can it be a good thing?

W2: We need medications so that we protect ourselves so that those ailments and bad symptoms someone is feeling cease

W3: So that that person gets better

M: What about if we talk about the issue of vaccination? That all the pigs that we have also received vaccination

All women: It can also be a good thing

M: Why?

W5: So that disease are prevented

W8: So that even when we slaughter the animals we would be eating the meat freely

W5: All of them would need to be vaccinated

M: Who should pay so that those pigs should be inspected or examined? I believe that you know that for pigs to be examined or to receive injections one needs to pay even something not matter how little?

W2: It can be manageable

Others agree

W8: Yes, the owners of the pigs themselves

W2: We can feel good about it

W1: You would just need to inform the chief about it, the chief would issue a decree that anyone with a pig should prepare some money

W2: Yes, that they should prepare some money so a doctor can give them drugs

W8: So that the vet can come to give medicines to these same pigs

W2: That can be a very good thing, we can be very happy

Ruth summarized at 13:41h

M: I believe that we have reached the end of our discussion. From the beginning, we talked about the goodness and badness of pigs, how we keep and feed our pigs, we have also discussed some diseases that can arise as a result of eating pork, and how we can prevent or protect against these diseases.

Is there any question or any additions of any kind? We are free to add on

W1: I can add something! We are very happy to have come here. We are going to tell our friends who could not come that for a pig to be good you need to do certain things. And also that pigs have such and such diseases. I have finished

W2: Another thing that we can add on is that we have heard about epilepsy and how it can arise, how our friends suffer from it. We are free such that when we go to the village we are going to explain to our friends that listen “these diseases arise as a result of this and that, and in order to protect against them you need to do certain and certain things”. Thank you

W8: One thing I need to ask about is: are these same cysts in pork the same ones that cause epilepsy?

R: Our agreement is that after we have shared these views we will call up a meeting where we are going to discuss and explain all those things. We are going to offer suggestions that in certain aspects these are the things that you need to do, these are good and these other aspects are not good and that what causes diseases are these and these things.

We have heard all that you have spoken about. We will call up all the women, men and children together and at meeting we will reach a resolution and what causes diseases are such and such things.

Is it possible that you wait for a few days?

All Women agree

M: We need therefore to be alert as women bearing in mind that we will need to go again at Kakwiya

W5: We are very grateful

**The End**
